# The Transcriptome of the Nosocomial Pathogen *Enterococcus faecalis* V583 Reveals Adaptive Responses to Growth in Blood

**DOI:** 10.1371/journal.pone.0007660

**Published:** 2009-11-04

**Authors:** Heidi C. Vebø, Lars Snipen, Ingolf F. Nes, Dag A. Brede

**Affiliations:** 1 Laboratory of Microbial Gene Technology and Food Microbiology, Biotechnology and Food Science, The Norwegian University of Life Sciences, Ås, Norway; 2 Section for Biostatistics, Department of Chemistry, Biotechnology and Food Science, The Norwegian University of Life Sciences, Ås, Norway; Charité-Universitätsmedizin Berlin, Germany

## Abstract

**Background:**

*Enterococcus faecalis* plays a dual role in human ecology, predominantly existing as a commensal in the alimentary canal, but also as an opportunistic pathogen that frequently causes nosocomial infections like bacteremia. A number of virulence factors that contribute to the pathogenic potential of *E. faecalis* have been established. However, the process in which *E. faecalis* gains access to the bloodstream and establishes a persistent infection is not well understood.

**Methodology/Principal Findings:**

To enhance our understanding of how this commensal bacterium adapts during a bloodstream infection and to examine the interplay between genes we designed an *in vitro* experiment using genome-wide microarrays to investigate what effects the presence of and growth in blood have on the transcriptome of *E. faecalis* strain V583. We showed that growth in both 2xYT supplemented with 10% blood and in 100% blood had a great impact on the transcription of many genes in the V583 genome. We identified several immediate changes signifying cellular processes that might contribute to adaptation and growth in blood. These include modulation of membrane fatty acid composition, oxidative and lytic stress protection, acquisition of new available substrates, transport functions including heme/iron transporters and genes associated with virulence in *E. faecalis*.

**Conclusions/Significance:**

The results presented here reveal that cultivation of *E. faecalis* in blood *in vitro* has a profound impact on its transcriptome, which includes a number of virulence traits. Observed regulation of genes and pathways revealed new insight into physiological features and metabolic capacities which enable *E. faecalis* to adapt and grow in blood. A number of the regulated genes might potentially be useful candidates for development of new therapeutic approaches for treatment of *E. faecalis* infections.

## Introduction

*Enterococcus faecalis* is a common resident of the gastrointestinal tract of humans [1]. This bacterium displays a rough physiology that enables it to withstand oxidative stress [2] and harsh conditions such as high pH and salt concentrations [3]. *E. faecalis* is also an opportunistic pathogen, ranked among the leading causes of nosocomial infections worldwide [4]. Enterococci constitute the third most prevalent pathogens isolated from bloodstream infections, and represent the most frequent cause of surgical-site infections in intensive care units [4]. In the United States, *E. faecalis* accounts for approximately 80% of all enterococcal nosocomial infections [5].

*E. faecalis* V583 (referred to as V583 hereafter) originates from a patient suffering from a persistent bloodstream infection, and it was the first vancomycin-resistant clinical isolate reported in the United States [6]. V583 is part of the high risk clonal complex 2 [7], [8], which comprises mostly of isolates derived from hospital infections world wide. The genome of V583 contains several virulence related genes [8], including several antigens such as *E. faecalis* antigen A (EfaA) [9], and two well characterized antigenic exopolysaccharides; the serotype 2 capsular polysaccharide (*cps*) [10], [11], and the enterococcal polysaccharide antigen biosynthesis cluster (*epa*) [12], [13]. It has been acknowledged that *E. faecalis* acquires genetic traits by horizontal gene transfer, which includes virulence and antibiotic resistance determinants, to survive and persist in complex environments such as different infection sites (reviewed in [14]). However, *E. faecalis* pathogenesis most likely involves an orchestrated interplay between the regulation of virulence factors and multiple genetic traits that govern adaptation of the bacterial cell physiology during the process of infection. Several functional studies have been performed to link genetic traits to virulence [10], [15]–[21], but few studies have examined such genome wide transcriptional interplay in *E. faecalis*.

Even though *E. faecalis* is a clinically significant pathogen implicated in different types of infections, little is known regarding the molecular mechanisms involved in the adaptive process this bacterium undertakes to permit survival and growth in e.g. the bloodstream of an infected patient. Several studies have demonstrated that *E. faecalis* has evolved opportunistic strategies to sense and respond to entrance into the bloodstream of a host [22]–[26]. To improve the current understanding of *E. faecalis*' ability to cause bloodstream infections, we performed a genome wide transcriptional analysis of V583 during growth in 2xYT supplemented with 10% blood (YTB). We have employed a biphasic approach, performing a time-course experiment to examine the immediate responses of *E. faecalis* to blood as a biological cue, but also to explore the adaptation of *E. faecalis* in the presence of blood. Secondly, to increase our knowledge regarding the initial phase of an *E. faecalis* bloodstream infection, growth capacity and transcriptome responses in 100% blood were assessed. These experiments revealed that both growth in the presence of a small percentage (10%) of blood, and pure blood alters the transcription of the bacterium extensively. The results presented here provide new insights into processes essential for the survival and growth of *E. faecalis* in the complex blood environment.

## Results

### Growth of V583 in Blood and YTB Compared to 2xYT-Culture Medium

*E. faecalis* is able to establish a persistent infection in the bloodstream and internal organs of an infected host [27], [28], and it is of utmost importance to understand the mechanisms that enable *E. faecalis* to survive in this complex growth environment. A rich laboratory medium (2xYT) was selected as the reference culture medium since it is considered to contain minuscule amounts of infection relevant biological cues [24]. Initial experiments were performed to assess growth and behavior of V583 in 2xYT, in 2xYT supplemented with different concentrations of blood, and in pure blood. Based on these experiments it was decided to use 10% blood in 2xYT (YTB) and 100% blood in the subsequent transcriptome profiling experiments. Since *E. faecalis* is known to sense and respond to target cells such as erythrocytes, e.g. by expressing virulence factors like the toxin cytolysin [23], we decided to use whole blood rather than serum or plasma to mimic the *in vivo* environment and to examine other responses possibly modulated by erythrocytes.

**Figure 1:**
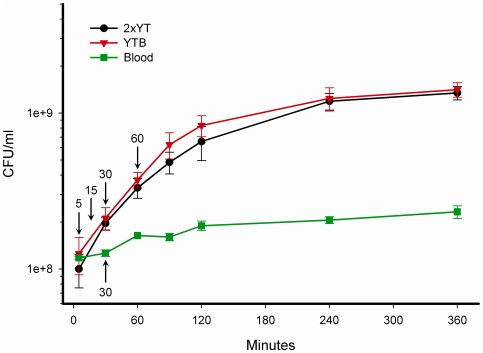
Characterization of *E. faecalis* V583 growth in 2xYT, YTB and blood. Growth of *E. faecalis* V583 was determined from cultures pre-cultivated in 2xYT, and transferred to a fresh medium (either 2xYT, YTB or blood). The growth curves are represented by colony forming units per millilitre (CFU/ml) on the Y-axis, and minutes as indicated on the X-axis. The growth curves correspond to the mean ± STD of three individual experiments. Arrows indicate the time points when samples were harvested prior to RNA extraction. Samples from 2xYT were harvested either only after 30 minutes (when compared to blood), or after 5, 15, 30 and 60 minutes (when compared to YTB).

The morphology of V583 was examined by light microscopy, revealing that it grew in chains consisting of up to 8 cells in 2xYT. It was also evident that in the presence of blood, bacterial cells aggregated, probably due to agglutination. In order to obtain reliable colony forming units (CFU) counts, gentle sonication was applied to break up aggregates and long chains of V583 cells. Several tests were performed to ensure that the aggregates and chains were properly dissolved without affecting the viability of the cells. V583 was cultivated in 2xYT until mid-exponential growth phase (cell density ∼1×10^8^ CFU/ml), prior to exposing the cells to the test conditions; pre-warmed 2xYT, YTB or blood, as described in the Materials and Methods section. The average growth curves of V583 grown in 2xYT, YTB and blood measured by CFU counts are presented in Fig. 1.

The doubling time (Td) of V583 was similar for growth in 2xYT and YTB, with a Td of 39.7 and 36.8 minutes respectively. However, growth of V583 in blood was constrained compared to in 2xYT with a Td of 80.5 minutes. When grown in 2xYT or YTB, V583 reached a maximum cell density of 1×10^9^ CFU/ml, whereas in blood V583 reached approximately 2×10^8^ CFU/ml. Based on these results, it was decided to investigate transcriptional responses after 5, 15, 30 and 60 minutes growth in YTB, and after 30 minutes growth in blood as indicated in Fig. 1.

### Global Adaptation of the V583 Transcriptome Reveals Changes Comprising Most Functional Gene Categories

**Figure 2:**
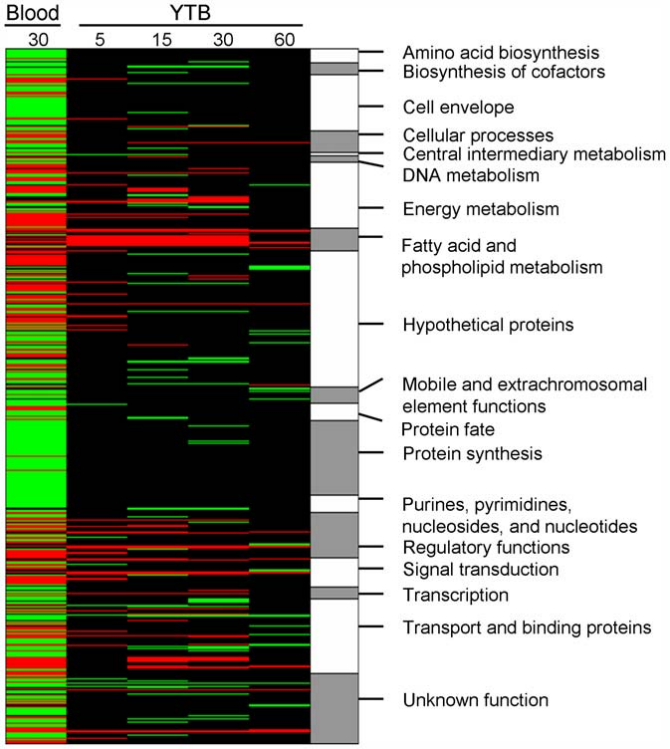
Heat map visualizing regulated genes in V583 grown in blood and YTB compared to growth in 2xYT. Genes found to be significantly regulated are indicated by either red (up-regulated), or green (down-regulated). Genes regulated after growth for 30 minutes in blood, compared to in 2xYT are listed in column 1. Genes regulated during growth in YTB compared to 2xYT are listed in columns 2–4, time-points in minutes indicated on the top of each column. The genes are sorted alphabetically by functional categories (column 5).

To examine *E. faecalis*' immediate response to blood as a biological cue and adaptation to a prolonged existence in blood, a time-course experiment was carried out. The time points for cell harvesting followed by RNA isolation (within 60 minutes after addition of blood), were carefully chosen to reflect the actively growing V583 cells in both 2xYT and in YTB (Fig. 1), as well as to portray the different stages of adaptation that V583 undergo upon the first encounter of blood. To further examine the *E. faecalis* adaptation towards persistence in blood, the transcriptional response of V583 grown for 30 minutes in blood was assessed. The obtained log_2_-ratios and p-values for all the V583 genes found during exposure to YTB and blood compared to 2xYT are listed in Table S1. Statistical analysis using a mixed model [29] combined with a stringent Bonferroni corrected confidence level of p<0.05, identified 148 significantly regulated genes during growth of *E. faecalis* in YTB. Of these, 72 genes were up-regulated, 73 where down-regulated and 3 genes where both up- and down-regulated at one or more time points. The most pronounced transcriptional responses to YTB occurred after 15 and 30 minutes. When V583 was grown in blood for 30 minutes, a total of 549 genes were differentially expressed (225 genes were up-regulated and 324 were down-regulated). The heat map in Fig. 2 presents an overview of the regulated genes within each functional category in the YTB and blood experiments. This revealed similarities in the expression patterns of a number of genes between the YTB and the blood experiments, e.g. genes involved in fatty acid and phospholipid metabolism, energy metabolism, transcription, genes with a regulatory function and genes encoding proteins with binding and/or transport functions (Fig. 2). Growth in pure blood caused additional transcriptional responses within several gene categories such as the cell envelope, protein synthesis and amino acid biosynthesis, which can be seen as a distinct pattern in the heat map (Fig. 2). The down-regulation of several genes within the latter two functional groups most likely reflects the reduced growth rate in blood compared to in 2xYT (Fig. 1).

### Stress Response of V583 Caused by Blood Exposure

Several of the V583 genes associated with stress protection mechanisms in enterococci were found to respond to blood, while only a few stress-related genes were regulated in response to growth in YTB as expected. For growth in blood an operon (EF0076-81) which includes the *gls24* and *glsB* genes [30], [31], was up-regulated (Table 1 and S1). The Gls24 protein appears to be implicated in both stress protection and virulence of *E. faecalis* [30], [32]. Exposure to blood also mounted universal stress protection genes, including EF1084 and the general stress operon *gspA1-2* (EF1810-11). A gene encoding a cold shock protein *cspC* (EF1991) was found to be up-regulated in both blood and in YTB after 15, 30 and 60 minutes, which indicates that this gene might be important for V583 to overcome the stress triggered by growth in blood. Interestingly, in response to growth in blood we also observed an up-regulation of EF1560, a hypothetical gene that has been reported to show an enhanced transcription under six different stress conditions in previous studies [33], [34].

**Table 1:** Genes proven or predicted to be important for virulence in V583 found to be regulated during growth in blood.

ORF #	Function (Gene name)	Characteristic	log_2_	Reference
EF0079	*gls24*	Stress response	3.9	[30], [32]
EF0169	lipase/acylhydrolase	Fatty acid and phospholipid degradation	2.9	[97]
EF0361	chitinase, family 2	Cell envelope	4.2	[8]
EF0362	chitin binding protein, putative	Cell envelope	4.4	[8]
EF0373	sensor histidine kinase	Signal transduction	3.0	[12]
EF0394	secreted antigen, putative (*salB*)	Protein fate	−1.6	[8]
EF0453	OsmC/Ohr family protein (*ohr*)	Oxidative stress response	3.2	[41], [42]
EF0463	superoxide dismutase, Mn (*sodA*)	Oxidative stress response	1.7	[38]
EF0606	Dps family protein (*dps*)	Oxidative stress response	2.2	[41]
EF0956	beta-phosphoglucomutase (*malB/bopB*)	Energy metabolism	2.5	[54]
EF0957	glycosyl hydrolase, family 65 (*malA/bopA*)	Energy metabolism	2.5	[54]
EF0958	PTS system, IIABC components (*malT*)	Signal transduction	2.2	[54]
EF1051	sensor histidine kinase (*etaS*)	Signal transduction	−2.6	[70]
EF1211	NADH peroxidase (*npr*)	Oxidative stress response	2.8	[41]
EF1586	NADH oxidase (*nox*)	Oxidative stress response	−2.4	[41]
EF1608	cardiolipin synthetase, putative	Fatty acid and phospholipid biosynthesis	2.5	[98]
EF2074	ABC transporter, ATP-binding protein (*efaC*)	Transport and binding proteins	3.6	[9], [12]
EF2075	ABC transporter, permease protein (*efaB*)	Transport and binding proteins	2.4	[9], [12]
EF2076	endocarditis specific antigen (*efaA*)	Cellular processes	1.4*	[9], [12]
EF2167	glycosyl transferase, group 2 family protein	Cell envelope	−2.1	[8]
EF2170	glycosyl transferase, group 2 family protein	Cell envelope	−2.2	[8]
EF2174	conserved domain protein	Hypothetical proteins	−1.7	[8]
EF2177	bacterial sugar transferase (*epaR*)	Cell envelope	−2.7	[8], [20]
EF2178	membrane protein, putative (*epaQ*)	Cell envelope	−2.8	[8], [20]
EF2179	conserved hypothetical protein (*epaP*)	Hypothetical proteins	−3.1	[8], [20]
EF2180	glycosyl transferase, group 2 family protein (*epaO*)	Cell envelope	−3.0	[8], [20]
EF2181	glycosyl transferase, group 2 family protein (*epaN*)	Cell envelope	−2.9	[8], [20]
EF2182	ABC transporter, ATP-binding protein (*epaM*)	Transport and binding proteins	−2.5	[8], [20]
EF2183	ABC transporter, permease protein (*epaL*)	Transport and binding proteins	−1.9	[8], [20]
EF2189	conserved hypothetical protein (*epaJ*)	Hypothetical proteins	−2.0	[8], [20]
EF2190	glycosyl transferase, group 2 family protein (*epaI*)	Cell envelope	−1.9	[8], [20]
EF2192	dTDP-glucose 4,6-dehydratase (*epaG*)	Cell envelope	−1.7	[8], [20]
EF2194	glucose-1-phosphate thymidylyltransferase (*epaE*)	Cell envelope	−2.2	[8], [20]
EF2195	glycosyl transferase, group 2 family protein (*epaD*)	Cell envelope	−1.9	[8], [20]
EF2197	glycosyl transferase, group 2 family protein (*epaB*)	Cell envelope	−1.8	[8], [20]
EF2198	glycosyl transferase, group 4 family protein (*epaA*)	Cell envelope	−2.1	[8], [20]
EF2439	undecaprenol kinase, putative	Toxin production and resistance	−3.2	[8]
EF2485	ABC transporter, permease protein (*cpsK*)	Transport and binding proteins	−2.9	[10]
EF2486	ABC transporter, ATP-binding protein (*cpsJ*)	Transport and binding proteins	−3.5	[10]
EF2487	UDP-galactopyranose mutase (*cpsI*)	Cell envelope	−2.4	[10]
EF2488	lipoprotein, putative (*cpsH*)	Cell envelope	−2.5	[10]
EF2489	MurB family protein (*cpsG*)	Cell envelope	−2.9	[10]
EF2490	conserved hypothetical protein (*cpsF*)	Hypothetical proteins	−3.2	[10]
EF2491	glycosyl transferase, group 2 family protein (*cpsE*)	Cell envelope	−1.9	[8], [10]
EF2492	glycosyl transferase, group 2 family protein (*cpsD*)	Cell envelope	−2.1	[8], [10]
EF2658	FemAB family protein	Toxin production and resistance	−1.7	[8]
EF2713	cell wall surface anchor family protein	Cell envelope	2.8	[8]
EF2738	thioredoxin reductase (*ahpF*)	Oxidative stress response	−1.8	[41]
EF2748	basic membrane protein DtlB (*dltB*)	Cell envelope	−2.2	[52]
EF2749	D-alanine-activating enzyme, putative (*dltA*)	Cell envelope	−2.9	[52]
EF2795	LysM domain lipoprotein	Cell envelope	−2.0	[8]
EF3082	iron compound ABC transporter (*fatB*)	Transport and binding proteins	3.5	[8]
EF3106	peptide ABC transporter, peptide-binding protein	Transport and binding proteins	6.1	[8]
EF3164	PilB family protein (*msrB*)	Oxidative stress response	2.1	[41]
EF3191	lipase, putative	Fatty acid and phospholipid degradation	2.3	[8]
EF3198	lipoprotein, YaeC family	Cell envelope	3.2	[8]
EF3245	cell-envelope associated acid phosphatase	Enzymes of unknown specificity	−1.8	[8]
EF3256	pheromone cAD1 precursor lipoprotein	Cell envelope	−2.9	[8]
EF3257	oxidoreductase	Oxidative stress response	−4.4	[41]
EFC0001	pheromone binding protein (*prgZ*)	Transport and binding proteins	1.6	[8]

**Figure 3:**
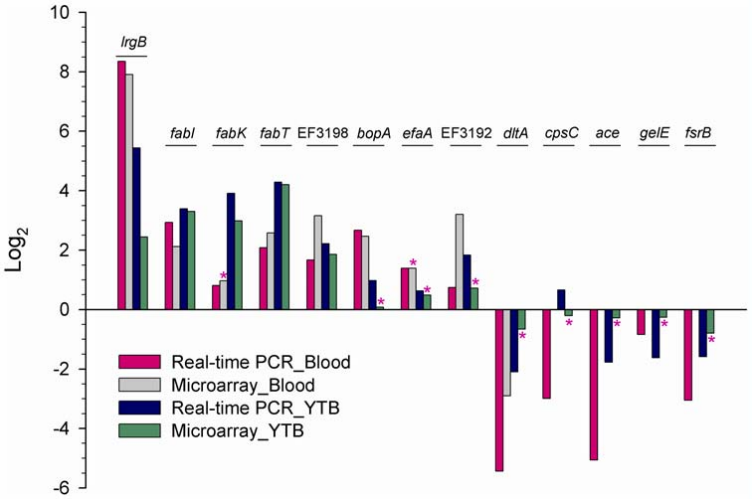
Gene regulation (log_2_) after 30 minutes growth in either blood or YTB compared to 2xYT analyzed by microarray or real-time quantitative RT-PCR. The asterisks indicate values from the microarray experiments that were found to be outside the rejection level. The corresponding orf-numbers for the genes tested are: *lrgB*; EF3193, *fabI*; EF0285, *fabK*; EF2883, *fabT*; EF2886, *bopA*; EF0957, *efaA*; EF2076, *dltA*; EF2749, cpsC; EF2493 ace; EF1099, *gelE*; EF1818, *fsrB*; EF1821.

*The corresponding p-value was above the chosen level of statistical significance. However the up-regulation of this gene was confirmed by real-time QPCR (Fig. 3).

During the course of infection, bacteria are exposed to massive oxidative stress [35], [36]. The microarray results revealed that the expression of several genes associated with oxidative stress response in *E. faecalis* was affected by blood exposure. Interestingly, previous studies have established a relationship between the oxidative stress response and virulence in *E. faecalis* [37]–[40]. A total number of nine oxidative stress protection genes [41] were regulated during growth in YTB or blood (Table 1 and S1). Five of these genes were up-regulated in response to growth in blood, including an organic hydroperoxide resistance gene *ohr* (EF0453) [42], the *dps* gene (EF0606) predicted to protect DNA from oxidative damage [8], the NADH peroxidase *npr* (EF1211), the peptide methionine reductase *msrB* (EF3164), and the superoxide dismutase gene *sodA* (EF0463). The *sodA* gene is important for *E. faecalis* to survive ingestion by macrophages [38] and was also up-regulated in *Streptococcus agalactiae* during growth in blood [43]. Four genes related to oxidative stress were found to be down-regulated in response to growth in blood (Table 1 and S1). Two of these genes, an oxidoreductase (EF3257) and a thioredoxin reductase *ahpF* (EF2738), were down-regulated both during growth in blood and YTB. An NADH oxidase *nox* (EF1586) was also found to be down-regulated during growth in blood, and an alkyl hydroperoxide reductase *ahpC* (EF2739) showed a reduced expression after 15 minutes growth in YTB.

### Exposure to Blood Induces Modifications to the Cell Envelope

The integrity and composition of the cell envelope of an invading bacterium is important to evade the challenges evoked by the host defense systems [44], [45]. A number of changes in the transcriptome of *E. faecalis* imply extensive adaptations in the cell membrane composition and surface related structures (Table S1). A particularly pronounced change was detected in two gene clusters (EF0282-84 and EF2886-75), responsible for type II fatty acid biosynthesis (FASII) and isomerization of membrane phospholipids. These loci were up-regulated throughout the time-course of the YTB experiment and also in response to growth in blood. Interestingly, these gene clusters have previously been shown to be regulated in response to exposure to the cell membrane detergents SDS and bovine bile [46], indicating that remodeling of the fatty acid composition in the cell membrane might be an important response to a broad range of external stressors. Furthermore, the up-regulation of the cardiolipin synthase gene EF1608 (Table 1 and S1), which modulates the phosphatidylglycerol content in the cell membrane, also suggests that exposure to blood affects the membrane composition of *E. faecalis*. Evidence of lipolytic activity in response to growth in blood was substantiated by the up-regulation of two lipases (EF0169 and EF3191) (Table 1 and S1). The latter gene did also show an enhanced expression after 60 minutes growth in YTB and it has been proposed by Paulsen *et al* [8] as a potential virulence factor.

The *lrgAB* operon (EF3194-93) that encodes a putative lysis inhibitory system was highly up-regulated in response to blood (Table S1) with a log_2_-value of 7.9 for *lrgB* and 5.6 for *lrgA*. The two genes were also found to be highly up-regulated throughout the time-course experiment in YTB. The rapid induction and consistently high level of transcription of the *lrgAB* operon, in addition to its putative function suggests a role in modification of the cell wall structure, which might be propitious for growth in blood. Noticeably, the synonymous genes (gbs0182-83) in *S. agalactiae* showed an increased expression during growth in blood [43]. The expression of *lrgAB* in *Staphylococcus aureus* is regulated by the closely located LytSR two-component system [47]. In V583, a two-component system (EF3197-96) homologous to the LytSR system in *S. aureus*, resides directly upstream from *lrgAB*. It is possible that this system is involved in the regulation of *lrgAB*, but only a modest enhanced expression (not statistically significant) of this two-component system was observed.

*E. faecalis* contains two gene clusters responsible for the production of two serotype-determining exopolysaccharides: the serotype 2 capsular polysaccharide (*cps*) [10], and the enterococcal polysaccharide antigen biosynthesis cluster (*epa*) [13]. It has previously been shown that the *cps* and *epa* clusters affect virulence in mice [48]–[50] and also contribute to resistance against phagocytic killing [10], [20], [51]. In our experiments most of the genes within the *epa* cluster (EF2200-2189 and EF2184-77) and the *cps* cluster (EF2492-84) were down-regulated during growth in blood (Table 1 and S1). In addition, some of the *cps* genes were also down-regulated after 15 and 30 minutes growth in YTB. These results are consistent with previous work on *E. faecalis* FA2-2, which showed that genes in the *cps* locus were down-regulated during growth in serum [11].

The V583 genome contains an operon, *dltABCD* (EF2749-46), responsible for incorporating d-alanine into cell-wall associated teichoic acids and lipoteichoic acids [52]. The first two genes of this operon, *dltA* and *dltB*, were found to be down-regulated in response to growth in blood (Table 1 and S1). Reduced content of D-alanine esters in the teichoic acid results in an increased net negative charge on the bacterial cell surface, which in turn can affect several bacterial properties such as susceptibility to cationic antimicrobial peptides and biofilm formation [52]. In addition, the expression of a genetic locus known to be involved in biofilm formation and maltose metabolism [53], [54] showed an enhanced expression in response to growth in blood (Table 1 and S1). This locus includes one operon, the *bopABCD*/*malPBMR* operon (EF0957-54), and a phosphoenolpyruvate phosphotransferase system (PTS) *malT* (EF0958) of which the genes *malT* and *bopAB/malPB* were up-regulated in blood. The genes *bopCD/malMR* showed the same trend, although not statistically significant. Furthermore, the secreted antigen *salB* (EF0394) showed reduced expression during growth in blood, and after 5 minutes growth in YTB (Table 1 and S1). This gene has also been demonstrated to be important for biofilm formation [55].

It has been proposed that lipoproteins are implicated in virulence in *E. faecalis* [56]. Interestingly, we found that the transcription of nine genes encoding lipoproteins were regulated (five up-regulated and four down-regulated). Among these, the cAD1 conjugation pheromone precursor (EF3256) showed decreased expression in blood and after 15 minutes in YTB (Table 1 and S1), while transcription of an operon encoding an ABC-transporter and an YaeC family lipoprotein (EF3200-EF3198) was elevated in blood and after 15 and 30 minutes in YTB (Table 1 and S1). The cAD1 conjugation pheromone precursor and the YaeC family lipoprotein have both been predicted by Paulsen *et al* to contribute to virulence in V583 [8]. Furthermore, twelve putative membrane-protein encoding genes were affected by growth in blood (six up-regulated and six down-regulated), and three glycosyl transferase genes were down-regulated. We also found a down-regulation of eight genes involved in peptidoglycan biosynthesis. Noticeably, we observed an up-regulation of two genes encoding chitin binding proteins (EF0361and EF0362) and a gene encoding a cell wall surface anchor family protein, EF2713 (Table 1 and S1), all three proposed as potential virulence factors by Paulsen *et al* [8].

### Adaptive Metabolic Shift during Whole Blood Exposure

A massive transcriptional response was observed for genes involved in metabolism when V583 was grown in blood or YTB compared to 2xYT, which could be expected from the results of the growth experiments (Fig. 1). Accordingly, the transcriptome analysis portrayed rapid adjustments of gene expression to accommodate the changed nutritional conditions. The results revealed changed expression of genes involved in pathways in the central metabolism of V583 indicating that a wide range of alternative energy sources were utilized (Table S1).

After 30 minutes of growth in YTB or blood we found a reduced transcription of the main uptake system of glucose in *E. faecalis*, the mannose PTS *mptBACD* (EF0019-22) [57], signifying exhaustion of the glucose reservoir at this stage of the experiment. This notion was supported by the down-regulation of the *ptsHI* operon (EF0709-10), which constitutes the signal transduction components that mediate carbon catabolite repression (CCR) [58]. Furthermore, the observed down-regulation of *pfk*-*pyk* (EF1045-46) and *fba* (EF1167) involved in the first steps of the glycolysis is also consistent with depletion of intracellular glucose catabolic intermediates. Simultaneously, glycolysis genes *gap-1* (EF1526), glycerate kinase (EF2646) and *pgm* (EF2982) were up-regulated, indicating increased carbon flux from sources other than hexose sugars. Interestingly, the glycerol catabolic pathway (EF1929-27), was highly up-regulated in response to growth in blood, and was also found to be up-regulated after 5, 15 and 30 minutes growth in YTB, suggesting that glycerol and other C3-glycerides from blood serves as a source of energy.

Several metabolic systems subject to catabolite control protein A (CcpA) mediated CCR [58] were also regulated during growth in blood, indicating the use of certain amino acids and available sugars as alternative energy sources. Of these, the gene cluster responsible for citrate catabolism (EF3327-15) and three genes involved in arginine catabolism, *argF-1* (EF0105), *arcC-1* (EF0106) and EF0108 were up-regulated, while the gene-cluster involved in serine degradation (EF0097-100) showed a reduced expression. After 30 minutes growth in YTB we also found an enhanced expression of catabolism of branched chain amino acids (*ptb, buk, bdkDAB*, EF1663-59), and the same trend was found during growth in blood. Furthermore, derepression of four PTS systems regulated by CCR mediated binding of CcpA to cis-acting catabolite-responsive elements (*cre*) [58] was observed. These include the predicted cellobiose (EF0292-91), N-acetyl galactoseamine (EF0456), lactose/galactose (EF1801) and gluconate (EF3139-36) PTS systems. The latter is part of a predicted metabolic pathway consisting of two operons (EF3142-37 and EF3136-34) that facilitates gluconate uptake and catabolism via the mannonate route [58]. The expression of both operons was enhanced during growth in blood, whereas only some of these genes showed a significantly enhanced expression during growth in YTB.

Significantly altered expression was observed for six PTS systems that probably are regulated by a sigma 54 dependent PTS regulation domain (PRD) and/or Bgl antiterminator mechanisms. Induced transcription of such PTS systems requires both release of CCR and availability of the specific sugars [58]. Two sigma 54 dependent PRD controlled PTS systems, *mphAD* (EF1953-50) [57], and a putative N-acetylglucosamine PTS (EF1516), were down-regulated in YTB and blood respectively. Also, the *mpoAD* PTS system (EF2980-76) [57] was found to be up-regulated and appeared to be co-regulated with EF2982-81. Similarly, the inferred sorbitol metabolism operon (EF3310-04) was up-regulated during growth in blood. Moreover, an indication of co-metabolism of glucose and other sugars in blood was seen by several up-regulated PTS systems for which no CCR mechanism has been identified. This includes the ascorbate PTS *sgaB* (EF1128), during growth in blood and mannitol (EF0411-12) during growth in YTB. The fructose PTS system (EF0717), was up-regulated in blood, but down-regulated in YTB. The PTS mannose/fructose/sorbose (EF3029) showed a reduced expression during growth in both blood and YTB.

The observed changes in substrate utilization and metabolism influenced the pyruvate metabolic pathways. An increased expression of L-lactate dehydrogenase (*ldh-1*; EF0255) was observed in YTB, whereas the *pflAB* genes (EF1612 and EF1613) involved in formate formation were reduced during growth in blood, and after 15 minutes growth in YTB. Furthermore, a reduced expression of *adhE* (EF0900) signifies low contribution of ethanol fermentation in blood. The pyruvate dehydrogenase complex gene-cluster *pdhAB, aceF* and *lpdA* (EF1353-56) involved in acetyl-CoA biosynthesis was up-regulated during growth in blood and also after 15 minutes growth in YTB. The cell can harvest an additional ATP from acetyl-CoA by oxidation to acetate, but no change was detected in transcription of the involved genes *eutD* (EF0949) or *ackA* (EF1983). We previously mentioned up-regulation of the fatty acid biosynthesis pathway FASII, and since acetyl-CoA also serves as the precursor of the FASII pathway, it is possible that acetyl-CoA is funneled to fatty acid biosynthesis.

### Blood Specific Components Influence Transport and Biosynthesis Pathways

Iron uptake is a crucial factor in bacterial virulence and in gut colonization of commensal bacteria [59], [60]. The mechanisms that enable bacteria to acquire iron from the surroundings have thus received a lot of attention [61]. Our data reveal a potentially important role of iron acquisition and homeostasis during growth in blood. Six genes involved in iron transport, including *feuA* (EF0188), *feoB* (EF0476), *ceuBCD* and *fatB* (EF3085-82) were up-regulated in YTB. The latter four genes comprise an operon that also was up-regulated during growth in blood (Table 1 and S1). The *hrtB* (EF0793) ABC-transporter gene, which homologue in *S. aureus* facilitates expulsion of toxic surplus of heme [60] was up-regulated after 15 minutes growth in YTB. Another scarcely available co-factor is manganese, which also is essential for growth of *E. faecalis in vitro* [62], [63]. Notably, a gene cluster, *efaCBA* (EF2074-76) originally identified to encode the endocarditis associated antigen is responsible for Mn^2+^ acquisition in manganese depleted environments [64]. The notion that the ability to acquire manganese is important for growth of *E. faecalis* was sustained by the observed up-regulation of *efaCB* in blood (Table 1 and S1).

The presence of blood also changed the transcription of a number of genes encoding other transport systems in the cell. For example the up-regulation of genes encoding two sugar ABC-transporters (EF1345 and EF1344-43), further supports that V583 utilizes alternative sugars from the blood. Certain transport system encoding genes were down-regulated during growth in blood including a major facilitator ABC transporter (EF0082), amino acid ABC-transporters (EF0761-60, EF2642 & EF2649), a cell division ABC-transporter (EF1760) and a phosphate ABC-transporter (EF1756). Thirteen additional genes encoding ABC-transporters with unknown substrates showed altered transcription in response to growth in blood (3 up-regulated and 10 down-regulated), indicating the requirement for balancing numerous solutes to maintain the cell homeostasis.

Previous studies on the biosynthetic capacities and nutritional requirements of *E. faecalis* have shown that all strains investigated, including the sequenced OG1RF [65], require histidine, isoleucine, methionine, and tryptophan for growth, and that arginine, glutamate, glycine, leucine, or valine was essential for growth of some strains [66]. By comparing the genome sequences of V583 and OG1RF they appear to have similar requirements for amino acids. Since our data show that the transcription of several genes encoding oligo-peptide ABC-transporters (EF0907 and EF3110-06) was up-regulated, and the transcription of two amino acid importer genes (EF0440 and EF0635) was down-regulated (Table S1), it is possible that V583 meets its demand for amino acids by acquiring oligo-peptides when growing in the host bloodstream. This is analogous to what was observed in *Streptococcus pyogenes* in a similar study [67]. Furthermore, the increased expression of *cysK* (EF1584) implies that cysteine is not abundant in blood. Glutamine and glutamate on the other hand seems to be readily available in blood, since we observed a reduced expression of the glutamine synthase operon *glnRA* (EF2160-59), glutamate synthase *gltA* (EF2560) and transamination of aspartate to glutamate by *aspB* (EF2372) during growth in blood and/or in YTB.

During growth in blood we noticed up-regulation of an isochorismatase gene (EF3192). This coincided with a down-regulation of the EF1561-68 operon, responsible for biosynthesis of chorismate, which is a precursor of aromatic amino acids, folate and quinones. In agreement with this observation we also noticed a reduced transcription of several genes responsible for biosynthesis of cofactors, prosthetic groups, and carriers including menaquinone and ubiquinone (EF0446-50, EF3255-54 and EF3260). Noticeably, modulation of chorismate acquisition was also observed in similar experiments in *S. pyogenes* and *S. agalactiae* [43], [67], suggesting that chorismate might be involved in virulence development of these bacteria.

*E. faecalis* is prototrophic for purines and pyrimidines [66]. The down-regulation of several genes involved in biosynthesis of these compounds (e.g. EF0014, EF0058, EF1547, EF2362-61 and EF3293) might imply that the requirement for nucleotides was covered by scavenging (Table S1). However, no evidence of modulation of ribose/deoxyribose metabolism was observed, which suggests a lowered demand for nucleotides, most likely as a result of the reduced growth rate (Fig. 1).

### Virulence Traits and Regulatory Genes

Several virulence traits have been identified in *E. faecalis* [10], [15]–[21]. The origin of V583 as a nosocomial isolate, causing a persistent bloodstream infection made this strain suitable for investigating responses of virulence traits by growth in blood. As previously described, the expression of several of the loci that contribute to *E. faecalis* virulence, such as oxidative and nutritional stress management genes, capsule formation genes and genes involved in acquisition of biometals, were affected by blood. In addition, some genes predicted to be involved in virulence in *E. faecalis* were found to be regulated, and a summary of these genes can be found in Table 1.

A number of regulatory genes showed altered expression in blood-containing growth environment, in particular regulators of carbohydrate metabolism. In addition, several genes encoding TetR-repressors and other unassigned regulators were found to be differentially expressed (Table S1). V583 inherits 4 sigma factors, of which the *sigA* (EF1522) and *sigV* (EF3180) showed a changed expression in blood. The sigma factor *sigV* was up-regulated, but only one (EF0315) of its five potential target genes [68] showed an altered transcription. The fact that *sigA*, the primary sigma factor of the cell was down-regulated after 30 minutes in blood, is consistent with the observed down-regulation of the entire transcription and protein synthesis apparatus, which in turn can be ascribed the lowered growth rate in blood compared to 2xYT (Fig. 1).

Of the 18 two-component systems present in V583 [8], [69], only two of the sensor histidine kinase genes were found to be significantly regulated in blood. Transcription of *etaS* (EF1051) a sensor histidine kinase involved in stress and virulence [70] was reduced, and the expression of a sensor histidine kinase gene (EF0373) previously identified as antigenic (antigen yx84) during infection in humans [12], was highly elevated (Table 1 and S1). However, the exact functions of these two-component systems are not well characterized. The *fsr* locus is an important virulence determinant in *E. faecalis* shown to contribute to virulence in mouse peritonitits models [17]. Due to microarray spot abnormalities, regulation of selected genes including the *fsr* locus was assessed by real-time quantitative RT-PCR (described in further detail below). The RT-PCR revealed that both *fsrB* and *gelE* were down-regulated both in blood and YTB.

### Gene Regulation Examined by Real-time Quantitative RT-PCR

In order to confirm the results from the microarray experiments and to investigate the transcription of a few genes of special interest (excluded from the microarray results due to spot abnormalities), real-time quantitative RT-PCR (QPCR) was performed on 13 genes listed in Table 2. We only examined the 30 minutes time point from the YTB experiment, which is most comparable to the blood experiment. The QPCR results were consistent with the results obtained by the microarray experiments (Fig. 3). For most of the genes the QPCR produced similar or greater log_2_ ratios than the corresponding microarray results. One gene, *efaA*, showed a log_2_-ratio of 1.4 during growth in blood, but with a p-value indicating non-significance. However, the QPCR showed a log_2_-ratio identical to the microarray results supporting that this gene is in fact up-regulated. Most of the genes tested by QPCR were not found to be significantly regulated in YTB using microarrays. However, the QPCR-analysis showed equal to larger responses in terms of fold change of these genes. An exception was the gene *cpsC* which showed a slightly reduced expression when examined by microarrays, while a slightly enhanced expression was seen using QPCR. Thus, the QPCR analysis confirmed the reliability of the transcriptional data obtained from the microarray experiments, indicating that the rejection level applied on the microarray data was adequate. Furthermore, the four genes which were excluded from the microarray results due to spot abnormalities (*cpsC*, *ace*, *gelE* and *fsrB*) were all found to be down-regulated in blood when examined by QPCR.

**Table 2:** List of genes and primers (5′→ 3′) used for real-time quantitative RT-PCR.

ORF	Gene	Forward primer	Reverse primer	Reference
EF0282	*fabI*	TGATGGTTTCCTATTAGCACAAG	GTTAGGAATCGCACGTTCGG	This work
EF0957	*bopA*	CAGCGACATGGACAGCCTAC	TTGCAGGACCGTCGAGTAAA	This work
EF1099	*ace*	CGGCGACTCAACGTTTGAC	TCCAGCCAAATCGCCTACTT	[24]
EF1818	*gelE*	CGGAACATACTGCCGGTTTAGA	TGGATTAGATGCACCCGAAAT	[24]
EF1821	*fsrB*	TGCTCAAAAAGCAAAGCCTTATAA	GATGACGAGACCGTAGAGTATTACTGAA	[24]
EF2076	*efaA*	TGGGACAGACCCTCACGAATA	CGCCTGTTTCTAAGTTCAAGCC	[24]
EF2493	*cpsC*	GGTTGATGCCAAGAGCTCAG	GTCCCATGCCACGTCTGTAT	This work
EF2749	*dltA*	ACGCGTTTGCCACAATTAAC	GCGCAGTGCTGGTAGATGTT	This work
EF2883	*fabK*	GCTGGATTGCCTGCACCTGTCG	GGTAGCCGATGCTTCATTAGCAAGTGC	This work
EF2886	*fabT*	ACTACACGTCGATCATCTTCACTACGC	CATTACGGAGATGCACACAATCGAAGC	This work
EF3192	-	GAACTGACGGGCGTAATCTG	GTCCAAATCCGTGCCACTAA	This work
EF3193	*lrgB*	CGACAGTAGCGTTTGCGATT	ACAGCCACTAGCGAACCAAA	This work
EF3198	-	GCTGATTTAGTGGCTGTGCAA	AGCACGACCTTCATTGGTTG	This work
	*23S*	CCTATCGGCCTCGGCTTAG	AGCGAAAGACAGGTGAGAATCC	[24]

## Discussion

In the present study we describe the first microarray analysis of the global transcriptional response of *E. faecalis* to blood. Although certain aspects of *E. faecalis* virulence development in blood have been studied [23]–[25], the global gene regulation and interplay during adaptation to the blood environment remained largely unaddressed. However, *in vivo* mouse infection models in which *E. faecalis* was intravenously injected, showed that the bacterial cell number initially dropped before stabilizing at about 10^3^ CFU/ml blood for at least 7 days [27]. This indicates that *E. faecalis* has to adapt to overcome serious challenges upon entering the bloodstream of a host. With the present study we provide novel information about the gene regulation relevant to such an adaptation process.

Transcriptomics has been successfully employed for studying adaptation to blood *ex vivo* of the closely related pathogens *S. pyogenes* [67] and *S. agalactiae* [43]. As emphasized in these studies [43], [67], it is important to note that *in vitro* experiments like the one we present here, can not entirely reproduce the environment encountered by the bacterium during *in vivo* infections with respect to oxygen tension, interaction with the host immune system, the potential depletion of nutrients etc. Consequently, our results have been cautiously interpreted with respect to inherent constraints. Hence, we have used this *in vitro* model to investigate the initial adaptation phase needed for dissemination of an infection, and to explore molecular mechanisms that might be involved in the complex host-bacterium interaction in blood.

The most significant transcriptional changes found in this study include genes relevant for cell envelope structures. In the cell membrane both the fatty acid (FASII) and phosphatidylglycerol biosynthesis genes were up-regulated. This suggests that *E. faecalis* adjusts its fatty acid composition and membrane fluidity to accommodate stress imposed by blood constituents, and possibly also by involving phospholipids from the blood. A recent study demonstrated the ability of *S. agalactiae, S. pyogenes, S. pneumoniae* and *E. faecalis* to utilize free phospholipids in serum and thus overcome the FASII pathway inhibiting antibiotics [71]. Brinster *et al* [71] also showed by QPCR that growth of *S. agalactiae* in serum results in a down-regulation of the eight FASII genes tested. Moreover, other studies have shown by microarray that the gene clusters involved in FASII were down-regulated in *S. pyogenes*, but not significantly regulated in *S. agalactiae* or *S. pneumniae* during growth in blood [43], [67], [72]. Although *E. faecalis* and the three streptococcal species mentioned above show a similar phenotype when grown in serum containing FASII inhibiting drugs, it appears that the regulation of the FASII pathway is different during growth in blood. This might be due to the differences in the genetic organization of the FASII pathway between enterococci and streptococci. Another possible explanation is that *E. faecalis* processes the unsaturated fatty acids from blood in order to accommodate a lipid composition compatible with its membrane. The observed increased transcription of two lipase genes during growth in blood might imply a connection between the modulation of fatty acid and phospholipid metabolism and lipolysis/tissue tropism. A recent study showed that 71% of the invasive *E. faecalis* isolates examined produced lipase, whereas only 35% of the noninvasive isolates produced lipase indicating that lipase activity might be important for the pathogenicity of *E. faecalis* [73].

It has been shown that orally administered or intravenously injected *E. faecalis* in mice can colonize internal organs of the host [27], [28]. Moreover, Guzman and co-workers [74] showed that growth of *E. faecalis* in serum enhanced its ability to bind Girardi Heart cells, an interaction which in a later study was shown to be inhibited by incubation of the target cells with specific sugar residues. This indicated that carbohydrate antigens were responsible for the enhanced binding [75]. We observed that transcription of two capsular polysaccharides encoded by *epa* and *cps* were down-regulated during growth in blood. Inactivation of these carbohydrate antigen loci cause reduced biofilm formation [20], [76], increased susceptibility to phagocytosis [20], [51], and attenuated virulence in mice [48]. More interestingly, these exopolysaccharides appear to play a role in adherence/colonization of tissues and organs, and in immune system evasion [10], [11], [20], [50], [51]. The involvement of carbohydrate antigens in binding has been further investigated by Singh *et al*, who showed that the *epa* locus is important for adherence/colonization of tissues and organs [50]. Capsule formation is crucial for virulence in *S. pneumonia*, which undergoes phase variation in order to establish infection [77]. However, an *in vivo* transcriptome analysis of *S. pneumonia* infection in mice has revealed that the *cps* transcription was not enhanced in blood. Furthermore, this study also demonstrated that the expression of a number of virulence traits in *S. pneumonia* was body site dependent [72]. Hence, it is possible that the transcription of the *E. faecalis epa* and *cps* loci might be more pronounced in colonized organs.

The effect of serum on *E. faecalis* adherence has been studied further by Nallapareddy and Murray [78], who found that 46 different *E. faecalis* strains all showed enhanced binding to fibronectin and fibrinogen after exposure to 40% horse serum. This property was eliminated by protease treatment, which indicated that adherence was mediated by surface exposed proteins [78]. In our study most genes encoding identified or putative adhesive proteins (such as microbial surface components recognizing adhesive matrix molecules (MSCRAMMs), the collagen adhesin *ace* and aggregation substances) were either not regulated or down-regulated during growth in blood. However, we observed enhanced transcription of several genes encoding membrane proteins or lipoproteins. It is tempting to speculate that these cell envelope bound proteins also might play a role in promoting the adherence of *E. faecali*s during infection.

Analysis of the V583 transcriptome revealed signs of lytic stress in response to blood. Particularly interesting was the immense up-regulation of the *lrgAB* operon. In *S. aureus* it has been shown that the transcription of *lrgAB* is affected by carbohydrate metabolism, acid production or cell wall active antibiotics. LrgAB inhibit murein hydrolase activity, and thus counteract lysis of the cell [79], [80]. The exact function of *lrgAB* in *E. faecalis* remains elusive. Even so, the immediate and continuously high level transcription of this operon observed in the present study, and the fact that the homologous genes in *S. agalactiae* also showed an enhanced transcription during growth in blood [43], suggests an important role for *lrgAB* upon blood exposure.

Biometal limitation constitutes a significant obstacle for bacterial pathogens to establish infection in vertebrate hosts [59], [60], [64]. Lactic acid bacteria (LAB) comprise one of the very few groups of bacteria for which iron is not an essential growth factor [63], [81]. In contrast, manganese is essential to the fermentative metabolism of LAB [62], [63]. The enhanced transcription of the main manganese scavenging mechanism encoded by *efaCBA* is a clear indication that *E. faecalis* experiences manganese constraints that might restrict its growth in blood. The strong increase in the superoxide dismutase (*sodA*) expression further emphasizes the importance of an effective manganese uptake during growth in blood. These observations are particularly interesting as both *efaCBA* and *sodA* have been shown to be implicated in virulence [38], [82]. In addition, our microarray data show that genes related to iron metabolism constituted one of the major changes of *E. faecalis* adaptation to blood, although iron is not an essential requirement for growth. This suggests a potential role of iron acquisition in virulence development of *E. faecalis*. Examination of the V583 genome has revealed approximately 53 genes with an apparent function in iron homeostasis and metabolism. It was recently demonstrated that many LAB can utilize heme to perform respiratory metabolism [83], [84], and that the pathogen *S. agalactiae* requires heme for full virulence [85]. Thus, the significance of the biometals manganese and iron/heme in virulence of *E. facealis* warrants further investigation.

Previous studies have demonstrated the ability of *E. faecalis* to sense its environment and modulate cytolysin production upon blood encounter [23]. The V583 strain is deficient of cytolysin production, but is classified as a pathogen since it was isolated from a patient with a persistent bloodstream infection [6] and belongs to a high risk clonal complex consisting of nosocomial isolates [7]. Consequently, it was relevant to look at the response of virulence related genes. The V583 strain harbors an incomplete (partially deleted) *E. faecalis* pathogenicity island (PAI) [86]. From the PAI of V583 only one gene, *dps* (EF0606) involved in oxidative stress management, was up-regulated. A few genes involved in pantothenate biosynthesis, amino acid metabolism and a transposase were down-regulated, while transcription of the remaining genes within the PAI were unaffected. It is important to note that most genes in the PAI were indeed expressed, and hence might contribute to the fitness of V583, but in the experimental growth conditions examined here, no major responses in transcription of PAI-genes were revealed.

A number of pathogens employ master regulatory systems to coordinate expression of virulence factors during infection e.g. *prfA* in *Listeria monocytogenes* [87], *agr* in *S. aureus* [88] and *covRS* in *S. pyogenes* [89]. The equivalent system in *E. faecalis* is the *fsr* quorum sensing system, which controls the expression of several genes including *gelE* (encoding a gelatinase) and *sprE* (encoding a serine proteinase) [17], [90], [91]. It has previously been shown that expression of this system is sensitive to environmental conditions [24], and that its level of expression varies between different bacterial strains [92]. In our study we readily detected the *fsrB* and *gelE* transcripts by QPCR, but growth in blood caused a moderate down-regulation of these genes in V583. This appears to be the opposite effect from growth in serum by the MMH594 strain [24]. Our results demonstrate that blood did not act as a cue to enhance *fsr* expression, but rather interfered with its expression. It has been shown that the cytolysin quorum sensing pheromones interact with blood cells in a positive manner [23]. It is possible that the down-regulation was caused by the presence of blood cells in our experiments, which might have adsorbed the quorum sensing signaling pheromone. It has been proposed that the GelE and SprE proteases contribute to virulence by degrading infected tissue [93], and it is possible that *fsr* expression is more pronounced in colonized organs.

In conclusion, this study provides new insights into the adaptive process of *E. faecalis* to growth and persistence in blood. Bacteremia caused by *E. faecalis* represents a major clinical problem, thus the results presented here could be valuable for future studies devoted to the development of new therapeutic approaches for preventing or treating enterococcal infections.

## Materials and Methods

### Cultivation and Growth Measurement

The strain used in this study was the sequenced *E. faecalis* clinical isolate, V583 ([6], [8]). For all experiments V583 was streaked on a 2xYT (1% (w/v) yeast extract, 1.6% (w/v) tryptone and 1% (w/v) NaCl) agar plate and incubated at 37°C o/n. Four individual colonies from the 2xYT plate were inoculated into the same tube of 5 ml 2xYT medium and grown for 17 hours without shaking at 37°C. The culture was then diluted 1000x in 2xYT medium (pre-warmed to 37°C) and incubated until the culture reached an optical density at 600 nm (OD_600_) of 0.1.

Growth of the bacterium was monitored by counting colony forming units (CFU). A 150 ml culture grown to an OD_600_ of 0.1 as described above was split in three and centrifuged at 8000 x *g* for 3 minutes at 37°C. The cells were resuspended in either (i) 50 ml defibrinated horse blood (TCS Biosciences Ltd.), (ii) 45 ml 2xYT medium and 5 ml blood (10% (v/v) blood) or (iii) 50 ml 2xYT medium (all pre-warmed to 37°C). After thorough mixing 5 ml from each culture was immediately transferred to a fresh tube and placed on ice and the remaining culture was incubated further at 37°C without shaking. The 5 ml cultures were sonicated at an amplitude of 25% 25 seconds (5 seconds on and 5 seconds off) using a Vibra-Cell VCX-500 ultrasonic processor (Sonics) with a microtip. Immediately after sonication 500 µl of each culture was serially diluted in 0.9% (w/v) NaCl (pre-chilled) and plated on 2xYT agar plates. The whole process was repeated after 30, 60, 90, 120, 240 and 360 minutes and the experiment was performed on three consecutive days.

### Cultivation and Sampling Prior to Microarray Analysis

A pre-culture of V583 was prepared as described above. After 17 hours the culture was diluted 1000x in 450 ml 2xYT medium and incubated at 37°C until the culture reached an OD_600_ of 0.1, when the culture was split in two. A volume of 25 ml pre-warmed 2xYT medium (37°C) was added to the control culture whereas 25 ml pre-warmed blood was added to the test culture, resulting in a final concentration of 10% (v/v) blood. Samples (25 ml) from each culture were collected by centrifugation (8000 x *g* for 2 minutes at 37°C) after 5, 15, 30 and 60 minutes, and the cell-pellets were immediately frozen in liquid Nitrogen and stored at −80°C prior to RNA extraction.

A parallel experiment was designed to investigate the transcriptional response in pure blood. A pre-culture of V583 was diluted 1000x in 200 ml 2xYT medium and incubated at 37°C until the culture reached an OD_600_ of 0.1, when the culture was split in two and centrifuged (8000 x *g* for 3 minutes at 37°C). For the control culture the cells were resuspended in 100 ml pre-warmed 2xYT (37°C) whereas for the test culture the cells were resuspended in 100 ml pre-warmed blood (37°C), resulting in a final concentration of 100% (v/v) blood in the test culture. Samples (35 ml) of each culture were collected by centrifugation (8000 x *g* for 2 minutes at 37°C) 30 minutes after addition of blood, and the cell-pellets were immediately frozen in liquid Nitrogen and stored at −80°C prior to RNA extraction.

### RNA Isolation

The bacterial cells were washed with 50 ml cold 0.1xTE (for 10% blood) and 3×50 ml cold 0.1xTE (for 100% blood) to remove blood constituents prior to RNA extraction. Then samples suspended in 700 µl RLT buffer (Qiagen) were transferred to a 2 ml screw cap FastPrep tube (Qbiogene) containing 0.6 g of glass beads (≤106 µm) (Sigma) and 300 µl chloroform (Merck). Cells were lysed by vigorous shaking for 20 seconds at 6.0 m/s in a FP120 FastPrep cell disruptor (Qbiogene). After lysis the samples were placed on ice for 5 minutes before glass beads and chloroform were removed by a brief centrifugation. The aqueous phase was transferred to a new tube and centrifuged at ∼8000 x *g* for 2 minutes to remove cell debris and unlysed cells. The supernatants were removed and kept on ice in separate tubes, while the pellets were suspended in 350 µl RLT buffer, transferred to new FastPrep tubes and subjected to another round of homogenization. The two supernatants from each sample were merged and added 750 µl 96% EtOH. Total RNA was then isolated using the RNeasy Mini kit (Qiagen) according to the manufacturer's protocol. The integrity of the RNA samples was analyzed using the RNA 600 Nano LabChip kit and a Bioanalyzer 2100 (Agilent Technologies). The concentration and purity of the total RNA was measured using a NanoDrop ND-1000 spectrophotometer (NanoDrop Technologies, Inc.).

### cDNA Synthesis and Fluorescent Labeling

Total RNA was reversed transcribed using a modified version of protocol #M007 from the Pathogen Functional Genomic Resource center at The Institute for Genomic Research (TIGR: http://pfgrc.tigr.org/protocols/M007.pdf). Accordingly, 5 µg of total RNA and 20 µg of random hexamers (Invitrogen) in a reaction volume of 17.3 µl were denatured at 70°C for 10 minutes and cooled on ice for 5 minutes. Then, 6 µl of 5x First Strand buffer (Invitrogen), 3 µl of 0.1 M dithiothreitol, 20 U rRNasin (Promega), 1.2 µl of a 12.5 mM dNTP (Invitrogen) and aminoallyl-dUTP (Ambion) labeling mixture (aa-dUTP-dTTP 2∶3), and 400 U of SuperScript III reverse transcriptase (Invitrogen) were added in a total volume of 30 µl. The labeling reaction mixture was incubated at 25°C for 5 minutes and then at 42°C for 16 hours. The RNA was hydrolyzed by adding 10 µl of 0.5 M EDTA and 10 µl of 1 M NaOH. The reaction mixture was incubated at 65°C for 15 minutes, and then neutralized by adding 25 µl of 1 M Tris-HCl (pH 7.0). Purification of the cDNA was performed using Microcon YM-30 filters (Millipore) according to the manufacturer's protocol. The cDNA was dried in a vacuum centrifuge and stored at −20°C. Coupling of aminoallyl-labeled cDNA to Cy3 and Cy5 (Amersham Biosciences) was done by resuspending the cDNA in 9 µl 0.1 M sodium carbonate buffer pH 9.3. The cDNA samples were transferred to dried Cy-dye aliquots (dissolved in DMSO and dried in a vacuum centrifuge prior to labeling), mixed and incubated for 1 hour. A volume of 35 µl 100 mM sodium acetate pH 5.2 was added and unincorporated dye was removed using QIAquick PCR purification kit (Qiagen) according to the manufacturer's protocol. Finally the Cy3- and Cy5-labeled samples were mixed and dried in a vacuum centrifuge.

### Hybridization and Microarray Data Analysis

Microarray experiments were performed using whole genome *E. faecalis* V583 PCR-based microarrays described by Aakra et al [94]. Prior to hybridization, the Ultra Gaps slides (Corning) were prehybridized according to the manufacturer's recommendations. Briefly, the arrays were incubated in a prehybridization solution (3x SSC, 0.1% (wt/vol) sodium dodecyl sulphate (SDS), 0.1 mg/ml Bovine Serum Albumin (BSA), Sigma) at 50°C for 30 minutes. After prehybridization, the arrays were washed twice in distilled water (RT) for 1 minute, then at 95°C in distilled water for 2 minutes, followed by a 1 minute wash in isopropanol and the slides were dried by centrifugation (70 x *g* for 5 minutes) in an Eppendorf 5810R tabletop centrifuge. The Cy3- and Cy5-labeled cDNA samples were resuspended in 40 µl hybridization solution (3x SSC, 0.1% (wt/vol) SDS, 1 mg/ml BSA, 0.1 mg/ml Salmon Sperm DNA (Invitrogen), 50% (vol/vol) Formamide), denatured by boiling for 2 minutes and cooled at room temperature for 5 minutes. The samples were centrifuged briefly and applied to the prehybridized microarray under a 25×60 mm LifterSlip (Erie Scientific Company). The microarrays were hybridized at 42°C for 16 hours. After hybridization, the slides were washed in 2x SSC buffer with 0.1% (wt/vol) SDS for 2 minutes, followed by a wash in 1x SSC for 2 minutes, then in 0.2x SSC for 2 minutes and finally in 0.05x SSC for 1 minute, each at room temperature. The slides were dried by centrifugation (70 x *g* for 5 minutes). Three biological replicates with one dyeswap were performed for all experiments. Microarray slides were scanned at 10 µm resolution using a Model G2505B (Agilent) microarrayscanner. Fluorescent intensities and morphologies were analyzed using GenePix Pro ver. 6.0 (Axon).

### Normalization and Data Analysis

Raw data from each array was preprocessed independently. A lowess-smoothed background was subtracted from all foreground intensities, and a cross-validated lowess-method was used in an intensity-dependent normalization of every array. The log_2_ ratios for each spot were further analyzed using a mixed model [29] to detect differentially expressed genes. For YTB a mixed model was fitted to the data for each of the four sample times (5, 15, 30 and 60 minutes) separately and for blood a mixed model was fitted to the data from the 30 minutes time point. Data for the three arrays at every sample time were described by
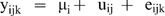
(1)


Where y_ijk_ is the observed log_2_ ratio of gene i (1,…,3502) on array j (1,2,3) and in spot k (1,…,5) on that array, µ_i_ is the expected log_2_ ratio for gene i, u_ij_ is a random effect of gene i on array j and e_ijk_ is the remaining noise. The variance components were estimated under the assumption of gaussian errors using a restricted maximum likelihood approach coping with the unbalanced data due to missing spots. Differentially expressed genes were identified by testing the hypothesis H_0_: µ_i_ = 0 against H_1_: µ_i_≠0. A chi-square test for every gene resolves this for the model in [1], [95], and a Bonferroni-corrected rejection level of p<0.05 was used throughout. If H_0_: µ_I_ = 0 was rejected, and µ_i_>0, genes were considered to be up-regulated in the cells grown in YTB and blood. If H_0_: µ_i_ = 0 was rejected, and µ_i_<0, genes were considered to be down-regulated. All data analysis algorithms were programmed in MATLAB (MathWorks inc). A gene was discarded from the final results (designated NA in Table S1) if it was replicated in less than 8 spots after filtering of the data.

### Microarray Data Accession Number

The microarray data obtained in this study has been deposited in the ArrayExpress database (http://www.ebi.ac.uk/arrayexpress/) according to the MIAME standard. The accession number is E-TABM-541.

### Real-time Quantitative RT-PCR

Real time quantitative RT-PCR (QPCR) was used to validate the expression levels for selected genes. QPCR was performed on a Rotor-Gene 6000 centrifugal amplification system (Corbett Research). The genes of interest and the corresponding primers are listed in Table 2. Total RNA used for cDNA synthesis was the same as described above (RNA samples harvested after 30 minutes growth in YTB or blood and their corresponding control samples). Synthesis of cDNA was performed using 1 µg total RNA, 6 µg random primers (Invitrogen) and 40 U RNase OUT (Invitrogen) in a reaction volume of 12.5 µl. The reaction mixture was denatured at 65°C for 10 minutes and cooled at 4°C for 5 minutes. The reaction mixture was then added 4 µl of 5x First Strand buffer (Invitrogen), 1 µl of 0.1 M dithiothreitol (Invitrogen), 1 µl of 10 µM dNTP (Invitrogen) and 140 U of SuperScript III reverse transcriptase (Invitrogen) to a total volume of 20 µl. The reaction mixture was incubated at 25°C for 5 minutes, 2 hours at 50°C, and then for 15 minutes at 70°C. Finally, 20 ng RNaseA (Sigma-Aldrich) was added to the reaction followed by an incubation at 37°C for 20 minutes. PCR amplification was performed using the recommendation by the manufacturer (with an annealing temperature of 60°C) with 2.5 µl 100x diluted cDNA in a 25-µl reaction mixture containing 12.5 µl FastStart SYBR green Master (Roche) and 7.5 µM of each primer. Standard curves with four dilutions were made in duplicates for each primer pair to calculate the amplification efficiency, and all genes were quantified in triplicates. Since the standard curves indicated a slight difference in amplification efficiencies of the different target genes and reference, differential expression was calculated by the Pfaffl method. This is an optimal method to use for calculating relative gene expression when the amplification efficiencies of the target gene and the reference gene are different since the amplification efficiency is included in the calculation [96]. The obtained C_T_ value for 23S from each sample was used as a reference for each gene in the corresponding samples.

## Supporting Information

Table S1 Microarray expression data from *E. faecalis* strain V583 during incubation in blood or 2xYT supplemented with 10% blood (YTB). Gene expression after 30 minutes (blood) or 5, 15, 30 and 60 minutes (YTB) of incubation is relative to the expression during growth in 2xYT for the corresponding time length. a) Genes comprising putative operon structures predicted by http://biocyc.org [1], [2] are marked with one color (red or light red for genes on the leading strand, blue or light blue for genes in on the lagging strand). b) Log2-values greater than 1 or less than -1 are highlighted in red or green respectively. Genes for which less than 8 spots were present were discarded from the analysis and are denoted “NA”. c) A significantly regulated gene (bonferroni corrected level of p<0.05) has the corresponding p-value written in bold. 1.Paley SM, Karp PD (2002) Evaluation of computational metabolic-pathway predictions for *Helicobacter pylori*. Bioinformatics 18: 715–724. 2. Romero PR, Karp PD (2004) Using functional and organizational information to improve genome-wide computational prediction of transcription units on pathway-genome databases. Bioinformatics 20: 709–717.
(0.34 MB PDF)
