# Supplementary material for: The Transcriptome of the Nosocomial Pathogen Enterococcus faecalis V583 Reveals Adaptive Responses to Growth in Blood
Source: PLoS One. 2009 Nov 4;4(11):e7660. doi: 10.1371/journal.pone.0007660 (PMC2766626; doi:10.1371/journal.pone.0007660)
Supplement: Table S1 — Microarray expression data from E. faecalis strain V583 during incubation in blood or 2xYT supplemented with 10% blood (YTB). Gene expression after 30 minutes (blood) or 5, 15, 30 and 60 minutes (YTB) of incubation is relative to the expression during growth in 2xYT for the corresponding time length. a) Genes comprising putative operon structures predicted by http://biocyc.org [1], [2] are marked with one color (red or light red for genes on the leading strand, blue or light blue for genes in on the lagging strand). b) Log2-values greater than 1 or less than -1 are highlighted in red or green respectively. Genes for which less than 8 spots were present were discarded from the analysis and are denoted “NA”. c) A significantly regulated gene (bonferroni corrected level of p<0.05) has the corresponding p-value written in bold. 1.Paley SM, Karp PD (2002) Evaluation of computational metabolic-pathway predictions for Helicobacter pylori. Bioinformatics 18: 715–724. 2. Romero PR, Karp PD (2004) Using functional and organizational information to improve genome-wide computational prediction of transcription units on pathway-genome databases. Bioinformatics 20: 709–717. (0.34 MB PDF) [file pone.0007660.s001.pdf]

Operon[a]	Locus	Gene	Functional category	Subcategory	Putative function	Blood[b]	P-value[c]	YTB_5[b]	P-value[c]	YTB_15[b]	P-value[c]	YTB_30[b]	P-value[c]	YTB_60[b]	P-value[c]
	EF0001	*dnaA*	DNA metabolism	DNA replication, recombination, and repair	chromosomal replication initiator protein DnaA	-0,5	0,18113	-0,1	0,7912967	0,0	0,8917466	-0,2	0,4738632	-0,1	0,7826289
	EF0002	*dnaN*	DNA metabolism	DNA replication, recombination, and repair	DNA polymerase III, beta subunit	-0,9	0,0103331	0,5	0,2741967	-0,2	0,457041	-0,3	0,4191454	0,1	0,743526
	EF0003		Hypothetical proteins	Conserved	conserved hypothetical protein	NA	NA	0,9	0,0587512	-0,6	0,0712811	0,1	0,7914504	0,2	0,5429133
	EF0004	*recF*	DNA metabolism	DNA replication, recombination, and repair	DNA replication and repair protein RecF	0,2	0,5796113	0,0	0,9688925	0,1	0,758433	0,7	0,0356471	0,6	0,0659643
	EF0005	*gyrB*	DNA metabolism	DNA replication, recombination, and repair	DNA gyrase, B subunit	-0,3	0,3661365	-0,4	0,3649903	-0,1	0,8490727	0,5	0,118234	0,2	0,5407155
	EF0006	*gyrA*	DNA metabolism	DNA replication, recombination, and repair	DNA gyrase, A subunit	-0,7	0,0644881	0,1	0,7938833	-0,1	0,798972	0,1	0,7716089	0,1	0,7003976
	EF0007	*rpsF*	Protein synthesis	Ribosomal proteins: synthesis and modification	ribosomal protein S6	-3,3	**0**	-0,5	0,2404138	-0,1	0,6367626	-0,9	0,0050709	-0,1	0,8423755
	EF0008	*ssb-1*	DNA metabolism	DNA replication, recombination, and repair	single-strand binding protein	NA	NA	NA	NA	NA	NA	NA	NA	NA	NA
	EF0009	*rpsR*	Protein synthesis	Ribosomal proteins: synthesis and modification	ribosomal protein S18	-3,8	**0**	-0,8	0,0749774	-0,3	0,3658335	-1,4	0,0000456	0,2	0,4725406
	EF0011		Unknown function	Enzymes of unknown specificity	DHH family protein	0,8	0,0397588	1,3	0,0061906	-0,3	0,3383823	0,1	0,683145	0,1	0,8048415
	EF0012	*rplI*	Protein synthesis	Ribosomal proteins: synthesis and modification	ribosomal protein L9	1,3	0,0003321	0,2	0,6275582	0,0	0,9218334	0,1	0,8509465	0,3	0,3088072
	EF0013	*dnaB*	DNA metabolism	DNA replication, recombination, and repair	replicative DNA helicase	0,0	0,9323191	0,6	0,2191531	-0,2	0,5031099	-0,2	0,4756545	-0,3	0,3212075
	EF0014	*purA*	Purines, pyrimidines, nucleosides, and nucleotides	Purine ribonucleotide biosynthesis	adenylosuccinate synthetase	-1,6	**0,0000122**	-0,4	0,3336186	-0,4	0,154874	0,8	0,0229844	-0,1	0,8739123
	EF0016		Unknown function	General	DegV family protein	1,0	0,0073013	0,2	0,722489	0,5	0,1416004	0,6	0,0792606	0,1	0,6994498
	EF0017		Transport and binding proteins	Unknown substrate	ABC transporter, ATP-binding protein	-1,4	0,0001086	-1,3	0,0068288	-0,3	0,3388547	-0,1	0,7878302	-0,3	0,4494393
	EF0018	*mptR*	Regulatory functions	Protein interactions	sigma-54 interaction domain protein	-0,5	0,1863939	0,3	0,5033889	-0,4	0,2344962	-1,0	0,0044894	-0,2	0,5122456
	EF0019	*mptB*	Transport and binding proteins	Carbohydrates, organic alcohols, and acids	PTS system, mannose-specific IIB component	-4,4	**0**	0,4	0,4061866	-0,7	0,0295245	-2,7	**0**	0,7	0,0332501
	EF0020	*mptA*	Transport and binding proteins	Carbohydrates, organic alcohols, and acids	PTS system, mannose-specific IIAB components	-3,4	**0**	0,4	0,4264271	-0,5	0,0818801	-2,1	**0**	0,6	0,0613793
	EF0021	*mptC*	Transport and binding proteins	Carbohydrates, organic alcohols, and acids	PTS system, mannose-specific IIC component	-1,0	0,0057645	0,6	0,2051869	-0,2	0,6254929	-1,8	**0,0000001**	0,2	0,4676455
	EF0022	*mptD*	Transport and binding proteins	Carbohydrates, organic alcohols, and acids	PTS system, mannose-specific IID component	-3,0	**0**	0,8	0,0813472	-0,3	0,2878063	-1,6	**0,0000021**	0,4	0,2212712
	EF0024		Hypothetical proteins	Conserved	conserved hypothetical protein	-1,1	0,0026791	0,5	0,237604	0,0	0,9232248	-1,1	0,0013599	0,1	0,6871873
	EF0025		Cell envelope	Other	membrane protein, putative	2,1	**0**	0,3	0,5866157	-0,3	0,3890418	-0,1	0,6696509	0,1	0,748527
	EF0026		Hypothetical proteins	Conserved	conserved hypothetical protein	2,2	**0**	2,5	**0**	-0,1	0,7611056	0,4	0,2174419	1,0	0,003485
	EF0027		Regulatory functions	Other	phosphosugar-binding transcriptional regulator, putative	1,0	0,0311627	0,1	0,8532939	-0,2	0,6598574	0,1	0,7928167	-0,1	0,7104039
	EF0028		Transport and binding proteins	Carbohydrates, organic alcohols, and acids	PTS system, IIBC components	1,2	0,0024187	0,4	0,3513049	-0,3	0,4245136	-0,4	0,1999101	-0,3	0,4439824
	EF0029		Unknown function	Enzymes of unknown specificity	aminotransferase, class II	0,5	0,1524074	0,5	0,2440662	-0,1	0,8562256	-0,1	0,7925144	0,0	0,9491712
	EF0030		Transcription	Degradation of RNA	endoribonuclease L-PSP, putative	0,8	0,0769506	0,0	0,9400342	-0,5	0,1298821	-0,4	0,2589293	0,0	0,9453581
	EF0031		Cell envelope	Other	membrane protein, putative	-0,5	0,1776284	-0,9	0,0471121	-0,8	0,0108046	-0,4	0,1883628	0,1	0,809972
	EF0032		Cell envelope	Other	membrane protein, putative	-0,5	0,1474949	0,1	0,8548258	0,2	0,4776173	-0,2	0,5837643	0,0	0,9591665
	EF0033		Hypothetical proteins		hypothetical protein	NA	NA	NA	NA	NA	NA	NA	NA	NA	NA
	EF0034		Hypothetical proteins	Conserved	conserved hypothetical protein	-0,8	0,0604038	-0,7	0,1511358	0,3	0,3623659	0,0	0,9181215	0,2	0,4670263
	EF0035		Hypothetical proteins	Conserved	conserved hypothetical protein	0,5	0,2566901	0,1	0,7470587	0,1	0,6748593	0,3	0,3281707	0,1	0,6820633
	EF0036		Hypothetical proteins	Conserved	conserved hypothetical protein	0,5	0,2260706	-0,4	0,366479	0,2	0,5630193	0,2	0,6243178	0,2	0,6243554
	EF0037	*proA*	Amino acid biosynthesis	Glutamate family	gamma-glutamyl phosphate reductase	-0,8	0,020253	-0,4	0,3973874	0,1	0,6486531	-0,2	0,6209636	0,1	0,8450259
	EF0038	*proB*	Amino acid biosynthesis	Glutamate family	glutamate 5-kinase	-0,5	0,1532974	-0,1	0,8579277	0,2	0,4534364	0,1	0,81175	0,3	0,3898055
	EF0039		Purines, pyrimidines, nucleosides, and nucleotides	2'-Deoxyribonucleotide metabolism	deoxyuridine 5`-triphosphate nucleotidohydrolase, putative	0,1	0,8343488	0,3	0,5609629	0,1	0,6478425	0,0	0,9520957	0,2	0,5145074
	EF0040	*radA*	DNA metabolism	DNA replication, recombination, and repair	DNA repair protein RadA	0,6	0,1208928	0,4	0,4135405	0,3	0,3588068	0,5	0,1245088	0,1	0,7163635
	EF0041		Unknown function	General	PIN domain protein	0,6	0,1145551	0,4	0,4135961	0,4	0,2051551	0,4	0,2803404	0,1	0,6726415
	EF0042	*ispF*	Biosynthesis of cofactors, prosthetic groups, carriers	Other	2C-methyl-D-erythritol 2,4-cyclodiphosphate synthase	0,7	0,0502024	0,6	0,1764782	0,1	0,6958244	0,3	0,4082325	0,1	0,8183071
	EF0043	*gltX*	Protein synthesis	tRNA aminoacylation	glutamyl-tRNA synthetase	0,1	0,8465763	-0,1	0,8170545	0,0	0,9504543	0,1	0,7730147	-0,1	0,8335945
	EF0044	*cysE*	Amino acid biosynthesis	Serine family	serine O-acetyltransferase	NA	NA	NA	NA	NA	NA	NA	NA	NA	NA
	EF0045	*cysS*	Protein synthesis	tRNA aminoacylation	cysteinyl-tRNA synthetase	-0,2	0,5546131	-0,1	0,9066205	-0,2	0,5803985	0,0	0,9161559	-0,3	0,3498891
	EF0046		Hypothetical proteins	Conserved	conserved hypothetical protein	0,0	0,8985763	-0,1	0,8853734	0,0	0,9108915	-0,1	0,8545729	-0,2	0,5442291
	EF0047		Protein synthesis	tRNA and rRNA base modification	RNA methyltransferase, TrmH family	0,1	0,8624641	NA	NA	NA	NA	NA	NA	NA	NA
	EF0048		Hypothetical proteins	Conserved	conserved hypothetical protein	0,1	0,6844177	0,0	0,943901	-0,3	0,2915605	-0,2	0,4579945	-0,2	0,5640878
	EF0049		Transcription	Transcription factors	sigma-70 factor family protien	0,9	0,0412406	0,9	0,1074511	-0,2	0,5196686	0,3	0,4559255	-0,2	0,5640149
	EF0050		Hypothetical proteins	Conserved	conserved hypothetical protein	0,1	0,8012899	0,5	0,3147384	0,5	0,1045689	0,2	0,5007341	0,4	0,1863464
	EF0051	*ispE*	Biosynthesis of cofactors, prosthetic groups, carriers	Other	4-diphosphocytidyl-2C-methyl-D-erythritol kinase	-0,4	0,2308274	-0,4	0,3754621	0,0	0,9915452	0,3	0,3394059	0,2	0,4877077
	EF0052		Hypothetical proteins		hypothetical protein	0,3	0,3875708	0,8	0,0684967	0,1	0,7768019	-0,3	0,4076695	-0,2	0,534174
	EF0053	*dnaQ*	DNA metabolism	DNA replication, recombination, and repair	DNA polymerase III, epsilon subunit	-0,5	0,18444	0,7	0,153086	0,2	0,4456361	-0,4	0,2051595	-0,6	0,0944775
	EF0054		Hypothetical proteins		hypothetical protein	0,5	0,2346738	0,3	0,5048524	0,1	0,7856862	-0,4	0,2829808	-0,5	0,1714618
	EF0055		Cellular processes	Cell adhesion	adhesion lipoprotein	-0,1	0,8047391	-0,2	0,5923174	0,0	0,9709319	-0,1	0,7198593	-0,5	0,1190414
	EF0056		Transport and binding proteins	Unknown substrate	ABC transporter, ATP-binding protein	-2,0	**0,0000001**	-0,4	0,412071	0,5	0,1438776	-0,1	0,6869334	-0,4	0,2744217
	EF0057		Transport and binding proteins	Unknown substrate	ABC transporter, permease protein	-0,9	0,0408618	0,2	0,6750935	0,7	0,0330088	-0,3	0,3423794	-0,3	0,4041274
	EF0058	*purR*	Purines, pyrimidines, nucleosides, and nucleotides	Purine ribonucleotide biosynthesis	pur operon repressor PurR	-1,9	**0,0000002**	-1,7	0,0001795	0,0	0,9631538	0,5	0,1166335	-0,2	0,5395959
	EF0059	*glmU*	Cell envelope	Biosynthesis and degradation of surface poly/liposaccharides	UDP-N-acetylglucosamine pyrophosphorylase	-2,0	**0**	-1,3	0,0043725	-0,3	0,3199398	0,1	0,7197315	-0,5	0,1476287
	EF0062		Purines, pyrimidines, nucleosides, and nucleotides	Nucleotide and nucleoside interconversions	5-nucleotidase family protein	1,4	0,0000835	0,4	0,4267606	0,4	0,262886	0,7	0,0308431	0,4	0,2149374
	EF0063		Transport and binding proteins	Amino acids, peptides and amines	pheromone binding protein, putative	2,5	**0**	0,6	0,2018098	-0,1	0,6965946	-0,3	0,333617	0,7	0,0432621
	EF0064		Hypothetical proteins	Conserved	conserved hypothetical protein	NA	NA	0,5	0,2425262	NA	NA	-0,5	0,1139831	0,0	0,8979151
	EF0065		Unknown function	General	bacterial luciferase family protein	1,8	0,0000423	0,6	0,1913593	-0,4	0,1601806	-0,4	0,2053261	-0,1	0,790043
	EF0066	*ruvA*	DNA metabolism	DNA replication, recombination, and repair	Holliday junction DNA helicase RuvA	-0,4	0,2702981	-0,8	0,0816768	0,0	0,898917	0,1	0,7185738	0,1	0,8442927
	EF0067	*ruvB*	DNA metabolism	DNA replication, recombination, and repair	Holliday junction DNA helicase RuvB	-0,7	0,0456325	-0,6	0,2088666	-0,1	0,6675171	-0,2	0,5155449	0,0	0,9865372
	EF0068		Hypothetical proteins		hypothetical protein	0,7	0,0569857	0,5	0,2993323	-0,8	0,0208634	-0,6	0,0768003	-0,1	0,7153483
	EF0069		Central intermediary metabolism	Amino sugars	N-acetylmannosamine-6-phosphate epimerase, putative	1,8	**0,0000009**	-0,3	0,4999498	0,3	0,3862779	-0,2	0,5243926	-0,4	0,2424282
	EF0071		Cell envelope	Other	lipoprotein, putative	-2,0	**0**	NA	NA	NA	NA	NA	NA	NA	NA
	EF0073		Regulatory functions	DNA interactions	transcriptional regulator, Cro/CI family	1,3	0,0007616	-0,2	0,6316739	-0,4	0,1876787	0,6	0,0905207	0,5	0,1278512
	EF0074		Regulatory functions	DNA interactions	transcriptional regulator, Crp/Fnr family	-1,4	0,0015236	-0,9	0,046631	-0,3	0,3886293	0,8	0,0237746	0,4	0,2205776
	EF0076		Unknown function	Enzymes of unknown specificity	oxidoreductase, short chain dehydrogenase/reductase family	3,2	**0**	0,6	0,1944698	1,0	0,0023012	1,0	0,002355	0,0	0,989743
	EF0077		Hypothetical proteins	Conserved	conserved hypothetical protein	3,7	**0**	0,7	0,1545295	1,1	0,0003823	1,2	0,0005423	0,1	0,7480478
	EF0078		Hypothetical proteins	Conserved	conserved hypothetical protein	3,9	**0**	0,5	0,2795742	1,0	0,0017293	1,3	0,0000881	-0,1	0,8791207
	EF0079	*gls24*	Cellular processes	Adaptations to atypical conditions	gls24 protein	3,9	**0**	0,5	0,271964	1,1	0,0008633	1,2	0,0006727	-0,1	0,8145023
	EF0080	*glsB*	Cellular processes	Adaptations to atypical conditions	gls24 protein	4,2	**0**	0,4	0,3936252	1,0	0,0025982	1,0	0,0023279	0,3	0,2944231
	EF0081		Cell envelope	Other	membrane protein, putative	4,3	**0**	0,6	0,1740478	1,2	0,000124	1,3	0,0001154	0,3	0,4471802
	EF0082		Transport and binding proteins	Unknown substrate	major facilitator family transporter	-1,4	0,0018856	-2,2	**0,0000024**	NA	NA	NA	NA	0,9	0,0054323
	EF0083		Hypothetical proteins		hypothetical protein	NA	NA	NA	NA	NA	NA	NA	NA	0,5	0,1490711
	EF0084		Hypothetical proteins		hypothetical protein	0,8	0,0378999	0,0	0,9848209	-0,5	0,1123186	-0,3	0,4040227	0,4	0,2188517
	EF0085		Hypothetical proteins	Domain	conserved domain protein	-0,2	0,6077142	0,3	0,5528277	-0,7	0,0245376	-0,2	0,4665251	0,2	0,5433109
	EF0086		Hypothetical proteins	Domain	conserved domain protein	NA	NA	1,5	0,0010738	-1,0	0,0132515	-0,6	0,0650023	0,2	0,4734675
	EF0089		Hypothetical proteins	Domain	conserved domain protein	0,0	0,9668822	-0,6	0,2068803	-2,0	**0**	-1,8	**0,0000001**	-0,5	0,1714622
	EF0090		Unknown function	General	diacylglycerol kinase catalytic domain protein	0,7	0,0744725	0,2	0,6668629	-0,1	0,7569688	0,3	0,3652836	-0,2	0,5122138
	EF0091		Hypothetical proteins	Conserved	conserved hypothetical protein	0,7	0,0539535	0,1	0,8299191	-0,2	0,5420575	-0,1	0,6573049	-0,4	0,182242
	EF0092		Hypothetical proteins		hypothetical protein	NA	NA	0,8	0,0790666	NA	NA	-0,7	0,0377525	0,0	0,9980866
	EF0093		Cell envelope	Other	cell wall surface anchor family protein	NA	NA	0,7	0,1145334	NA	NA	-0,8	0,0180468	0,2	0,5816698

Operon[a]	Locus	Gene	Functional category	Subcategory	Putative function	Blood[b]	P-value[c]	YTB_5[b]	P-value[c]	YTB_15[b]	P-value[c]	YTB_30[b]	P-value[c]	YTB_60[b]	P-value[c]
	EF0094		Transport and binding proteins	Carbohydrates, organic alcohols, and acids	formate/nitrite transporter family protein	-2,5	**0**	-1,5	0,0011846	-2,6	**0**	-1,6	**0,0000011**	-0,1	0,7800539
	EF0095		Cell envelope	Other	lipoprotein, putative	2,1	**0**	1,9	0,0000251	-0,4	0,2045676	0,9	0,0051382	1,2	0,0002074
	EF0096		Hypothetical proteins	Conserved	conserved hypothetical protein	0,0	0,9445707	0,7	0,1552184	0,1	0,7799403	0,3	0,3143153	0,8	0,0221052
	EF0097		Cellular processes	Pathogenesis	regulatory protein pfoR, putative	-3,9	**0**	-0,2	0,693112	-1,0	0,0025433	-0,6	0,0716566	-0,5	0,1722729
	EF0098	*sdhB-1*	Energy metabolism	Amino acids and amines	L-serine dehydratase, iron-sulfur-dependent, beta subunit	-4,9	**0**	-0,2	0,7308533	-0,8	0,0107483	-0,5	0,1173174	-0,4	0,2494426
	EF0099	*sdhA-1*	Energy metabolism	Amino acids and amines	L-serine dehydratase, iron-sulfur-dependent, alpha subunit	-3,0	**0**	0,1	0,8930264	-1,1	0,0008529	-0,6	0,0874026	-0,7	0,0374595
	EF0100	*serS-1*	Protein synthesis	tRNA aminoacylation	seryl-tRNA synthetase	-4,4	**0**	0,4	0,3868279	-1,2	0,0002341	-0,4	0,2788254	-0,5	0,1360493
	EF0101		Unknown function	Enzymes of unknown specificity	hydrolase, alpha/beta hydrolase fold family	0,7	0,0421736	0,3	0,4636667	0,0	0,9255544	0,2	0,460359	0,0	0,8975091
	EF0102		Regulatory functions	DNA interactions	transcriptional regulator, ArgR family	0,6	0,1042682	0,9	0,0547341	-0,2	0,4317998	0,3	0,3729961	0,1	0,6663868
	EF0103		Regulatory functions	DNA interactions	transcriptional regulator, ArgR family	0,9	0,0125643	-0,1	0,8880262	-0,6	0,0491752	0,0	0,9901445	-0,1	0,8016512
	EF0104	*arcA*	Energy metabolism	Amino acids and amines	arginine deiminase	1,0	0,0042316	1,2	0,0087727	-0,1	0,719931	-0,1	0,6757508	-0,6	0,0970653
	EF0105	*argF-1*	Energy metabolism	Amino acids and amines	ornithine carbamoyltransferase	1,7	**0,0000051**	1,1	0,018512	0,1	0,7925278	-0,1	0,7882118	-0,8	0,0224899
	EF0106	*arcC-1*	Energy metabolism	Amino acids and amines	carbamate kinase	1,9	**0,0000001**	0,7	0,1135282	-0,2	0,6063663	0,1	0,6556458	-1,1	0,001363
	EF0107		Regulatory functions	DNA interactions	transcriptional regulator, Crp/Fnr family	NA	NA	0,3	0,6543444	NA	NA	-1,0	0,004787	-0,2	0,4910431
	EF0108		Transport and binding proteins	Carbohydrates, organic alcohols, and acids	C4-dicarboxylate transporter, putative	1,9	**0,0000002**	0,7	0,1536947	-0,5	0,0917031	-0,3	0,4345391	-0,8	0,0190469
	EF0109		Unknown function	General	ThiJ/PfpI family protein	0,8	0,021211	0,1	0,7530046	-0,1	0,6517874	0,0	0,9755342	-0,3	0,4124896
	EF0110		Regulatory functions	DNA interactions	transcriptional regulator, ArsR family	NA	NA	1,1	0,0204538	-0,9	0,0035568	-0,3	0,3914479	-0,3	0,4067227
	EF0111		Unknown function	Enzymes of unknown specificity	oxidoreductase, zinc-binding	0,9	0,0185356	0,1	0,8139486	-0,4	0,2679235	0,1	0,7354145	-0,4	0,2113636
	EF0112		Hypothetical proteins	Domain	conserved domain protein	0,3	0,518253	0,7	0,1168851	NA	NA	-0,6	0,1243244	0,1	0,6889892
	EF0113		Hypothetical proteins		hypothetical protein	1,0	0,0077949	0,3	0,4536234	-0,7	0,0553839	0,0	0,9986076	0,1	0,7598922
	EF0114		Cell envelope	Biosynthesis and degradation of surface poly/liposaccharides	glycosyl hydrolase, family 20	0,6	0,0909281	0,7	0,1274873	-0,1	0,824807	0,1	0,7816399	-1,0	0,0022736
	EF0115		Transcription	Degradation of RNA	endoribonuclease L-PSP, putative	0,0	0,961487	0,3	0,5628841	0,4	0,2669011	0,1	0,7283084	-0,4	0,255313
	EF0116		Hypothetical proteins	Conserved	conserved hypothetical protein	0,3	0,4603425	0,3	0,4527182	0,1	0,7019029	0,3	0,3757752	-0,1	0,6863518
	EF0117		Regulatory functions	DNA interactions	transcriptional regulator, GntR family	1,0	0,0079631	0,1	0,8574287	0,1	0,8623582	0,2	0,4728711	-0,3	0,3362494
	EF0118		Energy metabolism	Amino acids and amines	ornithine cyclodeaminase, putative	-0,1	0,7763871	0,6	0,1884134	-0,7	0,0850114	-0,7	0,1099289	0,1	0,8122748
	EF0119		Cellular processes	Toxin production and resistance	phenazine biosynthesis protein PhzF family	0,4	0,4199166	0,3	0,4828118	-0,2	0,6112448	0,0	0,9031256	0,0	0,9524402
	EF0120		Hypothetical proteins	Conserved	conserved hypothetical protein	0,0	0,9732932	0,2	0,6036314	-0,6	0,0790192	0,1	0,8199288	0,1	0,8356515
	EF0121		Hypothetical proteins		hypothetical protein	0,7	0,0720603	0,3	0,543633	-0,9	0,005917	-0,5	0,1693937	-0,1	0,8691981
	EF0122		Hypothetical proteins	Domain	conserved domain protein	-0,4	0,2794249	-0,1	0,7690333	-0,7	0,030467	-0,7	0,0352799	0,2	0,6513143
	EF0123		Hypothetical proteins	Conserved	conserved hypothetical protein	-1,3	0,0008791	-0,1	0,8417066	-0,2	0,4629095	-0,5	0,1199884	0,0	0,9951958
	EF0124		Hypothetical proteins		hypothetical protein	NA	NA	NA	NA	NA	NA	NA	NA	NA	NA
	EF0125		Mobile and extrachromosomal element functions	Transposon functions	IS256, transposase	-1,3	0,0002411	-0,9	0,0427126	-0,2	0,495816	0,1	0,7791948	0,0	0,9839076
	EF0126		Hypothetical proteins	Conserved	conserved hypothetical protein	0,3	0,5054116	-0,2	0,6810957	-0,4	0,2574472	-0,2	0,6456057	0,1	0,8154007
	EF0127		Hypothetical proteins	Conserved	conserved hypothetical protein	1,0	0,0353359	0,5	0,2527816	-0,5	0,2097443	-0,9	0,0085507	0,2	0,6158798
	EF0128		Hypothetical proteins		hypothetical protein	-1,3	0,0039203	0,6	0,1840675	0,1	0,638189	-0,1	0,805263	0,1	0,7163576
	EF0129		Regulatory functions	DNA interactions	transcriptional regulator, Cro/CI family	0,4	0,2327828	0,0	0,9223463	0,1	0,7350431	0,2	0,6265802	0,0	0,9667095
	EF0130		Hypothetical proteins		hypothetical protein	NA	NA	0,0	0,9491431	-0,2	0,5081017	-0,2	0,5550813	-0,1	0,7501661
	EF0131		Hypothetical proteins	Domain	conserved domain protein	NA	NA	0,8	0,1342797	NA	NA	NA	NA	-0,2	0,4698378
	EF0132		Hypothetical proteins		hypothetical protein	NA	NA	0,5	0,3302997	-0,6	0,0812338	-0,1	0,8435855	0,0	0,9875766
	EF0133		Hypothetical proteins		hypothetical protein	0,7	0,1350103	0,2	0,7032186	NA	NA	NA	NA	0,2	0,5733095
	EF0134		Hypothetical proteins		hypothetical protein	1,1	0,0173903	0,9	0,1108411	NA	NA	-0,5	0,1725626	-0,2	0,6152477
	EF0135		Hypothetical proteins	Conserved	conserved hypothetical protein	NA	NA	1,2	0,0332471	NA	NA	NA	NA	0,2	0,5618189
	EF0136		Hypothetical proteins		hypothetical protein	NA	NA	1,0	0,0669919	NA	NA	-0,1	0,8713368	-0,1	0,7387888
	EF0137		Unknown function	General	nucleotidyltransferase domain protein	1,8	0,0000794	0,9	0,0431264	0,1	0,8559571	0,4	0,2834824	-0,2	0,5254316
	EF0138		Hypothetical proteins	Domain	conserved domain protein	NA	NA	0,8	0,1852252	-0,9	0,0168086	NA	NA	-0,1	0,8244485
	EF0139		Cellular processes	Cell division	FtsK/SpoIIIE family protein	0,6	0,2082558	0,9	0,0512536	-0,6	0,0451206	-0,3	0,4363637	-0,1	0,6526999
	EF0140		Hypothetical proteins	Domain	conserved domain protein	NA	NA	1,1	0,0583502	NA	NA	NA	NA	-0,3	0,3454638
	EF0141		Hypothetical proteins		hypothetical protein	NA	NA	0,7	0,2317934	NA	NA	NA	NA	0,1	0,6779512
	EF0142		Hypothetical proteins	Conserved	conserved hypothetical protein	NA	NA	0,7	0,2153323	NA	NA	-0,4	0,2915334	0,2	0,6280319
	EF0143		Regulatory functions	DNA interactions	transcriptional regulator, Cro/CI family	NA	NA	0,8	0,1034575	-0,4	0,2075006	0,0	0,9348043	-0,1	0,8428231
	EF0144		Hypothetical proteins	Domain	conserved domain protein	NA	NA	0,7	0,1904741	NA	NA	NA	NA	0,1	0,6530352
	EF0145		Hypothetical proteins		hypothetical protein	1,5	0,0011224	1,1	0,0442367	-0,2	0,5657947	0,4	0,2897975	-0,7	0,0479468
	EF0146		Cellular processes	Conjugation	surface exclusion protein, putative	1,2	0,0013428	0,6	0,2252662	-0,1	0,8629718	0,1	0,6837394	0,0	0,8850494
	EF0147		Hypothetical proteins		hypothetical protein	1,2	0,0013383	0,1	0,7931349	0,4	0,2041604	0,7	0,0375298	0,0	0,960667
	EF0149	*asa*	Cellular processes	Conjugation	aggregation substance, putative	0,4	0,3881221	0,2	0,5891988	0,2	0,5970475	0,2	0,4934528	-0,1	0,7114064
	EF0150		Cell envelope	Other	membrane protein, putative	-0,2	0,7106262	0,7	0,1317854	-0,2	0,5886497	0,2	0,6481547	-0,1	0,8545344
	EF0151		Hypothetical proteins		hypothetical protein	1,5	0,0010219	0,5	0,3659614	NA	NA	NA	NA	-0,6	0,0733593
	EF0152		Hypothetical proteins		hypothetical protein	NA	NA	-0,2	0,637781	NA	NA	NA	NA	-0,1	0,6762219
	EF0153		Cell envelope	Other	cell wall surface anchor family protein	NA	NA	0,5	0,3965544	-0,1	0,8943441	0,2	0,5831534	0,1	0,6767317
	EF0154		Hypothetical proteins	Conserved	conserved hypothetical protein	NA	NA	0,3	0,4775901	NA	NA	NA	NA	0,3	0,4103584
	EF0155		Hypothetical proteins	Conserved	conserved hypothetical protein	1,3	0,0043607	0,5	0,2356984	0,1	0,8223637	0,5	0,1650243	0,2	0,539514
	EF0156		Hypothetical proteins	Conserved	conserved hypothetical protein	1,2	0,0070081	0,6	0,2094772	-0,3	0,276687	0,3	0,4112337	0,2	0,589192
	EF0157		Hypothetical proteins	Domain	conserved domain protein	3,0	**0**	0,4	0,3381057	0,0	0,9804145	0,0	0,9659931	0,2	0,5635015
	EF0158		Mobile and extrachromosomal element functions	Plasmid functions	conjugal transfer protein, putative	0,3	0,4240754	0,7	0,189927	-0,6	0,0897297	-0,3	0,4051348	0,1	0,6885066
	EF0159		Cell envelope	Other	membrane protein, putative	-0,2	0,6768574	0,5	0,2553553	-0,4	0,281882	-0,1	0,8184883	0,1	0,6555204
	EF0160		Hypothetical proteins	Domain	conserved domain protein	NA	NA	0,7	0,2454076	NA	NA	-0,5	0,1924383	0,1	0,7974097
	EF0161		Hypothetical proteins		hypothetical protein	NA	NA	0,7	0,1391769	NA	NA	NA	NA	-0,4	0,2465382
	EF0162		Hypothetical proteins		hypothetical protein	0,6	0,2181561	0,4	0,4422934	-0,9	0,0066652	-0,2	0,5180578	0,1	0,7671904
	EF0163		Cell envelope	Other	lipoprotein, putative	NA	NA	0,9	0,0457104	NA	NA	NA	NA	-0,6	0,0798882
	EF0164		Cell envelope	Other	lipoprotein, putative	1,4	0,0002633	-0,3	0,5252351	0,0	0,9313633	-0,1	0,6905839	0,1	0,6868676
	EF0165		Hypothetical proteins	Conserved	conserved hypothetical protein	NA	NA	0,2	0,7120676	NA	NA	NA	NA	0,2	0,6406106
	EF0166		DNA metabolism	DNA replication, recombination, and repair	site-specific recombinase, phage integrase family	NA	NA	NA	NA	NA	NA	-0,7	0,0416002	0,1	0,7673418
	EF0167	*guaA*	Purines, pyrimidines, nucleosides, and nucleotides	Purine ribonucleotide biosynthesis	GMP synthase	-1,3	0,0002235	-0,9	0,0549287	0,0	0,87746	0,3	0,3748846	-0,2	0,5847985
	EF0168		Biosynthesis of cofactors, prosthetic groups, carriers	Pantothenate and coenzyme A	pantothenate kinase, putative	-0,9	0,0222181	-0,9	0,0637332	0,0	0,8819737	0,2	0,4754193	0,1	0,6555103
	EF0169		Fatty acid and phospholipid metabolism	Degradation	lipase/acylhydrolase	2,9	**0**	1,0	0,0379173	0,2	0,5061455	0,3	0,335936	0,0	0,9860182
	EF0170		Hypothetical proteins	Conserved	conserved hypothetical protein	NA	NA	1,0	0,0400895	-1,1	0,0004663	-0,7	0,0288439	0,6	0,0795689
	EF0171	*adD*	Purines, pyrimidines, nucleosides, and nucleotides	Salvage of nucleosides and nucleotides	adenosine deaminase	0,9	0,0213108	0,4	0,3340552	0,1	0,8024407	0,0	0,9942396	0,4	0,2777115
	EF0172		Regulatory functions	DNA interactions	sugar-binding transcriptional regulator, LacI family	-1,5	0,0000429	-0,7	0,1405568	0,1	0,7757751	0,3	0,3666197	0,0	0,9087502
	EF0173	*pyn*	Purines, pyrimidines, nucleosides, and nucleotides	Salvage of nucleosides and nucleotides	pyrimidine-nucleoside phosphorylase	-0,4	0,2203525	0,0	0,9595667	0,4	0,2237491	0,4	0,1882613	-0,1	0,7316249
	EF0174	*deoC*	Energy metabolism	Other	deoxyribose-phosphate aldolase	-0,1	0,7835563	0,3	0,5401027	0,3	0,3870816	0,4	0,2666667	-0,4	0,2222161
	EF0175	*cdd*	Purines, pyrimidines, nucleosides, and nucleotides	Salvage of nucleosides and nucleotides	cytidine deaminase	0,9	0,0099197	0,2	0,6564726	0,3	0,3494241	0,3	0,3744145	-0,1	0,7188052
	EF0176		Cell envelope	Other	basic membrane protein family	-0,2	0,5885887	0,0	0,9332843	0,3	0,39825	0,6	0,0533494	-0,2	0,531506
	EF0177		Cell envelope	Other	basic membrane protein family	0,9	0,0114622	0,1	0,854867	0,2	0,6195347	0,9	0,0061558	-0,1	0,7203052

Operon[a]	Locus	Gene	Functional category	Subcategory	Putative function	Blood[b]	P-value[c]	YTB_5[b]	P-value[c]	YTB_15[b]	P-value[c]	YTB_30[b]	P-value[c]	YTB_60[b]	P-value[c]
	EF0178		Transport and binding proteins	Unknown substrate	ABC transporter, ATP-binding protein	-0,2	0,6126947	-0,2	0,7091355	0,0	0,9079328	0,4	0,2614757	-0,3	0,3976223
	EF0179		Transport and binding proteins	Unknown substrate	ABC transporter, permease protein	0,1	0,7270913	0,1	0,8526894	0,0	0,9986881	0,3	0,2984842	-0,4	0,2374577
	EF0180		Transport and binding proteins	Unknown substrate	ABC transporter, permease protein	0,4	0,2787455	0,1	0,8331581	0,1	0,7073144	0,6	0,0756958	-0,4	0,2414753
	EF0183		Hypothetical proteins		hypothetical protein	NA	NA	0,9	0,0624978	-0,9	0,0052535	-0,2	0,5074427	0,1	0,679001
	EF0184		Hypothetical proteins		hypothetical protein	NA	NA	0,2	0,5995959	-0,7	0,0330517	-0,6	0,0669576	0,3	0,4132535
	EF0185	*deoB*	Purines, pyrimidines, nucleosides, and nucleotides	Other	phosphopentomutase	1,1	0,0019052	0,5	0,2769498	0,1	0,706758	1,0	0,0162375	0,0	0,978567
	EF0186	*deoD-1*	Purines, pyrimidines, nucleosides, and nucleotides	Salvage of nucleosides and nucleotides	purine nucleoside phosphorylase	1,2	0,0006946	0,7	0,1364309	0,2	0,5904141	0,9	0,0058204	-0,1	0,8322682
	EF0187	*deoD-2*	Purines, pyrimidines, nucleosides, and nucleotides	Salvage of nucleosides and nucleotides	purine nucleoside phosphorylase	1,3	0,0005969	0,6	0,187903	0,3	0,3774048	0,7	0,038774	0,0	0,9858179
	EF0188	*feuA*	Transport and binding proteins	Cations and iron carrying compounds	iron compound ABC transporter, substrate-binding protein	NA	NA	-0,7	0,1451523	1,4	**0,0000154**	1,7	**0,0000006**	-0,2	0,5179434
	EF0191		Transport and binding proteins	Cations and iron carrying compounds	ferrichrome ABC transporter, ATP-binding protein	NA	NA	0,1	0,7928011	0,1	0,6378654	-0,5	0,2390983	0,6	0,0572472
	EF0192		Transport and binding proteins	Cations and iron carrying compounds	ferrichrome ABC transporter, permease protein	0,8	0,063013	-0,2	0,7368789	0,1	0,7050563	0,7	0,0298913	0,0	0,9658663
	EF0193	*fhuG*	Transport and binding proteins	Cations and iron carrying compounds	ferrichrome ABC transporter, permease protein	NA	NA	0,0	0,9610313	-0,2	0,5725145	-0,3	0,3151832	-0,5	0,1653388
	EF0194		Energy metabolism	Fermentation	NADH-dependent butanol dehydrogenase, putative	1,6	**0,0000096**	0,5	0,2603026	0,4	0,1537835	0,5	0,1356158	-0,1	0,8000308
	EF0195	*gpm*	Energy metabolism	Glycolysis/gluconeogenesis	phosphoglycerate mutase 1	-1,1	0,0017317	-0,3	0,531101	-0,3	0,4097439	0,3	0,3511178	0,4	0,277746
	EF0196		DNA metabolism	DNA replication, recombination, and repair	site-specific recombinase, resolvase family	-1,0	0,0063716	-0,2	0,622899	-0,2	0,5648462	-0,3	0,4497493	-0,2	0,5535574
	EF0197	*rpiA*	Energy metabolism	Pentose phosphate pathway	ribose 5-phosphate isomerase A	0,8	0,0844269	0,6	0,1904621	-0,5	0,1250696	0,0	0,9619191	0,0	0,9969608
	EF0198	*rpsL*	Protein synthesis	Ribosomal proteins: synthesis and modification	ribosomal protein S12	-3,1	**0**	-0,8	0,0927654	-0,2	0,6350205	-1,1	0,0014606	-0,1	0,8619675
	EF0199	*rpsG*	Protein synthesis	Ribosomal proteins: synthesis and modification	ribosomal protein S7	-4,1	**0**	-0,6	0,1663898	-0,1	0,8411301	-1,1	0,0007667	0,1	0,7628685
	EF0200	*fusA*	Protein synthesis	Translation factors	translation elongation factor G	-3,4	**0**	-0,6	0,1922743	-0,2	0,4319224	-1,5	**0,0000137**	0,0	0,9912669
	EF0201	*tuf*	Protein synthesis	Translation factors	translation elongation factor Tu	-1,7	**0,0000028**	-0,3	0,5523037	-0,2	0,5375152	-0,7	0,0252327	0,1	0,8306717
	EF0202		Biosynthesis of cofactors, prosthetic groups, carriers	Thiamine	phosphomethylpyrimidine kinase, putative	0,5	0,21129	0,1	0,8589669	0,3	0,2878883	0,4	0,2413994	-0,2	0,5785558
	EF0203	*cfa*	Fatty acid and phospholipid metabolism	Biosynthesis	cyclopropane-fatty-acyl-phospholipid synthase	0,1	0,8619024	0,2	0,6187208	-0,1	0,7425226	0,3	0,3563924	0,4	0,2486263
	EF0205	*rpsJ*	Protein synthesis	Ribosomal proteins: synthesis and modification	ribosomal protein S10	-3,5	**0**	-1,0	0,0329888	-0,3	0,3650158	-0,9	0,0066427	0,0	0,9444274
	EF0206	*rplC*	Protein synthesis	Ribosomal proteins: synthesis and modification	ribosomal protein L3	-2,6	**0**	-0,7	0,1093759	-0,1	0,8123288	-1,0	0,0027353	0,1	0,7438108
	EF0207	*rplD*	Protein synthesis	Ribosomal proteins: synthesis and modification	ribosomal protein L4	-4,1	**0**	-0,8	0,0950641	-0,2	0,4860633	-1,0	0,0021499	0,0	0,8926669
	EF0208	*rplW*	Protein synthesis	Ribosomal proteins: synthesis and modification	ribosomal protein L23	-4,0	**0**	-0,8	0,0794502	-0,2	0,5461595	-1,3	0,0001178	0,1	0,7999434
	EF0209	*rplB*	Protein synthesis	Ribosomal proteins: synthesis and modification	ribosomal protein L2	-4,2	**0**	-0,9	0,0479872	-0,3	0,3455359	-1,2	0,0004239	0,0	0,9178349
	EF0210	*rpsS*	Protein synthesis	Ribosomal proteins: synthesis and modification	ribosomal protein S19	-1,7	**0,0000042**	-0,6	0,2216473	-0,4	0,2031352	-1,2	0,0002494	0,1	0,8511855
	EF0211	*rplV*	Protein synthesis	Ribosomal proteins: synthesis and modification	ribosomal protein L22	-3,0	**0**	-0,8	0,1019336	-0,1	0,6530221	-1,4	0,0000386	0,1	0,6991534
	EF0212	*rpsC*	Protein synthesis	Ribosomal proteins: synthesis and modification	ribosomal protein S3	-3,9	**0**	-0,7	0,1146483	-0,1	0,6505577	-1,3	0,0001092	0,2	0,6472572
	EF0213	*rplP*	Protein synthesis	Ribosomal proteins: synthesis and modification	ribosomal protein L16	-2,5	**0**	-0,6	0,2108536	0,0	0,9324032	-1,0	0,0028265	0,4	0,1791423
	EF0214	*rpmC*	Protein synthesis	Ribosomal proteins: synthesis and modification	ribosomal protein L29	-2,4	**0**	-0,4	0,385277	-0,3	0,4143897	-1,0	0,0025386	0,5	0,1032254
	EF0215	*rpsQ*	Protein synthesis	Ribosomal proteins: synthesis and modification	ribosomal protein S17	-3,5	**0**	-0,8	0,0733437	-0,1	0,6693849	-1,3	0,0001247	0,4	0,1805462
	EF0216	*rplN*	Protein synthesis	Ribosomal proteins: synthesis and modification	ribosomal protein L14	-4,8	**0**	-0,6	0,2031904	-0,2	0,4803138	-1,2	0,0002048	0,2	0,4928386
	EF0217	*rplX*	Protein synthesis	Ribosomal proteins: synthesis and modification	ribosomal protein L24	-2,0	**0,0000001**	-0,4	0,3463476	-0,1	0,6505513	-1,5	**0,000005**	0,3	0,3397961
	EF0218	*rplE*	Protein synthesis	Ribosomal proteins: synthesis and modification	ribosomal protein L5	-3,5	**0**	-0,5	0,2916714	0,0	0,9827354	-1,3	0,0001078	0,2	0,509584
	EF0219	*rpsN-1*	Protein synthesis	Ribosomal proteins: synthesis and modification	ribosomal protein S14	-3,2	**0**	-0,5	0,2670597	0,1	0,655484	-1,4	0,0000194	0,2	0,534627
	EF0220	*rpsH*	Protein synthesis	Ribosomal proteins: synthesis and modification	ribosomal protein S8	-4,1	**0**	-0,5	0,2742147	0,0	0,8895134	-1,2	0,000213	0,2	0,6291789
	EF0221	*rplF*	Protein synthesis	Ribosomal proteins: synthesis and modification	ribosomal protein L6	-2,4	**0**	-0,5	0,2866146	0,0	0,9589997	-1,3	0,0000837	0,4	0,2899008
	EF0223	*rplR*	Protein synthesis	Ribosomal proteins: synthesis and modification	ribosomal protein L18	-3,0	**0**	-0,5	0,2742403	-0,1	0,8342793	-1,2	0,0002212	0,3	0,3194087
	EF0224	*rpsE*	Protein synthesis	Ribosomal proteins: synthesis and modification	ribosomal protein S5	-3,5	**0**	-0,5	0,2375497	-0,1	0,7379037	-1,4	0,0000398	0,3	0,3357094
	EF0225	*rpmD*	Protein synthesis	Ribosomal proteins: synthesis and modification	ribosomal protein L30	-3,2	**0**	-0,6	0,1965815	-0,1	0,6885328	-1,4	0,000049	0,3	0,3162882
	EF0226	*rplO*	Protein synthesis	Ribosomal proteins: synthesis and modification	ribosomal protein L15	-3,4	**0**	-0,6	0,2149783	0,0	0,9738512	-1,3	0,0000911	0,3	0,3449194
	EF0227	*secY*	Protein fate	Protein and peptide secretion and trafficking	preprotein translocase, SecY subunit	-4,3	**0**	-0,4	0,4053247	-0,1	0,6759725	-1,3	0,0001343	0,2	0,5571034
	EF0228	*adk*	Purines, pyrimidines, nucleosides, and nucleotides	Nucleotide and nucleoside interconversions	adenylate kinase	-4,1	**0**	-0,8	0,092101	-0,2	0,5615267	-1,4	0,0000329	0,2	0,5356326
	EF0229	*infA*	Protein synthesis	Translation factors	translation initiation factor IF-1	-3,5	**0**	-0,9	0,0609389	-0,1	0,7326502	-1,0	0,0041719	0,2	0,6038876
	EF0230	*rpmJ*	Protein synthesis	Ribosomal proteins: synthesis and modification	ribosomal protein L36	-1,9	**0,0000003**	-0,7	0,1304699	-0,1	0,8664177	-0,8	0,0120886	0,1	0,6811566
	EF0231	*rpsM*	Protein synthesis	Ribosomal proteins: synthesis and modification	ribosomal protein S13	NA	NA	NA	NA	NA	NA	NA	NA	NA	NA
	EF0232	*rpsK*	Protein synthesis	Ribosomal proteins: synthesis and modification	ribosomal protein S11	NA	NA	0,3	0,5663599	NA	NA	-0,8	0,027193	-0,1	0,6732813
	EF0233	*rpoA*	Transcription	DNA-dependent RNA polymerase	DNA-directed RNA polymerase, alpha subunit	-3,5	**0**	-1,2	0,0109828	-0,2	0,5231231	-0,7	0,026272	0,0	0,9125869
	EF0234	*rplQ*	Protein synthesis	Ribosomal proteins: synthesis and modification	ribosomal protein L17	-3,4	**0**	-1,4	0,0020494	-0,4	0,2385661	-0,6	0,0924273	0,1	0,8808088
	EF0235		Cell envelope	Other	membrane protein, putative	-0,4	0,2389183	-0,8	0,0894292	-0,3	0,3030355	-0,4	0,1858069	-0,1	0,8369772
	EF0236		Protein fate	Degradation of proteins, peptides, and glycopeptides	peptidase, M20/M25/M40 family	-0,2	0,6052366	0,1	0,8985713	-0,3	0,3836945	-0,6	0,0718737	0,0	0,9714046
	EF0237		Transport and binding proteins	Unknown substrate	ABC transporter, ATP-binding protein	-1,3	0,0006249	-1,0	0,0283816	0,3	0,3909945	-0,2	0,5888923	0,0	0,9030398
	EF0238		Transport and binding proteins	Unknown substrate	ABC transporter, ATP-binding protein	-1,9	**0,0000003**	-0,9	0,0484193	0,0	0,9083735	0,0	0,954271	-0,3	0,3398908
	EF0239		Transport and binding proteins	Cations and iron carrying compounds	cobalt transport family protein	0,0	0,9225068	-1,2	0,0100671	-0,2	0,6278072	-0,2	0,6521666	-0,3	0,3826115
	EF0240	*truA-1*	Protein synthesis	tRNA and rRNA base modification	tRNA pseudouridine synthase A	-1,0	0,0091741	-0,8	0,0877418	-0,3	0,2809934	-0,2	0,5853738	-0,2	0,5024077
	EF0241		Hypothetical proteins	Conserved	conserved hypothetical protein	1,9	**0,000001**	0,1	0,845808	0,7	0,0367636	0,9	0,0357682	-0,6	0,0829307
	EF0243	*brnQ*	Transport and binding proteins	Amino acids, peptides and amines	branched-chain amino acid transport system II carrier protein	-0,2	0,5588831	-0,4	0,3909992	0,1	0,7893318	-0,2	0,4748049	0,1	0,676769
	EF0244		Unknown function	Enzymes of unknown specificity	acetyltransferase, GNAT family	NA	NA	0,0	0,9340068	-0,3	0,4495636	-0,3	0,3554992	0,1	0,6995959
	EF0245		Unknown function	Enzymes of unknown specificity	decarboxylase family protein	-0,1	0,8701403	0,0	0,9272589	0,4	0,245442	0,5	0,140468	-0,2	0,5331228
	EF0246		Transport and binding proteins	Amino acids, peptides and amines	amino acid ABC transporter, ATP-binding protein	-1,3	0,0003154	-0,2	0,6592088	-0,7	0,028288	-0,8	0,0234234	-0,5	0,1550221
	EF0247		Transport and binding proteins	Amino acids, peptides and amines	amino acid ABC transporter, amino acid-binding/permease protein	0,1	0,7817531	-0,6	0,1941835	-0,4	0,1702878	-0,2	0,5405698	-0,7	0,030194
	EF0248		Hypothetical proteins		hypothetical protein	-1,3	0,0004137	-0,4	0,4319912	-1,3	0,0000581	-0,7	0,0393059	0,1	0,7437243
	EF0249		Regulatory functions	Other	transcriptional regulator, Sir2 family	-0,6	0,0825543	-0,4	0,4336881	0,2	0,5460774	0,3	0,4413464	-0,1	0,8656237
	EF0250		Energy metabolism	Other	maltose O-acetyltransferase, putative	-0,5	0,1455047	-0,2	0,6300308	0,2	0,4462469	0,4	0,263703	0,0	0,9978903
	EF0251		Regulatory functions	Other	transcriptional regulator, putative	0,0	0,9314177	-0,2	0,7319623	0,0	0,9602002	0,2	0,5640627	0,0	0,9455827
	EF0252		Cell envelope	Biosynthesis/degradation of murein sacculus/peptidoglycan	N-acetylmuramoyl-L-alanine amidase, family 4	0,2	0,5798623	-0,3	0,5597077	0,2	0,6359425	0,3	0,4496115	-0,1	0,6976341
	EF0253		Energy metabolism	Fermentation	aldehyde dehydrogenase	2,8	**0**	0,5	0,2403123	-0,7	0,0227841	-0,3	0,4286364	-0,7	0,0361876
	EF0255	*ldh-1*	Energy metabolism	Glycolysis/gluconeogenesis	L-lactate dehydrogenase	0,1	0,7732335	-0,7	0,1448608	-0,6	0,0453712	1,7	**0,0000004**	0,3	0,3860885
	EF0256	*pth*	Protein synthesis	Other	peptidyl-tRNA hydrolase	NA	NA	-0,1	0,9003305	0,0	0,9467732	-1,1	0,0006413	0,0	0,9010675
	EF0257	*mfd*	DNA metabolism	DNA replication, recombination, and repair	transcription-repair coupling factor	-0,1	0,7092592	-0,2	0,6884908	-0,2	0,6049738	-0,3	0,323063	0,0	0,9403279
	EF0258		Cell envelope	Biosynthesis and degradation of surface poly/liposaccharides	polysaccharide biosynthesis family protein	0,0	0,9712508	0,0	0,9735873	-0,2	0,6206996	-0,4	0,187793	0,0	0,9776646
	EF0259		Unknown function	General	S4 RNA-binding domain protein	-0,5	0,3101821	-0,1	0,7587268	-0,3	0,3745563	-0,5	0,1788054	-0,3	0,4072199
	EF0260		Hypothetical proteins		hypothetical protein	NA	NA	NA	NA	NA	NA	NA	NA	NA	NA
	EF0261		Hypothetical proteins	Conserved	conserved hypothetical protein	0,0	0,9595612	-0,5	0,2730345	0,4	0,1684018	0,5	0,1281801	0,3	0,3872204
	EF0262		Unknown function	General	S1 RNA-binding domain protein	-1,0	0,0064413	-0,7	0,1351074	0,2	0,5877709	0,2	0,5097546	0,1	0,6914687
	EF0263	*tilS*	Hypothetical proteins	Domain	tRNA(Ile)-lysidine synthetase	-0,6	0,1233854	-0,7	0,1233868	-0,1	0,6935415	0,0	0,9194296	-0,1	0,6616277
	EF0264	*hpt*	Purines, pyrimidines, nucleosides, and nucleotides	Salvage of nucleosides and nucleotides	hypoxanthine-guanine phosphoribosyltransferase	-1,3	0,0002607	-0,7	0,1393869	-0,2	0,5234487	-0,2	0,5762559	-0,1	0,7686479
	EF0265	*ftsH*	Cellular processes	Cell division	cell division protein FtsH	0,8	0,0318712	0,0	0,9249527	-0,2	0,6344546	-0,2	0,4727594	-0,2	0,5769289
	EF0266		Protein fate	Protein folding and stabilization	chaperonin, 33 kDa	-0,1	0,7063519	1,0	0,0383475	-0,7	0,0371846	-0,6	0,0846435	1,0	0,0040719
	EF0267		Unknown function	General	zinc-binding TIM-barrel protein, nifR3 family, putative	-0,3	0,3724901	-0,7	0,1259715	0,4	0,1977115	0,3	0,3081658	-0,1	0,7324644
	EF0268	*lysS*	Protein synthesis	tRNA aminoacylation	lysyl-tRNA synthetase	-1,1	0,0028064	-0,3	0,5842453	0,3	0,4290064	0,1	0,8543111	0,0	0,9444272

Operon[a]	Locus	Gene	Functional category	Subcategory	Putative function	Blood[b]	P-value[c]	YTB_5[b]	P-value[c]	YTB_15[b]	P-value[c]	YTB_30[b]	P-value[c]	YTB_60[b]	P-value[c]
	EF0270		Signal transduction	PTS	PTS system, beta-glucoside-specific IIABC component	0,4	0,3418068	0,3	0,5727584	0,3	0,3217032	-0,1	0,7552561	-0,5	0,1544315
	EF0271		Energy metabolism	Biosynthesis and degradation of polysaccharides	glycosyl hydrolase, family 1	1,1	0,0137452	0,9	0,0470115	0,1	0,7294063	-0,2	0,6435085	-0,3	0,3329478
	EF0272		Energy metabolism	Biosynthesis and degradation of polysaccharides	glycosyl hydrolase, family 1	NA	NA	0,6	0,2172368	-0,1	0,7388253	-0,3	0,5316115	-0,9	0,0081244
	EF0273		Energy metabolism	Glycolysis/gluconeogenesis	phosphoglycerate mutase family protein	NA	NA	0,8	0,0960963	-1,1	0,0008023	-0,3	0,3814158	0,1	0,7421555
	EF0274		Hypothetical proteins	Conserved	conserved hypothetical protein	0,6	0,2004993	0,2	0,6154037	0,0	0,9832696	-0,1	0,8472793	0,4	0,1813584
	EF0275		Regulatory functions	DNA interactions	ada regulatory protein, putative	0,3	0,5019567	0,6	0,183778	0,1	0,7244742	0,1	0,7410445	0,2	0,4765173
	EF0277	*ogt*	DNA metabolism	DNA replication, recombination, and repair	methylated-DNA--protein-cysteine S-methyltransferase	-0,1	0,7450983	0,2	0,625756	0,3	0,3108704	-0,1	0,8262366	-0,3	0,3822428
	EF0278	*tag-1*	DNA metabolism	DNA replication, recombination, and repair	DNA-3-methyladenine glycosylase I	0,4	0,3478864	0,4	0,3546607	0,1	0,7111801	0,2	0,5761105	-0,1	0,8207817
	EF0279		Unknown function	General	HD domain protein	-0,2	0,6263135	0,2	0,6796771	-0,6	0,0487688	-0,5	0,1362128	0,2	0,5616833
	EF0280		Transport and binding proteins	Cations and iron carrying compounds	cation efflux family protein	0,3	0,392744	-0,4	0,388725	0,2	0,4578513	0,2	0,6425422	-0,1	0,7179941
	EF0281		Hypothetical proteins		hypothetical protein	0,2	0,5350924	-0,4	0,4198753	0,5	0,14168	1,0	0,0194594	0,6	0,0737794
	EF0282	*fabI*	Fatty acid and phospholipid metabolism	Biosynthesis	enoyl-(acyl-carrier-protein) reductase	2,1	**0**	4,3	**0**	3,1	**0**	3,3	**0**	1,1	0,0008824
	EF0283	*fabF-1*	Fatty acid and phospholipid metabolism	Biosynthesis	3-oxoacyl-(acyl-carrier-protein) synthase II	2,7	**0**	4,1	**0**	3,9	**0**	3,9	**0**	1,4	**0,0000158**
	EF0284	*fabZ-1*	Fatty acid and phospholipid metabolism	Biosynthesis	(3R)-hydroxymyristoyl-(acyl-carrier-protein) dehydratase	1,6	0,0004833	3,6	**0**	3,8	**0**	3,1	**0**	1,7	**0,0000002**
	EF0285	*pyrD-1*	Purines, pyrimidines, nucleosides, and nucleotides	Pyrimidine ribonucleotide biosynthesis	dihydroorotate dehydrogenase	0,9	0,0157408	1,0	0,0268575	1,0	0,0023873	0,8	0,0163508	0,8	0,0125549
	EF0286		Transport and binding proteins	Other	fibronectin-binding protein, putative	NA	NA	1,1	0,0135613	0,3	0,3434797	0,3	0,3313304	0,3	0,3432388
	EF0287	*efp*	Protein synthesis	Translation factors	translation elongation factor P	-0,8	0,0204216	-0,6	0,1820686	-0,3	0,3277195	-0,2	0,5747594	-0,4	0,2600285
	EF0288		Hypothetical proteins		hypothetical protein	-0,1	0,8018975	0,4	0,3477194	0,3	0,3447005	0,1	0,8970313	-0,5	0,1064344
	EF0289		Amino acid biosynthesis	Serine family	cysteine synthase B, putative	0,5	0,2213143	0,3	0,5326473	-0,5	0,1494135	-1,4	0,0000279	-1,1	0,0009678
	EF0290	*metC*	Amino acid biosynthesis	Aspartate family	cystathionine beta-lyase	-1,8	**0,000001**	-0,5	0,2354031	0,1	0,7185433	-0,5	0,1187855	-0,5	0,1386827
	EF0291		Energy metabolism	Biosynthesis and degradation of polysaccharides	glycosyl hydrolase, family 1	3,0	**0**	1,1	0,0182468	1,0	0,0009964	1,4	0,0000526	0,1	0,7557468
	EF0292		Signal transduction	PTS	PTS system, IIC component	2,4	**0**	1,4	0,0111095	1,2	0,0001717	1,2	0,0006059	0,5	0,1152486
	EF0293		Regulatory functions	Other	phosphosugar-binding transcriptional regulator, putative	0,2	0,6907857	-0,2	0,6345617	0,9	0,0054581	1,1	0,0010254	-0,4	0,184706
	EF0294		Hypothetical proteins	Conserved	conserved hypothetical protein	1,2	0,0015031	0,1	0,8998477	0,1	0,7886321	0,7	0,0483907	-0,2	0,5700951
	EF0295		Transport and binding proteins	Cations and iron carrying compounds	V-type ATPase, subunit J	0,5	0,1513802	0,2	0,6591581	-0,2	0,5713043	0,4	0,2530413	-0,3	0,3669731
	EF0296	*napA*	Transport and binding proteins	Cations and iron carrying compounds	Na+/H+ antiporter	0,4	0,2448473	0,2	0,7310736	0,0	0,9962535	0,3	0,4296175	-0,1	0,7711393
	EF0297	*copY*	Regulatory functions	DNA interactions	transcriptional repressor CopY	0,2	0,734426	0,0	0,971631	0,2	0,5980847	0,4	0,2452417	-0,7	0,0317512
	EF0298		Cellular processes	Detoxification	copper-translocating P-type ATPase	-0,2	0,6541321	-0,5	0,3094254	0,4	0,2534642	0,2	0,4901807	-0,6	0,0953665
	EF0299	*copZ*	Transport and binding proteins	Cations and iron carrying compounds	copper transport protein CopZ	0,7	0,1361496	-0,1	0,8920901	0,2	0,5981029	-0,3	0,3227379	-0,1	0,7389705
	EF0300		Cell envelope	Other	membrane protein, putative	0,7	0,06085	0,0	0,9552403	0,1	0,781247	0,2	0,6126036	-0,1	0,7433012
	EF0301		Regulatory functions	DNA interactions	transcriptional regulator, GntR family	0,0	0,9590402	-0,5	0,2981248	-0,1	0,7814752	0,6	0,0770817	-0,5	0,0972631
	EF0302	*pepC*	Protein fate	Degradation of proteins, peptides, and glycopeptides	aminopeptidase C	0,7	0,043788	0,0	0,9564312	0,0	0,900145	0,0	0,9716358	-0,1	0,6987118
	EF0303		Mobile and extrachromosomal element functions	Prophage functions	phage integrase	-0,1	0,7338031	-0,3	0,5737096	0,2	0,4555404	0,2	0,6397716	-0,2	0,4518225
	EF0304		Cell envelope	Other	lipoprotein, putative	0,4	0,2277899	-0,2	0,669049	0,2	0,4467292	0,3	0,4225906	0,0	0,8822854
	EF0305		Hypothetical proteins	Domain	conserved domain protein	0,2	0,6736497	-0,5	0,3070089	0,3	0,3580586	0,6	0,0841791	-0,4	0,287813
	EF0306		Mobile and extrachromosomal element functions	Prophage functions	transcriptional regulator, Cro/CI family	0,3	0,4323941	-0,6	0,1742903	0,2	0,4934146	0,8	0,0115216	-0,5	0,1271815
	EF0307		Mobile and extrachromosomal element functions	Prophage functions	transcriptional regulator, Cro/CI family	1,5	0,0000244	-0,1	0,9058062	0,3	0,3285148	0,3	0,3535821	0,0	0,9055351
	EF0308		Hypothetical proteins	Conserved	conserved hypothetical protein	2,0	**0**	-0,3	0,5450053	0,2	0,6180921	0,3	0,2998918	-0,7	0,0483902
	EF0309		Mobile and extrachromosomal element functions	Prophage functions	excisionase, putative	2,1	**0**	-0,3	0,4518686	0,2	0,5116263	0,0	0,9096368	-0,3	0,3974052
	EF0310		Hypothetical proteins		hypothetical protein	NA	NA	NA	NA	NA	NA	NA	NA	NA	NA
	EF0311		Hypothetical proteins		hypothetical protein	1,3	0,0003875	-0,3	0,530782	-0,1	0,7363361	-0,2	0,5892672	-0,1	0,7132974
	EF0312		Unknown function	General	aspartate 1-decarboxylase domain protein	1,5	0,0000979	0,1	0,762112	-0,3	0,3985812	0,1	0,7187673	-0,4	0,2758928
	EF0313		Hypothetical proteins		hypothetical protein	1,7	0,0002078	0,8	0,0900337	NA	NA	NA	NA	-0,8	0,0110267
	EF0314		Hypothetical proteins		hypothetical protein	1,9	**0,0000002**	0,6	0,2061788	0,2	0,5361747	0,6	0,0823013	-1,0	0,0021945
	EF0315		Hypothetical proteins	Conserved	conserved hypothetical protein	1,9	**0,0000004**	0,4	0,4500169	-0,2	0,6239322	0,0	0,9823659	-0,6	0,0787545
	EF0316		Hypothetical proteins		hypothetical protein	NA	NA	0,3	0,5391516	-0,1	0,8553975	NA	NA	-0,1	0,8009501
	EF0317		Mobile and extrachromosomal element functions	Prophage functions	transcriptional regulator, Cro/CI family	1,4	0,0001223	0,5	0,4206151	0,2	0,6243591	0,3	0,3448819	-1,2	0,0005636
	EF0318		Hypothetical proteins	Conserved	conserved hypothetical protein	2,1	**0**	0,2	0,6672806	0,4	0,2586433	-0,1	0,8643983	-0,7	0,0345057
	EF0319		Hypothetical proteins	Conserved	conserved hypothetical protein	2,4	**0**	-0,2	0,6750161	0,0	0,934285	-0,7	0,035206	-1,3	0,0000791
	EF0320		Hypothetical proteins		hypothetical protein	1,1	0,0030451	0,2	0,6520276	-0,5	0,1160065	-0,6	0,0566306	-0,8	0,0127433
	EF0321		Hypothetical proteins		hypothetical protein	1,6	**0,0000065**	0,1	0,9104134	-0,1	0,6939614	-0,1	0,7243083	-0,3	0,3912156
	EF0322		Hypothetical proteins	Conserved	conserved hypothetical protein	2,3	**0**	-0,5	0,254807	0,3	0,3351948	-0,2	0,5997486	-0,1	0,8397871
	EF0323		Hypothetical proteins		hypothetical protein	-0,9	0,0245614	-0,9	0,0530809	0,2	0,5304123	0,1	0,8224331	-0,7	0,0266595
	EF0324		Hypothetical proteins		hypothetical protein	1,8	**0,0000009**	-0,5	0,2425013	0,1	0,6370337	-0,1	0,7993743	-1,0	0,003827
	EF0325		DNA metabolism	DNA replication, recombination, and repair	DNA polymerase, putative	1,7	**0,0000023**	-0,3	0,5656532	-0,3	0,2972562	-0,9	0,0072189	-0,6	0,0509197
	EF0326		Hypothetical proteins	Conserved	conserved hypothetical protein	1,4	0,0001704	1,2	0,0382888	NA	NA	0,4	0,3950106	-0,6	0,077828
	EF0327		Hypothetical proteins		hypothetical protein	0,6	0,1045093	0,7	0,2159206	0,8	0,0384942	0,0	0,9633774	-0,8	0,0156835
	EF0328		Hypothetical proteins	Conserved	conserved hypothetical protein	1,2	0,0011631	0,0	0,9985581	0,2	0,618136	-0,4	0,2894189	-0,3	0,3539294
	EF0329		Hypothetical proteins	Conserved	conserved hypothetical protein	0,9	0,00973	0,5	0,2969692	0,4	0,1631349	-0,2	0,5493177	-0,5	0,1303766
	EF0330		Unknown function	General	SNF2 domain protein	1,3	0,0005701	-0,2	0,7708009	0,4	0,2235583	0,0	0,9374004	-0,7	0,0402264
	EF0331		Hypothetical proteins	Conserved	conserved hypothetical protein	1,4	0,0003461	0,4	0,4322472	0,0	0,8777139	-0,5	0,1738031	-0,3	0,3848124
	EF0332		Hypothetical proteins	Conserved	conserved hypothetical protein	-1,3	0,0003778	-0,4	0,3581683	0,1	0,8668489	-0,7	0,0319023	-1,7	**0,0000004**
	EF0333		Hypothetical proteins	Conserved	conserved hypothetical protein TIGR01630	-1,6	0,000018	-0,8	0,0762684	0,0	0,9604313	0,4	0,2315215	-1,4	0,0000426
	EF0334		Mobile and extrachromosomal element functions	Prophage functions	portal protein	-0,7	0,0857988	-0,7	0,1250309	0,4	0,2631017	-0,4	0,2236894	-0,4	0,280164
	EF0335		Mobile and extrachromosomal element functions	Prophage functions	minor head protein	NA	NA	NA	NA	NA	NA	NA	NA	NA	NA
	EF0336		Hypothetical proteins		hypothetical protein	0,6	0,0999673	0,2	0,7153332	0,4	0,2354749	0,3	0,3681207	-0,4	0,2761561
	EF0337		Hypothetical proteins		hypothetical protein	1,3	0,003394	-0,1	0,7543548	-0,7	0,035765	-0,6	0,0845167	-1,0	0,002816
	EF0338		Mobile and extrachromosomal element functions	Prophage functions	scaffold protein	NA	NA	-0,2	0,6246725	NA	NA	NA	NA	-0,7	0,0466001
	EF0339		Mobile and extrachromosomal element functions	Prophage functions	major capsid protein, putative	-1,4	0,0001326	0,0	0,9512235	-0,2	0,4507171	-0,6	0,0560561	-1,5	**0,0000043**
	EF0340		Hypothetical proteins		hypothetical protein	NA	NA	NA	NA	NA	NA	NA	NA	NA	NA
	EF0341		Hypothetical proteins		hypothetical protein	-1,7	**0,000006**	-0,1	0,7562764	-0,3	0,3979082	-0,6	0,0741814	-1,5	**0,0000035**
	EF0342		Hypothetical proteins		hypothetical protein	-1,4	0,000088	0,0	0,9609002	-0,4	0,2240721	-0,6	0,096611	-1,5	**0,0000061**
	EF0343		Hypothetical proteins	Conserved	conserved hypothetical protein TIGR01725	0,0	0,9027911	0,0	0,9730992	-0,7	0,0206289	-1,0	0,0031762	-1,8	**0,0000001**
	EF0344		Hypothetical proteins		hypothetical protein	-1,3	0,0004132	-0,3	0,5026833	-0,3	0,3416923	-0,4	0,2023484	-0,9	0,0077856
	EF0345		Hypothetical proteins	Domain	conserved domain protein	-1,3	0,0004459	-0,6	0,2232301	-0,2	0,520746	-0,1	0,6810021	-1,2	0,0002922
	EF0346		Hypothetical proteins		hypothetical protein	NA	NA	-0,8	0,0996118	0,3	0,3939171	-0,8	0,022047	-0,3	0,3411786
	EF0347		Unknown function	General	peptide methionine sulfoxide reductase domain protein	-0,9	0,0128577	-0,5	0,2488581	-0,1	0,8012274	-0,3	0,3296695	-1,3	0,0000991
	EF0348		Mobile and extrachromosomal element functions	Prophage functions	tail protein	-1,1	0,0030908	-0,3	0,5219122	-0,4	0,2466311	-0,5	0,1745997	-1,6	**0,0000025**
	EF0349		Mobile and extrachromosomal element functions	Prophage functions	tail protein, putative	-0,5	0,2163966	-0,9	0,0528458	-0,2	0,5374877	-0,9	0,0109506	-0,6	0,0593278
	EF0350		Hypothetical proteins	Conserved	conserved hypothetical protein	-0,8	0,0365628	-0,6	0,1912235	-0,1	0,7075084	-0,2	0,4828107	-0,6	0,0730334
	EF0351		Mobile and extrachromosomal element functions	Prophage functions	structural protein, putative	-1,7	**0,0000037**	NA	NA	-0,2	0,5063382	-0,2	0,4599327	-0,9	0,0075211
	EF0352		Hypothetical proteins		hypothetical protein	-2,0	**0**	-0,5	0,2865803	0,0	0,9092624	-0,5	0,1521479	-1,0	0,0019796
	EF0353		Mobile and extrachromosomal element functions	Prophage functions	holin, putative	-1,0	0,0047576	-0,4	0,403555	0,5	0,1231173	0,3	0,3734995	-0,7	0,0413985

Operon[a]	Locus	Gene	Functional category	Subcategory	Putative function	Blood[b]	P-value[c]	YTB_5[b]	P-value[c]	YTB_15[b]	P-value[c]	YTB_30[b]	P-value[c]	YTB_60[b]	P-value[c]
	EF0354		Mobile and extrachromosomal element functions	Prophage functions	holin, putative	NA	NA	NA	NA	NA	NA	NA	NA	NA	NA
	EF0355	*atlB*	Mobile and extrachromosomal element functions	Prophage functions	endolysin, putative	-1,5	0,0000279	0,1	0,8123529	-0,5	0,1419658	-0,9	0,0083395	-1,5	**0,0000061**
	EF0356		Hypothetical proteins		hypothetical protein	NA	NA	-0,3	0,5976889	-1,1	0,0012226	-0,3	0,4282091	-0,3	0,3198087
	EF0357		Hypothetical proteins	Conserved	conserved hypothetical protein	2,4	**0**	-0,2	0,7353246	0,9	0,0039498	0,3	0,3095418	-0,1	0,8604815
	EF0358		Unknown function	Enzymes of unknown specificity	glyoxalase family protein	2,5	**0**	0,2	0,6031872	1,1	0,0003514	0,8	0,0124709	-0,9	0,0042085
	EF0359	*sugE-1*	Protein fate	Protein folding and stabilization	sugE protein	0,8	0,0699087	1,9	0,0008808	NA	NA	NA	NA	-0,9	0,008253
	EF0360	*sugE-2*	Protein fate	Protein folding and stabilization	sugE protein	0,8	0,0851224	0,4	0,3378421	-0,2	0,5096272	0,0	0,8977151	0,1	0,7488899
	EF0361		Cell envelope	Biosynthesis and degradation of surface poly/liposaccharides	chitinase, family 2	4,2	**0**	1,6	0,0004974	1,2	0,0002217	0,8	0,0177331	-1,4	0,0000426
	EF0362		Cell envelope	Biosynthesis and degradation of surface poly/liposaccharides	chitin binding protein, putative	4,4	**0**	1,6	0,0004045	1,0	0,0015348	0,8	0,0161749	-1,0	0,0017747
	EF0363		Mobile and extrachromosomal element functions	Transposon functions	ISEf1, transposase	-2,4	**0**	-0,5	0,2777235	-0,2	0,571122	-0,6	0,0847541	-0,1	0,789972
	EF0365		Hypothetical proteins	Conserved	conserved hypothetical protein	-0,3	0,3777608	-0,1	0,7672913	0,0	0,95356	-0,1	0,801323	-0,1	0,7799731
	EF0366		Hypothetical proteins	Conserved	conserved hypothetical protein	-0,1	0,7283252	-0,2	0,6973512	0,0	0,9163252	0,1	0,7235975	-0,3	0,3499748
	EF0367		Hypothetical proteins	Conserved	conserved hypothetical protein	0,7	0,0632362	-0,3	0,5120376	0,1	0,7344824	0,3	0,4557011	-0,2	0,5776508
	EF0368		Amino acid biosynthesis	Aspartate family	aspartate kinase	0,5	0,1619296	0,6	0,1889672	0,0	0,9178192	0,0	0,9608865	0,8	0,0178045
	EF0369		Unknown function	Enzymes of unknown specificity	hydrolase, haloacid dehalogenase-like family	-1,4	0,0000931	-0,7	0,1147465	0,2	0,5633218	0,6	0,0762277	0,1	0,844887
	EF0370		Hypothetical proteins	Conserved	conserved hypothetical protein	-1,6	**0,0000075**	-0,4	0,3760189	0,1	0,750857	0,2	0,47224	-0,1	0,8500548
	EF0371		Unknown function	Enzymes of unknown specificity	aminotransferase, class V	-1,7	**0,0000063**	-1,5	0,0013017	-0,5	0,1323399	-0,4	0,1875242	-0,7	0,0463704
	EF0372		Signal transduction	Two-component systems	DNA-binding response regulator	0,5	0,1684132	0,4	0,3705836	0,5	0,1374661	-0,2	0,5003404	-0,3	0,4316233
	EF0373		Signal transduction	Two-component systems	sensor histidine kinase	3,0	**0**	0,7	0,1470988	1,1	0,0007608	1,0	0,0041084	-0,7	0,0384506
	EF0374		Cell envelope	Other	lipoprotein, putative	NA	NA	0,1	0,88418	NA	NA	NA	NA	-1,3	0,0001446
	EF0375		Hypothetical proteins		hypothetical protein	-1,0	0,0233689	0,4	0,4003759	-0,2	0,5145393	-0,3	0,4645935	0,2	0,5982994
	EF0376		Hypothetical proteins		hypothetical protein	0,5	0,233425	1,1	0,0613222	-0,1	0,7045756	0,1	0,803854	-0,3	0,4264259
	EF0377		Unknown function	General	ankyrin repeat family protein	0,1	0,8016495	0,0	0,9228628	1,2	0,0000975	0,3	0,3618589	-0,9	0,0079212
	EF0379		Unknown function	General	death-on-curing family protein	1,8	**0,0000009**	0,8	0,0974707	0,0	0,8850249	-0,3	0,3409118	0,0	0,9653382
	EF0380		Hypothetical proteins	Conserved	conserved hypothetical protein	NA	NA	NA	NA	NA	NA	NA	NA	NA	NA
	EF0381		Hypothetical proteins	Conserved	conserved hypothetical protein	NA	NA	-0,1	0,8087026	-0,9	0,005298	-1,3	0,0018189	0,1	0,680699
	EF0382		Hypothetical proteins	Conserved	conserved hypothetical protein	1,3	0,0006305	0,4	0,3754476	0,4	0,26345	0,0	0,9454251	-0,2	0,6418153
	EF0383		Hypothetical proteins	Conserved	protein FdrA/conserved hypothetical protein	4,0	**0**	0,6	0,2052955	0,4	0,2295122	1,1	0,0010067	-0,3	0,3140232
	EF0384		Hypothetical proteins		hypothetical protein	3,2	**0**	0,8	0,0971535	-0,5	0,0997137	1,4	0,0000312	-1,3	0,0001006
	EF0385		Transport and binding proteins	Unknown substrate	major facilitator family transporter	3,9	**0**	2,0	0,0000167	0,2	0,6000121	0,6	0,0968578	-0,7	0,027319
	EF0386	*arcC-2*	Energy metabolism	Amino acids and amines	carbamate kinase	2,6	**0**	1,0	0,0354683	-0,1	0,7860416	0,5	0,1755002	-0,7	0,0281189
	EF0387		Transport and binding proteins	Carbohydrates, organic alcohols, and acids	sodium/dicarboxylate symporter family protein	2,8	**0**	0,9	0,1176309	0,8	0,0101627	0,7	0,027166	-0,3	0,3764301
	EF0388	*allD*	Energy metabolism	Other	ureidoglycolate dehydrogenase	3,4	**0**	0,5	0,3108381	0,7	0,0188078	0,9	0,0049778	-0,1	0,7012353
	EF0389		Cell envelope	Other	membrane protein, putative	2,5	**0**	0,9	0,0960523	0,1	0,6565932	0,5	0,1402449	-0,4	0,1997175
	EF0390		Unknown function	Enzymes of unknown specificity	N-acyl-D-amino-acid deacylase family protein	2,6	**0**	0,1	0,8555584	0,5	0,0976805	0,9	0,0046003	-0,1	0,7604668
	EF0392		Hypothetical proteins		hypothetical protein	0,8	0,0860588	0,4	0,3382717	0,4	0,1560318	0,3	0,4395241	-0,1	0,8143715
	EF0393		Hypothetical proteins		hypothetical protein	NA	NA	NA	NA	NA	NA	NA	NA	NA	NA
	EF0394	*salB*	Protein fate	Other	secreted antigen, putative	-1,6	**0,0000127**	-2,0	**0,0000094**	0,4	0,2212073	0,2	0,4812005	-1,0	0,0034804
	EF0395		Amino acid biosynthesis	Aspartate family	methionine synthase, putative	1,3	0,0003828	0,8	0,095762	-0,5	0,1521039	-0,2	0,546349	0,4	0,2353783
	EF0396		Hypothetical proteins	Conserved	conserved hypothetical protein	1,3	0,000406	0,5	0,2503744	0,1	0,8351152	0,2	0,5300521	-0,1	0,687942
	EF0397		Hypothetical proteins	Conserved	conserved hypothetical protein	1,3	0,0003807	0,7	0,1321939	0,1	0,8700931	0,1	0,7889669	-0,2	0,4825303
	EF0398		Hypothetical proteins		hypothetical protein	NA	NA	NA	NA	NA	NA	NA	NA	NA	NA
	EF0399		Hypothetical proteins	Conserved	conserved hypothetical protein	-2,0	**0**	-0,9	0,0604009	0,0	0,8833142	0,1	0,7575143	0,2	0,6337091
	EF0400		Hypothetical proteins	Conserved	conserved hypothetical protein	-2,4	**0**	-0,4	0,4001589	-0,3	0,332303	-0,7	0,0457405	0,0	0,9328718
	EF0401	*pcp*	Protein fate	Degradation of proteins, peptides, and glycopeptides	pyrrolidone-carboxylate peptidase	-1,6	**0,0000137**	-0,4	0,4103916	0,0	0,9256725	-0,3	0,3718866	0,2	0,5131333
	EF0402	*nhaC-1*	Transport and binding proteins	Cations and iron carrying compounds	Na+/H+ antiporter	-1,3	0,0004384	0,4	0,3882001	0,4	0,2414494	1,0	0,0036944	0,5	0,1436347
	EF0403		Cellular processes	Toxin production and resistance	transcriptional regulator, MarR family	-0,1	0,8714438	0,7	0,1313777	0,0	0,8873989	0,1	0,8232783	0,0	0,9401116
	EF0404		Unknown function	Enzymes of unknown specificity	nitroreductase family protein	-0,3	0,4378783	-0,4	0,4387401	0,6	0,0498547	0,7	0,0395512	0,0	0,9942617
	EF0405		Unknown function	Enzymes of unknown specificity	hydrolase, haloacid dehalogenase-like family	0,9	0,0098192	1,2	0,0118311	0,5	0,1188239	0,4	0,2755257	-0,2	0,4687723
	EF0406		Signal transduction	PTS	PTS system, IIBC component	0,3	0,4849181	0,8	0,0949327	0,9	0,0056733	0,7	0,0253719	0,3	0,4165544
	EF0407		Regulatory functions	Other	transcriptional regulator, putative	-0,7	0,0408358	-0,3	0,5320782	0,6	0,0727819	0,2	0,5136306	-0,5	0,135278
	EF0408		Signal transduction	PTS	PTS system, IIA component	0,2	0,5959964	0,4	0,4475512	0,7	0,0186889	0,5	0,132903	0,1	0,7300443
	EF0409		Hypothetical proteins		hypothetical protein	0,6	0,0912312	0,5	0,2952826	NA	NA	0,2	0,5664619	-0,9	0,0043443
	EF0411		Signal transduction	PTS	PTS system, mannitol-specfic IIBC components	-0,3	0,3987978	3,8	**0**	2,0	**0**	0,1	0,77962	1,6	**0,0000012**
	EF0412	*mltF*	Signal transduction	PTS	PTS system, mannitol-specific IIA component	0,2	0,5445892	2,4	0,000019	3,4	**0**	1,8	**0,0000002**	0,8	0,0233832
	EF0413	*mtlD*	Energy metabolism	Sugars	mannitol-1-phosphate 5-dehydrogenase	1,9	**0,0000002**	4,5	**0**	2,8	**0**	1,4	0,0000274	0,4	0,2000267
	EF0414		Unknown function	Enzymes of unknown specificity	oxidoreductase, DadA family	-0,5	0,2102214	-0,8	0,0681643	0,5	0,1485033	0,6	0,0762954	-0,1	0,8046793
	EF0415		Hypothetical proteins	Conserved	conserved hypothetical protein	NA	NA	0,4	0,366919	0,2	0,5593189	1,5	**0,0000039**	-0,4	0,2277457
	EF0417		Hypothetical proteins	Conserved	conserved hypothetical protein	-1,2	0,0012017	0,8	0,0895791	-0,6	0,0465727	0,0	0,9981809	-0,1	0,8017391
	EF0419		Hypothetical proteins	Conserved	conserved hypothetical protein	-2,6	**0**	-0,6	0,1805593	0,4	0,1690007	-0,8	0,020442	-0,1	0,7900542
	EF0420		Transport and binding proteins	Cellular processes	drug resistance transporter, EmrB/QacA family protein	-0,7	0,0576291	-0,6	0,2295595	-0,1	0,8300356	-0,1	0,7321759	-0,1	0,7927413
	EF0421		Regulatory functions	DNA interactions	transcriptional regulator, MerR family	1,0	0,0080089	-0,2	0,6787175	0,8	0,0088192	0,2	0,4972257	-0,1	0,8175685
	EF0422		Regulatory functions	DNA interactions	transcriptional regulator, IclR family	0,0	0,9982041	0,5	0,3121694	-0,5	0,1312781	-0,9	0,0109512	0,0	0,9838328
	EF0423	*eda-1*	Energy metabolism	Entner-Doudoroff	2-dehydro-3-deoxyphosphogluconate aldolase	1,6	0,000016	-0,3	0,5656766	0,3	0,3196333	1,0	0,0031567	-0,1	0,6959026
	EF0424		Energy metabolism	Entner-Doudoroff	2-dehydro-3-deoxygluconokinase, putative	0,2	0,7253789	0,9	0,054927	-0,3	0,3347315	-0,8	0,0137799	0,1	0,6986436
	EF0425	*kduI-1*	Energy metabolism	Biosynthesis and degradation of polysaccharides	4-deoxy-l-threo-5-hexosulose-uronate ketol-isomerase	0,8	0,0611316	0,4	0,3584648	-0,9	0,0062175	-0,8	0,0146617	-0,1	0,7813973
	EF0426		Central intermediary metabolism	Other	gluconate 5-dehydrogenase, putative	NA	NA	-0,7	0,2388375	-0,8	0,0519827	-0,8	0,0133672	-0,2	0,5548476
	EF0428		Hypothetical proteins	Conserved	conserved hypothetical protein	-0,1	0,870568	0,0	0,9490015	-0,4	0,2566486	-0,5	0,1772317	-0,5	0,1309248
	EF0429		Transport and binding proteins	Carbohydrates, organic alcohols, and acids	TRAP dicarboxylate transporter, DctP subunit	NA	NA	NA	NA	NA	NA	NA	NA	0,1	0,8576624
	EF0430		Transport and binding proteins	Carbohydrates, organic alcohols, and acids	TRAP dicarboxylate transporter, DctQ subunit	NA	NA	0,9	0,0599241	NA	NA	0,1	0,8257331	-0,2	0,6427594
	EF0431		Transport and binding proteins	Carbohydrates, organic alcohols, and acids	TRAP dicarboxylate transporter, DctM subunit	-0,1	0,7205644	-1,2	0,0072848	1,1	0,0005718	-0,4	0,2168462	0,0	0,9017848
	EF0432		Regulatory functions	DNA interactions	transcriptional regulator, AraC family	1,5	0,0008247	0,1	0,8755036	-0,5	0,1614588	-0,1	0,7807826	0,4	0,2906224
	EF0433		Energy metabolism	Sugars	rhamnulokinase, putative	0,9	0,0163785	1,0	0,0868495	NA	NA	0,9	0,0081512	-0,6	0,0501883
	EF0434	*rhaA*	Energy metabolism	Sugars	L-rhamnose isomerase	NA	NA	0,9	0,0532088	NA	NA	NA	NA	-0,3	0,4221289
	EF0435	*rhaD*	Energy metabolism	Sugars	rhamnulose-1-phosphate aldolase	NA	NA	1,4	0,0106981	NA	NA	0,3	0,4272048	-0,2	0,5072313
	EF0436		Hypothetical proteins	Conserved	conserved hypothetical protein	NA	NA	0,5	0,3726394	NA	NA	-0,3	0,5067885	0,3	0,4550893
	EF0437		Regulatory functions	DNA interactions	transcriptional regulator, AraC family	1,1	0,0020204	0,0	0,9171919	NA	NA	-0,1	0,7438	0,0	0,9805764
	EF0438		Hypothetical proteins	Domain	conserved domain protein	0,9	0,0432667	0,4	0,3389812	-0,1	0,7861029	-0,1	0,8025474	0,1	0,7740839
	EF0439		Cellular processes	Toxin production and resistance	immunity protein PlnM, putative	1,0	0,0065263	0,8	0,0712401	0,3	0,3004837	-0,3	0,3263365	-0,7	0,0429121
	EF0440		Transport and binding proteins	Amino acids, peptides and amines	di-/tripeptide transporter	-3,0	**0**	-0,8	0,0778397	-0,4	0,2667	-0,8	0,0142935	-0,1	0,6887205
	EF0441		Hypothetical proteins		hypothetical protein	NA	NA	NA	NA	NA	NA	NA	NA	NA	NA
	EF0442		Hypothetical proteins		hypothetical protein	NA	NA	NA	NA	NA	NA	NA	NA	NA	NA
	EF0443		Unknown function	General	LysM domain protein	-2,8	**0**	-2,3	**0,0000004**	0,6	0,0448644	-0,1	0,8538459	0,0	0,9743461

Operon[a]	Locus	Gene	Functional category	Subcategory	Putative function	Blood[b]	P-value[c]	YTB_5[b]	P-value[c]	YTB_15[b]	P-value[c]	YTB_30[b]	P-value[c]	YTB_60[b]	P-value[c]
	EF0445	*menB*	Biosynthesis of cofactors, prosthetic groups, carriers	Menaquinone and ubiquinone	naphthoate synthase	-1,2	0,0007142	-0,7	0,1170511	0,2	0,5965236	0,3	0,3552288	0,0	0,9085376
	EF0446		Biosynthesis of cofactors, prosthetic groups, carriers	Menaquinone and ubiquinone	O-succinylbenzoic acid--CoA ligase, putative	-2,5	**0**	-0,8	0,098497	-0,1	0,725364	-0,3	0,3111495	-0,2	0,5151856
	EF0447		Biosynthesis of cofactors, prosthetic groups, carriers	Menaquinone and ubiquinone	menaquinone-specific isochorismate synthase, putative	-2,6	**0**	-0,6	0,2087045	0,4	0,2420486	0,3	0,3282437	-0,1	0,717241
	EF0448	*menD*	Biosynthesis of cofactors, prosthetic groups, carriers	Menaquinone and ubiquinone	2-succinyl-6-hydroxy-2,4-cyclohexadiene-1-carboxylic acid synthase	-2,3	**0**	-0,9	0,0614209	0,4	0,2375013	-0,3	0,3647832	-0,2	0,5305692
	EF0449		Unknown function	Enzymes of unknown specificity	hydrolase, alpha/beta hydrolase fold family	-2,3	**0**	-0,4	0,3982792	0,2	0,5444961	-0,5	0,1780495	-0,1	0,6716771
	EF0450		Energy metabolism	Other	mandelate racemase/muconate lactonizing enzyme family protein	-1,8	**0,0000016**	-0,2	0,6932906	0,3	0,2832663	-0,4	0,2424182	0,1	0,6606475
	EF0451		Central intermediary metabolism	Amino sugars	glucosamine-6-phosphate isomerase, putative	-2,0	**0,0000001**	-0,5	0,3107135	0,4	0,2506813	-0,7	0,0269585	0,1	0,8459352
	EF0452		Unknown function	General	AMP-binding family protein	-3,3	**0**	-1,8	0,0000734	-2,0	**0**	-0,8	0,0126478	-0,2	0,6212706
	EF0453	*ohr*	Unknown function	General	OsmC/Ohr family protein	3,2	**0**	0,7	0,1446052	0,8	0,0119016	0,6	0,0760616	0,0	0,9040664
	EF0454		Hypothetical proteins	Conserved	conserved hypothetical protein	1,9	**0,0000004**	0,8	0,0876913	0,8	0,0136003	-0,3	0,435883	0,2	0,5188797
	EF0455		Signal transduction	PTS	PTS system, IIC component	1,0	0,0120225	0,8	0,0720266	0,5	0,1100245	-0,4	0,2096767	-0,2	0,4900415
	EF0456		Signal transduction	PTS	PTS system, IID component	1,6	**0,0000137**	0,9	0,0506407	-0,2	0,488446	-0,3	0,3722079	-0,4	0,1999479
	EF0457		Signal transduction	PTS	PTS system, IIB component	0,0	0,9961548	-0,7	0,1343864	0,1	0,8576775	-0,2	0,6126712	-0,2	0,6458094
	EF0458		Regulatory functions	Other	phosphosugar-binding transcriptional regulator, putative	0,8	0,0265378	0,0	0,9288911	0,5	0,1279306	-0,4	0,1959265	-0,4	0,1853515
	EF0459		Unknown function	General	glucokinase regulator-related protein	1,2	0,0009592	0,4	0,378887	0,0	0,8967164	-0,5	0,1263523	-0,5	0,1659885
	EF0460		Hypothetical proteins	Conserved	conserved hypothetical protein	1,2	0,0009685	0,1	0,7613182	0,5	0,1193913	0,3	0,3101804	-0,5	0,1067063
	EF0461		Signal transduction	PTS	PTS system, IIA component	0,4	0,3363198	0,0	0,9610246	0,1	0,7359765	-0,3	0,3962116	-0,2	0,5039111
	EF0462		Hypothetical proteins	Domain	conserved domain protein	-1,2	0,0015328	-0,3	0,4625305	0,0	0,9811586	0,4	0,259463	-0,3	0,3116933
	EF0463	*sodA*	Cellular processes	Detoxification	superoxide dismutase, Mn	1,7	**0,0000028**	0,1	0,7873599	1,0	0,0024392	0,8	0,012985	-0,6	0,0655221
	EF0464	*dapF*	Amino acid biosynthesis	Aspartate family	diaminopimelate epimerase	-0,4	0,324263	0,1	0,8548318	0,8	0,0084565	0,5	0,1387596	0,2	0,6080754
	EF0465		Regulatory functions	Other	transcriptional regulator	-1,0	0,0084553	-0,5	0,2960204	0,0	0,886733	0,2	0,6543453	-0,1	0,6906856
	EF0466	*nagB*	Central intermediary metabolism	Amino sugars	glucosamine-6-phosphate isomerase	1,5	0,0008533	0,8	0,1017951	0,9	0,0072996	1,0	0,0041677	0,1	0,8460115
	EF0467		Transport and binding proteins	Cations and iron carrying compounds	MgtC family protein	NA	NA	0,4	0,4148583	NA	NA	-0,8	0,0118828	-0,2	0,5027329
	EF0468		Unknown function	General	LemA family protein	1,0	0,0047136	0,4	0,4355947	-0,9	0,0043624	-0,4	0,2610126	0,7	0,0421624
	EF0469		Hypothetical proteins	Domain	conserved domain protein	2,4	**0**	3,6	**0**	0,3	0,292736	1,2	0,0002604	1,4	0,000034
	EF0470	*nrdF*	Purines, pyrimidines, nucleosides, and nucleotides	2'-Deoxyribonucleotide metabolism	ribonucleoside-diphosphate reductase 2, beta subunit	-2,4	**0**	-0,7	0,1595647	0,0	0,983467	-0,7	0,027656	-0,4	0,1810171
	EF0471	*nrdE*	Purines, pyrimidines, nucleosides, and nucleotides	2'-Deoxyribonucleotide metabolism	ribonucleoside-diphosphate reductase 2, alpha subunit	-1,5	0,0000502	-0,6	0,164897	0,1	0,7957135	-1,0	0,0018965	-0,5	0,1475037
	EF0472	*nrdI*	Purines, pyrimidines, nucleosides, and nucleotides	2'-Deoxyribonucleotide metabolism	nrdI protein	-1,0	0,0220258	-1,9	0,0000293	0,1	0,7225538	-0,3	0,4441005	-0,4	0,2178034
	EF0473	*nrdH*	Purines, pyrimidines, nucleosides, and nucleotides	2'-Deoxyribonucleotide metabolism	ribonucleoside-diphosphate reductase 2, NrdH-redoxin	-0,1	0,7437437	-1,3	0,0058895	-0,2	0,448799	-0,1	0,8434745	-0,5	0,1654255
	EF0475	*feoA*	Transport and binding proteins	Cations and iron carrying compounds	ferrous iron transport protein A	NA	NA	-1,7	0,0002636	1,1	0,0005669	1,0	0,0032871	0,1	0,7963608
	EF0476	*feoB*	Transport and binding proteins	Cations and iron carrying compounds	ferrous iron transport protein B	1,5	0,0000914	-1,1	0,0139079	1,8	**0**	2,9	**0**	-0,4	0,2260947
	EF0477		Hypothetical proteins		hypothetical protein	NA	NA	NA	NA	NA	NA	NA	NA	NA	NA
	EF0478		Hypothetical proteins		hypothetical protein	NA	NA	NA	NA	NA	NA	NA	NA	NA	NA
	EF0479		DNA metabolism	DNA replication, recombination, and repair	site-specific recombinase, phage integrase family	-0,5	0,1502705	-1,5	0,001139	0,5	0,1090247	0,7	0,0372797	0,9	0,0046279
	EF0480		Mobile and extrachromosomal element functions	Prophage functions	excisionase, putative	0,9	0,0232835	0,4	0,440949	-1,2	0,0001232	NA	NA	0,0	0,9548717
	EF0481		Hypothetical proteins	Conserved	conserved hypothetical protein	1,1	0,0138241	0,7	0,1404505	-0,7	0,0258827	-0,3	0,4081967	-0,2	0,6169844
	EF0482		Hypothetical proteins	Conserved	conserved hypothetical protein	NA	NA	0,5	0,3564232	NA	NA	NA	NA	0,6	0,0922034
	EF0485		Cellular processes	Conjugation	aggregation substance	0,6	0,1526159	0,3	0,4941978	-1,2	0,0022938	NA	NA	0,3	0,3128534
	EF0486		Hypothetical proteins		hypothetical protein	1,3	0,0008816	NA	NA	NA	NA	NA	NA	NA	NA
	EF0487		Hypothetical proteins	Domain	conserved domain protein	NA	NA	NA	NA	NA	NA	NA	NA	NA	NA
	EF0488		Hypothetical proteins		hypothetical protein	NA	NA	0,7	0,1145916	NA	NA	-0,8	0,0146248	0,3	0,3670531
	EF0489		Hypothetical proteins	Domain	conserved domain protein	NA	NA	NA	NA	NA	NA	NA	NA	NA	NA
	EF0490		Cell envelope	Other	cell wall surface anchor family protein	0,4	0,3241496	-0,6	0,2170329	NA	NA	NA	NA	0,6	0,0723596
	EF0491		Hypothetical proteins	Domain	conserved domain protein	NA	NA	NA	NA	NA	NA	NA	NA	NA	NA
	EF0492		Hypothetical proteins		hypothetical protein	NA	NA	NA	NA	NA	NA	NA	NA	NA	NA
	EF0493		Hypothetical proteins	Conserved	conserved hypothetical protein	0,1	0,8560076	0,5	0,2649726	-1,1	0,0032075	-0,2	0,4970509	0,3	0,4071828
	EF0494		Hypothetical proteins	Conserved	conserved hypothetical protein	1,2	0,0016289	0,6	0,2027761	-0,6	0,073971	-0,2	0,4583502	0,1	0,6834065
	EF0495		Hypothetical proteins	Domain	conserved domain protein	NA	NA	0,1	0,7621884	NA	NA	NA	NA	0,1	0,7031667
	EF0496		Hypothetical proteins	Conserved	conserved hypothetical protein	0,6	0,1599338	0,7	0,122704	NA	NA	-0,5	0,1032275	0,1	0,8604824
	EF0497		Hypothetical proteins	Conserved	conserved hypothetical protein	0,7	0,0720023	0,3	0,4561318	-0,5	0,2381588	-0,3	0,3718351	0,2	0,4906092
	EF0498		Hypothetical proteins		hypothetical protein	0,9	0,025107	0,4	0,4130866	-0,8	0,014773	0,3	0,4335058	0,4	0,2732248
	EF0499	*ssb-2*	DNA metabolism	DNA replication, recombination, and repair	single-strand binding protein	0,1	0,7793405	0,5	0,3123417	-0,2	0,4510983	-0,1	0,7617791	0,1	0,6900114
	EF0500		Hypothetical proteins	Conserved	conserved hypothetical protein	NA	NA	NA	NA	NA	NA	NA	NA	NA	NA
	EF0501		Cell envelope	Other	lipoprotein, putative	NA	NA	0,2	0,7085403	0,5	0,208054	NA	NA	0,7	0,0436576
	EF0502		Cell envelope	Other	membrane protein, putative	0,0	0,9929184	NA	NA	NA	NA	-0,6	0,090626	0,5	0,1548029
	EF0503		Hypothetical proteins		hypothetical protein	1,0	0,0096531	NA	NA	NA	NA	NA	NA	0,3	0,3954016
	EF0504		Hypothetical proteins		hypothetical protein	1,0	0,0096589	0,5	0,3507877	-0,6	0,1035377	0,2	0,62119	0,0	0,9647727
	EF0505		Hypothetical proteins		hypothetical protein	0,7	0,0712979	-0,3	0,6022041	NA	NA	NA	NA	-1,2	0,0001654
	EF0506		Hypothetical proteins		hypothetical protein	NA	NA	0,8	0,0813976	NA	NA	NA	NA	0,9	0,0075577
	EF0507		Hypothetical proteins		hypothetical protein	NA	NA	NA	NA	NA	NA	NA	NA	0,0	0,9146814
	EF0508		Hypothetical proteins	Domain	conserved domain protein	-0,1	0,8651034	0,4	0,5173868	0,7	0,0412823	-0,3	0,3284265	0,0	0,9449916
	EF0509		Hypothetical proteins	Conserved	conserved hypothetical protein	0,9	0,0359364	0,9	0,1039237	NA	NA	0,4	0,2493243	0,3	0,39108
	EF0510	*ssb-3*	DNA metabolism	DNA replication, recombination, and repair	single-strand binding protein	1,0	0,0068782	-0,3	0,5650907	0,2	0,5027983	0,4	0,2093822	0,2	0,6207774
	EF0511	*nuc-1*	DNA metabolism	DNA replication, recombination, and repair	thermonuclease precursor	1,1	0,0122146	0,6	0,1863408	-0,2	0,4629318	0,0	0,9346746	0,8	0,0220301
	EF0512		Unknown function	General	DNA-damage-inducible protein J, putative	NA	NA	NA	NA	NA	NA	NA	NA	NA	NA
	EF0513		Hypothetical proteins	Conserved	conserved hypothetical protein TIGR00053	NA	NA	0,2	0,6832413	NA	NA	NA	NA	0,5	0,1144417
	EF0516		Cell envelope	Other	membrane protein, putative	-2,3	**0**	-1,4	0,0023741	-1,9	**0**	-1,4	**0,0000158**	-0,3	0,4093163
	EF0517		Biosynthesis of cofactors, prosthetic groups, carriers	Pantothenate and coenzyme A	2-dehydropantoate 2-reductase, putative	-3,7	**0**	-1,4	0,0020966	-2,3	**0**	-1,5	**0,0000124**	-0,2	0,4843345
	EF0518		Cell envelope	Other	cell wall surface anchor family protein	-0,8	0,0798145	0,3	0,5303262	NA	NA	-0,8	0,0181088	0,2	0,5553391
	EF0519		Hypothetical proteins		hypothetical protein	NA	NA	NA	NA	NA	NA	NA	NA	NA	NA
	EF0520		Disrupted reading frame		IS1216, transposase, degenerate	0,8	0,0222683	0,3	0,5390139	0,6	0,0471554	0,3	0,313843	0,2	0,6098533
	EF0521		Energy metabolism	Other	choloylglycine hydrolase family protein	1,3	0,000477	0,4	0,3585203	-0,1	0,8210111	0,2	0,4812523	-0,1	0,6787808
	EF0523		Hypothetical proteins		hypothetical protein	-0,7	0,0589963	0,6	0,1768882	0,5	0,1276378	-0,3	0,4265377	-0,1	0,8227206
	EF0524		Regulatory functions	DNA interactions	transcriptional regulator, Cro/CI family	NA	NA	0,2	0,7043291	NA	NA	NA	NA	-0,2	0,4657339
	EF0525		Unknown function	General	cylL-L protein	1,0	0,0065231	0,3	0,469564	0,3	0,3317034	0,5	0,1690577	0,3	0,3132721
	EF0526		Unknown function	General	cylL-S protein	0,7	0,0454246	0,0	0,9489484	0,4	0,1537508	-0,1	0,861529	0,4	0,2789409
	EF0527	*cylM*	Cellular processes	Toxin production and resistance	cylM protein	0,2	0,591071	0,9	0,0632581	0,1	0,8693083	-0,2	0,6371343	0,3	0,3506503
	EF0528		Disrupted reading frame		cytolysin B transport protein, truncation	NA	NA	-0,3	0,6197165	NA	NA	-0,5	0,185136	0,3	0,3878715
	EF0529		Mobile and extrachromosomal element functions	Transposon functions	IS256, transposase	0,1	0,8240243	-0,5	0,2734838	-0,2	0,5651915	-0,1	0,8814625	-0,1	0,6701734
	EF0530		Regulatory functions	DNA interactions	transcriptional regulator, AraC family	-0,5	0,3128426	-0,1	0,7884188	0,2	0,4534826	-0,4	0,279647	0,1	0,7658898
	EF0531		Hypothetical proteins		hypothetical protein	NA	NA	NA	NA	NA	NA	NA	NA	NA	NA
	EF0532		Hypothetical proteins		hypothetical protein	NA	NA	NA	NA	NA	NA	NA	NA	NA	NA
	EF0533		Hypothetical proteins		hypothetical protein	-0,2	0,586944	-0,6	0,1778164	0,5	0,0966067	0,1	0,7760812	0,1	0,7748538

Operon[a]	Locus	Gene	Functional category	Subcategory	Putative function	Blood[b]	P-value[c]	YTB_5[b]	P-value[c]	YTB_15[b]	P-value[c]	YTB_30[b]	P-value[c]	YTB_60[b]	P-value[c]
	EF0534		DNA metabolism	DNA replication, recombination, and repair	site-specific recombinase, resolvase family	-0,3	0,4929546	-0,2	0,6877995	0,7	0,0223441	0,5	0,1440639	0,3	0,3149988
	EF0539		Regulatory functions	DNA interactions	phosphosugar-binding transcriptional regulator, RpiR family	0,5	0,1547315	-0,1	0,8536687	-0,1	0,7503793	-0,2	0,57737	0,2	0,6224278
	EF0540		Central intermediary metabolism	Amino sugars	N-acetylmannosamine-6-phosphate epimerase, putative	0,5	0,1999564	0,1	0,8939747	-0,4	0,2332752	-0,2	0,571569	-0,4	0,1897313
	EF0542		Hypothetical proteins	Conserved	conserved hypothetical protein	NA	NA	0,2	0,6821563	NA	NA	-0,9	0,0054907	0,0	0,9081333
	EF0543		Cell envelope	Other	membrane protein, putative	0,3	0,5037707	0,3	0,572062	NA	NA	-1,3	0,0001902	0,2	0,6073934
	EF0544	*rpmF-1*	Protein synthesis	Ribosomal proteins: synthesis and modification	ribosomal protein L32	NA	NA	NA	NA	NA	NA	NA	NA	NA	NA
	EF0545		Hypothetical proteins	Domain	conserved domain protein	NA	NA	0,7	0,2074581	NA	NA	-0,8	0,0159826	0,0	0,9456214
	EF0546		Hypothetical proteins	Conserved	conserved hypothetical protein	0,4	0,389207	0,5	0,3283511	-0,4	0,170104	0,0	0,8895758	0,2	0,5374855
	EF0547		Transport and binding proteins	Cations and iron carrying compounds	ammonium transporter family protein	-1,2	0,0082614	0,2	0,706365	-0,3	0,2841802	-0,9	0,0372414	0,0	0,9741536
	EF0548		Hypothetical proteins	Conserved	conserved hypothetical protein	NA	NA	0,0	0,9610477	NA	NA	-1,0	0,0186597	0,2	0,6466328
	EF0550		Regulatory functions	DNA interactions	xylose repressor, putative	-1,2	0,0099419	-0,7	0,1536605	0,7	0,0175605	0,4	0,2925097	0,0	0,9614287
	EF0551		Energy metabolism	Biosynthesis and degradation of polysaccharides	glycosyl hydrolase, family 31	NA	NA	NA	NA	NA	NA	-0,8	0,0524298	NA	NA
	EF0552		Signal transduction	PTS	PTS system, IIC component	0,2	0,6632988	0,7	0,1093667	-0,5	0,1121095	-0,2	0,4714857	0,1	0,8075882
	EF0553		Signal transduction	PTS	PTS system, IID component	1,4	0,0002549	1,2	0,0116182	0,4	0,1900505	-0,5	0,1581584	-1,1	0,0012574
	EF0554		Signal transduction	PTS	PTS system, IIB component	NA	NA	1,2	0,0081554	NA	NA	-0,6	0,0564148	-0,2	0,5518796
	EF0555		Signal transduction	PTS	PTS system, IIA component	NA	NA	0,9	0,0566835	0,1	0,6684685	0,4	0,2563751	-0,2	0,5946974
	EF0556	*xylA*	Energy metabolism	Sugars	xylose isomerase	NA	NA	0,5	0,2495871	-0,8	0,0322346	-1,0	0,0026342	-0,2	0,5008852
	EF0557	*xylB*	Energy metabolism	Sugars	D-xylulose kinase	0,2	0,6448005	0,5	0,2649407	NA	NA	-0,7	0,029948	0,2	0,5475291
	EF0559		Cell envelope	Biosynthesis and degradation of surface poly/liposaccharides	polysaccharide biosynthesis family protein	1,1	0,0145522	0,3	0,4969586	NA	NA	NA	NA	0,1	0,652758
	EF0562		Hypothetical proteins		hypothetical protein	NA	NA	0,8	0,0797094	-0,3	0,3917703	-0,3	0,3752001	0,3	0,4331305
	EF0563		Hypothetical proteins		hypothetical protein	NA	NA	NA	NA	NA	NA	NA	NA	NA	NA
	EF0564		Hypothetical proteins		hypothetical protein	NA	NA	NA	NA	NA	NA	NA	NA	NA	NA
	EF0566.1	*kdpF*	Transport and binding proteins	Cations and iron carrying compounds	potassium transporter stabilization peptide KdpF	0,3	0,4817228	0,9	0,0589344	-1,0	0,0011493	-1,2	0,0003703	-0,6	0,0820897
	EF0567	*kdpA*	Transport and binding proteins	Cations and iron carrying compounds	potassium-transporting ATPase, subunit A	-0,3	0,3578823	-1,2	0,0119257	1,0	0,0022998	0,8	0,0193438	-0,1	0,8417854
	EF0568	*kdpB*	Transport and binding proteins	Cations and iron carrying compounds	potassium-transporting ATPase, subunit B	NA	NA	0,0	0,960637	0,3	0,4353061	-0,4	0,2706969	-0,1	0,7125838
	EF0569	*kdpC*	Transport and binding proteins	Cations and iron carrying compounds	potassium-transporting ATPase, subunit C	-1,4	0,0023304	-0,9	0,0596625	0,8	0,0162235	0,0	0,9841345	0,0	0,9448047
	EF0570	*kdpD*	Signal transduction	Two-component systems	sensor histidine kinase KdpD	NA	NA	0,6	0,1716663	0,0	0,8938736	0,5	0,2285861	0,1	0,8149292
	EF0571		Signal transduction	Two-component systems	DNA-binding response regulator	0,8	0,0290596	-0,3	0,5586475	1,0	0,002016	0,9	0,0101656	-0,2	0,5649743
	EF0573		Hypothetical proteins		hypothetical protein	NA	NA	0,1	0,8931023	-0,7	0,0736255	NA	NA	0,1	0,6720533
	EF0574		Hypothetical proteins		hypothetical protein	NA	NA	NA	NA	NA	NA	NA	NA	NA	NA
	EF0575		Transport and binding proteins	Cations and iron carrying compounds	cationic ABC transporter, ATP-binding protein	NA	NA	-0,6	0,2776835	NA	NA	NA	NA	0,1	0,687896
	EF0576		Transport and binding proteins	Cations and iron carrying compounds	cation ABC transporter, permease protein	0,2	0,6768676	0,0	0,9966703	0,9	0,0034878	-0,3	0,4363607	0,0	0,9682512
	EF0577		Cellular processes	Cell adhesion	adhesion lipoprotein	1,3	0,005063	0,1	0,8644727	-0,6	0,1110929	-0,5	0,2176713	-0,8	0,0243471
	EF0578		Regulatory functions	DNA interactions	helix-turn-helix protein, iron-dependent repressor family	NA	NA	NA	NA	NA	NA	NA	NA	NA	NA
	EF0579		Regulatory functions	Other	transcriptional regulator, putative	NA	NA	0,9	0,0514124	NA	NA	-0,1	0,8384813	0,2	0,6355961
	EF0580		Hypothetical proteins	Conserved	conserved hypothetical protein	NA	NA	0,4	0,4972066	NA	NA	-0,4	0,2128911	-0,1	0,7912411
	EF0581		Transport and binding proteins	Unknown substrate	ABC transporter, ATP-binding protein	NA	NA	0,4	0,342852	0,1	0,8083561	0,1	0,7069457	0,1	0,7341466
	EF0582		Cell envelope	Other	membrane protein, putative	1,3	0,0004853	0,1	0,8526868	0,6	0,079666	0,5	0,1758637	0,0	0,9211867
	EF0583		Transport and binding proteins	Unknown substrate	ABC transporter, ATP-binding protein/permease protein	1,4	0,0024716	0,1	0,8584417	0,6	0,0661148	-0,2	0,5240169	0,0	0,9890006
	EF0584		Transport and binding proteins	Unknown substrate	ABC transporter, ATP-binding/permease protein	NA	NA	0,7	0,1091774	-0,6	0,0485778	-0,8	0,0120118	-0,2	0,648511
	EF0585	*rpsN-2*	Protein synthesis	Ribosomal proteins: synthesis and modification	ribosomal protein S14	NA	NA	0,9	0,0666228	NA	NA	-0,3	0,396204	0,2	0,6467522
	EF0586	*rpmF-2*	Protein synthesis	Ribosomal proteins: synthesis and modification	ribosomal protein L32	NA	NA	0,0	0,9937445	NA	NA	-0,7	0,0432041	0,2	0,6719362
	EF0587		Hypothetical proteins		hypothetical protein	NA	NA	1,3	0,0077186	NA	NA	-1,8	**0,0000002**	-0,2	0,5577312
	EF0588	*rpmG-1*	Protein synthesis	Ribosomal proteins: synthesis and modification	ribosomal protein L33	0,9	0,0142704	-0,5	0,3371045	-1,2	0,0002934	-1,1	0,0014921	-0,1	0,8450823
	EF0589		Hypothetical proteins	Domain	conserved domain protein	NA	NA	NA	NA	NA	NA	NA	NA	NA	NA
	EF0590		Energy metabolism	Biosynthesis and degradation of polysaccharides	polysaccharide deacetylase family protein	NA	NA	1,2	0,0103814	NA	NA	-0,6	0,0596041	-0,1	0,7752725
	EF0594		Hypothetical proteins		hypothetical protein	NA	NA	NA	NA	NA	NA	NA	NA	NA	NA
	EF0595		Mobile and extrachromosomal element functions	Transposon functions	ISEf1, transposase	-2,6	**0**	-0,6	0,2066995	-0,1	0,7302755	-0,6	0,0576145	0,0	0,9202735
	EF0599		Hypothetical proteins	Domain	conserved domain protein	1,1	0,0128849	0,3	0,621719	-0,4	0,2359614	-0,4	0,2161739	-0,2	0,5782928
	EF0600		Regulatory functions	DNA interactions	transcriptional regulator, TetR family	NA	NA	NA	NA	NA	NA	NA	NA	NA	NA
	EF0601		Regulatory functions	DNA interactions	transcriptional regulator, TetR family	NA	NA	1,2	0,0085513	0,1	0,7742147	0,3	0,3480934	0,5	0,1157306
	EF0603		Hypothetical proteins	Domain	conserved domain protein	NA	NA	0,9	0,0938709	NA	NA	NA	NA	0,2	0,4911263
	EF0604		Cellular processes	Adaptations to atypical conditions	gls24 protein	NA	NA	0,2	0,7715573	NA	NA	NA	NA	0,2	0,6024766
	EF0605		Hypothetical proteins	Conserved	conserved hypothetical protein	NA	NA	0,7	0,203997	NA	NA	NA	NA	0,0	0,888439
	EF0606	*dps*	Cellular processes	Adaptations to atypical conditions	Dps family protein	2,2	**0**	1,1	0,0200702	0,4	0,1704063	1,2	0,0002716	0,6	0,062021
	EF0607		Unknown function	General	ParB-like nuclease domain protein	NA	NA	NA	NA	NA	NA	NA	NA	NA	NA
	EF0608		Hypothetical proteins		hypothetical protein	NA	NA	1,1	0,0207212	NA	NA	0,1	0,6579672	0,3	0,4381465
	EF0609		Hypothetical proteins	Conserved	conserved hypothetical protein	NA	NA	NA	NA	NA	NA	NA	NA	NA	NA
	EF0610		Hypothetical proteins		hypothetical protein	NA	NA	NA	NA	NA	NA	NA	NA	NA	NA
	EF0611		Hypothetical proteins		hypothetical protein	NA	NA	0,7	0,2185784	NA	NA	NA	NA	0,4	0,2051431
	EF0612		Hypothetical proteins		hypothetical protein	0,9	0,0151287	0,3	0,4923779	0,0	0,9309029	0,4	0,2169398	0,4	0,1897364
	EF0613		Hypothetical proteins		hypothetical protein	NA	NA	NA	NA	NA	NA	NA	NA	NA	NA
	EF0615		Mobile and extrachromosomal element functions	Transposon functions	transposase, IS200 family	NA	NA	0,1	0,81872	NA	NA	-0,5	0,1208428	0,3	0,4121505
	EF0616		Energy metabolism	Amino acids and amines	ornithine cyclodeaminase, putative	-1,9	**0,0000003**	-0,9	0,0657826	-1,3	0,0000247	-0,6	0,0672729	-0,2	0,5991509
	EF0617		Cell envelope	Other	membrane protein, putative	-0,9	0,052313	-0,9	0,0514825	-1,3	0,0000501	-0,3	0,2963446	-0,4	0,2738218
	EF0618		Hypothetical proteins	Conserved	conserved hypothetical protein	NA	NA	-0,8	0,0821988	-0,8	0,0133991	-0,5	0,1754299	0,1	0,7766377
	EF0619		Hypothetical proteins		hypothetical protein	NA	NA	NA	NA	NA	NA	NA	NA	NA	NA
	EF0621		Hypothetical proteins		hypothetical protein	NA	NA	NA	NA	NA	NA	NA	NA	NA	NA
	EF0622		Hypothetical proteins		hypothetical protein	NA	NA	NA	NA	NA	NA	NA	NA	NA	NA
	EF0624		Hypothetical proteins		hypothetical protein	NA	NA	-0,3	0,582287	-0,7	0,0610474	-0,4	0,3775352	-0,2	0,6101496
	EF0625		Hypothetical proteins		hypothetical protein	NA	NA	NA	NA	NA	NA	NA	NA	-0,6	0,0572591
	EF0626		Hypothetical proteins		hypothetical protein	NA	NA	NA	NA	NA	NA	NA	NA	NA	NA
	EF0627		Hypothetical proteins		hypothetical protein	NA	NA	0,5	0,4256462	NA	NA	0,2	0,5973573	0,3	0,2976624
	EF0628		Signal transduction	PTS	PTS system, IIA component, putative	NA	NA	0,6	0,2634539	NA	NA	NA	NA	NA	NA
	EF0629		Unknown function	Enzymes of unknown specificity	oxidoreductase, aldo/keto reductase family	0,1	0,8409514	-0,1	0,7994546	-0,8	0,0140381	-0,3	0,3448686	0,0	0,9745802
	EF0630		Unknown function	Enzymes of unknown specificity	glyoxalase family protein	-0,2	0,7302625	-0,5	0,2858626	-1,1	0,0003236	-0,8	0,011768	-0,1	0,8434454
	EF0631		Fatty acid and phospholipid metabolism	Biosynthesis	cardiolipin synthetase, putative	0,0	0,9533031	-0,5	0,2961676	-0,2	0,4820242	-0,3	0,4404386	0,3	0,410751
	EF0633	*tryS-1*	Protein synthesis	tRNA aminoacylation	tyrosyl-tRNA synthetase	-1,9	**0,0000002**	0,3	0,5833692	-0,9	0,0054928	-0,2	0,6223697	0,1	0,7419543
	EF0634		Unknown function	Enzymes of unknown specificity	decarboxylase, putative	-4,4	**0**	-2,3	**0,000001**	-4,7	**0**	-3,1	**0**	-2,4	**0**
	EF0635		Transport and binding proteins	Amino acids, peptides and amines	amino acid permease family protein	-4,9	**0**	-2,3	**0,0000011**	-4,8	**0**	-3,3	**0**	-2,6	**0**
	EF0636	*nhaC-2*	Transport and binding proteins	Cations and iron carrying compounds	Na+/H+ antiporter	-3,0	**0**	-1,3	0,005443	-3,4	**0**	-2,6	**0**	-1,5	**0,0000055**
	EF0637		Hypothetical proteins		hypothetical protein	2,8	**0**	0,8	0,0931197	0,3	0,3833746	0,4	0,2544406	0,0	0,9526743
	EF0638		Hypothetical proteins	Conserved	conserved hypothetical protein	2,3	**0**	0,6	0,2780409	0,5	0,105395	0,5	0,2403078	0,4	0,2464389

Operon[a]	Locus	Gene	Functional category	Subcategory	Putative function	Blood[b]	P-value[c]	YTB_5[b]	P-value[c]	YTB_15[b]	P-value[c]	YTB_30[b]	P-value[c]	YTB_60[b]	P-value[c]
	EF0639		Cellular processes	Adaptations to atypical conditions	low temperature requirement C protein, putative	-0,2	0,6365974	0,3	0,549346	0,1	0,6702838	0,3	0,3353998	0,3	0,3063357
	EF0640		Hypothetical proteins		hypothetical protein	NA	NA	NA	NA	NA	NA	NA	NA	NA	NA
	EF0641	*ldh-2*	Energy metabolism	Glycolysis/gluconeogenesis	L-lactate dehydrogenase	-1,1	0,0159377	0,1	0,7524438	-0,8	0,0123575	0,3	0,3822698	0,7	0,0390935
	EF0642		Hypothetical proteins		hypothetical protein	-0,2	0,5990444	0,3	0,5308976	-0,2	0,5876797	0,2	0,6524276	0,4	0,262568
	EF0643		Hypothetical proteins		hypothetical protein	NA	NA	-0,3	0,6481059	NA	NA	-0,2	0,4564946	0,4	0,2316854
	EF0644		Regulatory functions	DNA interactions	transcriptional regulator, LysR family	NA	NA	-0,1	0,9015756	-1,0	0,0104982	-0,8	0,0273821	0,1	0,7415372
	EF0645		Cellular processes	Pathogenesis	exfoliative toxin A, putative	NA	NA	0,0	0,9280068	-0,5	0,2374638	-0,2	0,5788298	0,2	0,6464802
	EF0646		Energy metabolism	Sugars	NAD-dependent epimerase/dehydratase family protein	NA	NA	0,4	0,4092693	-0,3	0,4116981	-0,7	0,0390373	0,1	0,7270272
	EF0647		Hypothetical proteins	Conserved	conserved hypothetical protein	0,5	0,2591212	0,2	0,6344499	-0,2	0,6196611	-0,1	0,6613727	0,4	0,1978282
	EF0648		Unknown function	Enzymes of unknown specificity	nitroreductase family protein	NA	NA	-0,3	0,6519668	-0,9	0,0039581	-0,4	0,2302177	0,4	0,2798901
	EF0650	*lplA-1*	Protein fate	Protein modification and repair	lipoate-protein ligase A	0,7	0,0814238	0,5	0,2908784	-0,5	0,1047735	-0,5	0,2419603	1,0	0,0018722
	EF0652		Hypothetical proteins		hypothetical protein	0,8	0,0680281	0,6	0,1773064	0,1	0,6705008	0,0	0,9499774	0,3	0,2984077
	EF0653		Hypothetical proteins		hypothetical protein	NA	NA	0,8	0,0710474	-0,2	0,4552545	NA	NA	0,3	0,4000785
	EF0654		Regulatory functions	DNA interactions	sugar-binding transcriptional regulator, LacI family	0,9	0,0122036	0,5	0,2355028	-0,3	0,320912	-0,4	0,3108835	0,2	0,5859906
	EF0655		Unknown function	Enzymes of unknown specificity	nitroreductase family protein, putative	1,8	0,0000853	0,5	0,2650053	-0,5	0,1501402	NA	NA	-0,1	0,7338958
	EF0656		Unknown function	Enzymes of unknown specificity	glyoxalase family protein	NA	NA	0,6	0,2161155	-1,3	0,0000549	NA	NA	0,7	0,0246682
	EF0657		Regulatory functions	DNA interactions	transcriptional regulator, DeoR family	NA	NA	0,6	0,1959035	-0,3	0,3244604	-0,1	0,8062615	0,0	0,9207787
	EF0658		Hypothetical proteins	Conserved	conserved hypothetical protein	NA	NA	0,2	0,694529	-0,5	0,1279731	-0,4	0,3018288	0,2	0,4874
	EF0659		Unknown function	General	phage SPO1 DNA polymerase-related protein, putative	0,4	0,4277836	-0,2	0,6792328	0,4	0,2000032	-0,8	0,0644646	0,4	0,2687872
	EF0660		Transport and binding proteins	Other	MATE efflux family protein	0,1	0,7403191	-0,3	0,4845108	-0,2	0,5891177	-0,4	0,275575	0,1	0,8380922
	EF0661		Protein fate	Degradation of proteins, peptides, and glycopeptides	oligoendopeptidase F, putative	1,6	**0,00001**	0,2	0,6617587	0,4	0,2484072	-0,3	0,3334229	0,0	0,8856369
	EF0662		Hypothetical proteins	Conserved	conserved hypothetical protein	NA	NA	0,2	0,6479061	-0,5	0,1209455	-0,2	0,4740492	0,2	0,4665632
	EF0663		Hypothetical proteins	Conserved	conserved hypothetical protein TIGR01033	-0,4	0,3611903	0,3	0,5664126	-0,7	0,0332151	-0,6	0,0667859	0,7	0,0342866
	EF0664		Hypothetical proteins		hypothetical protein	1,7	**0,0000023**	0,4	0,4348914	-0,1	0,8176877	-0,2	0,6326109	-0,1	0,7098281
	EF0665		Hypothetical proteins		hypothetical protein	NA	NA	-0,5	0,3649659	-0,4	0,240175	NA	NA	0,3	0,3583976
	EF0666		Unknown function	Enzymes of unknown specificity	glyoxalase family protein	0,5	0,2062089	0,6	0,162765	0,3	0,4054941	0,3	0,4020466	-0,3	0,4182325
	EF0667		Hypothetical proteins	Conserved	conserved hypothetical protein	0,6	0,0915812	0,3	0,5440098	0,2	0,5138463	0,1	0,6915109	0,0	0,9165868
	EF0668		Cell envelope	Biosynthesis/degradation of murein sacculus/peptidoglycan	UDP-N-acetylmuramoylalanyl-D-glutamate diaminopimelate ligase	-1,6	**0,0000092**	-0,6	0,223652	0,0	0,8887197	-0,2	0,4822424	0,1	0,8050808
	EF0669		Cell envelope	Biosynthesis and degradation of surface poly/liposaccharides	polysaccharide biosynthesis family protein	-1,3	0,000689	-0,5	0,3271603	-0,2	0,4571901	-0,3	0,3241811	0,2	0,4748842
	EF0670		Protein synthesis	tRNA and rRNA base modification	ribosomal small subunit pseudouridine synthase A, putative	NA	NA	0,0	0,9370047	-0,8	0,0207873	NA	NA	0,6	0,0665649
	EF0671		Protein fate	Degradation of proteins, peptides, and glycopeptides	xaa-his dipeptidase	0,2	0,6227837	0,0	0,9273354	0,0	0,9465007	-0,2	0,6865038	0,1	0,84597
	EF0672		Hypothetical proteins		hypothetical protein	NA	NA	0,5	0,2897062	-0,7	0,0301846	NA	NA	-0,4	0,1893294
	EF0673		Cell envelope	Other	membrane protein, putative	-0,1	0,8318926	0,3	0,4791632	0,6	0,0536712	0,2	0,6128149	-0,2	0,495073
	EF0674		Transport and binding proteins	Amino acids, peptides and amines	glycine betaine/carnitine/choline ABC trans, ATP-binding protein	1,4	0,0001146	1,0	0,025765	0,4	0,2433461	0,2	0,488695	0,1	0,7239783
	EF0675		Transport and binding proteins	Amino acids, peptides and amines	glycine betaine/carnitine/choline ABC trans/binding protein	1,4	0,0001072	1,0	0,0267271	0,9	0,0065201	0,5	0,1413808	-0,1	0,7710582
	EF0676	*argR*	Regulatory functions	DNA interactions	arginine repressor	0,7	0,0617667	0,6	0,1890556	0,3	0,3196084	0,1	0,7083036	0,2	0,6058416
	EF0677		Energy metabolism	Sugars	phosphoglucomutase/phosphomannomutase family protein	1,2	0,0006667	0,7	0,1081878	0,2	0,4525625	0,0	0,9684466	-0,1	0,7366395
	EF0678		Unknown function	Enzymes of unknown specificity	acetyltransferase, GNAT family	1,4	0,000205	0,8	0,0819844	0,1	0,662066	-0,1	0,8112277	0,0	0,964988
	EF0679		Protein synthesis	tRNA and rRNA base modification	ribosomal large subunit pseudouridine synthase, RluD subfamily	-1,2	0,0066091	-1,0	0,0385118	-0,4	0,1880899	-0,1	0,6717971	-0,2	0,5947496
	EF0680		Cell envelope	Biosynthesis/degradation of murein sacculus/peptidoglycan	penicillin-binding protein 2A	0,0	0,9460865	0,3	0,45381	-0,4	0,2452856	-0,3	0,3507848	0,1	0,6965794
	EF0681		Hypothetical proteins	Conserved	conserved hypothetical protein	0,8	0,0282069	0,5	0,292488	-0,1	0,704449	0,2	0,4757817	0,1	0,6695444
	EF0682		DNA metabolism	DNA replication, recombination, and repair	DNA repair exonuclease family protein	0,4	0,2737486	0,1	0,7534733	0,1	0,6526485	0,0	0,9924718	0,1	0,7639332
	EF0683		Hypothetical proteins	Conserved	conserved hypothetical protein	0,4	0,3194907	0,2	0,6761068	-0,2	0,4990205	-0,4	0,1972527	0,2	0,6100693
	EF0684		Unknown function	General	cmp-binding protein, putative	0,7	0,0598204	0,0	0,9294443	-0,2	0,5975726	-0,3	0,3169148	-0,1	0,8654863
	EF0685		Protein fate	Protein folding and stabilization	rotamase family protein	0,3	0,4384001	0,3	0,5873972	-0,1	0,8653804	0,1	0,8669978	0,0	0,9354591
	EF0686		Hypothetical proteins		hypothetical protein	0,9	0,0166278	0,7	0,1324616	0,2	0,6245536	0,1	0,7727524	0,1	0,7402848
	EF0687		Unknown function	General	HIT family protein	0,8	0,0342335	0,4	0,3442915	0,3	0,2780652	0,5	0,1586196	0,1	0,6836983
	EF0688		Transport and binding proteins	Unknown substrate	ABC transporter, ATP-binding protein	-0,3	0,3819683	0,3	0,5386337	0,2	0,4781284	0,2	0,5185767	0,2	0,4533989
	EF0689		Cell envelope	Other	membrane protein, putative	0,1	0,742441	0,1	0,8860076	0,1	0,7568216	0,3	0,35265	0,2	0,6063216
	EF0690		Hypothetical proteins	Conserved	conserved hypothetical protein	-0,4	0,2645917	-0,2	0,6470589	0,0	0,9891522	0,5	0,1377569	0,2	0,6202346
	EF0691		Unknown function	Enzymes of unknown specificity	methyltransferase, putative	-0,5	0,2042025	-0,6	0,182934	-0,1	0,6784922	0,5	0,1514945	0,0	0,9141093
	EF0692		Regulatory functions	Other	phosphosugar-binding transcriptional regulator, RpiR family, putative	NA	NA	0,8	0,0922751	-0,5	0,090815	-0,2	0,5268224	0,3	0,3136467
	EF0693	*fruK-1*	Energy metabolism	Glycolysis/gluconeogenesis	1-phosphofructokinase	0,6	0,1675285	1,2	0,0371338	NA	NA	0,3	0,3327939	0,5	0,1070627
	EF0694		Signal transduction	PTS	PTS system, fructose-specific family, IIBC components	NA	NA	1,0	0,0796415	0,1	0,8753911	-0,3	0,3599282	0,6	0,058954
	EF0695		Signal transduction	PTS	PTS system, IIA component	1,5	0,0000833	0,9	0,1032546	-0,2	0,6239265	0,2	0,6356919	0,4	0,2377997
	EF0696	*lacD-1*	Energy metabolism	Biosynthesis and degradation of polysaccharides	tagatose 1,6-diphosphate aldolase	0,5	0,1632188	0,9	0,1146432	-0,3	0,4081745	0,3	0,5177717	0,2	0,4542885
	EF0697		Hypothetical proteins	Conserved	conserved hypothetical protein	0,4	0,2229284	0,1	0,7746001	-0,4	0,2638644	0,0	0,9207948	-0,1	0,7160301
	EF0698		Unknown function	Enzymes of unknown specificity	acetyltransferase, GNAT family	0,8	0,033909	0,4	0,4184402	0,3	0,2769933	0,5	0,1571475	0,6	0,0910303
	EF0699		Hypothetical proteins	Conserved	conserved hypothetical protein	NA	NA	-0,5	0,2824153	0,4	0,2311161	0,1	0,7345204	0,2	0,6441297
	EF0700		Cellular processes	Pathogenesis	hemolysin	-0,8	0,0303433	-0,9	0,0618055	-0,1	0,836883	0,0	0,9855704	-0,1	0,8205855
	EF0701	*prfC*	Protein synthesis	Translation factors	peptide chain release factor 3	-2,4	**0**	-1,0	0,0343414	-0,2	0,4993749	0,1	0,7118328	0,0	0,9113346
	EF0702		Hypothetical proteins	Conserved	conserved hypothetical protein	NA	NA	0,2	0,6606511	-0,3	0,3860918	-0,1	0,8554986	0,1	0,7270574
	EF0703		Hypothetical proteins	Conserved	conserved hypothetical protein	0,7	0,1295024	0,4	0,3388747	-0,7	0,0348037	-0,3	0,3317199	0,1	0,6656725
	EF0704		Cell envelope	Other	lipoprotein, putative	0,0	0,9630874	0,2	0,599884	0,3	0,3125903	-0,2	0,5230425	0,5	0,1633395
	EF0705		Hypothetical proteins	Conserved	conserved hypothetical protein	NA	NA	0,3	0,5875938	NA	NA	NA	NA	0,0	0,9091569
	EF0706	*clpE*	Protein fate	Degradation of proteins, peptides, and glycopeptides	ATP-dependent Clp protease, ATP-binding subunit ClpE	1,8	**0,0000004**	0,6	0,1961418	0,0	0,9981575	-0,2	0,4686292	-0,6	0,0939591
	EF0707		Hypothetical proteins		hypothetical protein	NA	NA	NA	NA	NA	NA	NA	NA	NA	NA
	EF0708		Hypothetical proteins	Conserved	conserved hypothetical protein	NA	NA	-0,3	0,5463271	-0,2	0,6226632	-0,1	0,8457471	-0,2	0,4776751
	EF0709	*ptsH*	Signal transduction	PTS	phosphocarrier protein HPr	-2,0	**0**	-0,3	0,5022597	-0,2	0,5860251	-0,4	0,2447514	-0,1	0,8676894
	EF0710	*ptsI*	Signal transduction	PTS	phosphoenolpyruvate-protein phosphotransferase enzyme I	-2,6	**0**	-0,5	0,3280404	-0,3	0,3698857	-0,5	0,1099944	0,0	0,950684
	EF0711		Hypothetical proteins	Conserved	conserved hypothetical protein	1,7	0,0001557	0,6	0,2364155	0,7	0,0280052	0,9	0,0108589	0,3	0,417861
	EF0713		Hypothetical proteins	Conserved	conserved hypothetical protein	3,3	**0**	1,2	0,0346821	0,8	0,009104	1,3	0,0000969	0,4	0,1955824
	EF0714		Hypothetical proteins		hypothetical protein	NA	NA	-0,5	0,3979963	NA	NA	-0,3	0,3650067	0,4	0,2402085
	EF0715	*tig*	Protein fate	Protein and peptide secretion and trafficking	trigger factor	NA	NA	NA	NA	NA	NA	NA	NA	NA	NA
	EF0716		Hypothetical proteins	Conserved	conserved hypothetical protein	-1,5	0,0006684	-0,2	0,617728	0,5	0,1452892	-0,2	0,6499202	0,5	0,0987
	EF0717		Signal transduction	PTS	PTS system, fructose-specific family, IIABC components	2,8	**0**	-2,3	**0,0000009**	0,1	0,8525663	0,0	0,944916	0,5	0,1710612
	EF0718	*fruK-2*	Energy metabolism	Glycolysis/gluconeogenesis	1-phosphofructokinase	NA	NA	-2,3	0,000042	NA	NA	NA	NA	NA	NA
	EF0719		Regulatory functions	DNA interactions	transcriptional regulator, DeoR family	NA	NA	-1,8	0,0000833	0,6	0,0812538	0,4	0,1893143	0,2	0,6335372
	EF0720		Transport and binding proteins	Anions	voltage-gated chloride channel family protein	-1,6	0,0004336	-1,0	0,0242112	-0,9	0,007411	0,1	0,8687056	0,4	0,177513
	EF0721	*pcrA*	DNA metabolism	DNA replication, recombination, and repair	ATP-dependent DNA helicase PcrA	-0,4	0,2488994	-1,0	0,0384919	-0,5	0,0907234	-0,3	0,3954006	-0,4	0,1776229
	EF0722	*ligA*	DNA metabolism	DNA replication, recombination, and repair	DNA ligase, NAD-dependent	-1,2	0,0006634	-0,7	0,1504021	-0,3	0,3740543	-0,2	0,5047679	0,0	0,9878079
	EF0723		Hypothetical proteins		hypothetical protein	NA	NA	NA	NA	NA	NA	NA	NA	NA	NA
	EF0724	*gatC*	Protein synthesis	tRNA aminoacylation	glutamyl-tRNA(Gln) amidotransferase, C subunit	-1,5	0,0000425	-0,8	0,0701006	0,2	0,5047208	0,2	0,615153	0,0	0,9270377

Operon[a)]	Locus	Gene	Functional category	Subcategory	Putative function	Blood[b)]	P-value[c)]	YTB_5[b)]	P-value[c)]	YTB_15[b)]	P-value[c)]	YTB_30[b)]	P-value[c)]	YTB_60[b)]	P-value[c)]
	EF0725	*gatA*	Protein synthesis	tRNA aminoacylation	glutamyl-tRNA(Gln) amidotransferase, A subunit	-1,0	0,0092563	-0,4	0,4368496	-0,2	0,5608843	-0,1	0,6622421	-0,1	0,8050992
	EF0726	*gatB*	Protein synthesis	tRNA aminoacylation	glutamyl-tRNA(Gln) amidotransferase, B subunit	-1,1	0,0032818	-0,3	0,5715557	-0,2	0,6305732	0,0	0,8930501	0,0	0,9979719
	EF0727		Unknown function	General	diacylglycerol kinase catalytic domain protein	-0,2	0,6396085	-0,4	0,3766475	-0,1	0,7975877	-0,3	0,3970652	0,1	0,7404488
	EF0728		Protein synthesis	tRNA and rRNA base modification	RNA methyltransferase, TrmA family	-1,5	0,0000566	-0,5	0,2410809	-0,2	0,5804554	-0,2	0,5612276	0,5	0,1446246
	EF0730		Hypothetical proteins	Conserved	conserved hypothetical protein	NA	NA	0,3	0,5509666	NA	NA	0,5	0,2314328	-0,2	0,5116057
	EF0731		Regulatory functions	DNA interactions	transcriptional regulator, luxR family	0,3	0,4361967	0,2	0,6074515	0,0	0,9631537	0,0	0,9266173	-0,1	0,7439534
	EF0732	*argF-2*	Energy metabolism	Amino acids and amines	ornithine carbamoyltransferase	NA	NA	0,7	0,1133037	-0,3	0,2854221	0,2	0,5659079	0,0	0,9162069
	EF0733		Transport and binding proteins	Amino acids, peptides and amines	amino acid permease family protein	NA	NA	0,4	0,376376	NA	NA	NA	NA	0,0	0,9015369
	EF0734		Hypothetical proteins	Conserved	conserved hypothetical protein	NA	NA	1,1	0,0613854	NA	NA	NA	NA	0,0	0,9236789
	EF0735	*arcC-3*	Energy metabolism	Amino acids and amines	carbamate kinase	NA	NA	0,6	0,2622482	NA	NA	NA	NA	0,1	0,7080263
	EF0737		Unknown function	Enzymes of unknown specificity	amidase, putative	-2,1	**0**	0,0	0,9478246	-0,2	0,5922982	-0,2	0,4830915	-0,3	0,3486509
	EF0738		Hypothetical proteins		hypothetical protein	NA	NA	0,0	0,9437957	-0,6	0,1534843	NA	NA	0,1	0,7076698
	EF0739		Transport and binding proteins	Other	nicotinamide mononucleotide transporter PnuC, putative	-1,9	**0,0000001**	-1,0	0,0293752	-0,7	0,0353498	-1,0	0,0018199	-0,7	0,0251245
	EF0740		Purines, pyrimidines, nucleosides, and nucleotides	2'-Deoxyribonucleotide metabolism	deoxynucleoside kinase	-0,9	0,0453138	-1,2	0,0111502	-0,7	0,0257361	-0,6	0,0530414	-0,7	0,0318634
	EF0741		Hypothetical proteins	Domain	conserved domain protein	0,2	0,6233614	-0,4	0,3757476	-0,2	0,5834888	-0,4	0,1900144	0,1	0,6518708
	EF0742		Hypothetical proteins	Conserved	conserved hypothetical protein	-0,5	0,1975466	0,3	0,5528563	0,3	0,2897017	-0,1	0,7494499	0,3	0,3264007
	EF0743		Hypothetical proteins		hypothetical protein	1,7	**0,0000041**	0,5	0,3128575	0,3	0,3754851	0,1	0,703603	-0,1	0,7546321
	EF0744		Transport and binding proteins	Carbohydrates, organic alcohols, and acids	sodium/dicarboxylate symporter family protein	-0,3	0,419679	1,0	0,0366511	0,0	0,9648655	-0,5	0,1524388	-0,4	0,2256793
	EF0745		Unknown function	Enzymes of unknown specificity	glyoxalase family protein	1,3	0,0006025	0,7	0,1078894	0,0	0,9812767	0,2	0,4806685	0,0	0,9790418
	EF0746		Cell envelope	Biosynthesis/degradation of murein sacculus/peptidoglycan	penicillin-binding protein, putative	-1,2	0,0110184	0,5	0,2444012	-0,6	0,049979	0,1	0,8201861	-0,3	0,3001728
	EF0747		Hypothetical proteins	Conserved	conserved hypothetical protein	2,0	**0,0000001**	0,6	0,183107	-0,1	0,6786017	0,3	0,4206705	0,0	0,9135673
	EF0748		Cellular processes	Detoxification	rhodanese family protein	0,9	0,0111856	-0,1	0,7555188	1,3	0,000028	1,0	0,0037972	0,4	0,214225
	EF0750		Cell envelope	Other	cell wall surface anchor family protein	1,2	0,0012963	0,6	0,2974609	-0,7	0,0271209	NA	NA	-0,2	0,4688243
	EF0751		Hypothetical proteins	Conserved	conserved hypothetical protein	NA	NA	NA	NA	-0,2	0,6244415	NA	NA	-0,4	0,2147839
	EF0752		Hypothetical proteins	Conserved	conserved hypothetical protein	NA	NA	1,1	0,0211328	-0,5	0,0983886	NA	NA	0,1	0,7807966
	EF0753		Hypothetical proteins		hypothetical protein	1,7	**0,0000051**	0,7	0,1542968	-0,4	0,361889	0,4	0,2568077	-0,2	0,5184802
	EF0754		Hypothetical proteins	Conserved	conserved hypothetical protein	NA	NA	0,8	0,1441288	NA	NA	NA	NA	0,2	0,5760223
	EF0755		Hypothetical proteins	Conserved	conserved hypothetical protein	NA	NA	1,3	0,0210808	NA	NA	NA	NA	0,2	0,5901357
	EF0756		Hypothetical proteins		hypothetical protein	0,9	0,0199106	0,6	0,1942999	-0,6	0,0775893	NA	NA	-0,4	0,2167486
	EF0757		Hypothetical proteins		hypothetical protein	NA	NA	NA	NA	NA	NA	NA	NA	-0,4	0,2002846
	EF0758		Transport and binding proteins	Cations and iron carrying compounds	cadmium-translocating P-type ATPase	NA	NA	NA	NA	NA	NA	NA	NA	NA	NA
	EF0759		Unknown function	General	sapB protein, putative	1,4	0,0021656	0,6	0,201109	-0,5	0,1000735	-0,2	0,6356678	0,3	0,35079
	EF0760		Transport and binding proteins	Amino acids, peptides and amines	amino acid ABC transporter, ATP-binding protein	-3,7	**0**	-0,9	0,0486302	-0,8	0,007028	-1,2	0,0005577	0,1	0,8229551
	EF0761		Transport and binding proteins	Amino acids, peptides and amines	amino acid ABC transporter, amino acid-binding/permease protein	-4,1	**0**	-1,4	0,0017254	-0,9	0,0053841	-1,0	0,0036689	-0,3	0,4372114
	EF0762	*uvrB*	DNA metabolism	DNA replication, recombination, and repair	excinuclease ABC, subunit B	0,6	0,0942154	0,0	0,9922609	-0,1	0,786051	-0,1	0,8462664	-0,2	0,4944055
	EF0763	*uvrA*	DNA metabolism	DNA replication, recombination, and repair	excinuclease ABC, subunit A	-0,2	0,6087361	0,2	0,72548	0,0	0,8803131	-0,4	0,2666567	-0,1	0,8352466
	EF0764		Hypothetical proteins		hypothetical protein	NA	NA	1,4	0,0131059	0,2	0,5406842	0,6	0,081041	1,1	0,0007913
	EF0765		Hypothetical proteins		hypothetical protein	NA	NA	NA	NA	NA	NA	NA	NA	NA	NA
	EF0766		Hypothetical proteins	Conserved	conserved hypothetical protein	1,1	0,0023683	0,7	0,1189388	0,0	0,9192719	0,1	0,8636218	0,0	0,9464005
	EF0767		Hypothetical proteins	Conserved	conserved hypothetical protein	0,8	0,031857	0,6	0,1910431	0,1	0,8297906	0,1	0,7473837	-0,3	0,4365217
	EF0768		Hypothetical proteins	Conserved	conserved hypothetical protein	1,2	0,0014664	0,6	0,2292452	0,0	0,9083608	0,1	0,6772181	0,0	0,9421367
	EF0769		Hypothetical proteins	Conserved	conserved hypothetical protein	1,3	0,000419	0,3	0,4896156	-0,3	0,3530168	0,2	0,5215741	0,1	0,7185891
	EF0770		Hypothetical proteins	Conserved	conserved hypothetical protein	1,6	0,0003828	0,8	0,0994926	0,5	0,1498391	0,6	0,0754145	0,4	0,230127
	EF0771	*clpP*	Protein fate	Degradation of proteins, peptides, and glycopeptides	ATP-dependent Clp protease, proteolytic subunit ClpP	1,1	0,0024122	0,4	0,4024563	0,0	0,9797692	0,1	0,7120747	-0,4	0,2521082
	EF0773		Hypothetical proteins	Conserved	conserved hypothetical protein	NA	NA	NA	NA	NA	NA	NA	NA	NA	NA
	EF0774		Hypothetical proteins	Conserved	conserved hypothetical protein	0,8	0,0319293	0,2	0,7252434	-0,1	0,6974846	0,3	0,4552577	0,4	0,2251993
	EF0775		Cell envelope	Other	gram positive anchor protein, putative	NA	NA	0,5	0,405337	NA	NA	0,0	0,989806	-0,1	0,7724604
	EF0776		Hypothetical proteins		hypothetical protein	NA	NA	0,4	0,4714541	NA	NA	0,0	0,9832763	0,3	0,4615473
	EF0778		Hypothetical proteins		hypothetical protein	NA	NA	NA	NA	NA	NA	NA	NA	NA	NA
	EF0779		Fatty acid and phospholipid metabolism	Degradation	glycerophosphoryl diester phosphodiesterase family protein	-1,0	0,0059542	0,1	0,8454137	-0,7	0,0190357	-0,5	0,1409465	0,0	0,958773
	EF0780		DNA metabolism	DNA replication, recombination, and repair	MutT/nudix family protein	0,3	0,4563619	-0,2	0,6025905	-0,7	0,0192179	-0,3	0,4239466	0,0	0,8885863
	EF0781		Cellular processes	Adaptations to atypical conditions	cold shock domain family protein	0,5	0,2306973	0,2	0,6119586	0,1	0,6497217	1,3	0,000079	1,0	0,0017949
	EF0782	*rpoN*	Transcription	Transcription factors	RNA polymerase sigma-54 factor	-1,0	0,0315011	0,1	0,9035499	-0,5	0,0866258	0,2	0,5513575	-0,1	0,8288077
	EF0783		Unknown function	Enzymes of unknown specificity	acyltransferase, putative	-1,1	0,0028365	-0,2	0,6306831	-0,4	0,2166206	-0,4	0,1979479	-0,4	0,2003507
	EF0784	*metK*	Central intermediary metabolism	Other	S-adenosylmethionine synthetase	-0,7	0,0740387	-0,1	0,8274058	0,1	0,8438065	0,7	0,0323419	-0,1	0,8611588
	EF0785		Transport and binding proteins	Other	drug resistance transporter, EmrB/QacA family protein	-0,1	0,7723199	-0,3	0,5535103	-0,1	0,8017796	-0,1	0,8103847	-0,2	0,5103733
	EF0786		Fatty acid and phospholipid metabolism	Degradation	tributyrin esterase, putative	-0,4	0,2421329	0,0	0,9382545	0,0	0,9962878	0,0	0,8958901	-0,1	0,8802609
	EF0787		Regulatory functions	DNA interactions	transcriptional regulator, TetR family	0,4	0,3338739	0,3	0,4989392	0,0	0,9036531	0,3	0,3191434	-0,8	0,0136174
	EF0789		Transport and binding proteins	Unknown substrate	ABC transporter, ATP-binding/permease protein	-0,9	0,017609	-0,5	0,2943772	-0,1	0,794044	-0,8	0,0137726	-0,3	0,383549
	EF0790		Transport and binding proteins	Unknown substrate	ABC transporter, ATP-binding/permease protein	-1,4	0,0000823	-0,3	0,4511276	0,2	0,5566625	-0,9	0,0059277	0,0	0,8834197
	EF0791		Regulatory functions	DNA interactions	transcriptional regulator, TetR family	NA	NA	NA	NA	NA	NA	NA	NA	NA	NA
	EF0792		Transport and binding proteins	Unknown substrate	permease domain protein	NA	NA	0,5	0,3560872	1,3	0,000072	0,9	0,0100244	0,5	0,1549495
	EF0793	*hrtB*	Transport and binding proteins	Unknown substrate	ABC transporter, ATP-binding protein	1,0	0,0304555	-0,1	0,8950998	2,0	**0**	1,0	0,002195	0,2	0,4660459
	EF0794		Hypothetical proteins	Conserved	conserved hypothetical protein	-2,1	**0**	-0,5	0,2765149	-0,1	0,7585947	-0,1	0,7361515	-0,3	0,369463
	EF0795		Hypothetical proteins	Conserved	conserved hypothetical protein TIGR01212	-2,7	**0**	-0,6	0,1834588	-0,2	0,6280841	-0,3	0,3010705	-0,1	0,7694261
	EF0796		Unknown function	Enzymes of unknown specificity	type 2 phosphatidic acid phosphatase family protein	-2,6	**0**	-0,8	0,0894664	0,1	0,7674611	-0,2	0,5653463	0,0	0,912459
	EF0797		Hypothetical proteins	Domain	conserved domain protein	0,4	0,3761108	2,0	0,0000226	0,1	0,8389056	0,4	0,2361113	0,1	0,7484223
	EF0798		Hypothetical proteins		hypothetical protein	0,2	0,5305821	2,2	**0,000002**	-0,1	0,7669829	0,1	0,6635165	0,5	0,1408709
	EF0799	*atlA*	Cell envelope	Biosynthesis and degradation of surface poly/liposaccharides	autolysin	0,1	0,8651504	-0,3	0,5670513	0,3	0,2937388	-0,5	0,1263554	-0,4	0,2808919
	EF0801	*leuS*	Protein synthesis	tRNA aminoacylation	leucyl-tRNA synthetase	0,0	0,9498485	-0,2	0,7113204	0,0	0,8955766	-0,4	0,2709084	0,0	0,9304496
	EF0802		Hypothetical proteins		hypothetical protein	NA	NA	NA	NA	NA	NA	NA	NA	NA	NA
	EF0803		Hypothetical proteins	Conserved	conserved hypothetical protein	0,1	0,7948351	-0,3	0,4935628	-0,1	0,6544922	-0,3	0,4018918	-0,1	0,8327311
	EF0804		Transport and binding proteins	Amino acids, peptides and amines	amino acid ABC transporter, amino acid-binding protein	0,7	0,1327975	-0,1	0,832313	-0,4	0,2454786	NA	NA	0,0	0,9119043
	EF0805		Transport and binding proteins	Amino acids, peptides and amines	amino acid ABC transporter, ATP-binding protein	-0,3	0,5703179	-0,1	0,815896	NA	NA	-0,4	0,2976955	0,2	0,6052189
	EF0806		Transport and binding proteins	Amino acids, peptides and amines	amino acid ABC transporter, permease protein	-0,5	0,2198774	0,1	0,787377	-0,8	0,0384151	-0,2	0,5791387	0,2	0,4799227
	EF0807		Transport and binding proteins	Amino acids, peptides and amines	pheromone binding protein, putative	1,6	0,0000208	1,0	0,0254113	0,5	0,1149349	0,7	0,0439349	0,4	0,2774815
	EF0808		Hypothetical proteins		hypothetical protein	NA	NA	NA	NA	NA	NA	NA	NA	-0,5	0,2454197
	EF0809		Cell envelope	Other	membrane protein, putative	3,2	**0**	0,8	0,0841077	1,2	0,0001534	1,2	0,0005769	0,1	0,6820719
	EF0810		Hypothetical proteins	Conserved	conserved hypothetical protein	0,9	0,0218141	0,0	0,9421032	-0,8	0,0453496	-0,3	0,452117	0,4	0,1884278
	EF0811		Hypothetical proteins		hypothetical protein	NA	NA	0,3	0,5710275	NA	NA	NA	NA	0,1	0,719662
	EF0812		Energy metabolism	Biosynthesis and degradation of polysaccharides	glucuronyl hydrolase, putative	NA	NA	0,3	0,5738079	NA	NA	-0,1	0,6679047	0,0	0,9937169
	EF0813		Energy metabolism	Biosynthesis and degradation of polysaccharides	glycosyl hydrolase, family 35	0,1	0,7461018	0,0	0,9750015	-0,1	0,8687266	-0,4	0,2179877	0,0	0,9547883
	EF0814		Regulatory functions	DNA interactions	transcriptional regulator, GntR family	-0,5	0,2824842	-0,2	0,6145856	0,3	0,4058866	-0,3	0,4548239	0,0	0,9129709

Operon[a]	Locus	Gene	Functional category	Subcategory	Putative function	Blood[b]	P-value[c]	YTB_5[b]	P-value[c]	YTB_15[b]	P-value[c]	YTB_30[b]	P-value[c]	YTB_60[b]	P-value[c]
	EF0815		Signal transduction	PTS	PTS system, IIAB components	0,7	0,1280527	0,8	0,0900152	-0,5	0,1079925	-0,1	0,8190844	-0,1	0,6792788
	EF0816		Signal transduction	PTS	PTS system, IIC component	0,5	0,2153179	0,1	0,8613622	-0,5	0,1922915	-0,3	0,3539631	-0,1	0,7148705
	EF0817		Signal transduction	PTS	PTS system, IID component	0,5	0,1975302	0,6	0,1901548	-0,6	0,0468825	-0,6	0,0763655	-0,4	0,2360428
	EF0818		Cellular processes	Pathogenesis	polysaccharide lyase, family 8	NA	NA	0,4	0,3501133	0,2	0,5300991	0,0	0,9316837	-0,2	0,5077991
	EF0819		Hypothetical proteins	Conserved	conserved hypothetical protein	0,8	0,0272026	0,0	0,9683862	0,7	0,0386149	0,3	0,3308287	-0,1	0,828677
	EF0820	*rplY*	Protein synthesis	Ribosomal proteins: synthesis and modification	ribosomal protein L25	2,3	**0**	1,6	0,0004562	-0,1	0,7876685	0,6	0,0837645	-0,3	0,3357243
	EF0821		Protein synthesis	tRNA and rRNA base modification	ribosomal small subunit pseudouridine synthase A, putative	-1,4	0,002352	-1,0	0,0346594	NA	NA	NA	NA	-0,2	0,5255342
	EF0822		Unknown function	Enzymes of unknown specificity	hydrolase, haloacid dehalogenase-like family	-1,2	0,0011969	-0,1	0,7603448	0,1	0,7019982	0,0	0,9304203	-0,2	0,5739639
	EF0823		Hypothetical proteins		hypothetical protein	NA	NA	NA	NA	NA	NA	NA	NA	NA	NA
	EF0824		Unknown function	Enzymes of unknown specificity	acetyltransferase, GNAT family	NA	NA	-0,3	0,546434	0,3	0,3597784	0,0	0,9750144	-0,1	0,8140979
	EF0825	*udk*	Purines, pyrimidines, nucleosides, and nucleotides	Salvage of nucleosides and nucleotides	uridine kinase	-1,5	0,0008692	-1,0	0,0361434	0,2	0,4587721	0,3	0,3727653	-0,3	0,3651932
	EF0826		Regulatory functions	DNA interactions	transcriptional regulator, PemK family	NA	NA	NA	NA	NA	NA	NA	NA	0,4	0,2762652
	EF0827		Unknown function	Enzymes of unknown specificity	oxidoreductase, Gfo/Idh/MocA family	0,5	0,1665693	-0,2	0,7366432	0,2	0,4963674	0,1	0,8110476	-0,1	0,8376601
	EF0828		Hypothetical proteins	Conserved	conserved hypothetical protein	NA	NA	NA	NA	NA	NA	NA	NA	NA	NA
	EF0829		Hypothetical proteins	Conserved	conserved hypothetical protein	NA	NA	0,8	0,1568166	NA	NA	0,6	0,0674344	-0,2	0,5822966
	EF0830		Hypothetical proteins	Conserved	conserved hypothetical protein	NA	NA	0,5	0,2706107	-0,1	0,7608827	0,1	0,7818921	0,0	0,9596758
	EF0831		Hypothetical proteins	Conserved	conserved hypothetical protein	NA	NA	NA	NA	NA	NA	0,7	0,0300905	-0,3	0,3915238
	EF0832		Hypothetical proteins	Conserved	conserved hypothetical protein	1,1	0,0180363	0,7	0,1458978	0,3	0,3000826	0,2	0,5683933	-0,1	0,8575382
	EF0833		Hypothetical proteins	Conserved	conserved hypothetical protein	1,8	**0,0000017**	0,7	0,1198348	0,0	0,8769999	0,2	0,6042491	-0,2	0,5272594
	EF0834		Signal transduction	PTS	PTS system, IIC component	0,9	0,0520699	-0,1	0,8699265	-0,1	0,8178856	-0,2	0,4994417	0,0	0,9622692
	EF0835		Hypothetical proteins		hypothetical protein	NA	NA	NA	NA	NA	NA	NA	NA	NA	NA
	EF0836		Hypothetical proteins	Conserved	conserved hypothetical protein	NA	NA	0,2	0,6133784	-0,3	0,3120061	-0,3	0,3040903	-0,2	0,4531948
	EF0837		Hypothetical proteins	Conserved	conserved hypothetical protein	NA	NA	-0,1	0,7878113	0,4	0,2862025	0,1	0,7717369	-0,1	0,7021775
	EF0838		Unknown function	Enzymes of unknown specificity	pyridoxal phosphate-dependent enzyme, putative	0,8	0,0397201	0,1	0,8502664	0,1	0,8157996	-0,2	0,4909824	-0,3	0,3190536
	EF0839		Hypothetical proteins	Conserved	conserved hypothetical protein	1,0	0,0054508	0,3	0,5574373	0,2	0,4492983	0,0	0,9858296	-0,4	0,1963993
	EF0840		Energy metabolism	Sugars	carbohydrate kinase, pfkB family	1,0	0,0099592	0,2	0,6455222	-0,3	0,4767021	0,0	0,89825	-0,3	0,3515575
	EF0841		Hypothetical proteins	Domain	conserved domain protein	1,0	0,0044054	0,8	0,0898326	0,1	0,7989362	-0,1	0,7666588	-0,1	0,6915357
	EF0842		Unknown function	General	YaiI/YqxD family protein	0,6	0,115524	0,8	0,083584	0,3	0,3902667	0,1	0,7183542	0,2	0,55106
	EF0843		Cell envelope	Biosynthesis/degradation of murein sacculus/peptidoglycan	D-alanine--D-alanine ligase	-0,4	0,2841587	-0,1	0,7760952	0,1	0,7265576	0,2	0,4672908	0,1	0,8040612
	EF0844		Hypothetical proteins		hypothetical protein	NA	NA	NA	NA	NA	NA	NA	NA	NA	NA
	EF0845	*murF*	Cell envelope	Biosynthesis/degradation of murein sacculus/peptidoglycan	UDP-N-acetylmuramoylalanyl-D-glutamyl-diaminopimelate- ligase	-0,8	0,0326932	-0,3	0,5374061	-0,3	0,4145376	-0,2	0,557973	0,0	0,8822682
	EF0846		Transcription	Other	ATP-dependent RNA helicase, DEAD/DEAH box family	-1,1	0,0027807	-0,9	0,0539908	-0,3	0,2699318	-0,3	0,3863954	0,1	0,7073525
	EF0847		Hypothetical proteins		hypothetical protein	NA	NA	NA	NA	NA	NA	NA	NA	NA	NA
	EF0848	*acpS*	Fatty acid and phospholipid metabolism	Biosynthesis	holo-(acyl-carrier-protein) synthase	-0,4	0,3001956	-0,4	0,4334976	0,1	0,6783945	0,0	0,9027407	-0,2	0,4975285
	EF0849	*alr*	Cell envelope	Biosynthesis/degradation of murein sacculus/peptidoglycan	alanine racemase	-0,6	0,0832009	-0,6	0,1791938	0,1	0,7747778	-0,1	0,8034691	-0,2	0,6491806
	EF0850		Regulatory functions	DNA interactions	transcriptional regulator, PemK family	-0,8	0,0243103	-0,6	0,1937655	0,1	0,682301	0,0	0,9355596	-0,2	0,5671523
	EF0851		Hypothetical proteins		hypothetical protein	NA	NA	0,3	0,5593152	NA	NA	-0,4	0,2611589	0,1	0,6992628
	EF0852		Hypothetical proteins		hypothetical protein	1,1	0,0032543	0,8	0,1840465	-0,4	0,2126434	NA	NA	0,2	0,470539
	EF0853		Hypothetical proteins		hypothetical protein	1,3	0,0009991	0,2	0,7025476	0,1	0,6887807	0,1	0,7146789	-0,3	0,3261911
	EF0854		Protein fate	Protein and peptide secretion and trafficking	signal peptidase I	NA	NA	1,2	0,0381369	NA	NA	0,2	0,5627466	0,1	0,7877946
	EF0855		Hypothetical proteins	Domain	conserved domain protein	NA	NA	NA	NA	NA	NA	NA	NA	NA	NA
	EF0856		Unknown function	General	aldo/keto reductase family protein	0,2	0,6399939	0,5	0,2872371	-0,5	0,109929	-0,7	0,0509287	0,3	0,3058795
	EF0857		Hypothetical proteins	Conserved	conserved hypothetical protein	-0,9	0,0253283	-0,2	0,6778607	-0,6	0,0599814	-0,4	0,2611143	0,0	0,9955291
	EF0858	*pip*	Unknown function	General	phage infection protein	-1,7	**0,0000028**	-1,9	0,0000245	-1,5	**0,0000016**	-1,7	**0,0000004**	-0,7	0,049081
	EF0859		Transport and binding proteins	Cations and iron carrying compounds	cation efflux family protein	-1,2	0,0065261	-0,9	0,0614347	0,2	0,5425525	0,3	0,3534922	0,0	0,9361135
	EF0860		Cell envelope	Other	membrane protein, putative	-1,6	0,0000194	-0,9	0,0524468	-0,8	0,0133816	-0,7	0,0378494	-0,5	0,1017144
	EF0861		Unknown function	Enzymes of unknown specificity	acetyltransferase, GNAT family	0,1	0,8306431	0,5	0,2841759	-0,6	0,1216059	-0,5	0,1130413	0,4	0,1871228
	EF0862		Transport and binding proteins	Amino acids, peptides and amines	glycine betaine/carnitine/choline ABC transporter, permease protein	1,3	0,0005196	0,4	0,4154405	0,2	0,5484408	0,3	0,4212295	0,3	0,3850628
	EF0863		Transport and binding proteins	Amino acids, peptides and amines	glycine betaine/carnitine/choline ABC transporter/binding protein	1,2	0,000783	0,4	0,4173141	0,2	0,5398454	-0,1	0,8786147	0,4	0,2840245
	EF0864		Transport and binding proteins	Amino acids, peptides and amines	glycine betaine/carnitine/choline ABC transporter, permease protein	1,1	0,0033249	0,5	0,3010532	0,2	0,5389406	-0,1	0,8563056	0,1	0,7940659
	EF0865		Transport and binding proteins	Amino acids, peptides and amines	glycine betaine/carnitine/choline transporter, ATP-binding protein	1,2	0,0015982	0,4	0,4143996	0,2	0,4384011	0,0	0,9729604	0,4	0,2271211
	EF0867		Unknown function	Enzymes of unknown specificity	glyoxalase family protein	0,5	0,2564228	0,4	0,4219596	0,0	0,9899785	0,4	0,2792972	0,1	0,7531866
	EF0868	*queA*	Protein synthesis	tRNA and rRNA base modification	S-adenosylmethionine:tRNA ribosyltransferase-isomerase	0,1	0,8831345	-0,7	0,1334774	0,1	0,7993834	0,0	0,9400315	-0,1	0,725592
	EF0869		Regulatory functions	DNA interactions	transcriptional regulator, Cro/CI family	0,6	0,1730307	-0,2	0,6520288	-0,1	0,7661571	0,4	0,2554419	-0,1	0,7987698
	EF0871		Transport and binding proteins	Cations and iron carrying compounds	cation-transporting ATPase, E1-E2 family	0,8	0,0592354	-0,3	0,5541755	-1,0	0,0022142	-0,9	0,0095835	0,7	0,0330436
	EF0872		Transport and binding proteins	Cations and iron carrying compounds	potassium uptake protein	-2,5	**0**	0,4	0,4126747	0,8	0,01278	0,6	0,0903385	-0,1	0,8444803
	EF0873		Regulatory functions	DNA interactions	transcriptional regulator, Cro/CI family	NA	NA	NA	NA	NA	NA	NA	NA	NA	NA
	EF0875		Cellular processes	Detoxification	copper-translocating P-type ATPase	1,7	**0,0000072**	0,3	0,4767405	0,3	0,3674793	0,5	0,1240383	-0,3	0,435147
	EF0876		Hypothetical proteins		hypothetical protein	1,1	0,0030153	0,5	0,3593064	-0,6	0,1425216	0,1	0,7482664	-0,2	0,5205051
	EF0877		Unknown function	Enzymes of unknown specificity	oxidoreductase, aldo/keto reductase 2 family	1,1	0,0033664	1,0	0,0310484	0,0	0,8955665	0,3	0,341906	0,1	0,6637448
	EF0878	*polA*	DNA metabolism	DNA replication, recombination, and repair	DNA polymerase I	NA	NA	0,4	0,3419327	0,0	0,9576331	0,1	0,6889336	0,1	0,7114154
	EF0879	*fpg*	DNA metabolism	DNA replication, recombination, and repair	formamidopyrimidine-DNA glycosylase	NA	NA	-0,5	0,3116167	NA	NA	NA	NA	NA	NA
	EF0880		Biosynthesis of cofactors, prosthetic groups, carriers	Pantothenate and coenzyme A	dephospho-CoA kinase, putative	0,0	0,9753011	0,3	0,5668231	0,3	0,3512509	0,2	0,5602255	0,2	0,4989202
	EF0881		Hypothetical proteins	Conserved	conserved hypothetical protein TIGR00244	0,6	0,1104028	-0,1	0,905111	0,3	0,325561	0,2	0,505615	0,2	0,6229799
	EF0882		DNA metabolism	DNA replication, recombination, and repair	replication initiation and membrane attachment protein DnaB, putativ	1,4	0,0001359	0,5	0,2463492	0,1	0,6350932	0,3	0,3170148	0,3	0,32041
	EF0883	*dnaI*	DNA metabolism	DNA replication, recombination, and repair	primosomal protein DnaI	1,0	0,0044838	0,6	0,1644915	0,2	0,466153	0,4	0,182794	0,1	0,6685379
	EF0884		Hypothetical proteins	Conserved	conserved hypothetical protein	NA	NA	0,4	0,3573747	0,0	0,9642207	0,4	0,2226412	-0,1	0,7772641
	EF0885		Hypothetical proteins	Conserved	conserved hypothetical protein	1,2	0,0022771	0,1	0,7822331	-0,2	0,5898084	-0,6	0,083386	-0,3	0,3111667
	EF0886		Hypothetical proteins		hypothetical protein	0,2	0,6059096	0,0	0,9630749	0,2	0,5094247	0,1	0,7072739	0,2	0,5634033
	EF0887		Cell envelope	Biosynthesis and degradation of surface poly/liposaccharides	glycosyl transferase, group 2 family protein	0,3	0,3696165	-0,2	0,6308565	0,0	0,8759509	0,1	0,6960308	0,2	0,5638247
	EF0888		Hypothetical proteins	Conserved	conserved hypothetical protein	1,1	0,0042347	-0,2	0,6726062	-0,1	0,6581821	0,5	0,1546502	0,0	0,9872371
	EF0889		Hypothetical proteins	Conserved	conserved hypothetical protein	NA	NA	NA	NA	NA	NA	NA	NA	NA	NA
	EF0890		Hypothetical proteins		hypothetical protein	NA	NA	NA	NA	0,8	0,0406504	NA	NA	-0,2	0,5932935
	EF0891		Amino acid biosynthesis	Aspartate family	aspartate aminotransferase, putative	1,0	0,0066004	0,3	0,4951318	0,1	0,7253984	-0,5	0,1679337	0,4	0,2612135
	EF0892		Transport and binding proteins	Amino acids, peptides and amines	amino acid ABC transporter, ATP-binding protein	-0,6	0,1936171	0,3	0,4737055	0,0	0,9607104	-0,2	0,5886314	1,0	0,002567
	EF0893		Transport and binding proteins	Amino acids, peptides and amines	amino acid ABC transporter, amino acid-binding/permease protein	-0,6	0,1125718	0,5	0,2741038	-0,1	0,7778797	0,2	0,5864032	1,0	0,0023763
	EF0895		Central intermediary metabolism	Other	glycerol dehydrogenase, putative	NA	NA	NA	NA	NA	NA	NA	NA	NA	NA
	EF0896	*tgt*	Protein synthesis	tRNA and rRNA base modification	queuine tRNA-ribosyltransferase	-2,0	**0,0000048**	-1,0	0,0352061	-0,1	0,8694646	0,0	0,9270213	0,1	0,8702904
	EF0897		Protein fate	Protein and peptide secretion and trafficking	preprotein translocase, YajC subunit, putative	NA	NA	-0,5	0,2597142	0,0	0,9050343	0,2	0,4586289	0,0	0,9117603
	EF0898		Hypothetical proteins		hypothetical protein	NA	NA	-0,3	0,5025485	NA	NA	0,1	0,7475349	0,0	0,9522542
	EF0899		Hypothetical proteins	Conserved	conserved hypothetical protein	-0,6	0,2084933	-0,7	0,1269864	-0,2	0,5946399	0,2	0,6085229	0,0	0,8833382
	EF0900	*adhE*	Energy metabolism	Fermentation	aldehyde-alcohol dehydrogenase	-3,0	**0**	-1,7	0,0002464	-1,1	0,0004059	0,6	0,0938969	0,5	0,1727861
	EF0901		Biosynthesis of cofactors, prosthetic groups, carriers	Other	isopentenyl diphosphate delta isomerase, putative	NA	NA	0,0	0,9921814	NA	NA	-0,9	0,0118386	0,2	0,6425162

Operon[a)]	Locus	Gene	Functional category	Subcategory	Putative function	Blood[b)]	P-value[c)]	YTB_5[b)]	P-value[c)]	YTB_15[b)]	P-value[c)]	YTB_30[b)]	P-value[c)]	YTB_60[b)]	P-value[c)]
	EF0902		Central intermediary metabolism	Other	phosphomevalonate kinase	0,7	0,0556329	-0,9	0,0429109	0,1	0,7086755	-1,0	0,0041692	-0,4	0,1879514
	EF0903	*mvaD*	Central intermediary metabolism	Other	mevalonate diphosphate decarboxylase	0,1	0,7919597	-0,9	0,0562294	-0,2	0,5992157	-0,9	0,0075586	-0,3	0,4363637
	EF0904	*mvk*	Central intermediary metabolism	Other	mevalonate kinase	-1,3	0,0030751	-0,7	0,1165645	0,5	0,1096936	0,2	0,5775079	-0,1	0,7855813
	EF0905		Unknown function	General	pentapeptide repeat family protein	1,6	0,0000194	-0,2	0,5980752	-0,2	0,607518	-0,7	0,0508508	0,0	0,9921211
	EF0906		Hypothetical proteins	Conserved	conserved hypothetical protein	1,0	0,0312286	0,3	0,632178	-0,3	0,4485708	0,0	0,9544048	0,1	0,7716517
	EF0907		Transport and binding proteins	Amino acids, peptides and amines	peptide ABC transporter, peptide-binding protein	1,9	**0,0000011**	0,1	0,7931406	0,9	0,0048298	-0,8	0,0215619	1,1	0,000712
	EF0908		Hypothetical proteins	Conserved	conserved hypothetical protein	-0,9	0,0202752	-0,3	0,5364416	0,2	0,6064034	-0,7	0,042019	0,4	0,2388413
	EF0909		Transport and binding proteins	Amino acids, peptides and amines	peptide ABC transporter, permease protein	-1,0	0,0094103	-0,4	0,3402459	0,4	0,163841	-0,9	0,0080657	0,3	0,3800041
	EF0910		Transport and binding proteins	Amino acids, peptides and amines	peptide ABC transporter, permease protein	-0,6	0,0877603	-0,3	0,5763358	0,2	0,5027983	-0,7	0,0286745	0,2	0,5341928
	EF0911		Transport and binding proteins	Amino acids, peptides and amines	peptide ABC transporter, ATP-binding protein	-0,3	0,3567004	-0,3	0,4494516	0,0	0,8966604	-1,0	0,0023285	0,4	0,234744
	EF0912		Transport and binding proteins	Amino acids, peptides and amines	peptide ABC transporter, ATP-binding protein	-0,1	0,8484078	-0,1	0,8701719	-0,1	0,873433	-0,9	0,0099808	0,3	0,3332864
	EF0913		Mobile and extrachromosomal element functions	Transposon functions	transposase, putative	1,8	**0,0000011**	0,2	0,7281126	0,2	0,5496465	-0,5	0,1614805	0,3	0,3869901
	EF0914	*infC*	Protein synthesis	Translation factors	translation initiation factor IF-3	-3,1	**0**	-1,2	0,0078519	-0,6	0,0755562	-0,5	0,1105854	-0,1	0,6966246
	EF0915	*rpmI*	Protein synthesis	Ribosomal proteins: synthesis and modification	ribosomal protein L35	-3,1	**0**	-1,3	0,0061167	-0,6	0,0501588	-0,9	0,007005	0,1	0,7336886
	EF0916	*rplT*	Protein synthesis	Ribosomal proteins: synthesis and modification	ribosomal protein L20	-3,1	**0**	-1,3	0,0058453	-0,6	0,0606357	-0,9	0,009865	-0,2	0,6296072
	EF0917		Hypothetical proteins	Conserved	conserved hypothetical protein	1,3	0,0035207	-0,2	0,7400628	0,4	0,2693253	0,2	0,5972413	0,2	0,5918263
	EF0918		Cell envelope	Other	membrane protein, putative	0,8	0,0358972	-0,1	0,9037775	0,8	0,0079084	0,6	0,0918869	-0,4	0,2508326
	EF0919		Unknown function	Enzymes of unknown specificity	acetyltransferase, GNAT family	0,7	0,1447281	0,4	0,4444308	0,3	0,3373434	0,0	0,9729831	-0,3	0,3462956
	EF0920		Hypothetical proteins	Conserved	conserved hypothetical protein	NA	NA	0,4	0,3452925	-0,2	0,6417503	-0,3	0,3667732	0,2	0,4851422
	EF0921		Transport and binding proteins	Anions	sulfate transporter family protein	-0,5	0,3193057	-0,6	0,2163549	-0,1	0,8180515	0,3	0,4075622	0,5	0,1414652
	EF0922		Cell envelope	Other	membrane protein, putative	NA	NA	NA	NA	NA	NA	NA	NA	0,2	0,6474935
	EF0923		Regulatory functions	DNA interactions	transcriptional regulator, LysR family	0,4	0,3430475	0,2	0,6049063	0,3	0,2911725	0,1	0,6829694	0,0	0,91568
	EF0924		Hypothetical proteins	Conserved	conserved hypothetical protein	2,0	**0**	0,9	0,0517718	0,0	0,8795188	0,2	0,6436629	-0,1	0,7170994
	EF0925		Hypothetical proteins		hypothetical protein	1,8	**0,0000022**	-1,9	0,0009004	0,9	0,0164278	-0,4	0,2567362	-0,8	0,0135849
	EF0926		Signal transduction	Two-component systems	DNA-binding response regulator	-0,1	0,8038582	0,1	0,8307375	0,2	0,5104119	-0,1	0,8245294	0,1	0,7870637
	EF0927		Signal transduction	Two-component systems	sensor histidine kinase	NA	NA	-0,1	0,8043364	0,3	0,2825847	0,1	0,6696305	0,1	0,6919774
	EF0928		Transport and binding proteins	Carbohydrates, organic alcohols, and acids	glucose uptake protein	NA	NA	NA	NA	NA	NA	NA	NA	NA	NA
	EF0929		Transport and binding proteins	Amino acids, peptides and amines	amino acid permease family protein	-0,9	0,0474179	-0,8	0,1000356	-0,4	0,2005922	-0,5	0,1726241	-0,2	0,5483763
	EF0930	*metG*	Protein synthesis	tRNA aminoacylation	methionyl-tRNA synthetase	-1,2	0,0009134	-0,6	0,1606245	0,2	0,6322558	0,3	0,4520891	0,2	0,4874203
	EF0931		Hypothetical proteins		hypothetical protein	NA	NA	0,4	0,3903115	NA	NA	0,3	0,4564896	0,1	0,836544
	EF0932		Hypothetical proteins		hypothetical protein	NA	NA	0,8	0,0743805	-0,2	0,610948	0,1	0,6888318	0,4	0,2403479
	EF0933		Hypothetical proteins	Conserved	conserved hypothetical protein	NA	NA	-0,1	0,8052237	0,0	0,9235157	-0,3	0,4118374	0,2	0,6010688
	EF0934		Unknown function	Enzymes of unknown specificity	hydrolase, TatD family	-0,5	0,1729633	-0,1	0,9067019	0,3	0,2911233	0,0	0,9710906	0,0	0,8878693
	EF0935		Unknown function	General	primase-related protein	-0,3	0,4612494	0,0	0,9498547	0,1	0,7788165	0,5	0,168531	-0,1	0,7951951
	EF0936	*ksgA*	Protein synthesis	tRNA and rRNA base modification	dimethyladenosine transferase	NA	NA	0,1	0,873894	-0,1	0,7912047	0,2	0,4872607	0,1	0,6629142
	EF0937		Hypothetical proteins	Conserved	conserved hypothetical protein	1,2	0,0057711	0,1	0,8531449	0,0	0,9998292	0,0	0,9697206	-0,3	0,366139
	EF0938		Transport and binding proteins	Unknown substrate	ABC transporter, ATP-binding/TOBE domain protein	0,7	0,0420075	1,1	0,0149618	0,4	0,2347637	0,5	0,1703464	-0,2	0,5979302
	EF0939	*mgsA*	Energy metabolism	Other	methylglyoxal synthase	1,6	**0,0000096**	0,3	0,5835898	0,3	0,283561	0,0	0,9470256	-0,5	0,1694769
	EF0940		Hypothetical proteins	Conserved	conserved hypothetical protein	0,6	0,1000143	0,3	0,511527	0,5	0,1167116	0,2	0,6431172	0,0	0,9662373
	EF0941		Transport and binding proteins	Unknown substrate	ABC transporter, ATP-binding/permease protein	NA	NA	-0,2	0,6098402	0,3	0,3887648	0,3	0,3295491	0,4	0,2747575
	EF0942		Transport and binding proteins	Unknown substrate	ABC transporter, ATP-binding/permease protein	-0,2	0,6054041	-0,1	0,7607908	0,4	0,2588546	0,1	0,7355692	0,1	0,6779197
	EF0943		Hypothetical proteins	Conserved	conserved hypothetical protein	1,2	0,0013821	0,4	0,3918821	0,0	0,9912833	-0,2	0,6572285	0,2	0,6401897
	EF0944		Cell envelope	Other	extracellular protein, putative	-1,4	0,0000957	-1,0	0,0387072	-0,1	0,7913023	-0,1	0,777655	-0,5	0,1160958
	EF0945		Unknown function	Enzymes of unknown specificity	acetyltransferase, GNAT family	-1,6	0,0003179	-0,1	0,8061102	0,1	0,7223415	0,1	0,8647517	0,0	0,9522702
	EF0946		Hypothetical proteins	Conserved	conserved hypothetical protein	0,5	0,1590008	0,6	0,2124125	0,3	0,2928533	0,3	0,3475357	0,3	0,4208477
	EF0947		Unknown function	Enzymes of unknown specificity	hydrolase, haloacid dehalogenase-like family	0,4	0,2864967	0,7	0,1282791	0,4	0,2285104	0,2	0,5727762	0,0	0,9129528
	EF0948	*ung*	DNA metabolism	DNA replication, recombination, and repair	uracil-DNA glycosylase	-0,1	0,8654542	0,0	0,9237275	-0,2	0,6099039	0,2	0,5222207	0,0	0,9956464
	EF0949	*eutD*	Energy metabolism	Fermentation	phosphotransacetylase	-1,1	0,0038782	-0,7	0,1399257	0,1	0,7264947	0,0	0,9389614	0,2	0,4978518
	EF0950		Hypothetical proteins	Conserved	conserved hypothetical protein TIGR00150	-1,0	0,0096957	-0,4	0,3450855	-0,2	0,5008413	0,1	0,8408209	0,2	0,6082754
	EF0951		Unknown function	Enzymes of unknown specificity	acetyltransferase, GNAT family	-0,3	0,4703431	0,0	0,9224677	-0,6	0,0528491	-0,6	0,0629995	0,5	0,1636466
	EF0953		Hypothetical proteins		hypothetical protein	NA	NA	0,3	0,5338053	0,2	0,5167383	NA	NA	0,4	0,2744917
	EF0954	*malR/bopD*	Regulatory functions	DNA interactions	sugar-binding transcriptional regulator, LacI family	0,9	0,0179408	0,5	0,2504297	0,1	0,6657962	0,3	0,4419822	-0,1	0,8791914
	EF0955	*malM/bopC*	Energy metabolism	Sugars	aldose 1-epimerase, putative	1,4	0,0016144	0,4	0,4428153	0,9	0,0045509	-0,2	0,5309767	-0,2	0,5622031
	EF0956	*malB/bopB*	Energy metabolism	Sugars	beta-phosphoglucomutase	2,5	**0**	1,0	0,0267526	0,3	0,3538521	0,2	0,4843652	0,0	0,9008423
	EF0957	*malP/bopA*	Energy metabolism	Biosynthesis and degradation of polysaccharides	glycosyl hydrolase, family 65	2,5	**0**	1,0	0,0287942	0,2	0,4981198	0,1	0,8096625	-0,2	0,5695097
	EF0958	*malT*	Signal transduction	PTS	PTS system, IIABC components	2,2	**0**	1,0	0,0307786	0,4	0,1845135	0,0	0,9926296	0,0	0,9436923
	EF0959		Hypothetical proteins		hypothetical protein	NA	NA	NA	NA	NA	NA	NA	NA	NA	NA
	EF0960		Unknown function	Enzymes of unknown specificity	endonuclease/exonuclease/phosphatase family protein	1,1	0,0127219	0,6	0,2110805	0,5	0,1343117	0,6	0,0556999	0,2	0,59724
	EF0961		Amino acid biosynthesis	Glutamate family	pyrroline-5-carboxylate reductase, putative	0,3	0,486072	0,0	0,9896424	0,2	0,5163862	0,2	0,4733188	-0,5	0,1115011
	EF0962		Regulatory functions	DNA interactions	transcriptional regulator, AraC family	-0,4	0,3207593	0,3	0,5335749	-0,2	0,4739165	-0,1	0,7245972	0,0	0,9568958
	EF0963		Hypothetical proteins		hypothetical protein	NA	NA	0,3	0,4564551	0,1	0,6560231	0,5	0,1159423	-0,3	0,4195616
	EF0964		Hypothetical proteins	Conserved	conserved hypothetical protein	-0,3	0,5706344	-0,3	0,5487998	0,3	0,4071162	0,6	0,0714821	-0,4	0,2570065
	EF0965		Hypothetical proteins	Conserved	conserved hypothetical protein	0,9	0,0438236	0,1	0,7761123	-0,1	0,6960854	0,5	0,1344218	-0,1	0,7520377
	EF0966		Regulatory functions	DNA interactions	transcriptional regulator, MerR family	0,4	0,3090057	-0,2	0,5997202	0,2	0,5148653	0,7	0,0485736	-0,2	0,6386491
	EF0967		Hypothetical proteins	Domain	conserved domain protein	NA	NA	0,4	0,4109245	-0,1	0,7754296	0,3	0,4037241	-0,1	0,7699684
	EF0968	*rplU*	Protein synthesis	Ribosomal proteins: synthesis and modification	ribosomal protein L21	-3,8	**0**	-1,0	0,0328301	-0,3	0,403788	-0,4	0,2922593	0,0	0,9573333
	EF0969		Hypothetical proteins	Conserved	conserved hypothetical protein	NA	NA	0,2	0,6806969	-0,1	0,8237022	-0,3	0,3878192	0,1	0,7141158
	EF0970	*rpmA*	Protein synthesis	Ribosomal proteins: synthesis and modification	ribosomal protein L27	-3,7	**0**	-1,0	0,0274446	0,0	0,9838954	-0,2	0,4651183	0,0	0,9421902
	EF0971		Hypothetical proteins	Conserved	conserved hypothetical protein	NA	NA	0,4	0,4064836	0,0	0,9445905	0,4	0,2468569	0,0	0,925635
	EF0972		DNA metabolism	DNA replication, recombination, and repair	DNA repair exonuclease family protein	0,3	0,4031954	-0,4	0,3870353	-0,2	0,6232276	-0,1	0,8484847	-0,2	0,5005956
	EF0973	*pepQ-1*	Protein fate	Degradation of proteins, peptides, and glycopeptides	proline dipeptidase	0,3	0,3445094	0,3	0,5190339	0,0	0,9138623	0,4	0,1856381	0,0	0,8897065
	EF0974		Hypothetical proteins	Conserved	conserved hypothetical protein	0,5	0,2746578	0,4	0,35242	0,0	0,9017258	0,4	0,2543465	0,0	0,8979068
	EF0976		Hypothetical proteins	Conserved	conserved hypothetical protein	0,0	0,9152414	-0,1	0,8520761	0,1	0,7965043	0,3	0,3074096	0,1	0,660435
	EF0977	*nusB*	Transcription	Transcription factors	N utilization substance protein B	0,1	0,8560806	-0,1	0,8707432	0,1	0,8707563	0,3	0,3846399	-0,1	0,6852572
	EF0978	*folD*	Biosynthesis of cofactors, prosthetic groups, carriers	Folic acid	methylenetetrahydrofolate dehydrogenase	-0,1	0,8494296	0,3	0,5726444	-0,2	0,5140888	0,5	0,1265746	0,1	0,7336209
	EF0979	*xseA*	DNA metabolism	Degradation of DNA	exodeoxyribonuclease VII, large subunit	-0,8	0,0365441	0,0	0,9961515	0,1	0,6596766	0,3	0,4147184	-0,2	0,5849234
	EF0980	*xseB*	DNA metabolism	Degradation of DNA	exodeoxyribonuclease VII, small subunit	-0,9	0,0380932	0,2	0,7067388	-0,1	0,7629184	0,1	0,8090398	0,1	0,8021046
	EF0981	*ispA*	Biosynthesis of cofactors, prosthetic groups, carriers	Menaquinone and ubiquinone	geranyltranstransferase	NA	NA	0,0	0,9457478	0,2	0,6342458	0,3	0,3409463	-0,2	0,6262059
	EF0982	*tlyA*	Cellular processes	Pathogenesis	hemolysin A	-1,2	0,0006997	-0,1	0,9016316	-0,1	0,7789685	0,0	0,8916626	0,0	0,960417
	EF0983		Regulatory functions	DNA interactions	transcriptional regulator, ArgR family	0,9	0,0172318	-0,1	0,8312656	0,1	0,7624	0,3	0,3053747	-0,1	0,7940283
	EF0984	*recN*	DNA metabolism	DNA replication, recombination, and repair	DNA repair protein RecN	-0,2	0,5657378	0,3	0,4901773	-0,1	0,8010998	0,4	0,188297	0,1	0,7435985
	EF0985		Hypothetical proteins		hypothetical protein	NA	NA	0,2	0,6369616	0,0	0,9447273	0,4	0,3635647	-0,5	0,1495108
	EF0986		Transport and binding proteins	Cations and iron carrying compounds	cation transporter	-0,4	0,4194606	0,1	0,8416707	0,4	0,2018285	0,3	0,4434977	-0,2	0,4581809

Operon[a)]	Locus	Gene	Functional category	Subcategory	Putative function	Blood[b)]	P-value[c)]	YTB_5[b)]	P-value[c)]	YTB_15[b)]	P-value[c)]	YTB_30[b)]	P-value[c)]	YTB_60[b)]	P-value[c)]
	EF0987		Cell envelope	Other	lipoprotein, putative	NA	NA	-0,9	0,061459	1,0	0,0019672	0,3	0,3783601	-0,1	0,7729825
	EF0988		Hypothetical proteins	Conserved	conserved hypothetical protein TIGR00242	-0,3	0,3507525	-0,3	0,4931475	0,7	0,0189787	0,4	0,2514659	0,1	0,7304135
	EF0989		Hypothetical proteins	Conserved	conserved hypothetical protein TIGR00006	0,1	0,8485723	-0,5	0,2771327	0,7	0,0233803	0,3	0,40866	0,0	0,9371598
	EF0990		Cellular processes	Cell division	cell division protein	0,0	0,9221391	-0,5	0,3280405	0,5	0,0854354	0,5	0,1252136	0,3	0,3824308
	EF0991	*pbpC*	Cell envelope	Biosynthesis/degradation of murein sacculus/peptidoglycan	penicillin-binding protein C	-1,5	0,0000454	-0,6	0,1953434	0,0	0,9090723	-0,1	0,736795	0,2	0,6375303
	EF0992	*mraY*	Cell envelope	Biosynthesis/degradation of murein sacculus/peptidoglycan	phospho-N-acetylmuramoyl-pentapeptide-transferase	-2,0	**0**	-0,9	0,0651059	-0,1	0,8431863	-0,4	0,2464658	0,0	0,9532923
	EF0993	*murD*	Cell envelope	Biosynthesis/degradation of murein sacculus/peptidoglycan	UDP-N-acetylmuramoylalanine--D-glutamate ligase	-2,0	**0**	-1,2	0,008467	0,1	0,7244005	-0,2	0,618544	0,1	0,8494828
	EF0994	*murG*	Cell envelope	Biosynthesis/degradation of murein sacculus/peptidoglycan	UDP-N-acetylglucosamine-N-acetylmuramyl pyrophosphoryl transf	-2,7	**0**	-0,8	0,0710026	0,0	0,9184004	-0,1	0,7609821	0,1	0,8578166
	EF0995	*ftsQ*	Cellular processes	Cell division	cell division protein FtsQ	-0,9	0,0196032	-0,4	0,3915222	0,0	0,9636092	0,1	0,8442187	-0,1	0,7687518
	EF0996	*ftsA*	Cellular processes	Cell division	cell division protein FtsA	-0,8	0,0343605	-0,2	0,6408973	0,0	0,9400501	-0,1	0,8703889	0,1	0,8360292
	EF0997	*ftsZ*	Cellular processes	Cell division	cell division protein FtsZ	-0,7	0,0514354	-0,2	0,5948696	0,0	0,9465682	0,0	0,9840077	0,0	0,8921653
	EF0998		Hypothetical proteins	Conserved	conserved hypothetical protein TIGR00044	-0,7	0,048579	-0,5	0,3243835	0,2	0,5202146	0,0	0,9596036	0,1	0,774833
	EF0999		Hypothetical proteins	Conserved	conserved hypothetical protein	-0,9	0,0196568	-0,5	0,3098724	0,2	0,4843915	0,0	0,9955559	0,1	0,7112887
	EF1000		Hypothetical proteins	Conserved	conserved hypothetical protein	-0,4	0,226193	-0,3	0,4662363	0,4	0,1781064	-0,1	0,7491004	0,1	0,7612859
	EF1001		Unknown function	General	S4 domain protein	-0,4	0,3356642	-0,4	0,4039287	0,1	0,6689492	0,0	0,9320714	-0,1	0,7849128
	EF1002	*divIVA*	Cellular processes	Cell division	cell division protein DivIVA	0,0	0,9717799	0,0	0,990443	-0,3	0,3931786	-0,1	0,7628965	0,0	0,9459879
	EF1003	*ileS*	Protein synthesis	tRNA aminoacylation	isoleucyl-tRNA synthetase	-1,0	0,0092365	-0,5	0,235618	-0,3	0,3986677	-0,8	0,0133018	0,0	0,9126503
	EF1004	*zwf*	Energy metabolism	Pentose phosphate pathway	glucose-6-phosphate 1-dehydrogenase	-1,6	0,0000175	-0,3	0,4958041	0,2	0,5801535	0,1	0,8633115	0,0	0,9860554
	EF1005		Regulatory functions	DNA interactions	iron-dependent repressor	-1,0	0,0101889	-0,8	0,0875099	0,2	0,4520529	0,5	0,1186119	-0,1	0,8265714
	EF1006		Hypothetical proteins	Conserved	conserved hypothetical protein	1,4	0,0015438	0,4	0,3579487	0,8	0,0148069	0,4	0,2600763	-0,2	0,6135369
	EF1008		Unknown function	Enzymes of unknown specificity	oxidoreductase, Gfo/Idh/MocA family	0,8	0,0363832	0,3	0,5077756	0,3	0,3362797	0,4	0,2690004	0,0	0,8954251
	EF1009		Transcription	Other	ATP-dependent RNA helicase, DEAD/DEAH box family	1,0	0,0050223	0,2	0,640762	0,2	0,4565143	0,3	0,4408175	-0,2	0,4974993
	EF1010		Regulatory functions	Protein interactions	sigma-54 interaction domain protein	-0,4	0,3241182	0,0	0,9429573	0,4	0,2472911	-0,2	0,6177697	0,0	0,9484644
	EF1012		Signal transduction	PTS	PTS system, IIB component	-0,4	0,3397756	0,0	0,9893917	0,4	0,1689036	0,0	0,9958546	0,6	0,0834257
	EF1013		Signal transduction	PTS	PTS system, IIC component	NA	NA	NA	NA	NA	NA	NA	NA	NA	NA
	EF1014		Hypothetical proteins		hypothetical protein	1,5	0,0000267	1,3	0,006005	0,9	0,0046092	0,5	0,1009019	0,3	0,3656099
	EF1015		Hypothetical proteins		hypothetical protein	1,3	0,0034143	1,0	0,0404128	1,1	0,0004258	0,8	0,012574	-0,2	0,6516501
	EF1016		Hypothetical proteins	Conserved	conserved hypothetical protein	1,0	0,0111321	1,2	0,0070843	1,2	0,0000848	0,6	0,081604	0,5	0,1599723
	EF1017		Signal transduction	PTS	PTS system, IIB component	-0,7	0,0418259	0,0	0,9355471	0,5	0,1181646	0,0	0,942863	0,5	0,1578718
	EF1018		Signal transduction	PTS	PTS system, IIA component	-0,8	0,0881409	0,1	0,8225676	0,2	0,4377395	-0,2	0,5149083	0,2	0,4725577
	EF1019		Signal transduction	PTS	PTS system, IIC component	-0,6	0,1209627	0,3	0,479264	0,1	0,8525372	-0,4	0,179946	0,4	0,2664598
	EF1020		Energy metabolism	Biosynthesis and degradation of polysaccharides	glycosyl hydrolase, family 1	-0,2	0,6291029	0,5	0,2428682	0,1	0,7870982	0,0	0,9897419	0,5	0,1343222
	EF1021		Hypothetical proteins	Conserved	conserved hypothetical protein	-0,1	0,8571862	0,9	0,061438	0,4	0,2102729	0,3	0,3947918	0,3	0,3682347
	EF1022		Hypothetical proteins		hypothetical protein	3,0	**0**	0,3	0,650402	0,2	0,4693932	0,3	0,3770385	0,0	0,9953737
	EF1023		Hypothetical proteins	Conserved	conserved hypothetical protein	NA	NA	NA	NA	NA	NA	NA	NA	0,1	0,7502803
	EF1024	*ppdK*	Energy metabolism	Other	pyruvate phosphate dikinase	5,0	**0**	0,5	0,2642246	-0,3	0,4057952	-1,2	0,0005529	0,0	0,9416514
	EF1025		Unknown function	General	CBS domain protein	-0,2	0,5056379	-0,6	0,2230662	-0,5	0,1002855	-0,4	0,2433661	-0,2	0,5209658
	EF1026		Hypothetical proteins	Conserved	conserved hypothetical protein	0,4	0,281433	-0,4	0,3451944	-0,6	0,0489374	-0,7	0,0263754	0,0	0,9926326
	EF1028		Unknown function	Enzymes of unknown specificity	hydrolase, alpha/beta hydrolase fold family	NA	NA	-0,4	0,4467403	0,0	0,9012442	0,3	0,3011524	0,0	0,9358867
	EF1029		Hypothetical proteins	Conserved	conserved hypothetical protein	NA	NA	-0,1	0,8227612	0,1	0,7409661	0,0	0,9548261	0,4	0,2035567
	EF1030		Unknown function	Enzymes of unknown specificity	endonuclease/exonuclease/phosphatase family protein	NA	NA	-0,1	0,7906143	0,4	0,1739775	0,0	0,8893226	0,2	0,5887235
	EF1031		Unknown function	Enzymes of unknown specificity	phosphorylase family protein	NA	NA	0,1	0,8293946	0,4	0,2081015	NA	NA	-0,1	0,8439243
	EF1032	*drrC*	Transport and binding proteins	Other	daunorubicin resistance protein	1,3	0,0006471	0,5	0,2670967	0,3	0,3882366	0,5	0,1127766	0,1	0,6856865
	EF1033		Central intermediary metabolism	Other	6-aminohexanoate-cyclic-dimer hydrolase, putative	1,5	0,0000662	1,0	0,0233341	1,4	**0,0000056**	0,4	0,2343263	-0,2	0,5816456
	EF1034		Hypothetical proteins	Domain	conserved domain protein	0,5	0,1812024	0,6	0,1788415	0,4	0,2248294	0,1	0,8123627	0,1	0,7581422
	EF1035		Cell envelope	Other	lipoprotein, putative	NA	NA	0,9	0,057409	0,0	0,9151057	0,1	0,7572775	-0,2	0,5349592
	EF1036		Purines, pyrimidines, nucleosides, and nucleotides	Nucleotide and nucleoside interconversions	nucleoside diphosphate kinase	1,3	0,0008363	0,5	0,2596738	0,6	0,0676802	0,1	0,7871546	0,1	0,8206257
	EF1037		Amino acid biosynthesis	Aspartate family	L-aspartate beta-decarboxylase, putative	0,4	0,3698342	0,0	0,9307197	0,1	0,6858524	NA	NA	-0,2	0,5166844
	EF1038		Cell envelope	Other	lipoprotein, putative	0,9	0,0108979	0,5	0,3165036	-0,4	0,1985478	0,0	0,9561933	-0,4	0,2721172
	EF1039		Unknown function	Enzymes of unknown specificity	hydrolase, haloacid dehalogenase-like family	0,8	0,0380923	-0,1	0,9028194	0,1	0,6740539	-0,2	0,5513829	0,0	0,9310657
	EF1040		Hypothetical proteins	Conserved	conserved hypothetical protein	NA	NA	0,9	0,0463484	0,3	0,349022	0,2	0,4690123	0,1	0,7108217
	EF1041		Transport and binding proteins	Nucleosides, purines and pyrimidines	xanthine/uracil permeases family protein	NA	NA	0,4	0,3920923	0,1	0,777724	0,5	0,1356812	0,4	0,2756269
	EF1042		Transport and binding proteins	Other	multidrug resistance protein, putative	0,4	0,2646273	0,4	0,3989219	0,5	0,1395467	0,5	0,1621298	0,2	0,5223717
	EF1043		Hypothetical proteins	Conserved	conserved hypothetical protein	-0,6	0,1237773	0,2	0,6568166	-0,1	0,7314567	-0,1	0,7226464	0,2	0,6255098
	EF1044	*dnaE*	DNA metabolism	DNA replication, recombination, and repair	DNA polymerase III, alpha subunit	-0,3	0,4746212	-0,2	0,6336683	0,0	0,9675996	-0,3	0,4967481	-0,3	0,3501278
	EF1045	*pfk*	Energy metabolism	Glycolysis/gluconeogenesis	6-phosphofructokinase	-1,6	**0,0000083**	-0,8	0,0963867	-0,1	0,7062341	0,1	0,8372918	0,0	0,9746274
	EF1046	*pyk*	Energy metabolism	Glycolysis/gluconeogenesis	pyruvate kinase	-1,8	**0,0000006**	-0,5	0,2421613	-0,3	0,4002872	0,1	0,7804788	0,1	0,8285456
	EF1047		Hypothetical proteins	Conserved	conserved hypothetical protein	-2,0	**0,0000001**	-0,5	0,2751266	0,1	0,8057886	0,5	0,2474329	0,4	0,2150369
	EF1048	*rpmF-3*	Protein synthesis	Ribosomal proteins: synthesis and modification	ribosomal protein L32	NA	NA	0,2	0,7252428	-0,3	0,3653877	NA	NA	0,3	0,3259742
	EF1049	*gnd*	Energy metabolism	Pentose phosphate pathway	6-phosphogluconate dehydrogenase, decarboxylating	-0,9	0,0158355	-0,2	0,6747499	0,1	0,7769351	-0,1	0,84875	-0,1	0,8517986
	EF1050	*etaR*	Signal transduction	Two-component systems	DNA-binding response regulator	-1,5	0,0000643	-0,6	0,2259827	-0,1	0,6458868	-0,2	0,5626458	-0,1	0,7353721
	EF1051	*etaS*	Signal transduction	Two-component systems	sensor histidine kinase	-2,6	**0**	-0,4	0,3480035	-0,1	0,7504618	-1,1	0,005333	-0,1	0,6821333
	EF1052		Hypothetical proteins		hypothetical protein	NA	NA	NA	NA	NA	NA	NA	NA	NA	NA
	EF1053		Transport and binding proteins	Unknown substrate	ABC transporter, ATP-binding protein	NA	NA	0,5	0,3068132	-0,5	0,2213541	NA	NA	0,2	0,6427475
	EF1054		Transport and binding proteins	Unknown substrate	ABC transporter, permease protein	NA	NA	0,5	0,2884664	NA	NA	NA	NA	-0,2	0,6357366
	EF1055	*efaR*	Cellular processes	Toxin production and resistance	tunicamycin resistance protein, putative	NA	NA	1,3	0,0060827	-0,7	0,0386159	NA	NA	0,1	0,8276022
	EF1056		Hypothetical proteins		hypothetical protein	NA	NA	NA	NA	NA	NA	NA	NA	NA	NA
	EF1057		Transport and binding proteins	Cations and iron carrying compounds	Mn2+/Fe2+ transporter, NRAMP family	1,3	0,0048916	0,5	0,2972881	0,4	0,1681052	0,5	0,1333042	-0,5	0,1392818
	EF1058		Cellular processes	Adaptations to atypical conditions	universal stress protein family	1,4	0,002209	0,9	0,0965518	NA	NA	NA	NA	-0,5	0,1313211
	EF1059		Hypothetical proteins	Conserved	conserved hypothetical protein	1,1	0,015624	1,1	0,0614851	NA	NA	0,7	0,0517604	-0,3	0,3266028
	EF1060		Transport and binding proteins	Amino acids, peptides and amines	pheromone binding protein	NA	NA	1,3	0,0254876	NA	NA	NA	NA	-0,3	0,3373471
	EF1061		Unknown function	Enzymes of unknown specificity	N-acyl-D-amino-acid deacylase family protein	NA	NA	NA	NA	NA	NA	NA	NA	-0,3	0,3175759
	EF1062		Unknown function	Enzymes of unknown specificity	N-acyl-D-amino-acid deacylase family protein	1,1	0,0036881	0,8	0,1453698	NA	NA	NA	NA	-0,1	0,8377677
	EF1063		Hypothetical proteins	Conserved	conserved hypothetical protein	1,0	0,0090518	0,1	0,901849	0,5	0,0911225	0,5	0,1040903	-0,1	0,6784504
	EF1065		Hypothetical proteins		hypothetical protein	NA	NA	NA	NA	NA	NA	NA	NA	NA	NA
	EF1066		Unknown function	Enzymes of unknown specificity	hexapeptide-repeat containing-acetyltransferase	0,5	0,287566	-0,7	0,1430011	0,5	0,0906727	0,6	0,0596229	-0,1	0,6606332
	EF1067		Hypothetical proteins		hypothetical protein	NA	NA	1,0	0,0910337	NA	NA	0,3	0,4615053	0,0	0,8980703
	EF1068	*galM*	Energy metabolism	Sugars	aldose 1-epimerase	1,4	0,0000871	0,6	0,1994426	0,3	0,355146	-0,2	0,5146876	-0,5	0,1666455
	EF1069	*galK*	Energy metabolism	Sugars	galactokinase	0,8	0,0425651	0,4	0,4133766	0,4	0,1813423	-0,2	0,5127941	-0,3	0,3772006
	EF1070	*galE-1*	Energy metabolism	Sugars	UDP-glucose 4-epimerase	NA	NA	0,1	0,8347425	0,0	0,9268208	-0,2	0,5645733	-0,2	0,5031334
	EF1071	*galT*	Energy metabolism	Sugars	galactose-1-phosphate uridylyltransferase	0,9	0,0362453	0,2	0,6106359	0,4	0,214592	0,0	0,9722636	-0,2	0,5495195
	EF1072	*galR*	Regulatory functions	DNA interactions	galactose operon repressor galR	0,4	0,3920539	0,4	0,5039214	0,4	0,1821698	0,0	0,9833523	-0,3	0,3275824
	EF1073		Hypothetical proteins	Conserved	conserved hypothetical protein	0,8	0,0839754	-0,3	0,4711279	0,2	0,4332953	0,5	0,1520086	-0,3	0,3880415

Operon[a]	Locus	Gene	Functional category	Subcategory	Putative function	Blood[b]	P-value[c]	YTB_5[b]	P-value[c]	YTB_15[b]	P-value[c]	YTB_30[b]	P-value[c]	YTB_60[b]	P-value[c]
	EF1074		Hypothetical proteins		hypothetical protein	1,8	0,0000533	0,2	0,6757693	0,0	0,8919887	0,8	0,0184884	-0,2	0,5734034
	EF1075		Unknown function	Enzymes of unknown specificity	acetyltransferase, GNAT family	NA	NA	1,1	0,0601335	NA	NA	0,4	0,3475321	-0,3	0,4460181
	EF1076		Cellular processes	Toxin production and resistance	streptomycin 3-adenylyltransferase, putative	0,9	0,0218284	0,5	0,2426005	0,2	0,513926	0,6	0,0949315	-0,7	0,0465895
	EF1077		Unknown function	Enzymes of unknown specificity	acetyltransferase, GNAT family	NA	NA	1,1	0,0620137	NA	NA	0,6	0,1376485	-0,3	0,3578883
	EF1078		Transport and binding proteins	Other	multidrug resistance protein, putative	-1,6	**0,0000156**	-0,5	0,2993527	-0,2	0,5071725	0,2	0,5944985	-0,1	0,6766449
	EF1080		DNA metabolism	DNA replication, recombination, and repair	ImpB/MucB/SamB family protein	1,5	0,0000378	-0,1	0,8570808	-0,4	0,170491	0,0	0,8926898	-0,7	0,0356182
	EF1081		Hypothetical proteins	Conserved	conserved hypothetical protein	1,0	0,0068152	-0,2	0,6105901	-0,1	0,6485256	-0,4	0,3303692	-0,4	0,2012723
	EF1082		Hypothetical proteins		hypothetical protein	1,6	0,0006007	0,7	0,2397354	0,7	0,0833699	0,5	0,1079192	-0,5	0,1517009
	EF1083		Hypothetical proteins		hypothetical protein	NA	NA	NA	NA	NA	NA	NA	NA	NA	NA
	EF1084		Cellular processes	Adaptations to atypical conditions	universal stress protein family	2,6	**0**	-0,3	0,5201512	0,4	0,2133607	0,4	0,2497145	-0,1	0,744452
	EF1085		Hypothetical proteins	Domain	conserved domain protein	1,5	0,0010358	0,7	0,1867086	NA	NA	0,6	0,1506885	0,9	0,0333682
	EF1086		Energy metabolism	Amino acids and amines	spermine/spermidine acetyltransferase, putative	NA	NA	NA	NA	NA	NA	NA	NA	NA	NA
	EF1088		Hypothetical proteins	Conserved	conserved hypothetical protein	NA	NA	0,3	0,5731208	1,6	0,0000465	NA	NA	-0,3	0,407805
	EF1089		Hypothetical proteins	Conserved	conserved hypothetical protein	0,6	0,1062154	0,2	0,7388365	0,4	0,1646048	0,3	0,3587233	0,1	0,8270107
	EF1090		Hypothetical proteins		hypothetical protein	NA	NA	0,4	0,3925042	NA	NA	NA	NA	0,0	0,9052128
	EF1091	*ebpA*	Unknown function	General	von Willebrand factor type A domain protein	-0,4	0,281552	-0,5	0,2793522	0,3	0,3485264	-0,1	0,8082763	0,5	0,125167
	EF1092	*ebpB*	Cell envelope	Other	cell wall surface anchor family protein	-0,7	0,1279743	-0,2	0,6528778	0,3	0,3860868	-0,1	0,6780974	0,4	0,1895358
	EF1093	*ebpC*	Cell envelope	Other	cell wall surface anchor family protein	NA	NA	0,4	0,3942147	0,5	0,1103048	0,1	0,8594007	0,5	0,1313375
	EF1094	*srtC*	Cell envelope	Surface structures	sortase family protein	NA	NA	-0,5	0,2783089	0,3	0,3220066	0,1	0,7348238	0,2	0,4965925
	EF1095		Hypothetical proteins		hypothetical protein	2,3	**0**	-0,3	0,5412794	0,4	0,2548212	0,6	0,0610725	0,1	0,6711499
	EF1096		Hypothetical proteins	Conserved	conserved hypothetical protein	NA	NA	0,0	0,9900268	0,7	0,0674047	0,5	0,1664275	0,1	0,8564713
	EF1097		Hypothetical proteins		hypothetical protein	NA	NA	1,3	0,0264195	NA	NA	NA	NA	0,5	0,1202123
	EF1098		Hypothetical proteins	Conserved	conserved hypothetical protein	-0,1	0,8963642	-0,2	0,6269302	-0,2	0,6266044	0,1	0,8016812	0,1	0,8789864
	EF1099	*ace*	Cell envelope	Other	collagen adhesin protein	NA	NA	NA	NA	NA	NA	NA	NA	NA	NA
	EF1100		Transport and binding proteins	Unknown substrate	ABC transporter, ATP-binding/permease protein	NA	NA	0,7	0,2045978	NA	NA	0,3	0,4148679	-0,2	0,5124574
	EF1101		Hypothetical proteins	Conserved	conserved hypothetical protein	NA	NA	-0,2	0,7261572	0,7	0,0432281	0,8	0,0195193	-0,1	0,8016289
	EF1102		Hypothetical proteins	Conserved	conserved hypothetical protein TIGR01655	NA	NA	0,8	0,1335736	NA	NA	NA	NA	NA	NA
	EF1103		Transport and binding proteins	Amino acids, peptides and amines	amino acid permease family protein	1,2	0,0082643	-0,4	0,3622758	-0,5	0,1497357	0,3	0,3926167	0,0	0,8887527
	EF1104		Hypothetical proteins	Domain	conserved domain protein	0,8	0,0835669	-0,1	0,8945524	-0,3	0,4698465	0,2	0,5445396	0,3	0,4434283
	EF1105		Hypothetical proteins		hypothetical protein	1,2	0,0082078	0,6	0,2225831	NA	NA	NA	NA	-0,3	0,4513521
	EF1106		Hypothetical proteins		hypothetical protein	NA	NA	0,3	0,5512088	NA	NA	NA	NA	NA	NA
	EF1107		Hypothetical proteins		hypothetical protein	-0,2	0,6416891	-0,2	0,7237713	-0,8	0,016464	-0,5	0,1497275	0,0	0,9063168
	EF1108		Unknown function	Enzymes of unknown specificity	oxidoreductase, putative	-0,4	0,2597972	-0,3	0,4895516	-0,9	0,0035117	-0,6	0,069473	0,1	0,6726635
	EF1109		Energy metabolism	Electron transport	iron-sulfur cluster binding protein	0,8	0,0288389	-0,1	0,8667184	-1,0	0,0009378	-0,8	0,0170267	0,0	0,9061367
	EF1110		Unknown function	General	YkgG family protein	1,1	0,0031474	0,1	0,8956524	-1,5	**0,0000031**	-1,2	0,0004396	0,1	0,7936105
	EF1111		Protein fate	Protein and peptide secretion and trafficking	signal peptidase I	1,3	0,0040956	0,3	0,5508539	0,6	0,0571165	0,8	0,0197993	-0,1	0,857361
	EF1112	*rexB*	DNA metabolism	DNA replication, recombination, and repair	exonuclease RexB	NA	NA	0,0	0,9689862	0,6	0,0646648	0,7	0,0451062	-0,1	0,887706
	EF1113	*rexA*	DNA metabolism	DNA replication, recombination, and repair	exonuclease RexA	0,3	0,3409991	-0,1	0,8491824	0,2	0,6338055	-0,1	0,8236952	0,0	0,9426089
	EF1114		Hypothetical proteins	Conserved	conserved hypothetical protein	-0,5	0,1657085	-0,6	0,1935772	0,0	0,9287462	-0,3	0,4368619	0,0	0,9842057
	EF1115	*pheS*	Protein synthesis	tRNA aminoacylation	phenylalanyl-tRNA synthetase, alpha subunit	0,7	0,0694019	0,0	0,9673414	0,0	0,9382217	0,3	0,3725629	-0,1	0,7974575
	EF1116	*pheT*	Protein synthesis	tRNA aminoacylation	phenylalanyl-tRNA synthetase, beta subunit	-0,1	0,8576191	-0,3	0,5526222	-0,3	0,3242998	-0,3	0,325054	0,0	0,95427
	EF1117		Transport and binding proteins	Amino acids, peptides and amines	amino acid ABC transporter, permease protein	NA	NA	0,3	0,4602724	NA	NA	NA	NA	0,4	0,2072711
	EF1118		Transport and binding proteins	Amino acids, peptides and amines	amino acid ABC transporter, permease protein	NA	NA	0,9	0,1268475	NA	NA	NA	NA	0,6	0,0637647
	EF1119		Transport and binding proteins	Amino acids, peptides and amines	amino acid ABC transporter, amino acid-binding protein	NA	NA	-0,9	0,1311349	NA	NA	NA	NA	-0,1	0,7564806
	EF1120		Transport and binding proteins	Amino acids, peptides and amines	amino acid ABC transporter, ATP-binding protein	0,4	0,333498	1,2	0,0337442	NA	NA	NA	NA	0,7	0,0244147
	EF1121	*murI*	Cell envelope	Biosynthesis/degradation of murein sacculus/peptidoglycan	glutamate racemase	-0,1	0,7795308	-0,8	0,1030515	0,4	0,2027351	0,8	0,0173476	-0,1	0,8283062
	EF1122	*rph*	Transcription	RNA processing	ribonuclease PH/Ham1 protein	0,5	0,2219619	-0,3	0,5370631	0,3	0,2859944	0,3	0,3161521	-0,1	0,7909762
	EF1123		Hypothetical proteins	Conserved	conserved hypothetical protein	0,8	0,0762648	0,1	0,8581553	0,7	0,0373111	0,8	0,0199354	0,1	0,8079444
	EF1124		Regulatory functions	DNA interactions	transcriptional regulator, DeoR family	NA	NA	1,1	0,0521691	NA	NA	0,6	0,1449382	0,0	0,9027369
	EF1125		Hypothetical proteins	Conserved	conserved hypothetical protein	NA	NA	0,7	0,2018159	NA	NA	1,0	0,0035822	0,3	0,3740743
	EF1126		Signal transduction	PTS	PTS system, IIA component	NA	NA	1,1	0,0541523	NA	NA	0,4	0,2325019	0,2	0,4562036
	EF1127	*sgaT*	Transport and binding proteins	Unknown substrate	putative transport protein SgaT protein	0,8	0,0926937	0,6	0,2436689	0,5	0,1174721	0,4	0,277068	0,1	0,7009389
	EF1128	*sgaB*	Transport and binding proteins	Carbohydrates, organic alcohols, and acids	phosphotransferase enzyme II, B compnent SgaB	1,9	**0,0000008**	0,6	0,278201	0,2	0,5254612	0,7	0,0502243	-0,2	0,4926781
	EF1129		Energy metabolism	Sugars	hexulose-6-phosphate synthase, putative	NA	NA	NA	NA	NA	NA	NA	NA	-0,2	0,4597707
	EF1130		Energy metabolism	Sugars	hexulose-6-phosphate isomerase SgbU, putative	NA	NA	NA	NA	NA	NA	NA	NA	-0,3	0,3797221
	EF1131	*araD*	Energy metabolism	Sugars	L-ribulose-5-phosphate 4-epimerase	NA	NA	1,4	0,0125702	NA	NA	NA	NA	0,1	0,7407763
	EF1132		Unknown function	General	CBS domain protein	0,2	0,6171145	-0,3	0,5737992	0,0	0,9025668	0,3	0,3705867	-0,5	0,158426
	EF1133	*dapD*	Amino acid biosynthesis	Aspartate family	2,3,4,5-tetrahydropyridine-2,6-dicarboxylate N-succinyltransferase	0,5	0,2070114	-0,2	0,5985029	0,0	0,8907649	0,3	0,4569345	0,1	0,7829961
	EF1134		Protein fate	Degradation of proteins, peptides, and glycopeptides	peptidase, M20/M25/M40 family	0,6	0,0999804	0,4	0,3524012	-0,2	0,6311125	0,0	0,9926014	-0,2	0,5117178
	EF1135		Hypothetical proteins	Conserved	conserved hypothetical protein	1,2	0,0015326	0,5	0,3272356	0,0	0,9800026	0,0	0,9315371	-0,5	0,1679141
	EF1136		Hypothetical proteins		hypothetical protein	-0,2	0,7068013	0,6	0,2122217	-0,1	0,8427351	-0,2	0,6465286	0,1	0,8554593
	EF1137		Hypothetical proteins		hypothetical protein	0,7	0,1304453	0,6	0,1674591	0,3	0,3995053	0,2	0,4819755	0,0	0,9581926
	EF1138		Unknown function	Enzymes of unknown specificity	oxidoreductase, aldo/keto reductase family	2,8	**0**	1,1	0,0216736	0,6	0,0504262	1,0	0,003301	-0,1	0,8055176
	EF1139		Unknown function	Enzymes of unknown specificity	glutamine amidotransferase, class I	0,3	0,5274574	-0,7	0,1134869	-0,1	0,8038781	0,7	0,0367631	-0,3	0,3703441
	EF1140	*gloA*	Energy metabolism	Other	lactoylglutathione lyase	1,2	0,0006522	0,2	0,6450583	0,8	0,0102563	0,8	0,0173697	-0,3	0,3316333
	EF1141		DNA metabolism	DNA replication, recombination, and repair	MutT/nudix family protein	1,9	**0,0000007**	0,7	0,1221305	0,5	0,1123603	0,7	0,0857273	0,1	0,8531592
	EF1142		Unknown function	Enzymes of unknown specificity	hydrolase, haloacid dehalogenase-like family	0,9	0,0167558	0,4	0,3958174	0,1	0,8302907	0,3	0,4078108	0,0	0,9762735
	EF1143		Unknown function	General	HD domain protein	0,9	0,0462343	0,3	0,5030608	1,0	0,0023499	0,6	0,0985747	0,0	0,9781916
	EF1144		Protein fate	Protein modification and repair	lipoate-protein ligase A family protein, putative	0,3	0,4934072	0,1	0,7950117	0,3	0,3094282	0,4	0,2653143	-0,1	0,8299213
	EF1145		Hypothetical proteins	Conserved	conserved hypothetical protein	0,1	0,8451505	0,0	0,9639592	0,1	0,7856286	0,4	0,2573749	-0,2	0,4870984
	EF1146		Transcription	DNA-dependent RNA polymerase	DNA-directed RNA polymerase, delta subunit, putative	-0,2	0,6495925	0,0	0,9890452	-0,5	0,0873225	-0,1	0,8076102	-0,1	0,6821279
	EF1147	*pyrG*	Purines, pyrimidines, nucleosides, and nucleotides	Pyrimidine ribonucleotide biosynthesis	CTP synthase	-1,3	0,0006421	-1,0	0,023197	-0,6	0,054756	-0,2	0,5805404	-0,6	0,0912699
	EF1148		Cell envelope	Biosynthesis/degradation of murein sacculus/peptidoglycan	penicillin-binding protein 1A	-0,5	0,1984289	-0,5	0,2843922	0,0	0,9095405	0,0	0,9728722	0,1	0,8231043
	EF1149	*recU*	DNA metabolism	DNA replication, recombination, and repair	recombination protein U	NA	NA	-0,6	0,1991639	0,5	0,1338235	1,1	0,001112	0,2	0,6032996
	EF1150		Hypothetical proteins	Conserved	conserved hypothetical protein	0,4	0,2321424	0,5	0,3042462	0,2	0,5513834	0,2	0,5012248	0,2	0,4818868
	EF1151		Cellular processes	Cell division	cell division protein DivIVA, putative	-0,4	0,292906	-0,4	0,3905451	-0,1	0,7566213	0,5	0,1708234	-0,2	0,5667542
	EF1152		Hypothetical proteins	Conserved	conserved hypothetical protein	-1,2	0,0012459	-0,2	0,6365282	-0,3	0,325776	-0,7	0,0473432	0,0	0,903881
	EF1153		Protein fate	Degradation of proteins, peptides, and glycopeptides	thermostable carboxypeptidase 1	-1,0	0,0083405	-0,4	0,4232143	-0,2	0,5121448	-0,3	0,3709742	0,0	0,9927152
	EF1154		DNA metabolism	DNA replication, recombination, and repair	DNA replication protein DnaD, putative	-1,1	0,0037179	-0,2	0,6950848	-0,1	0,7128741	-0,2	0,5103123	-0,1	0,7831711
	EF1155	*nth*	DNA metabolism	DNA replication, recombination, and repair	endonuclease III	-0,3	0,3736331	0,0	0,9297924	0,1	0,8710251	0,1	0,7129288	0,1	0,8211898
	EF1156		Regulatory functions	DNA interactions	transcriptional regulator, GntR family	NA	NA	0,7	0,1543773	-0,3	0,4089528	0,4	0,2761559	-0,3	0,4480639
	EF1157		Protein fate	Degradation of proteins, peptides, and glycopeptides	peptidase, M20/M25/M40 family	-0,1	0,79152	0,6	0,2295335	-0,2	0,4942625	-0,1	0,6788755	0,0	0,9864103
	EF1158		Energy metabolism	Biosynthesis and degradation of polysaccharides	N4-(beta-N-acetylglucosaminyl)-L-asparaginase, putative	0,6	0,1121874	0,4	0,4355542	-0,2	0,5751646	-0,2	0,5405971	-0,2	0,601909

Operon[a]	Locus	Gene	Functional category	Subcategory	Putative function	Blood[b]	P-value[c]	YTB_5[b]	P-value[c]	YTB_15[b]	P-value[c]	YTB_30[b]	P-value[c]	YTB_60[b]	P-value[c]
	EF1159		Signal transduction	PTS	PTS system, cellobiose-specific IIB component	NA	NA	0,7	0,2153568	-0,7	0,0245859	-0,1	0,7615889	-0,4	0,2729226
	EF1160		Signal transduction	PTS	PTS system, cellobiose-specific IIC component	NA	NA	0,7	0,1397447	-0,4	0,1654145	-0,6	0,0960575	-0,3	0,3300052
	EF1161		Hypothetical proteins	Domain	conserved domain protein	NA	NA	0,7	0,1852702	-0,8	0,0410281	NA	NA	-0,1	0,8255738
	EF1162		Unknown function	Enzymes of unknown specificity	helicase, putative	-0,1	0,8415815	-0,2	0,7281036	-0,1	0,8106585	-0,1	0,7578196	-0,4	0,1792479
	EF1163		Energy metabolism	Amino acids and amines	L-asparaginase, putative	-0,5	0,1618179	-0,1	0,7657509	-0,3	0,4269851	-0,3	0,34641	-0,3	0,4259549
	EF1164		Unknown function	General	HD domain protein	0,1	0,772169	-0,5	0,2627059	-0,3	0,3354423	-0,3	0,3535833	-0,2	0,5563912
	EF1165		Hypothetical proteins	Conserved	conserved hypothetical protein	0,6	0,1277423	0,4	0,4070921	-0,1	0,8668928	0,4	0,2139016	0,0	0,905906
	EF1166		Unknown function	General	YitT family protein	NA	NA	0,5	0,3302128	-0,6	0,0454646	0,1	0,8016761	-0,1	0,6866351
	EF1167	*fba*	Energy metabolism	Glycolysis/gluconeogenesis	fructose-bisphosphate aldolase class-II	-2,0	**0,0000001**	-0,2	0,6151268	-0,5	0,1348581	0,7	0,0392035	0,2	0,6114084
	EF1168		Hypothetical proteins		hypothetical protein	NA	NA	NA	NA	NA	NA	NA	NA	NA	NA
	EF1169	*murAB*	Cell envelope	Biosynthesis/degradation of murein sacculus/peptidoglycan	UDP-N-acetylglucosamine 1-carboxyvinyltransferase 2	-1,4	0,0002091	-0,7	0,1221603	-0,2	0,4435148	0,1	0,7500659	0,0	0,9368439
	EF1170	*rho*	Transcription	Transcription factors	transcription termination factor Rho	-1,3	0,000715	-0,6	0,2251778	-0,4	0,1583486	-0,3	0,3454182	-0,1	0,714196
	EF1171	*rpmE*	Protein synthesis	Ribosomal proteins: synthesis and modification	ribosomal protein L31	NA	NA	0,0	0,9706844	-0,5	0,1056269	0,1	0,7957473	0,2	0,4772336
	EF1172		Cell envelope	Biosynthesis/degradation of murein sacculus/peptidoglycan	teichoic acid biosynthesis protein B, putative	NA	NA	0,3	0,4997551	-0,1	0,8559556	0,3	0,3056606	0,5	0,1465249
	EF1173		Cell envelope	Biosynthesis and degradation of surface poly/liposaccharides	glycosyl transferase, WecB/TagA/CpsF family	NA	NA	0,4	0,4274918	-0,3	0,429846	0,5	0,2481829	0,2	0,5992311
	EF1174		Hypothetical proteins		hypothetical protein	NA	NA	NA	NA	-1,0	0,0066899	NA	NA	-0,2	0,5195266
	EF1175	*gct*	Cell envelope	Biosynthesis/degradation of murein sacculus/peptidoglycan	glycerol-3-phosphate cytidylyltransferase	0,0	0,973448	-0,4	0,4279214	-1,1	0,0034339	NA	NA	0,0	0,8926621
	EF1176		Hypothetical proteins	Conserved	conserved hypothetical protein	0,9	0,0165283	0,3	0,559663	-0,5	0,1414966	-0,5	0,1571399	0,2	0,5969995
	EF1177		Hypothetical proteins		hypothetical protein	NA	NA	NA	NA	NA	NA	NA	NA	NA	NA
	EF1179	*cscK*	Energy metabolism	Sugars	fructokinase	NA	NA	0,5	0,2982999	0,2	0,4935748	0,2	0,514701	-0,3	0,3909705
	EF1180		Hypothetical proteins	Conserved	conserved hypothetical protein	4,3	**0**	1,3	0,0061675	0,5	0,1272323	0,7	0,0388515	-0,3	0,3903982
	EF1181		Unknown function	Enzymes of unknown specificity	nitroreductase family protein	0,0	0,9936622	-0,4	0,3897266	0,3	0,2790359	0,5	0,1493402	0,0	0,888335
	EF1182	*luxS*	Cellular processes	Other	autoinducer-2 production protein LuxS	-1,4	0,0000811	-0,5	0,2747412	-0,3	0,2894912	-0,2	0,5486697	-0,2	0,5972955
	EF1183	*asd*	Amino acid biosynthesis	Aspartate family	aspartate-semialdehyde dehydrogenase	-0,7	0,0771054	-0,4	0,4148183	0,0	0,9960212	0,0	0,9426566	0,5	0,161402
	EF1184	*dapA*	Amino acid biosynthesis	Aspartate family	dihydrodipicolinate synthase	-1,6	0,0002232	-0,5	0,3221692	0,4	0,2321081	0,1	0,8134917	0,2	0,6457876
	EF1185		Unknown function	Enzymes of unknown specificity	metallo-beta-lactamase superfamily protein	-0,9	0,0162784	-0,2	0,6065944	0,2	0,5717282	0,0	0,9493862	0,1	0,6789738
	EF1186		Unknown function	Enzymes of unknown specificity	Ser/Thr protein phosphatase family protein	-0,5	0,1321676	-0,4	0,4115655	0,1	0,8623028	0,1	0,7564824	0,1	0,7625731
	EF1187		Hypothetical proteins	Conserved	conserved hypothetical protein	-0,4	0,261216	-0,4	0,4347966	0,4	0,1697965	0,2	0,5500333	0,1	0,7974259
	EF1188		Unknown function	Enzymes of unknown specificity	hydrolase, haloacid dehalogenase family	-0,7	0,0766289	-0,4	0,4484934	0,2	0,6297403	0,1	0,7730114	0,4	0,2768946
	EF1189		Hypothetical proteins	Conserved	conserved hypothetical protein	-0,9	0,0174155	-0,2	0,7426783	0,1	0,865697	-0,2	0,5126634	0,1	0,7562536
	EF1190		Hypothetical proteins	Conserved	conserved hypothetical protein	0,9	0,0419127	2,3	**0,0000004**	1,8	**0**	1,6	**0,0000009**	1,7	**0,0000002**
	EF1191		Unknown function	General	DegV family protein	1,4	0,0001086	3,0	**0**	2,1	**0**	1,6	**0,0000019**	1,4	0,0000229
	EF1192		Transport and binding proteins	Porins	aquaporin Z	NA	NA	NA	NA	NA	NA	NA	NA	NA	NA
	EF1193	*vicR*	Regulatory functions	DNA interactions	DNA-binding response regulator VicR	-0,5	0,1607874	-0,2	0,6978844	0,0	0,994338	0,1	0,8242253	0,2	0,5666287
	EF1194	*vicK*	Signal transduction	Two-component systems	sensory box histidine kinase VicK	-0,5	0,1526006	-0,1	0,8992413	-0,2	0,5959155	-0,1	0,8601753	0,3	0,3812917
	EF1195		Hypothetical proteins		hypothetical protein	-1,8	**0,000001**	-0,2	0,7026353	-0,1	0,859531	-0,3	0,4359291	0,2	0,4793864
	EF1196		Hypothetical proteins	Conserved	conserved hypothetical protein	-1,9	0,000026	0,0	0,9310194	0,1	0,6769565	-0,3	0,4259012	0,4	0,2528101
	EF1197		Unknown function	Enzymes of unknown specificity	metallo-beta-lactamase YycJ	-2,0	**0,0000001**	-0,3	0,467857	0,0	0,9766196	0,0	0,9106707	0,1	0,7195949
	EF1198		Hypothetical proteins	Conserved	conserved hypothetical protein	-2,1	**0,0000035**	-0,7	0,1555381	0,3	0,2825318	-0,1	0,6680349	-0,1	0,7991563
	EF1199		Hypothetical proteins	Conserved	conserved hypothetical protein	-2,5	**0**	-0,6	0,2035555	0,2	0,5271334	0,0	0,9295093	0,0	0,9342813
	EF1200		Cell envelope	Other	membrane protein, putative	0,8	0,0829118	0,0	0,9990737	-0,1	0,7241387	0,0	0,9265529	0,3	0,3198709
	EF1201		Hypothetical proteins	Conserved	conserved hypothetical protein	0,5	0,2198865	0,5	0,2988847	0,0	0,9599291	-0,1	0,7304319	-0,3	0,3228871
	EF1202		Hypothetical proteins	Conserved	conserved hypothetical protein	1,6	**0,0000156**	0,9	0,0642976	0,2	0,5522155	0,1	0,8096883	-0,1	0,7819164
	EF1203		Hypothetical proteins	Conserved	conserved hypothetical protein TIGR00250	2,0	**0,0000001**	0,9	0,0416612	0,3	0,3727848	0,4	0,225184	0,1	0,7356421
	EF1204		Hypothetical proteins	Conserved	conserved hypothetical protein	1,7	**0,0000041**	0,4	0,4317589	0,6	0,0605597	0,1	0,6785744	0,0	0,9911175
	EF1206		Energy metabolism	Other	malate dehydrogenase, decarboxylating	0,0	0,9612285	2,2	**0,0000027**	0,8	0,0157136	0,8	0,0147187	-2,3	**0**
	EF1207		Transport and binding proteins	Carbohydrates, organic alcohols, and acids	citrate carrier protein, CCS family	0,2	0,721214	1,2	0,038601	0,4	0,2222996	0,1	0,8309951	-2,0	**0**
	EF1209		Signal transduction	Two-component systems	sensory box histidine kinase	NA	NA	0,9	0,048699	0,7	0,0181677	0,4	0,2483033	-1,1	0,0005603
	EF1210		Signal transduction	Two-component systems	response regulator	-0,4	0,4023115	0,8	0,0856016	0,7	0,0267248	0,6	0,0678259	-1,2	0,0002276
	EF1211	*npr*	Energy metabolism	Electron transport	NADH peroxidase	2,8	**0**	0,5	0,2357851	0,4	0,1615781	1,2	0,000232	-0,7	0,039637
	EF1212		Regulatory functions	Other	transcriptional regulator	0,4	0,2504984	0,1	0,8056628	-0,4	0,2427363	0,0	0,9321793	0,2	0,5161247
	EF1213	*alsS*	Amino acid biosynthesis	Pyruvate family	acetolactate synthase, catabolic	NA	NA	-0,2	0,6344777	0,2	0,5496257	0,7	0,0472275	-0,2	0,5214565
	EF1214	*budA*	Energy metabolism	Fermentation	alpha-acetolactate decarboxylase	0,1	0,7078054	0,5	0,2778586	0,7	0,0180138	0,6	0,0705235	0,1	0,6593191
	EF1215		Hypothetical proteins		hypothetical protein	1,2	0,0014837	-0,1	0,8175647	0,4	0,1831065	0,2	0,6517098	0,2	0,5190254
	EF1216		Hypothetical proteins		hypothetical protein	NA	NA	0,0	0,9796384	NA	NA	0,0	0,9623415	0,1	0,7520348
	EF1217		Cell envelope	Other	lipoprotein, putative	NA	NA	0,4	0,3699681	-0,7	0,0424289	-0,3	0,3039464	0,0	0,9904974
	EF1218		Transport and binding proteins	Amino acids, peptides and amines	spermidine/putrescine ABC transporter, permease protein	-0,5	0,2753549	-0,4	0,3828671	-0,5	0,2003367	0,0	0,9157925	0,2	0,5273183
	EF1219		Transport and binding proteins	Amino acids, peptides and amines	spermidine/putrescine ABC transporter, permease protein	NA	NA	0,6	0,234522	-0,1	0,8770128	0,8	0,011871	0,5	0,141278
	EF1220		Transport and binding proteins	Amino acids, peptides and amines	spermidine/putrescine ABC transporter, ATP-binding protein	NA	NA	0,6	0,2128863	NA	NA	0,0	0,9673486	0,3	0,3679148
	EF1221		Transport and binding proteins	Amino acids, peptides and amines	spermidine/putrescine ABC transporter/-binding protein	NA	NA	-0,1	0,7940357	-0,1	0,8211152	-0,5	0,1049672	0,6	0,0867468
	EF1222	*ade*	Purines, pyrimidines, nucleosides, and nucleotides	Salvage of nucleosides and nucleotides	adenine deaminase	NA	NA	NA	NA	NA	NA	NA	NA	NA	NA
	EF1223		Unknown function	Enzymes of unknown specificity	chlorohydrolase family protein	0,9	0,0583263	0,1	0,8554604	0,3	0,4074071	0,3	0,3829943	0,0	0,9526757
	EF1224		Regulatory functions	DNA interactions	transcriptional regulator, Cro/CI family	0,4	0,3276314	0,2	0,7257927	-0,1	0,8316477	0,2	0,5833956	0,1	0,6543593
	EF1225		Biosynthesis of cofactors, prosthetic groups, carriers	Thiamine	thiamin biosynthesis ApbE, putative	-1,3	0,0002961	-1,5	0,0015906	-3,0	**0**	-1,5	**0,0000063**	-0,8	0,0110585
	EF1226		Unknown function	Enzymes of unknown specificity	oxidoreductase, putative	NA	NA	NA	NA	NA	NA	NA	NA	NA	NA
	EF1227		Hypothetical proteins	Conserved	conserved hypothetical protein	-2,4	**0**	-0,3	0,4838219	-2,7	**0**	-1,6	**0,0000014**	-0,4	0,2002808
	EF1228		Hypothetical proteins		hypothetical protein	0,4	0,2631926	-0,2	0,6101626	-0,2	0,4681585	-0,2	0,5452418	-0,2	0,4533043
	EF1229		Hypothetical proteins	Conserved	conserved hypothetical protein	NA	NA	0,3	0,5514833	-0,5	0,0847042	-0,2	0,6020814	0,1	0,728964
	EF1230		Hypothetical proteins		hypothetical protein	NA	NA	NA	NA	NA	NA	NA	NA	NA	NA
	EF1231		Hypothetical proteins	Conserved	conserved hypothetical protein	NA	NA	0,2	0,6994081	NA	NA	0,2	0,7022723	-0,5	0,134971
	EF1232		Transport and binding proteins	Unknown substrate	ABC transporter, permease protein	NA	NA	0,9	0,0569209	0,4	0,2109288	0,0	0,9511995	-0,1	0,671018
	EF1233		Transport and binding proteins	Unknown substrate	ABC transporter, permease protein	NA	NA	0,2	0,6744048	-0,3	0,4343809	-0,1	0,7122225	0,0	0,9409333
	EF1234		Transport and binding proteins	Unknown substrate	ABC transporter, substrate-binding protein, putative	NA	NA	0,4	0,3800813	0,0	0,9377009	0,0	0,9535211	0,0	0,961526
	EF1235		Hypothetical proteins		hypothetical protein	0,7	0,0561193	0,2	0,6195273	-0,2	0,5365021	0,0	0,9822862	0,2	0,4634619
	EF1236		Energy metabolism	Biosynthesis and degradation of polysaccharides	acetyl xylan esterase, putative	1,0	0,0045257	0,9	0,0595683	0,5	0,1354506	0,1	0,7557048	-0,2	0,5011041
	EF1237		Hypothetical proteins	Conserved	conserved hypothetical protein	0,9	0,0140487	0,0	0,9286564	0,7	0,0366371	0,2	0,5504701	-0,3	0,3375102
	EF1238		Energy metabolism	Biosynthesis and degradation of polysaccharides	glycosyl hydrolase, family 3	1,5	0,00005	0,7	0,1518629	0,4	0,239851	0,4	0,217022	-0,4	0,1891548
	EF1239		Hypothetical proteins	Conserved	conserved hypothetical protein	0,4	0,3573945	0,4	0,3352349	-0,6	0,0449442	-0,3	0,3903145	-0,2	0,4678343
	EF1240		Regulatory functions	DNA interactions	sugar-binding transcriptional regulator, LacI family	0,1	0,7136492	0,2	0,6916882	0,1	0,7531716	0,4	0,1910766	-0,1	0,8574814
	EF1241		Hypothetical proteins		hypothetical protein	-0,6	0,200119	-0,4	0,4490035	0,2	0,481181	0,3	0,3788042	0,3	0,3296374
	EF1242		Regulatory functions	DNA interactions	transcriptional regulator, GntR family	0,9	0,0169301	0,5	0,2703628	0,1	0,7983218	0,5	0,1006778	0,7	0,0265518
	EF1243		Energy metabolism	Biosynthesis and degradation of polysaccharides	glycosyl hydrolase, family 1	-0,6	0,0780253	-0,1	0,8726443	1,2	0,0000927	-0,2	0,5532181	0,0	0,9996125
	EF1244		Unknown function	Enzymes of unknown specificity	oxidoreductase, Gfo/Idh/MocA family	-0,9	0,0153144	0,4	0,3653839	1,2	0,0001076	-0,3	0,3256381	0,2	0,5685966

Operon[a]	Locus	Gene	Functional category	Subcategory	Putative function	Blood[b]	P-value[c]	YTB_5[b]	P-value[c]	YTB_15[b]	P-value[c]	YTB_30[b]	P-value[c]	YTB_60[b]	P-value[c]
	EF1246		Hypothetical proteins	Conserved	conserved hypothetical protein	NA	NA	-0,5	0,2447821	-0,2	0,5040433	-0,4	0,2743909	0,4	0,2165364
	EF1247		Hypothetical proteins	Conserved	conserved hypothetical protein	-0,5	0,2885644	-0,5	0,3117101	-0,8	0,0148286	0,3	0,4321607	-0,6	0,0638286
	EF1248		Hypothetical proteins		hypothetical protein	-0,2	0,5839825	0,0	0,9845715	0,5	0,1451883	0,0	0,9356316	-0,1	0,7400753
	EF1249		Transport and binding proteins	Other	fibronectin/fibrinogen-binding protein, putative	-1,2	0,0014639	-0,4	0,4267189	0,0	0,9601007	0,0	0,9224028	-0,2	0,6370598
	EF1250		Hypothetical proteins		hypothetical protein	-0,3	0,4667885	-0,1	0,8212465	-0,2	0,5058375	0,3	0,4138477	-0,2	0,4635241
	EF1251		Hypothetical proteins		hypothetical protein	NA	NA	NA	NA	NA	NA	NA	NA	NA	NA
	EF1253		Hypothetical proteins	Conserved	conserved hypothetical protein	-0,1	0,8726025	0,1	0,7792562	-0,4	0,1856043	0,1	0,7237228	0,7	0,0280805
	EF1254		Transport and binding proteins	Unknown substrate	ABC transporter, permease protein	0,0	0,9880843	-0,1	0,771608	-0,2	0,5625852	-0,1	0,8434054	0,7	0,0491526
	EF1255		Transport and binding proteins	Unknown substrate	ABC transporter, ATP-binding protein	-0,5	0,163001	0,1	0,7829759	-0,2	0,559296	-0,4	0,2848461	0,4	0,2791896
	EF1258		Hypothetical proteins		hypothetical protein	NA	NA	NA	NA	NA	NA	NA	NA	NA	NA
	EF1259		Unknown function	Enzymes of unknown specificity	hydrolase, haloacid dehalogenase-like family	-0,3	0,3839143	-0,2	0,6867874	-0,1	0,7436276	0,0	0,9548629	0,1	0,6966093
	EF1260		Regulatory functions	DNA interactions	DNA-binding response regulator	0,4	0,2312035	-0,3	0,550602	-0,3	0,3099743	0,2	0,6518152	0,1	0,8800361
	EF1261		Signal transduction	Two-component systems	sensor histidine kinase	-0,6	0,2076123	-0,2	0,619418	-0,4	0,2114664	0,1	0,7818822	-0,3	0,3344521
	EF1262		Hypothetical proteins		hypothetical protein	0,6	0,0964812	0,4	0,3468214	-0,2	0,4292817	0,4	0,2867841	-0,4	0,1848609
	EF1263		Hypothetical proteins		hypothetical protein	NA	NA	0,2	0,7214783	0,1	0,7635556	0,5	0,142515	-0,1	0,7962317
	EF1264		Unknown function	General	sulfatase domain protein	-1,8	**0,000001**	-0,7	0,1190968	-0,3	0,4219921	-0,1	0,6591441	-0,2	0,4847052
	EF1265		Hypothetical proteins	Conserved	conserved hypothetical protein	-0,8	0,0336285	-0,9	0,041026	0,0	0,9482218	0,3	0,2985402	0,2	0,5609352
	EF1266		Hypothetical proteins		hypothetical protein	NA	NA	NA	NA	NA	NA	NA	NA	NA	NA
	EF1267	*rnhC*	Transcription	Degradation of RNA	ribonuclease HIII	NA	NA	0,2	0,6179512	NA	NA	0,4	0,2125703	0,1	0,8774987
	EF1268		Transport and binding proteins	Cations and iron carrying compounds	cation-transporting ATPase, E1-E2 family	NA	NA	0,0	0,9580925	NA	NA	NA	NA	-0,3	0,415376
	EF1269		Cell envelope	Other	cell wall surface anchor family protein	-0,6	0,1052575	0,2	0,6189238	-0,5	0,092199	-0,1	0,6716498	-0,1	0,696372
	EF1270		Hypothetical proteins	Conserved	conserved hypothetical protein	-1,8	**0,0000017**	-0,7	0,1105294	-0,1	0,8052761	0,1	0,6667547	0,3	0,3727423
	EF1271	*nusA*	Transcription	Transcription factors	N utilization substance protein A	-1,7	**0,0000044**	-0,8	0,0767353	-0,3	0,4150784	-0,5	0,1302526	0,0	0,8872248
	EF1272		Hypothetical proteins	Conserved	conserved hypothetical protein	-1,0	0,0057369	-0,5	0,2956721	-0,5	0,1522911	-0,7	0,0406066	0,3	0,4067453
	EF1273		Protein synthesis	Ribosomal proteins: synthesis and modification	ribosomal protein L7A family	-0,4	0,3021743	-0,8	0,0931952	0,0	0,9588513	-0,2	0,4896172	0,0	0,8986536
	EF1274	*infB*	Protein synthesis	Translation factors	translation initiation factor IF-2	-2,7	**0**	-0,4	0,3792067	-0,1	0,7636498	-1,0	0,0021518	0,3	0,4328925
	EF1275	*rbfA*	Transcription	RNA processing	ribosome-binding factor A	-1,2	0,0064078	-0,3	0,5494418	0,2	0,5181689	-0,4	0,2384769	0,1	0,7918013
	EF1276		Hypothetical proteins	Conserved	conserved hypothetical protein	NA	NA	0,0	0,9393119	NA	NA	0,1	0,7583424	0,1	0,6546945
	EF1277		Regulatory functions	DNA interactions	transcriptional regulator, Cro/CI family	-0,3	0,4604346	0,1	0,8566488	0,4	0,1851612	0,1	0,6720061	-0,1	0,742241
	EF1278		Hypothetical proteins		hypothetical protein	NA	NA	0,1	0,8630241	-0,1	0,757286	-0,1	0,7559396	0,1	0,7368395
	EF1279		Mobile and extrachromosomal element functions	Prophage functions	DNA replication protein, putative	0,0	0,9416208	-0,1	0,7831954	-0,1	0,8681857	-0,1	0,7826124	0,2	0,6341789
	EF1280		Mobile and extrachromosomal element functions	Prophage functions	DNA replication protein, putative	NA	NA	0,0	0,9957382	0,1	0,7462023	0,4	0,2489902	-0,1	0,7620667
	EF1281		Hypothetical proteins		hypothetical protein	NA	NA	NA	NA	NA	NA	NA	NA	NA	NA
	EF1282		Hypothetical proteins		hypothetical protein	0,1	0,8896022	-1,1	0,0172399	1,2	0,0001351	0,8	0,0160522	-0,4	0,2380429
	EF1283		Regulatory functions	DNA interactions	transcriptional regulator, RinA family	-0,2	0,6155515	-0,6	0,2225552	0,0	0,9539554	-0,4	0,2403474	-0,5	0,1018122
	EF1284		Mobile and extrachromosomal element functions	Prophage functions	structural protein, putative	0,5	0,2497148	0,0	0,9676754	-0,1	0,8212714	0,0	0,9870093	0,2	0,6059219
	EF1285		Mobile and extrachromosomal element functions	Prophage functions	major tail protein	0,7	0,0971984	0,4	0,4361234	0,0	0,9238031	0,1	0,6619332	-0,4	0,20405
	EF1286		Hypothetical proteins	Conserved	conserved hypothetical protein	NA	NA	0,3	0,5735806	0,2	0,5234095	0,0	0,9287056	-0,3	0,4323577
	EF1287		Hypothetical proteins	Conserved	conserved hypothetical protein	NA	NA	NA	NA	NA	NA	NA	NA	NA	NA
	EF1288		Hypothetical proteins	Conserved	conserved hypothetical protein	NA	NA	0,2	0,6306097	NA	NA	0,3	0,3997158	0,0	0,9253555
	EF1289		Mobile and extrachromosomal element functions	Prophage functions	tail protein, putative	0,1	0,7423257	NA	NA	NA	NA	NA	NA	0,0	0,9888983
	EF1290		Mobile and extrachromosomal element functions	Prophage functions	structural protein, putative	1,0	0,0317476	0,0	0,9914623	-0,6	0,138935	-0,1	0,8195019	0,1	0,6854206
	EF1291		Hypothetical proteins		hypothetical protein	NA	NA	0,6	0,302486	NA	NA	0,0	0,9671879	-0,2	0,6278352
	EF1292		Mobile and extrachromosomal element functions	Prophage functions	holin, putative	NA	NA	NA	NA	NA	NA	NA	NA	-0,2	0,5889969
	EF1293	*ply-1*	Mobile and extrachromosomal element functions	Prophage functions	endolysin	NA	NA	NA	NA	NA	NA	NA	NA	NA	NA
	EF1294	*truB*	Protein synthesis	tRNA and rRNA base modification	tRNA pseudouridine synthase B	-2,9	**0**	-1,6	0,000721	0,1	0,7627713	0,2	0,5291831	-0,3	0,3300001
	EF1295	*ribF*	Biosynthesis of cofactors, prosthetic groups, carriers	Riboflavin, FMN, and FAD	riboflavin biosynthesis protein RibF	-1,7	**0,0000154**	-1,1	0,0205117	-0,3	0,3192225	-0,3	0,4206082	-0,1	0,6718019
	EF1296		Unknown function	Enzymes of unknown specificity	acetyltransferase, GNAT family	-1,0	0,0041371	-0,7	0,1538743	-0,5	0,1510916	-0,5	0,1733123	0,0	0,9879965
	EF1297		Regulatory functions	DNA interactions	transcriptional regulator, PadR family	NA	NA	NA	NA	NA	NA	NA	NA	NA	NA
	EF1298		Hypothetical proteins	Conserved	conserved hypothetical protein	NA	NA	0,3	0,4834277	0,0	0,9920995	-0,1	0,7750378	-0,2	0,4943662
	EF1299		Hypothetical proteins	Conserved	conserved hypothetical protein	NA	NA	0,1	0,8568615	0,9	0,0225929	0,0	0,9348803	-0,3	0,3648404
	EF1300		Cellular processes	Cell division	cell division protein, FtsW/RodA/SpovE family	-1,0	0,0086239	-0,7	0,1388925	0,1	0,6448334	-0,3	0,378402	-0,3	0,4385082
	EF1301		Cellular processes	Cell division	cell division protein, FtsW/RodA/SpovE family	0,5	0,192874	-0,4	0,4385817	-0,1	0,7534745	-0,1	0,7109117	0,0	0,9290221
	EF1302		Regulatory functions	Other	transcriptional regulator, putative	0,0	0,9017574	-0,5	0,32694	0,0	0,9570843	0,0	0,9469836	-0,6	0,0581265
	EF1303		Regulatory functions	DNA interactions	transcriptional regulator, LysR family	0,4	0,4355029	0,3	0,5264321	0,2	0,4489362	0,0	0,9477976	-0,2	0,5492446
	EF1304		Transport and binding proteins	Cations and iron carrying compounds	magnesium-translocating P-type ATPase	NA	NA	NA	NA	NA	NA	NA	NA	NA	NA
	EF1305		Biosynthesis of cofactors, prosthetic groups, carriers	Heme, porphyrin, and cobalamin	oxygen-independent coproporphyrinogen III oxidase, putative	NA	NA	-1,0	0,0232925	0,0	0,9265071	0,6	0,0892502	-0,4	0,2888259
	EF1306	*hrcA*	Regulatory functions	DNA interactions	heat-inducible transcription repressor HrcA	-0,8	0,0267337	-0,6	0,2311675	-0,8	0,01122	-0,7	0,0262322	-1,1	0,0010358
	EF1307	*grpE*	Protein fate	Protein folding and stabilization	heat shock protein GrpE	-1,0	0,0073227	-0,5	0,3278023	-1,1	0,0006845	-0,7	0,0292982	-1,1	0,0006228
	EF1308	*dnaK*	Protein fate	Protein folding and stabilization	dnak protein	-1,0	0,0091788	-0,5	0,2941696	-1,1	0,0007793	-0,8	0,0118925	-1,1	0,0005987
	EF1309		Hypothetical proteins		hypothetical protein	NA	NA	NA	NA	NA	NA	NA	NA	NA	NA
	EF1310	*dnaJ*	Protein fate	Protein folding and stabilization	dnaJ protein	-0,9	0,0182023	-0,3	0,5399922	-0,6	0,0733692	-0,5	0,1018494	-0,3	0,2928004
	EF1311		Hypothetical proteins	Conserved	conserved hypothetical protein	-0,7	0,0691197	-0,5	0,3176463	0,1	0,7630646	-0,3	0,457104	0,2	0,5739103
	EF1312		Unknown function	General	S1 RNA binding domain protein	-1,8	**0,0000013**	-0,6	0,186983	0,3	0,3035075	-0,2	0,5524306	0,4	0,2689087
	EF1313		Hypothetical proteins	Conserved	conserved hypothetical protein	0,1	0,7190167	-0,6	0,2199777	0,0	0,9092346	0,1	0,7109302	0,0	0,9412282
	EF1314		Amino acid biosynthesis	Aspartate family	aspartate aminotransferase, putative	-0,6	0,1443894	-0,5	0,2992059	0,2	0,5697471	0,0	0,9787155	0,0	0,9116312
	EF1315		Hypothetical proteins		hypothetical protein	-0,4	0,3564963	NA	NA	0,0	0,954902	-0,2	0,6805864	0,6	0,0894632
	EF1316		Regulatory functions	DNA interactions	transcriptional regulator, Cro/CI family	-0,3	0,4526224	-0,3	0,5361281	0,0	0,9805969	-0,5	0,1617088	-0,2	0,6147625
	EF1317	*nagA-1*	Central intermediary metabolism	Amino sugars	N-acetylglucosamine-6-phosphate deacetylase	NA	NA	NA	NA	NA	NA	NA	NA	NA	NA
	EF1318		Hypothetical proteins		hypothetical protein	NA	NA	-0,2	0,6297041	NA	NA	-0,1	0,7683943	0,0	0,9859393
	EF1319		Hypothetical proteins	Domain	conserved domain protein	-0,2	0,5352163	-0,8	0,0679232	0,0	0,9453023	0,0	0,9785428	0,0	0,968397
	EF1320		Transport and binding proteins	Unknown substrate	ABC transporter, ATP-binding protein	0,1	0,7256625	0,1	0,8113529	0,2	0,5339249	0,4	0,2575633	0,1	0,6725318
	EF1321		Transport and binding proteins	Unknown substrate	permease domain protein	1,0	0,0059961	0,1	0,9103176	0,2	0,5997053	-0,1	0,6909443	-0,1	0,6569741
	EF1322		Hypothetical proteins	Conserved	conserved hypothetical protein	2,4	**0**	1,9	0,0000393	0,0	0,9963536	0,5	0,1643952	0,5	0,1166301
	EF1324		Hypothetical proteins	Conserved	conserved hypothetical protein	NA	NA	-0,2	0,7340629	-0,2	0,6197649	0,5	0,2085331	0,6	0,0933894
	EF1325		Hypothetical proteins		hypothetical protein	0,8	0,0308438	0,0	0,9934377	0,1	0,8658143	0,3	0,4365894	0,3	0,4470996
	EF1326		Regulatory functions	DNA interactions	transcriptional regulator, TetR family	-0,5	0,2004246	-0,4	0,4457639	-1,3	0,0000517	-0,8	0,0166113	-0,1	0,8266955
	EF1327		Unknown function	General	BadF/BadG/BcrA/BcrD ATPase family protein	-0,8	0,0281381	-0,9	0,041211	-1,9	**0**	-1,7	**0,0000003**	-0,5	0,1637082
	EF1328		Regulatory functions	DNA interactions	transcriptional regulator, GntR family	NA	NA	0,0	0,9934966	NA	NA	-0,3	0,3303795	0,2	0,5023721
	EF1329		Unknown function	General	HesA/MoeB/ThiF family protein	NA	NA	0,5	0,416787	NA	NA	1,4	0,000049	0,0	0,9345794
	EF1330		Hypothetical proteins		hypothetical protein	NA	NA	0,8	0,1036353	NA	NA	NA	NA	0,0	0,9153186
	EF1331		Transport and binding proteins	Unknown substrate	ABC transporter, ATP-binding protein	NA	NA	0,3	0,5765579	NA	NA	NA	NA	-0,6	0,1170116
	EF1332		Cell envelope	Other	membrane protein, putative	0,4	0,326445	-0,2	0,7063544	-0,3	0,4138706	NA	NA	-0,3	0,3450109

Operon[a]	Locus	Gene	Functional category	Subcategory	Putative function	Blood[b]	P-value[c]	YTB_5[b]	P-value[c]	YTB_15[b]	P-value[c]	YTB_30[b]	P-value[c]	YTB_60[b]	P-value[c]
	EF1333		Transport and binding proteins	Unknown substrate	ABC transporter, ATP-binding protein	NA	NA	-0,4	0,3623527	NA	NA	0,9	0,006282	0,0	0,9081874
	EF1334		Unknown function	General	AgrC domain protein	NA	NA	NA	NA	NA	NA	NA	NA	NA	NA
	EF1335		Signal transduction	Two-component systems	sensor histidine kinase, putative	NA	NA	0,4	0,4898603	NA	NA	NA	NA	0,4	0,2697079
	EF1336		Signal transduction	Two-component systems	response regulator	NA	NA	0,7	0,122277	0,3	0,4234193	0,5	0,1683564	0,8	0,0217076
	EF1337		Hypothetical proteins	Conserved	conserved hypothetical protein	NA	NA	0,5	0,3286453	NA	NA	NA	NA	0,1	0,8641662
	EF1338	*trxB*	Energy metabolism	Electron transport	thioredoxin reductase	0,1	0,8159546	-0,5	0,2950117	0,3	0,3247483	0,2	0,6483948	-0,1	0,7835087
	EF1339		Hypothetical proteins	Conserved	conserved hypothetical protein	0,5	0,2145588	-0,3	0,5424635	0,1	0,6931263	0,3	0,4287619	-0,1	0,8629425
	EF1340		Cellular processes	Other	pheromone cAM373 precursor lipoprotein	-0,7	0,1052366	-0,5	0,3135425	0,4	0,1783595	0,5	0,1717136	-0,1	0,8078603
	EF1341		Transport and binding proteins	Unknown substrate	ABC transporter, ATP-binding/permease protein	-1,7	0,0001063	-0,4	0,3658701	-0,7	0,0180738	-0,7	0,0487224	0,0	0,9362648
	EF1342		Regulatory functions	DNA interactions	transcriptional regulator, Mar family	NA	NA	-0,1	0,7699884	0,0	0,9739278	0,5	0,1506335	0,0	0,9316199
	EF1343		Transport and binding proteins	Carbohydrates, organic alcohols, and acids	sugar ABC transporter, permease protein	3,8	**0**	1,1	0,0467925	1,1	0,0059509	NA	NA	0,3	0,3073518
	EF1344		Transport and binding proteins	Carbohydrates, organic alcohols, and acids	sugar ABC transporter, permease protein	3,3	**0**	1,1	0,0183753	0,4	0,2382131	0,0	0,9590825	-0,4	0,2643947
	EF1345		Transport and binding proteins	Carbohydrates, organic alcohols, and acids	sugar ABC transporter, sugar-binding protein	2,8	**0**	1,2	0,0084106	0,5	0,1498409	0,5	0,1068996	-0,5	0,1554132
	EF1346		Hypothetical proteins		hypothetical protein	NA	NA	NA	NA	NA	NA	NA	NA	NA	NA
	EF1347		Energy metabolism	Biosynthesis and degradation of polysaccharides	glycosyl hydrolase, family 13	1,1	0,0115446	0,3	0,5626119	0,3	0,3276047	0,0	0,9420929	-0,3	0,3614847
	EF1348		Energy metabolism	Biosynthesis and degradation of polysaccharides	glucan 1,6-alpha-glucosidase, putative	0,6	0,1235883	0,3	0,5032139	0,2	0,4540972	0,1	0,8630698	-0,4	0,2038223
	EF1349		Energy metabolism	Biosynthesis and degradation of polysaccharides	glycosyl hydrolase, family 13	2,0	**0**	0,6	0,1763182	0,3	0,4193664	0,3	0,3928018	-0,1	0,6545445
	EF1350		Hypothetical proteins	Conserved	conserved hypothetical protein	1,1	0,0024505	0,4	0,3887447	0,1	0,8253072	0,1	0,7307478	0,0	0,9958893
	EF1351		Hypothetical proteins		hypothetical protein	1,7	0,0001778	0,4	0,3464536	0,0	0,8758883	0,8	0,0149559	1,1	0,0010316
	EF1352		Transport and binding proteins	Cations and iron carrying compounds	magnesium-translocating P-type ATPase	0,7	0,0773156	1,0	0,0247244	-0,2	0,5837756	0,7	0,0278245	0,9	0,005635
	EF1353	*pdhA*	Energy metabolism	Pyruvate dehydrogenase	pyruvate dehydrogenase complex E1 component, alpha subunit	1,7	**0,0000037**	0,5	0,3154205	1,5	**0,0000017**	1,0	0,0020571	0,0	0,925497
	EF1354	*pdhB*	Energy metabolism	Pyruvate dehydrogenase	pyruvate dehydrogenase complex, E1 component, beta subunit	2,0	**0,0000001**	0,6	0,1841787	1,5	**0,0000025**	1,2	0,000396	0,2	0,5859683
	EF1355	*aceF*	Energy metabolism	Pyruvate dehydrogenase	pyruvate dehydrogenase E2, dihydrolipoamide acetyltransferase	1,8	**0,0000005**	0,3	0,4738007	1,6	**0,0000007**	1,2	0,0002631	0,1	0,7694599
	EF1356	*lpdA*	Energy metabolism	Pyruvate dehydrogenase	pyruvate dehydrogenase E3, dihydrolipoamide dehydrogenase	1,9	**0,0000004**	0,4	0,3519261	1,5	**0,0000013**	1,3	0,0000921	0,1	0,6876223
	EF1357		Regulatory functions	DNA interactions	transcriptional regulator, AraC family	NA	NA	NA	NA	NA	NA	NA	NA	NA	NA
	EF1358		Central intermediary metabolism	Other	glycerol dehydrogenase, putative	0,7	0,0447228	0,9	0,0544834	0,0	0,968705	-0,9	0,0047983	-0,9	0,0075805
	EF1359		Hypothetical proteins	Conserved	conserved hypothetical protein	0,6	0,0906976	0,8	0,0842187	0,1	0,8738119	-0,8	0,019428	-1,0	0,0029723
	EF1360		Unknown function	Enzymes of unknown specificity	dihydroxyacetone kinase family protein	1,2	0,0008335	0,9	0,0547838	0,0	0,9976371	-0,7	0,0286955	-0,8	0,0488173
	EF1361		Unknown function	Enzymes of unknown specificity	dihydroxyacetone kinase family protein	1,8	**0,0000011**	0,9	0,043801	0,1	0,6600293	-0,5	0,1532787	-0,8	0,0125168
	EF1362		Hypothetical proteins	Domain	conserved domain protein	1,3	0,0005007	-0,1	0,7940735	-0,1	0,6932744	-0,3	0,4364614	-0,2	0,4720174
	EF1363		Central intermediary metabolism	Other	hydroxymethylglutaryl-CoA synthase	-1,0	0,0064208	-0,9	0,0531919	-0,3	0,4240887	-0,5	0,1678014	-0,3	0,2996042
	EF1364		Central intermediary metabolism	Other	acetyl-CoA acetyltransferase/hydroxymethylglutaryl-CoA reductase	-1,3	0,0048163	-0,5	0,2447546	0,0	0,8968618	-0,3	0,3187569	0,0	0,9044407
	EF1365		Hypothetical proteins	Conserved	conserved hypothetical protein	-1,0	0,0195163	-0,5	0,2915897	0,2	0,5980129	0,0	0,9125356	0,3	0,4337469
	EF1366		Hypothetical proteins	Conserved	conserved hypothetical protein	-0,7	0,0571874	-0,4	0,3842159	0,1	0,8413504	0,2	0,4795599	0,4	0,2584496
	EF1367		Cellular processes	Adaptations to atypical conditions	cold-shock domain family protein	-0,5	0,1427043	-0,4	0,4483174	0,0	0,9008291	-0,1	0,8260365	0,7	0,042448
	EF1368		Hypothetical proteins	Conserved	conserved hypothetical protein	3,3	**0**	1,9	0,0000312	0,6	0,0498514	0,9	0,0053138	-0,2	0,6057299
	EF1369		Regulatory functions	DNA interactions	transcriptional regulator, Cro/CI family	0,2	0,6661253	-0,3	0,4975074	-0,2	0,6120802	-0,1	0,7556984	0,0	0,93583
	EF1370		Transport and binding proteins	Other	drug resistance transporter, EmrB/QacA family protein	-1,6	0,0002427	-0,5	0,3096591	-0,1	0,8284777	-0,1	0,6696092	-0,2	0,5851833
	EF1371		Hypothetical proteins	Conserved	conserved hypothetical protein	NA	NA	-0,6	0,1664227	0,1	0,7234172	-0,2	0,575105	0,0	0,8983126
	EF1372		Unknown function	General	CBS domain protein	-0,7	0,0721185	-0,4	0,415261	0,0	0,8970538	-0,2	0,5354518	0,1	0,8545341
	EF1373		Unknown function	Enzymes of unknown specificity	DHH family protein	-0,1	0,7883607	0,1	0,9118791	0,0	0,9485046	-0,2	0,5265203	0,1	0,8319066
	EF1374	*phnA*	Unknown function	General	phnA protein	-0,6	0,1093836	-0,6	0,194486	-0,4	0,1778069	-0,3	0,3176722	0,2	0,6002135
	EF1375		Hypothetical proteins		hypothetical protein	0,7	0,1293571	-0,3	0,5348388	-0,5	0,1292704	-0,3	0,3499144	0,2	0,488672
	EF1376		Hypothetical proteins	Conserved	conserved hypothetical protein	-1,4	0,0001637	-0,4	0,3439902	-0,2	0,5772617	-0,4	0,1800383	0,0	0,9616044
	EF1377		Transcription	Other	ATP-dependent RNA helicase, DEAD/DEAH box family	-1,4	0,0001399	-0,8	0,0696553	0,2	0,6029202	-0,1	0,8489923	0,0	0,9490183
	EF1378		Regulatory functions	Other	transcriptional regulator, putative	NA	NA	-0,1	0,7559716	NA	NA	0,3	0,384083	0,2	0,6439085
	EF1379	*alaS*	Protein synthesis	tRNA aminoacylation	alanyl-tRNA synthetase	-1,3	0,0002572	-0,5	0,2627713	-0,1	0,672772	-0,3	0,4460581	0,1	0,6991335
	EF1380		Hypothetical proteins	Conserved	conserved hypothetical protein	-1,3	0,0005219	-0,6	0,1958261	-0,3	0,3718255	-0,2	0,5678758	0,2	0,5401088
	EF1381		Hypothetical proteins	Conserved	conserved hypothetical protein TIGR00486	-1,3	0,0005733	-0,4	0,3383925	-0,1	0,8664548	-0,3	0,3079158	-0,1	0,8468952
	EF1382	*pepT-1*	Protein fate	Degradation of proteins, peptides, and glycopeptides	peptidase T	-0,8	0,0299903	-0,3	0,5867546	0,1	0,8462052	-0,1	0,6901168	0,0	0,9489047
	EF1383		Cell envelope	Other	membrane protein, putative	-1,7	0,0001774	-1,1	0,0181327	0,1	0,7348568	-0,2	0,5268078	0,1	0,8367081
	EF1384		Cell envelope	Other	membrane protein, putative	-2,1	**0**	-0,9	0,040842	-0,1	0,7919387	-0,4	0,242881	-0,3	0,3579002
	EF1385		Biosynthesis of cofactors, prosthetic groups, carriers	Molybdopterin	molybdopterin-guanine dinucleotide biosynthesis protein A, putative	NA	NA	0,1	0,8309031	NA	NA	NA	NA	0,2	0,6315087
	EF1386		Transport and binding proteins	Carbohydrates, organic alcohols, and acids	formate/nitrite transporter family protein	NA	NA	-0,4	0,4870006	NA	NA	NA	NA	0,1	0,8046938
	EF1387		Hypothetical proteins	Conserved	conserved hypothetical protein	NA	NA	-0,2	0,7643132	NA	NA	NA	NA	0,1	0,7884664
	EF1388		Energy metabolism	Electron transport	NAD-dependent formate dehydrogenase, gamma subunit, putative	NA	NA	0,2	0,6894721	NA	NA	-0,2	0,6207241	0,0	0,9069903
	EF1389		Energy metabolism	Electron transport	NAD-dependent formate dehydrogenase, beta subunit, putative	NA	NA	NA	NA	NA	NA	NA	NA	NA	NA
	EF1390	*fdhA*	Energy metabolism	Electron transport	NAD-dependent formate dehydrogenase, alpha subunit	NA	NA	0,7	0,2063372	NA	NA	NA	NA	0,0	0,9404462
	EF1391		Biosynthesis of cofactors, prosthetic groups, carriers	Molybdopterin	molybdenum cofactor biosynthesis family protein	1,4	0,001993	0,6	0,3215069	NA	NA	0,6	0,0620665	0,0	0,918148
	EF1392	*moaC*	Biosynthesis of cofactors, prosthetic groups, carriers	Molybdopterin	molybdenum cofactor biosynthesis protein MoaC	-0,1	0,7870902	-0,2	0,6942143	-0,7	0,0625602	-0,7	0,102234	0,0	0,9572296
	EF1393		Biosynthesis of cofactors, prosthetic groups, carriers	Molybdopterin	molybdopterin cofactor biosynthesis protein A, putative	0,0	0,9936185	-0,1	0,8419441	-0,6	0,0401282	-0,7	0,0392178	-0,2	0,5883143
	EF1394		Hypothetical proteins	Conserved	conserved hypothetical protein	NA	NA	0,3	0,4909043	-0,4	0,2629944	-0,5	0,1230112	0,1	0,7522192
	EF1395		Biosynthesis of cofactors, prosthetic groups, carriers	Molybdopterin	molybdenum cofactor biosynthesis family protein	NA	NA	0,1	0,7857488	-0,5	0,0911128	-0,8	0,02135	-0,3	0,3826261
	EF1396		Biosynthesis of cofactors, prosthetic groups, carriers	Molybdopterin	molybdenum cofactor biosynthesis family protein, putative	0,2	0,5490754	-0,1	0,8585335	-0,3	0,4253243	-0,6	0,0706083	0,0	0,9066371
	EF1397		Transport and binding proteins	Cations and iron carrying compounds	molybdenum ABC transporter, molybdenum-binding protein	0,9	0,0197547	0,2	0,7216152	-0,4	0,2095539	-0,7	0,0303815	-0,2	0,5088475
	EF1398		Transport and binding proteins	Cations and iron carrying compounds	molybdenum ABC transporter, permease protein	0,9	0,0187186	0,4	0,403528	-0,4	0,2395946	-1,3	0,0000924	-0,6	0,059534
	EF1399		Transport and binding proteins	Cations and iron carrying compounds	molybdenum ABC transporter, ATP-binding protein, putative	0,6	0,1484847	0,1	0,8116039	-0,3	0,4133789	-1,4	0,0000401	-0,5	0,1407619
	EF1400		Transport and binding proteins	Cations and iron carrying compounds	cadmium-translocating P-type ATPase	0,2	0,6072937	-0,4	0,3959196	-0,7	0,0393594	-1,4	0,0000298	-0,3	0,318791
	EF1401		Hypothetical proteins		hypothetical protein	NA	NA	0,3	0,6392105	NA	NA	0,1	0,7697839	-0,1	0,8363134
	EF1402		Hypothetical proteins	Domain	conserved domain protein	0,2	0,6471272	-0,7	0,1148662	0,3	0,3667229	0,3	0,3207649	0,1	0,6820913
	EF1403		Hypothetical proteins	Conserved	conserved hypothetical protein	0,0	0,9020021	-0,4	0,4056368	-0,1	0,8267854	0,1	0,6586549	0,0	0,9791212
	EF1404		DNA metabolism	Other	MutS2 family protein	-0,1	0,7772309	-0,1	0,8621458	0,0	0,916514	-0,3	0,4515176	0,2	0,5132048
	EF1405	*trx*	Energy metabolism	Electron transport	thioredoxin	1,3	0,0003175	0,2	0,6773988	0,6	0,0564649	0,2	0,5839841	0,0	0,9971123
	EF1406	*uvrC*	DNA metabolism	DNA replication, recombination, and repair	excinuclease ABC, subunit C	-1,4	0,0019531	-0,3	0,5060108	-0,1	0,8058333	-0,2	0,63919	0,0	0,9206892
	EF1407		Hypothetical proteins		hypothetical protein	1,3	0,0009135	NA	NA	NA	NA	NA	NA	0,2	0,5275976
	EF1408		Transport and binding proteins	Unknown substrate	ABC transporter, ATP-binding protein	0,5	0,1412834	0,0	0,9228192	0,4	0,1764592	0,3	0,4246845	0,5	0,1296693
	EF1409		Hypothetical proteins	Conserved	conserved hypothetical protein	0,3	0,4454275	0,0	0,9678388	0,2	0,4958608	0,0	0,9996158	0,3	0,4177816
	EF1410		Regulatory functions	DNA interactions	sugar-binding transcriptional regulator, LacI family	NA	NA	-0,2	0,7241216	-0,4	0,266312	0,0	0,9464543	0,2	0,4818173
	EF1411		Energy metabolism	Biosynthesis and degradation of polysaccharides	glycosyl hydrolase, family 4	NA	NA	0,5	0,3133472	NA	NA	0,1	0,7709754	0,0	0,9654221
	EF1412		Hypothetical proteins	Domain	conserved domain protein	0,1	0,818095	2,0	**0,0000132**	-0,3	0,5128584	0,2	0,6231806	0,3	0,3355394
	EF1413		Cellular processes	Toxin production and resistance	msrC protein, putative	NA	NA	NA	NA	NA	NA	NA	NA	0,4	0,2441079
	EF1414		Hypothetical proteins	Conserved	conserved hypothetical protein	-0,8	0,0760183	-0,8	0,0819005	-0,2	0,507935	0,1	0,7654663	-0,2	0,4822408
	EF1415	*gdhA*	Amino acid biosynthesis	Glutamate family	glutamate dehydrogenase	1,1	0,0018853	0,2	0,7144063	0,1	0,6700835	0,2	0,5668346	0,3	0,3930726

Operon[a]	Locus	Gene	Functional category	Subcategory	Putative function	Blood[b]	P-value[c]	YTB_5[b]	P-value[c]	YTB_15[b]	P-value[c]	YTB_30[b]	P-value[c]	YTB_60[b]	P-value[c]
	EF1416	*pgi*	Energy metabolism	Glycolysis/gluconeogenesis	glucose-6-phosphate isomerase	-0,6	0,107667	0,0	0,9915697	-0,1	0,6988664	0,1	0,7520833	0,0	0,9752036
	EF1417		Mobile and extrachromosomal element functions	Prophage functions	site-specific recombinase, phage integrase family	1,4	0,0002219	0,6	0,1760897	0,0	0,8851093	-0,4	0,2486241	-0,5	0,1125539
	EF1418		Hypothetical proteins		hypothetical protein	NA	NA	0,8	0,0970694	-0,3	0,4319072	-0,2	0,6230778	-0,1	0,7026251
	EF1419		Hypothetical proteins	Conserved	conserved hypothetical protein	NA	NA	0,2	0,7304476	NA	NA	-0,1	0,7867285	0,0	0,9807285
	EF1420		Hypothetical proteins		hypothetical protein	-0,4	0,2222619	0,1	0,8718096	0,2	0,5679333	0,1	0,6637839	0,2	0,5264839
	EF1421		Hypothetical proteins	Conserved	conserved hypothetical protein	-0,4	0,3225792	0,5	0,2495277	0,1	0,7395397	-0,1	0,7903258	-0,3	0,4273665
	EF1422		Regulatory functions	DNA interactions	transcriptional regulator, Cro/CI family	-0,1	0,7638408	0,7	0,1415423	0,0	0,9436713	0,0	0,9982446	-0,4	0,2114203
	EF1423		Regulatory functions	DNA interactions	transcriptional regulator, Cro/CI family	1,5	0,0001631	0,0	0,9582427	NA	NA	NA	NA	-0,1	0,7663578
	EF1424		Hypothetical proteins		hypothetical protein	NA	NA	NA	NA	NA	NA	NA	NA	NA	NA
	EF1425		Hypothetical proteins		hypothetical protein	NA	NA	0,2	0,7268628	NA	NA	0,1	0,8799838	-0,2	0,4570385
	EF1426		Cellular processes	Pathogenesis	vrlI protein, putative	NA	NA	NA	NA	NA	NA	NA	NA	NA	NA
	EF1427		Hypothetical proteins		hypothetical protein	NA	NA	NA	NA	NA	NA	NA	NA	NA	NA
	EF1428		Hypothetical proteins		hypothetical protein	1,1	0,0123835	0,3	0,5327318	-0,5	0,1221557	NA	NA	-0,1	0,8383784
	EF1429		Hypothetical proteins		hypothetical protein	NA	NA	NA	NA	NA	NA	NA	NA	NA	NA
	EF1430		Hypothetical proteins	Conserved	conserved hypothetical protein	NA	NA	NA	NA	NA	NA	NA	NA	0,3	0,3811283
	EF1431		Hypothetical proteins		hypothetical protein	NA	NA	0,4	0,3716664	NA	NA	NA	NA	-0,1	0,8691329
	EF1432		Hypothetical proteins		hypothetical protein	1,0	0,0098798	-0,1	0,779556	-0,9	0,0047733	NA	NA	-0,1	0,702165
	EF1433		Hypothetical proteins	Conserved	conserved hypothetical protein	0,9	0,0184479	0,4	0,4288904	-0,4	0,1798595	NA	NA	-0,5	0,1463531
	EF1434		Unknown function	General	DnaD domain protein	0,7	0,0581528	-0,2	0,7160777	-0,6	0,0492363	NA	NA	-0,4	0,2141389
	EF1435		Mobile and extrachromosomal element functions	Prophage functions	recombination protein U, putative	0,9	0,0519723	NA	NA	0,1	0,8147075	NA	NA	0,0	0,9029708
	EF1436		Hypothetical proteins		hypothetical protein	NA	NA	0,2	0,7147056	-0,8	0,0447881	-0,6	0,1772707	0,0	0,9622637
	EF1437		Hypothetical proteins		hypothetical protein	NA	NA	-0,5	0,3797832	NA	NA	NA	NA	0,2	0,5374222
	EF1438		Hypothetical proteins		hypothetical protein	NA	NA	NA	NA	NA	NA	NA	NA	-0,1	0,6879748
	EF1439		Hypothetical proteins		hypothetical protein	1,3	0,0037962	0,3	0,6076796	NA	NA	NA	NA	-0,4	0,2722913
	EF1440		Hypothetical proteins	Conserved	conserved hypothetical protein TIGR01671	1,2	0,0015839	-0,3	0,508406	-0,3	0,2709171	-0,3	0,3156395	-0,4	0,2655177
	EF1441		Hypothetical proteins		hypothetical protein	1,1	0,0107238	0,4	0,4008033	-0,4	0,2061193	NA	NA	-0,9	0,009743
	EF1442		Unknown function	General	DNA topoisomerase domain protein	NA	NA	0,6	0,2232935	-0,5	0,1901595	-0,4	0,2095688	-0,4	0,1870115
	EF1443		Hypothetical proteins	Conserved	conserved hypothetical protein	NA	NA	NA	NA	NA	NA	NA	NA	-0,5	0,154456
	EF1444		Hypothetical proteins	Conserved	conserved hypothetical protein	0,5	0,2781628	0,1	0,8219189	NA	NA	-0,1	0,7672365	-0,2	0,6541766
	EF1445		Unknown function	General	replicase domain protein	NA	NA	0,5	0,2964357	-0,8	0,0129437	-0,3	0,4414688	-0,1	0,8466337
	EF1446		Hypothetical proteins		hypothetical protein	0,9	0,0122788	-0,5	0,3158496	-0,3	0,2719152	-0,2	0,7160181	-0,1	0,696925
	EF1447		Hypothetical proteins	Conserved	conserved hypothetical protein	NA	NA	NA	NA	NA	NA	NA	NA	NA	NA
	EF1448		Hypothetical proteins		hypothetical protein	NA	NA	NA	NA	NA	NA	NA	NA	NA	NA
	EF1449		Hypothetical proteins	Conserved	conserved hypothetical protein	NA	NA	NA	NA	NA	NA	NA	NA	NA	NA
	EF1450		Regulatory functions	DNA interactions	positive control factor, putative	0,3	0,4645957	0,2	0,6621599	NA	NA	0,3	0,4416117	-0,1	0,796968
	EF1451		Hypothetical proteins	Domain	conserved domain protein	NA	NA	0,2	0,6278235	NA	NA	-0,2	0,6264845	0,2	0,6468203
	EF1452		DNA metabolism	Restriction/modification	adenine methyltransferase, putative	1,1	0,0024097	0,2	0,6038326	0,4	0,1649211	0,8	0,0174587	0,0	0,9581593
	EF1453		Hypothetical proteins	Conserved	conserved hypothetical protein	NA	NA	0,7	0,2099799	NA	NA	NA	NA	-0,1	0,7468312
	EF1454		Disrupted reading frame		terminase, small subunit, internal deletion	NA	NA	0,7	0,2200899	NA	NA	NA	NA	-0,2	0,6399967
	EF1455		Mobile and extrachromosomal element functions	Prophage functions	terminase, large subunit, putative	NA	NA	NA	NA	NA	NA	0,1	0,8821957	0,0	0,8831754
	EF1456		Hypothetical proteins	Conserved	conserved hypothetical protein TIGR01555	NA	NA	0,5	0,3482326	NA	NA	0,2	0,6485804	0,0	0,9101419
	EF1457		Mobile and extrachromosomal element functions	Prophage functions	minor head protein	1,0	0,0327326	0,3	0,5436381	-0,6	0,1028896	NA	NA	-0,1	0,8461438
	EF1458		Hypothetical proteins		hypothetical protein	NA	NA	NA	NA	NA	NA	NA	NA	NA	NA
	EF1459		Hypothetical proteins	Conserved	conserved hypothetical protein	NA	NA	NA	NA	NA	NA	NA	NA	-0,2	0,6462584
	EF1460		Unknown function	General	LysM domain protein	NA	NA	NA	NA	NA	NA	NA	NA	NA	NA
	EF1461		Hypothetical proteins	Conserved	conserved hypothetical protein	NA	NA	0,5	0,2575956	NA	NA	-0,2	0,6350277	0,0	0,9674724
	EF1462		Hypothetical proteins	Conserved	conserved hypothetical protein	NA	NA	0,6	0,2590617	NA	NA	NA	NA	0,0	0,9569178
	EF1463		Hypothetical proteins		hypothetical protein	0,5	0,2516699	0,0	0,9704967	NA	NA	NA	NA	0,3	0,3710514
	EF1464		Hypothetical proteins	Conserved	conserved hypothetical protein	0,7	0,0757223	0,6	0,2965404	-0,5	0,1634317	0,4	0,2992127	0,1	0,7574089
	EF1465		Hypothetical proteins	Conserved	conserved hypothetical protein	NA	NA	0,1	0,7970343	NA	NA	NA	NA	-0,4	0,233784
	EF1466		Hypothetical proteins	Conserved	conserved hypothetical protein	NA	NA	0,2	0,759831	NA	NA	NA	NA	0,0	0,8952652
	EF1467		Hypothetical proteins	Conserved	conserved hypothetical protein	NA	NA	NA	NA	NA	NA	NA	NA	0,1	0,8775941
	EF1468		Hypothetical proteins	Conserved	conserved hypothetical protein	NA	NA	NA	NA	NA	NA	NA	NA	NA	NA
	EF1469		Hypothetical proteins	Conserved	conserved hypothetical protein	0,6	0,2178034	0,6	0,2840187	NA	NA	NA	NA	0,2	0,7058396
	EF1470		Hypothetical proteins	Conserved	conserved hypothetical protein	NA	NA	0,5	0,3428119	NA	NA	NA	NA	0,2	0,5294539
	EF1471		Hypothetical proteins	Conserved	conserved hypothetical protein	NA	NA	-0,3	0,6134921	NA	NA	NA	NA	0,0	0,9320855
	EF1472		Hypothetical proteins	Conserved	conserved hypothetical protein	NA	NA	0,1	0,8756475	NA	NA	NA	NA	0,0	0,9316323
	EF1473		Hypothetical proteins	Conserved	conserved hypothetical protein	NA	NA	NA	NA	NA	NA	NA	NA	0,4	0,2556764
	EF1474		Unknown function	General	LysM domain protein	NA	NA	0,1	0,7598599	NA	NA	0,3	0,4532591	0,4	0,2255548
	EF1475		Hypothetical proteins	Conserved	conserved hypothetical protein	NA	NA	NA	NA	NA	NA	NA	NA	NA	NA
	EF1476		Hypothetical proteins	Conserved	conserved hypothetical protein	0,6	0,2027055	0,4	0,501952	NA	NA	NA	NA	0,3	0,2975477
	EF1477		Hypothetical proteins	Conserved	conserved hypothetical protein	NA	NA	-0,1	0,9172613	NA	NA	0,2	0,5037788	-0,1	0,8739708
	EF1478		Hypothetical proteins	Conserved	conserved hypothetical protein	NA	NA	0,7	0,2073174	NA	NA	0,3	0,4294009	-0,1	0,7869985
	EF1479		Hypothetical proteins	Conserved	conserved hypothetical protein	NA	NA	-0,2	0,6352934	NA	NA	-0,1	0,8712372	0,2	0,628933
	EF1480		Hypothetical proteins	Conserved	conserved hypothetical protein	NA	NA	NA	NA	NA	NA	NA	NA	NA	NA
	EF1481		Hypothetical proteins		hypothetical protein	NA	NA	NA	NA	NA	NA	NA	NA	-0,1	0,7505848
	EF1482		Hypothetical proteins		hypothetical protein	NA	NA	0,5	0,335419	-0,7	0,0840348	NA	NA	0,4	0,1829752
	EF1483		Hypothetical proteins	Conserved	conserved hypothetical protein	NA	NA	-0,3	0,6015936	NA	NA	NA	NA	-0,2	0,4970253
	EF1484		Hypothetical proteins	Conserved	conserved hypothetical protein	NA	NA	0,6	0,3260934	NA	NA	NA	NA	NA	NA
	EF1485		Hypothetical proteins	Conserved	conserved hypothetical protein	NA	NA	0,1	0,8995624	NA	NA	NA	NA	NA	NA
	EF1486	*ply-2*	Mobile and extrachromosomal element functions	Prophage functions	endolysin	NA	NA	0,4	0,5028927	NA	NA	0,5	0,1989348	0,4	0,37623
	EF1487		Hypothetical proteins		hypothetical protein	0,2	0,6805295	-0,1	0,8550951	0,4	0,1711792	0,6	0,0543339	0,5	0,1075031
	EF1488		Hypothetical proteins		hypothetical protein	-0,2	0,6598197	0,0	0,9502495	-0,2	0,5503848	0,2	0,4775124	0,6	0,0635879
	EF1489		Hypothetical proteins		hypothetical protein	2,0	**0,0000002**	0,9	0,0634805	0,2	0,5590542	NA	NA	-0,1	0,7353157
	EF1490		Hypothetical proteins		hypothetical protein	NA	NA	-0,1	0,7902449	-0,4	0,1793008	-0,1	0,8505948	0,4	0,2129408
	EF1491		Purines, pyrimidines, nucleosides, and nucleotides	2'-Deoxyribonucleotide metabolism	nrdI family protein	NA	NA	-0,4	0,3648109	-0,9	0,0064311	-0,3	0,3837028	0,2	0,589817
	EF1492		Transport and binding proteins	Cations and iron carrying compounds	V-type ATPase, subunit F	0,5	0,1385241	0,4	0,3336785	0,0	0,9709013	0,4	0,1879571	0,6	0,0961881
	EF1493		Transport and binding proteins	Cations and iron carrying compounds	V-type ATPase, subunit I	0,6	0,1080713	0,4	0,3703924	-0,3	0,3361602	0,1	0,8346535	0,2	0,5584576
	EF1494		Transport and binding proteins	Cations and iron carrying compounds	V-type ATPase, subunit K	0,5	0,1481971	0,4	0,4125863	-0,5	0,1051894	0,0	0,9704612	0,1	0,6606985
	EF1495		Transport and binding proteins	Cations and iron carrying compounds	V-type ATPase, subunit E	0,8	0,0386049	0,3	0,4953956	-0,5	0,0945339	-0,3	0,3725711	0,1	0,6598597
	EF1496		Transport and binding proteins	Cations and iron carrying compounds	V-type ATPase, subunit C	0,5	0,1374149	0,3	0,4822391	-0,6	0,0780476	-0,3	0,3398688	0,3	0,4121209
	EF1497		Transport and binding proteins	Cations and iron carrying compounds	V-type ATPase, subunit G	0,1	0,8750837	0,3	0,5484396	-0,2	0,536944	-0,4	0,272901	0,3	0,3843647
	EF1498		Transport and binding proteins	Cations and iron carrying compounds	V-type ATPase, subunit A	0,5	0,1622396	0,1	0,8596978	-0,5	0,1134775	-0,3	0,4418273	0,1	0,6620195

Operon[a)]	Locus	Gene	Functional category	Subcategory	Putative function	Blood[b)]	P-value[c)]	YTB_5[b)]	P-value[c)]	YTB_15[b)]	P-value[c)]	YTB_30[b)]	P-value[c)]	YTB_60[b)]	P-value[c)]
	EF1499		Transport and binding proteins	Cations and iron carrying compounds	V-type ATPase, subunit B	0,4	0,2327587	0,1	0,8256231	-0,7	0,0194931	-0,6	0,0562773	0,1	0,8531758
	EF1500		Transport and binding proteins	Cations and iron carrying compounds	V-type ATPase, subunit D	0,7	0,0430444	0,1	0,8191844	-0,8	0,0093608	-0,5	0,1737712	0,0	0,9496351
	EF1501		Hypothetical proteins		hypothetical protein	0,0	0,9062998	-0,1	0,8528578	-0,6	0,0548435	-0,6	0,0536139	0,4	0,2389095
	EF1502		Cellular processes	Toxin production and resistance	beta-lactamase, putative	-1,4	0,0015013	0,0	0,916918	0,0	0,9806389	-0,3	0,3907418	0,6	0,0589373
	EF1503		Energy metabolism	Glycolysis/gluconeogenesis	fructose-1,6-bisphosphatase, putative	-1,2	0,0099625	-0,2	0,6643893	-0,5	0,1385695	-0,6	0,081268	0,7	0,0392034
	EF1504	*lysA*	Amino acid biosynthesis	Aspartate family	diaminopimelate decarboxylase	NA	NA	-0,1	0,8499849	0,4	0,2449931	0,1	0,7467519	0,2	0,559528
	EF1505		Hypothetical proteins	Conserved	conserved hypothetical protein	2,2	**0**	0,7	0,141196	0,3	0,3930763	0,7	0,0348903	0,1	0,7139481
	EF1506		Hypothetical proteins		hypothetical protein	-0,3	0,5386888	-0,8	0,086292	-1,0	0,000963	-0,1	0,6643907	0,2	0,4917236
	EF1507		Hypothetical proteins		hypothetical protein	NA	NA	NA	NA	NA	NA	NA	NA	0,1	0,79228
	EF1508		Hypothetical proteins		hypothetical protein	NA	NA	0,5	0,3380738	NA	NA	NA	NA	0,3	0,4345063
	EF1509		Hypothetical proteins	Conserved	conserved hypothetical protein	NA	NA	-0,6	0,2635687	NA	NA	NA	NA	0,4	0,2547264
	EF1510		Hypothetical proteins	Conserved	conserved hypothetical protein	NA	NA	-0,5	0,2877979	-0,2	0,5844761	-0,2	0,4926012	0,2	0,5588844
	EF1511		Energy metabolism	Other	mandelate racemase/muconate lactonizing enzyme family protein	0,8	0,0844243	-0,1	0,8971438	NA	NA	NA	NA	0,5	0,0980138
	EF1512		Hypothetical proteins	Conserved	conserved hypothetical protein	0,2	0,5805294	-0,1	0,8229465	-0,4	0,182374	0,2	0,6009695	0,4	0,2274191
	EF1513		Transport and binding proteins	Amino acids, peptides and amines	pheromone binding protein	NA	NA	0,7	0,1237546	0,0	0,8971851	0,2	0,5476586	-0,2	0,6075706
	EF1515		Regulatory functions	RNA interactions	transcription antiterminator, bglG family	NA	NA	-0,3	0,5765181	0,0	0,9574622	1,2	0,0003525	0,8	0,0190616
	EF1516		Signal transduction	PTS	PTS system, IIABC components	-2,9	**0**	-0,5	0,31931	0,3	0,4234066	0,7	0,0308249	0,3	0,3058371
	EF1517		Hypothetical proteins		hypothetical protein	NA	NA	0,3	0,5436406	NA	NA	NA	NA	-0,2	0,506571
	EF1518		Hypothetical proteins	Conserved	conserved hypothetical protein	0,9	0,0134345	0,0	0,9169055	-0,5	0,1066576	-0,2	0,5515313	-0,2	0,472805
	EF1519		Transport and binding proteins	Cations and iron carrying compounds	cation-transporting ATPase, E1-E2 family	-0,5	0,186192	-1,0	0,0367739	-0,4	0,235522	-0,4	0,2395418	-0,2	0,5381067
	EF1521	*dnaG*	DNA metabolism	DNA replication, recombination, and repair	DNA primase	-0,5	0,1475311	-0,6	0,1905262	0,0	0,9010022	0,3	0,437334	0,4	0,1831526
	EF1522	*sigA*	Transcription	Transcription factors	RNA polymerase sigma-43 factor	-1,8	**0,0000007**	-0,5	0,2855288	0,0	0,9580194	-0,1	0,6698483	0,1	0,8181413
	EF1523		Hypothetical proteins	Domain	conserved domainl protein	NA	NA	NA	NA	NA	NA	NA	NA	NA	NA
	EF1524		Hypothetical proteins	Conserved	conserved hypothetical protein	-0,7	0,0653016	-0,3	0,5546252	-0,2	0,5595118	0,1	0,8762999	-0,2	0,6265159
	EF1525		Regulatory functions	DNA interactions	transcriptional regulator, Fur family	-0,5	0,1648949	-0,4	0,3766709	-0,5	0,1166891	-0,4	0,2893559	0,3	0,3267616
	EF1526	*gap-1*	Energy metabolism	Glycolysis/gluconeogenesis	glyceraldehyde 3-phosphate dehydrogenase	3,1	**0**	0,8	0,0873043	0,4	0,2281501	0,6	0,0759731	0,1	0,6706405
	EF1527		Unknown function	General	GTP-binding protein	-1,6	**0,0000088**	-0,9	0,0472225	-0,3	0,413882	-0,1	0,833805	-0,1	0,6921631
	EF1528		Hypothetical proteins		hypothetical protein	0,8	0,0804541	0,4	0,3896053	-0,2	0,575895	-0,5	0,1719368	-0,6	0,0973401
	EF1529		Signal transduction	PTS	PTS system, IIC component, putative	1,9	**0,0000001**	0,7	0,1358467	-0,1	0,7164401	-0,5	0,1642988	-0,8	0,018897
	EF1531		Regulatory functions	DNA interactions	transcriptional regulator, TetR family	NA	NA	0,3	0,5762192	-0,5	0,2332015	-0,1	0,7868141	0,0	0,8985888
	EF1532		Hypothetical proteins		hypothetical protein	NA	NA	0,4	0,4884671	NA	NA	NA	NA	NA	NA
	EF1533		Hypothetical proteins	Conserved	conserved hypothetical protein	NA	NA	0,3	0,6408134	NA	NA	NA	NA	0,2	0,5871893
	EF1534		Protein fate	Protein folding and stabilization	peptidyl-prolyl cis-trans isomerase, cyclophilin-type	-1,3	0,0043818	-0,3	0,5819779	-0,3	0,3748983	-0,8	0,0236526	0,1	0,7374059
	EF1535		Hypothetical proteins	Conserved	conserved hypothetical protein	1,3	0,0003228	1,8	0,0001304	0,8	0,011274	0,3	0,412468	-0,1	0,7871423
	EF1536		Hypothetical proteins	Conserved	conserved hypothetical protein	0,1	0,7032246	0,1	0,8205663	-0,1	0,6501998	-0,1	0,6801567	0,0	0,9155053
	EF1537		DNA metabolism	DNA replication, recombination, and repair	integrase/recombinase XerD, putative	1,0	0,0049848	-0,1	0,8085221	0,1	0,784002	0,1	0,7074389	0,0	0,9754242
	EF1538	*scpA*	Segregation and condensation protein A	Conserved	conserved hypothetical protein	-1,4	0,0001106	-0,9	0,065816	-0,2	0,484974	0,1	0,730315	-0,1	0,7954467
	EF1539	*scpB*	Segregation and condensation protein B	Conserved	conserved hypothetical protein TIGR00281	0,0	0,9222529	-0,3	0,588724	-0,3	0,4150172	0,2	0,4911008	-0,1	0,6988113
	EF1540	*rluB*	Protein synthesis	tRNA and rRNA base modification	ribosomal large subunit pseudouridine synthase B	-1,6	**0,0000133**	-0,4	0,446897	-0,3	0,3264825	0,1	0,8722079	-0,1	0,8175621
	EF1541		Hypothetical proteins	Conserved	conserved hypothetical protein	0,7	0,0720014	1,5	0,0014217	0,9	0,0055139	1,4	0,0000357	0,0	0,9537047
	EF1542		Hypothetical proteins	Conserved	conserved hypothetical protein	0,4	0,2638406	-0,7	0,1363947	-0,1	0,764248	0,5	0,1136763	0,0	0,9657444
	EF1543	*fer*	Energy metabolism	Electron transport	ferredoxin	NA	NA	0,0	0,9218045	0,1	0,712891	0,4	0,2762923	-0,1	0,7035444
	EF1544		Hypothetical proteins	Conserved	conserved hypothetical protein	-0,3	0,4334561	0,1	0,7544446	-0,1	0,7501691	-0,2	0,4975256	0,2	0,6240176
	EF1545	*recQ-1*	DNA metabolism	DNA replication, recombination, and repair	ATP-dependent DNA helicase RecQ	0,1	0,897469	0,2	0,6851339	0,0	0,9392582	-0,2	0,6436144	0,2	0,6262628
	EF1546		Unknown function	General	LysM domain protein	-0,4	0,3128022	-0,5	0,2469459	-0,1	0,8615131	0,3	0,3629081	-0,2	0,595576
	EF1547	*cmk*	Purines, pyrimidines, nucleosides, and nucleotides	Nucleotide and nucleoside interconversions	cytidylate kinase	-2,5	**0**	-0,6	0,2237736	-0,3	0,2937713	-0,4	0,2410146	-0,2	0,5872898
	EF1548		Protein synthesis	Ribosomal proteins: synthesis and modification	ribosomal protein S1	0,3	0,3414785	0,2	0,6173636	0,0	0,9821453	-0,1	0,7566387	-0,1	0,8623935
	EF1549		Unknown function	General	GTPase, putative	-2,3	**0**	-0,6	0,1678986	-0,2	0,5298088	-0,4	0,2583137	-0,2	0,6116451
	EF1550	*hup*	DNA metabolism	Chromosome-associated proteins	DNA-binding protein HU	0,1	0,8750482	0,3	0,4793888	-0,3	0,3626038	0,3	0,3717088	0,2	0,6425032
	EF1551		Hypothetical proteins		hypothetical protein	NA	NA	NA	NA	NA	NA	NA	NA	NA	NA
	EF1552		Hypothetical proteins		hypothetical protein	1,6	0,0000199	0,6	0,1966349	0,1	0,8470074	0,5	0,178697	0,2	0,5195584
	EF1553		Unknown function	General	TPR domain protein	1,0	0,0044915	0,1	0,8131362	0,1	0,8507827	0,5	0,1448866	0,2	0,6490636
	EF1554		Hypothetical proteins	Conserved	conserved hypothetical protein	1,4	0,000144	0,2	0,6101418	-0,1	0,7449332	0,4	0,1900044	0,0	0,9560242
	EF1555		Unknown function	General	YitT family protein	-1,0	0,0075511	0,2	0,6993407	-0,2	0,464959	0,1	0,6676219	0,0	0,9205806
	EF1556		Hypothetical proteins	Conserved	conserved hypothetical protein	-0,7	0,1321869	0,1	0,7750232	0,1	0,8133652	0,3	0,3905762	0,1	0,7455326
	EF1557	*dapB*	Amino acid biosynthesis	Aspartate family	dihydrodipicolinate reductase	-0,7	0,0423518	-0,2	0,6708398	0,3	0,3622636	0,4	0,2202578	0,0	0,896958
	EF1558	*papS*	Transcription	RNA processing	poly A polymerase	-1,3	0,0035029	-0,2	0,6195491	0,0	0,8779736	0,1	0,8080093	0,1	0,6994545
	EF1559		Hypothetical proteins	Conserved	conserved hypothetical protein	-0,2	0,5042257	-0,2	0,7195026	-0,1	0,7820247	0,2	0,5608617	0,2	0,527729
	EF1560		Hypothetical proteins		hypothetical protein	1,7	**0,0000026**	0,7	0,1406367	0,1	0,6763966	0,7	0,0435617	0,3	0,4356555
	EF1561	*aroE*	Amino acid biosynthesis	Aromatic amino acid family	shikimate 5-dehydrogenase	-2,8	**0**	0,2	0,7419467	-0,1	0,8622498	0,3	0,3236337	0,2	0,544832
	EF1562		Amino acid biosynthesis	Aromatic amino acid family	phospho-2-dehydro-3-deoxyheptonate aldolase, putative	-3,1	**0**	0,2	0,7124047	0,1	0,6519493	0,4	0,2044872	0,4	0,222342
	EF1563	*aroB*	Amino acid biosynthesis	Aromatic amino acid family	3-dehydroquinate synthase	-2,5	**0**	0,0	0,9995545	0,4	0,2091446	0,6	0,0568979	0,6	0,0541368
	EF1564	*aroC*	Amino acid biosynthesis	Aromatic amino acid family	chorismate synthase	-2,2	**0**	-0,2	0,6270995	0,6	0,0835063	0,8	0,0238299	1,0	0,0041083
	EF1565		Amino acid biosynthesis	Aromatic amino acid family	prephenate dehydrogenase	-3,5	**0**	0,1	0,8226242	0,6	0,0434029	0,5	0,120867	1,0	0,0027047
	EF1566	*aroA*	Amino acid biosynthesis	Aromatic amino acid family	3-phosphoshikimate 1-carboxyvinyltransferase	-3,4	**0**	0,1	0,826736	0,6	0,0556322	0,3	0,4161235	1,0	0,0027869
	EF1567	*aroK*	Amino acid biosynthesis	Aromatic amino acid family	shikimate kinase	-2,2	**0**	0,1	0,8064239	0,4	0,2597318	0,0	0,9911193	0,9	0,0068569
	EF1568		Amino acid biosynthesis	Aromatic amino acid family	prephenate dehydratase	-2,7	**0**	0,1	0,8809271	0,6	0,0641699	0,0	0,9866539	1,0	0,0033133
	EF1569	*psr*	Regulatory functions	Other	transcriptional regulator, PSR protein	-1,3	0,0047029	-1,5	0,0009799	-0,2	0,4776749	0,1	0,8618668	-0,1	0,8070568
	EF1570		Unknown function	General	DegV family protein	-3,1	**0**	-1,5	0,0015095	-0,1	0,7374565	0,1	0,8350839	-0,2	0,4627012
	EF1571		Hypothetical proteins		hypothetical protein	0,4	0,2465505	0,6	0,2952907	0,3	0,3624674	0,6	0,0723321	-0,1	0,8201869
	EF1572		Hypothetical proteins		hypothetical protein	0,8	0,080484	0,4	0,4140161	-0,2	0,4853072	0,2	0,4665538	0,2	0,5790739
	EF1573		Hypothetical proteins		hypothetical protein	-0,4	0,3013489	0,0	0,9997755	-0,4	0,1698638	0,4	0,1855129	-0,1	0,6857301
	EF1574		Transport and binding proteins	Cations and iron carrying compounds	Na+/H+ antiporter, putaive	-1,5	0,0006778	-0,5	0,2729071	-0,1	0,816329	-0,3	0,3021806	-0,1	0,8449883
	EF1575		Transport and binding proteins	Unknown substrate	ABC transporter, ATP-binding protein	-1,7	**0,0000021**	-1,0	0,0293092	-0,1	0,6443324	-0,2	0,5737007	-0,1	0,7159736
	EF1576	*thyA*	Purines, pyrimidines, nucleosides, and nucleotides	2'-Deoxyribonucleotide metabolism	thymidylate synthase	-2,4	**0**	-1,0	0,0302166	0,0	0,9012238	0,0	0,9620611	0,0	0,9141896
	EF1577	*folA*	Biosynthesis of cofactors, prosthetic groups, carriers	Folic acid	dihydrofolate reductase	-1,3	0,0002796	-0,7	0,1228874	-0,3	0,4137166	-0,1	0,6622901	-0,1	0,7411624
	EF1578		Energy metabolism	Electron transport	iron-sulfur cluster-binding protein, putative	0,7	0,0614859	0,9	0,0553602	-0,1	0,8431786	0,3	0,421997	0,2	0,5051526
	EF1579	*lexA*	Regulatory functions	DNA interactions	transcriptional repressor LexA	NA	NA	0,4	0,3775962	0,0	0,9852944	0,3	0,3819408	0,1	0,7167148
	EF1580		Hypothetical proteins	Conserved	conserved hypothetical protein	-0,8	0,0272459	-0,3	0,49072	0,0	0,8958933	0,3	0,4450506	-0,2	0,6376897
	EF1582		Transport and binding proteins	Unknown substrate	major facilitator family transporter	-0,4	0,3470608	-0,7	0,1322289	-0,4	0,2902726	NA	NA	0,2	0,5136114
	EF1583		Cell envelope	Biosynthesis/degradation of murein sacculus/peptidoglycan	N-acetylmuramoyl-L-alanine amidase, family 4	-1,5	0,0000501	-1,0	0,0302195	0,2	0,5680655	0,3	0,3428755	-0,4	0,2421103
	EF1584	*cysK*	Amino acid biosynthesis	Serine family	cysteine synthase A	2,8	**0**	0,7	0,1070247	0,3	0,305568	0,8	0,0201314	0,0	0,9022229
	EF1585		Regulatory functions	DNA interactions	transcriptional regulator, Fur family	1,3	0,0002357	0,6	0,2078351	-0,1	0,789425	0,1	0,7618853	-0,1	0,6912688

Operon[a)]	Locus	Gene	Functional category	Subcategory	Putative function	Blood[b)]	P-value[c)]	YTB_5[b)]	P-value[c)]	YTB_15[b)]	P-value[c)]	YTB_30[b)]	P-value[c)]	YTB_60[b)]	P-value[c)]
	EF1586	*nox*	Energy metabolism	Electron transport	NADH oxidase	-2,4	**0**	-0,4	0,4431235	-0,4	0,2158211	-1,1	0,001473	-0,6	0,0803325
	EF1587		DNA metabolism	DNA replication, recombination, and repair	MutT/nudix family protein	NA	NA	-0,4	0,3667475	0,0	0,9552637	-0,1	0,8705494	0,0	0,9245692
	EF1589		Unknown function	Enzymes of unknown specificity	acetyltransferase, GNAT family	0,5	0,19568	-0,2	0,6864021	0,5	0,0948367	0,1	0,7227387	0,0	0,8943872
	EF1590		Regulatory functions	Other	protease synthase and sporulation negative regulatory protein pai 1	-1,2	0,0015655	-0,4	0,3560008	1,2	0,0003252	-0,2	0,6192998	-0,4	0,2802293
	EF1591		Regulatory functions	DNA interactions	transcriptional regulator, AraC family	1,2	0,0018085	1,1	0,0167908	0,3	0,4211812	0,1	0,7559411	-0,3	0,3140506
	EF1592		Transport and binding proteins	Unknown substrate	ABC transporter, ATP-binding/permease protein	NA	NA	-0,1	0,7614095	NA	NA	0,0	0,8947078	0,1	0,8262611
	EF1593		Transport and binding proteins	Unknown substrate	ABC transporter, ATP-binding/permease protein	NA	NA	NA	NA	NA	NA	NA	NA	0,2	0,6290364
	EF1594		Hypothetical proteins		hypothetical protein	NA	NA	NA	NA	NA	NA	NA	NA	NA	NA
	EF1595		DNA metabolism	DNA replication, recombination, and repair	MutT/nudix family protein	1,3	0,0046169	-0,1	0,8004049	0,3	0,2879369	0,6	0,0585219	0,1	0,7893535
	EF1596		Cell envelope	Other	lipoprotein, putative	0,2	0,5574688	0,5	0,3244444	0,1	0,6660147	0,2	0,4678523	0,0	0,9813239
	EF1597	*katA*	Cellular processes	Detoxification	catalase/peroxidase	NA	NA	0,1	0,7659754	NA	NA	NA	NA	NA	NA
	EF1598	*phrB*	DNA metabolism	DNA replication, recombination, and repair	deoxyribodipyrimidine photolyase	NA	NA	NA	NA	NA	NA	NA	NA	-0,1	0,6665813
	EF1599		Regulatory functions	DNA interactions	TPR domain transcriptional regulator, Cro/CI family	NA	NA	NA	NA	NA	NA	NA	NA	0,1	0,7303918
	EF1601		Signal transduction	PTS	PTS system, IIABC components	0,7	0,1237198	-0,2	0,7123279	-0,2	0,529105	-0,2	0,4784679	-0,4	0,2232079
	EF1602		Energy metabolism	Biosynthesis and degradation of polysaccharides	glycosyl hydrolase, family 13	0,1	0,7608482	-0,1	0,9000482	-0,7	0,0786589	-0,2	0,5264022	0,0	0,9010411
	EF1603	*scrB-1*	Energy metabolism	Biosynthesis and degradation of polysaccharides	sucrose-6-phosphate dehydrogenase	-1,0	0,0257535	0,1	0,8312251	NA	NA	0,2	0,516271	-0,2	0,5778688
	EF1604	*scrR-1*	Regulatory functions	DNA interactions	sucrose operon repressor ScrR	NA	NA	-0,2	0,6638939	0,3	0,4468696	0,1	0,8419102	-0,1	0,6760691
	EF1605		Hypothetical proteins		hypothetical protein	NA	NA	NA	NA	NA	NA	NA	NA	-0,3	0,4426547
	EF1606		Energy metabolism	Biosynthesis and degradation of polysaccharides	glycosyl hydrolase, family 1	-1,2	0,009907	-0,2	0,6636851	0,6	0,0426373	0,3	0,429048	0,2	0,4724294
	EF1608		Fatty acid and phospholipid metabolism	Biosynthesis	cardiolipin synthetase, putative	2,5	**0**	1,1	0,0154552	-0,2	0,5959339	0,3	0,3831106	0,1	0,8707301
	EF1609		Hypothetical proteins	Conserved	conserved hypothetical protein	1,1	0,0034251	-0,2	0,6322202	NA	NA	0,3	0,4624577	-0,4	0,2772511
	EF1610		Hypothetical proteins	Domain	conserved domain protein	NA	NA	-0,2	0,6671847	-0,7	0,0276451	-0,8	0,0148218	0,0	0,9361151
	EF1611	*ppaC*	Central intermediary metabolism	Phosphorus compounds	inorganic pyrophosphatase, manganese-dependent	NA	NA	-0,4	0,3437791	0,1	0,780768	-0,1	0,6757039	0,0	0,9784909
	EF1612	*pflA*	Energy metabolism	Fermentation	pyruvate formate-lyase activating enzyme	-3,0	**0**	-0,9	0,0502868	-2,2	**0**	-1,1	0,0010425	0,0	0,9330254
	EF1613	*pflB*	Energy metabolism	Fermentation	formate acetyltransferase	-3,2	**0**	-0,8	0,0956574	-2,3	**0**	-1,1	0,0006797	-0,1	0,8227838
	EF1614	*parC*	DNA metabolism	DNA replication, recombination, and repair	DNA topoisomerase IV, A subunit	-0,6	0,0864778	-0,5	0,2678656	-0,1	0,8497557	-0,2	0,5682338	-0,1	0,8220769
	EF1615	*parE*	DNA metabolism	DNA replication, recombination, and repair	DNA topoisomerase IV, B subunit	NA	NA	-1,1	0,0187497	-0,1	0,7721682	-0,2	0,5152709	-0,2	0,6502015
	EF1616		Unknown function	General	CoA-binding domain protein	NA	NA	0,4	0,3444083	0,2	0,6337121	0,4	0,2275149	0,1	0,6858529
	EF1617		Hypothetical proteins	Conserved	conserved hypothetical protein	NA	NA	0,3	0,5394601	NA	NA	-0,1	0,8191449	0,2	0,4805503
	EF1618	*eutH*	Energy metabolism	Amino acids and amines	ethanolamine utilization protein EutH	NA	NA	0,4	0,4452464	NA	NA	NA	NA	0,0	0,9644556
	EF1619		Cellular processes	Other	carbon dioxide concentrating mechanism protein CcmL, putative	NA	NA	0,2	0,7451403	NA	NA	0,4	0,3898559	-0,1	0,8042258
	EF1620		Hypothetical proteins		hypothetical protein	0,6	0,0927339	0,2	0,6053076	NA	NA	NA	NA	-0,4	0,2705071
	EF1621		Hypothetical proteins	Conserved	conserved hypothetical protein	0,7	0,0539595	0,3	0,5574776	-0,2	0,5726445	0,4	0,1888754	0,0	0,8868815
	EF1622		Hypothetical proteins	Domain	conserved domain protein	1,8	**0,0000039**	0,2	0,725391	-0,3	0,4046288	0,0	0,91999	0,1	0,874285
	EF1623		Central intermediary metabolism	Other	microcompartment protein	NA	NA	-0,1	0,8212921	NA	NA	NA	NA	0,0	0,927699
	EF1624		Energy metabolism	Fermentation	aldehyde dehydrogenase, putative	NA	NA	0,1	0,9071122	-0,6	0,0641234	-0,4	0,2673138	0,2	0,5513006
	EF1625		Unknown function	General	microcompartment protein family	NA	NA	0,1	0,8427701	NA	NA	NA	NA	0,4	0,2842588
	EF1626	*eutL*	Energy metabolism	Amino acids and amines	ethanolamine utilization protein EutL	NA	NA	-0,2	0,6827578	NA	NA	NA	NA	0,1	0,721041
	EF1627	*eutC*	Energy metabolism	Amino acids and amines	ethanolamine ammonia-lyase small subunit	NA	NA	0,5	0,2707732	NA	NA	NA	NA	0,1	0,819925
	EF1629	*eutB*	Energy metabolism	Amino acids and amines	ethanolamine ammonia-lyase large subunit	NA	NA	0,4	0,4301137	NA	NA	NA	NA	0,2	0,5571683
	EF1630		Energy metabolism	Amino acids and amines	ethanolamine utilization protein EutA, putative	0,9	0,0348813	0,9	0,1283202	-0,2	0,6777567	0,8	0,0457321	0,2	0,6117822
	EF1632		Signal transduction	Two-component systems	sensor histidine kinase	-0,2	0,6126449	0,2	0,7189071	-0,7	0,0268222	0,2	0,5590819	0,0	0,9867021
	EF1633		Signal transduction	Two-component systems	response regulator	0,6	0,2000111	0,1	0,8346328	-0,4	0,2339391	0,2	0,6382345	-0,4	0,2200819
	EF1634		Unknown function	General	propanediol utilization protein PduU	NA	NA	0,7	0,1497987	-0,4	0,2359585	0,0	0,9228369	0,1	0,8707043
	EF1635		Energy metabolism	Fermentation	propanol dehydrogenase PduQ, putative	NA	NA	NA	NA	NA	NA	NA	NA	NA	NA
	EF1637		Energy metabolism	Other	ATP:cob(I)alamin adenosyltransferase, putative	NA	NA	NA	NA	NA	NA	0,0	0,9569773	0,0	0,969493
	EF1638	*eutP*	Unknown function	General	ethanolamine utilization protein, EutP	1,1	0,003458	-0,2	0,677397	-0,4	0,2596937	0,3	0,3330913	0,1	0,7723467
	EF1639		Transport and binding proteins	Cations and iron carrying compounds	iron compound ABC transporter, ATP-binding protein	-0,4	0,3221864	-0,4	0,4412258	0,0	0,9613297	-0,3	0,3279914	0,0	0,9787962
	EF1640		Transport and binding proteins	Cations and iron carrying compounds	iron compound ABC transporter, permease protein	-1,3	0,0036307	-0,1	0,7646964	0,0	0,9171251	-0,8	0,0213733	0,2	0,6156367
	EF1641		Transport and binding proteins	Cations and iron carrying compounds	iron compound ABC transporter, iron compound-binding protein	NA	NA	-0,1	0,8305706	0,1	0,7462827	-0,4	0,2876693	0,3	0,3728239
	EF1643		Hypothetical proteins	Conserved	conserved hypothetical protein TIGR00023	NA	NA	-0,2	0,6367882	-0,4	0,2381638	-0,1	0,8627354	-0,2	0,6237341
	EF1644		Unknown function	General	lacX protein, putative	0,8	0,0358008	0,1	0,8997648	-0,2	0,584209	0,0	0,8920828	-0,4	0,2720419
	EF1645	*codY*	Regulatory functions	DNA interactions	transcriptional regulator CodY	0,6	0,1269946	0,1	0,825926	-0,1	0,7902367	0,0	0,9536097	-0,4	0,2380288
	EF1646	*hslU*	Cellular processes	Adaptations to atypical conditions	heat shock protein HslVU, ATPase subunit HslU	NA	NA	0,0	0,9266193	-0,3	0,2891729	-0,3	0,3360813	-0,3	0,4337311
	EF1647	*hslV*	Protein fate	Protein folding and stabilization	heat shock protein HslV	0,2	0,5799324	-0,2	0,6449683	0,1	0,7757838	-0,1	0,7157729	-0,1	0,836645
	EF1648		DNA metabolism	DNA replication, recombination, and repair	site-specific recombinase, phage integrase family	0,1	0,7524542	-0,3	0,5665132	0,1	0,803678	0,3	0,3212768	-0,2	0,6352412
	EF1649	*gid*	Unknown function	General	glucose-inhibited division protein	-1,5	0,0000478	-0,9	0,0506447	-0,2	0,6171352	0,0	0,9350178	-0,2	0,5108005
	EF1650	*topA*	DNA metabolism	DNA replication, recombination, and repair	DNA topoisomerase I	NA	NA	NA	NA	NA	NA	NA	NA	NA	NA
	EF1651		Unknown function	General	abortive infection protein	NA	NA	-0,3	0,5093834	-0,2	0,6014964	-0,4	0,2026585	0,2	0,5646701
	EF1652		Cellular processes	DNA transformation	DNA processing protein DprA, putative	NA	NA	0,6	0,2004359	NA	NA	NA	NA	0,2	0,5950655
	EF1653	*rnhB*	Transcription	Degradation of RNA	ribonuclease HII	-1,3	0,00328	-0,8	0,0704398	-0,3	0,3955915	-0,1	0,7288216	-0,1	0,8548313
	EF1654		Unknown function	General	GTPase of unknown function	-0,4	0,4032073	-0,9	0,0608148	-0,6	0,0759978	0,1	0,7660858	-0,1	0,767446
	EF1655		Biosynthesis of cofactors, prosthetic groups, carriers	Pantothenate and coenzyme A	2-dehydropantoate 2-reductase, putative	0,9	0,0165934	-0,1	0,7868225	NA	NA	0,9	0,0061586	0,0	0,9942571
	EF1656		Regulatory functions	DNA interactions	transcriptional regulator, LysR family	0,5	0,1799571	0,6	0,1942257	-0,2	0,6025295	-0,2	0,5105249	-0,5	0,1694905
	EF1657		Cell envelope	Other	membrane protein, putative	-0,3	0,5688109	0,7	0,1507333	0,0	0,8816361	0,2	0,5136665	-0,4	0,1895711
	EF1658	*bkdC*	Energy metabolism	Amino acids and amines	branched-chain alpha-keto acid, E2, dihydrolipoamide acetyltransfer[illegible]	NA	NA	0,7	0,1520178	0,2	0,5582058	1,0	0,0041206	0,1	0,8575997
	EF1659	*bkdB*	Energy metabolism	Amino acids and amines	branched-chain alpha-keto acid dehydrogenase, E1 beta subunit	NA	NA	0,6	0,2169749	NA	NA	2,4	**0**	0,2	0,5466763
	EF1660	*bkdA*	Energy metabolism	Amino acids and amines	branched-chain alpha-keto acid dehydrogenase, E1 alpha subunit	1,7	0,0001951	-0,6	0,3174973	1,2	0,002379	2,6	**0**	0,4	0,1830594
	EF1661	*bkdD*	Energy metabolism	Amino acids and amines	branched-chain alpha-keto acid dehydrogenase, E3	NA	NA	NA	NA	NA	NA	3,2	**0**	0,5	0,1109086
	EF1662	*buk*	Energy metabolism	Other	butyrate kinase	1,1	0,00339	0,3	0,5911734	1,6	**0,0000006**	3,3	**0**	1,0	0,0018947
	EF1663	*ptb*	Fatty acid and phospholipid metabolism	Degradation	branched-chain phosphotransacylase	NA	NA	0,5	0,3272116	0,7	0,0333312	1,7	**0,0000002**	0,7	0,0294925
	EF1664		Hypothetical proteins	Conserved	conserved hypothetical protein	-0,7	0,1108332	0,1	0,7512483	0,6	0,0516129	0,4	0,2601818	0,1	0,7079855
	EF1665		Hypothetical proteins	Conserved	conserved hypothetical protein	NA	NA	-0,4	0,4535154	NA	NA	NA	NA	0,0	0,9812965
	EF1666		Hypothetical proteins		hypothetical protein	NA	NA	NA	NA	NA	NA	NA	NA	0,3	0,3357192
	EF1667		Unknown function	Enzymes of unknown specificity	short chain dehydrogenase family protein	1,6	0,0004471	-0,2	0,6511979	1,0	0,0022562	0,8	0,0174033	0,0	0,9170586
	EF1668		Regulatory functions	DNA interactions	transcriptional regulator, MarR family	NA	NA	-0,2	0,7258603	NA	NA	NA	NA	0,5	0,1775412
	EF1669		Unknown function	Enzymes of unknown specificity	glyoxylase family protein	NA	NA	0,2	0,6839844	-0,1	0,8725924	0,2	0,569999	-0,3	0,3464977
	EF1670		Unknown function	Enzymes of unknown specificity	phospholipase/carboxylesterase family protein	NA	NA	0,5	0,3274641	NA	NA	-0,7	0,0334492	0,0	0,9215255
	EF1671		Unknown function	Enzymes of unknown specificity	oxidoreductase, zinc-binding	2,8	**0**	1,0	0,0376094	0,5	0,1303609	0,5	0,1698537	0,1	0,7839228
	EF1672		Transport and binding proteins	Unknown substrate	permease protein, putative	2,1	**0**	2,1	**0,0000073**	0,5	0,2142899	0,5	0,2483724	0,5	0,1602295
	EF1673		Transport and binding proteins	Unknown substrate	ABC transporter, ATP-binding protein	1,9	**0,0000004**	0,6	0,329552	-0,2	0,5815252	0,7	0,0406355	1,0	0,002066
	EF1674		Hypothetical proteins		hypothetical protein	-0,2	0,5819383	0,0	0,9755296	-0,1	0,7139368	-0,3	0,3875419	-0,1	0,8421504
	EF1675		Transport and binding proteins	Unknown substrate	ABC transporter, ATP-binding protein	NA	NA	0,0	0,9766184	-0,5	0,215961	-0,4	0,2076943	0,1	0,7866068

Operon[a]	Locus	Gene	Functional category	Subcategory	Putative function	Blood[b]	P-value[c]	YTB_5[b]	P-value[c]	YTB_15[b]	P-value[c]	YTB_30[b]	P-value[c]	YTB_60[b]	P-value[c]
	EF1676		Regulatory functions	DNA interactions	transcriptional regulator, GntR family	NA	NA	0,1	0,7555291	-0,9	0,0039209	-0,4	0,2041015	0,2	0,5643922
	EF1677		Cell envelope	Other	lipoprotein, putative	1,8	**0,0000007**	0,0	0,9676544	0,6	0,1033856	NA	NA	0,3	0,3454236
	EF1678		Protein fate	Protein and peptide secretion and trafficking	signal peptidase I	-0,5	0,3047544	0,0	0,9874916	0,5	0,0847661	0,5	0,128411	0,2	0,4812535
	EF1679		Protein fate	Degradation of proteins, peptides, and glycopeptides	carboxyl-terminal protease	-0,3	0,5615618	0,1	0,7626363	0,3	0,3533854	0,3	0,3863781	0,2	0,5303856
	EF1680		Hypothetical proteins	Conserved	conserved hypothetical protein	0,8	0,0286347	0,1	0,7878225	-0,1	0,8019317	0,3	0,2974745	0,3	0,3238872
	EF1681	*msrA*	Protein fate	Protein modification and repair	peptide methionine sulfoxide reductase	0,9	0,0103227	0,5	0,2429547	0,3	0,3719026	0,0	0,8902352	0,1	0,8279789
	EF1682		Hypothetical proteins	Conserved	conserved hypothetical protein	NA	NA	-0,1	0,7520587	-0,1	0,769272	-0,1	0,8555098	0,3	0,3068431
	EF1683		Unknown function	Enzymes of unknown specificity	Lipase/Acylhydrolase, putative	-0,3	0,4947257	0,0	0,9782569	0,5	0,1118443	0,3	0,3602642	0,3	0,4421494
	EF1684		Unknown function	General	DegV family protein, putative	0,1	0,7701904	0,6	0,1673566	0,2	0,4970258	0,1	0,7323523	-0,2	0,6239189
	EF1685	*hlyIII*	Cellular processes	Pathogenesis	hemolysin III	0,6	0,089172	0,5	0,3097689	0,2	0,5404443	0,3	0,3968837	0,1	0,8527991
	EF1686		Hypothetical proteins		hypothetical protein	2,0	**0,0000001**	0,1	0,765979	-0,1	0,7947547	-0,1	0,7999183	-0,2	0,5284184
	EF1687	*apt*	Purines, pyrimidines, nucleosides, and nucleotides	Salvage of nucleosides and nucleotides	adenine phosphoribosyltransferase	-2,1	**0**	-0,7	0,1207504	-0,2	0,532647	-0,4	0,244331	0,0	0,9824589
	EF1688	*recJ*	DNA metabolism	DNA replication, recombination, and repair	single-stranded-DNA-specific exonuclease RecJ	-1,6	0,0002772	-1,0	0,0387633	-0,4	0,2675518	-0,4	0,2451327	0,1	0,8447342
	EF1689		Hypothetical proteins		hypothetical protein	NA	NA	-0,5	0,287057	-0,1	0,8090657	0,1	0,6549781	0,3	0,446586
	EF1690		Unknown function	Enzymes of unknown specificity	oxidoreductase, short-chain dehydrogenase/reductase family	NA	NA	-0,7	0,1144588	0,2	0,4968962	-0,1	0,7377961	0,3	0,3975794
	EF1691		Unknown function	Enzymes of unknown specificity	metallo-beta-lactamase, AtsA/ElaC family	0,1	0,7228278	-0,6	0,1742696	-0,2	0,510252	0,3	0,3556405	-0,1	0,7376955
	EF1692		Hypothetical proteins	Conserved	conserved hypothetical protein	1,3	0,0004832	0,5	0,308496	0,2	0,4289785	0,4	0,2879196	0,1	0,6675752
	EF1693		Unknown function	General	KH domain protein	-2,5	**0**	-0,4	0,3751564	-0,2	0,5450288	-0,9	0,0070479	0,5	0,1269305
	EF1694	*rpsP*	Protein synthesis	Ribosomal proteins: synthesis and modification	ribosomal protein S16	-2,9	**0**	-0,1	0,8026592	-0,1	0,8150069	-0,7	0,0378384	0,6	0,0931895
	EF1695		Unknown function	Enzymes of unknown specificity	acetyltransferase, GNAT family	NA	NA	-0,7	0,1053953	0,2	0,4469157	-0,1	0,6921503	0,6	0,0690066
	EF1698		Unknown function	General	NADPH-dependent FMN reductase domain protein	1,7	**0,0000049**	0,3	0,4983957	0,5	0,1334705	0,3	0,4055226	0,1	0,8276111
	EF1699		Regulatory functions	DNA interactions	transcriptional regulator, MerR family	NA	NA	-0,2	0,664227	0,0	0,9494588	-0,3	0,45637	0,4	0,1834823
	EF1700	*ffh*	Protein fate	Protein and peptide secretion and trafficking	signal recognition particle protein	-1,3	0,0003369	-0,5	0,294926	-0,1	0,7836204	0,3	0,4546489	0,0	0,886719
	EF1701		Hypothetical proteins	Conserved	conserved hypothetical protein	NA	NA	NA	NA	NA	NA	NA	NA	NA	NA
	EF1702		Hypothetical proteins	Conserved	conserved hypothetical protein	-1,0	0,0330185	-0,4	0,3724196	0,6	0,0566443	0,2	0,5290096	0,4	0,2543254
	EF1703	*phoP*	Regulatory functions	DNA interactions	alkaline phosphatase synthesis transcr regulatory protein PhoP	-0,7	0,0667097	-0,5	0,2580727	0,1	0,6394533	0,3	0,3040857	0,3	0,4200961
	EF1704		Regulatory functions	Small molecule interactions	sensory box histidine kinase	NA	NA	NA	NA	NA	NA	NA	NA	NA	NA
	EF1705		Transport and binding proteins	Anions	phosphate-binding protein	0,4	0,2259874	-0,1	0,8061582	-0,2	0,5570293	-0,4	0,2199168	0,1	0,7333207
	EF1706		Unknown function	Enzymes of unknown specificity	aminotransferase, class I	-0,9	0,0459593	-0,2	0,7370118	0,1	0,680813	-0,4	0,2505597	0,3	0,4291115
	EF1707		Energy metabolism	Biosynthesis and degradation of polysaccharides	glycosyl hydrolase, family 38	0,0	0,8927108	0,5	0,2961523	0,2	0,5286	0,0	0,9483827	-0,9	0,0071122
	EF1708		Hypothetical proteins	Conserved	conserved hypothetical protein	-0,6	0,1793558	0,2	0,740786	-0,1	0,8614436	-0,1	0,8600041	-1,3	0,0001041
	EF1709		Regulatory functions	DNA interactions	sugar-binding transcriptional regulator, GntR family	0,3	0,4892866	0,3	0,4734839	0,6	0,0805317	0,3	0,4357351	-0,2	0,5943275
	EF1710		Regulatory functions	DNA interactions	transcriptional regulator, LysR family	0,1	0,7526021	-0,4	0,4393651	0,6	0,0651042	0,3	0,3134908	0,1	0,7357306
	EF1711		Central intermediary metabolism	Other	carbonic anhydrase, putative	-0,1	0,9042266	-0,4	0,4090755	1,0	0,0010721	0,2	0,6266282	-0,1	0,8657905
	EF1712	*pyrE*	Purines, pyrimidines, nucleosides, and nucleotides	Pyrimidine ribonucleotide biosynthesis	orotate phosphoribosyltransferase	-1,0	0,0089311	-0,7	0,125832	1,2	0,0001975	-0,5	0,1642448	0,2	0,6188046
	EF1713	*pyrF*	Purines, pyrimidines, nucleosides, and nucleotides	Pyrimidine ribonucleotide biosynthesis	orotidine 5`-phosphate decarboxylase	0,8	0,0386507	-0,5	0,3183573	0,8	0,0191165	0,0	0,9622056	0,2	0,5440218
	EF1714	*pyrD-2*	Purines, pyrimidines, nucleosides, and nucleotides	Pyrimidine ribonucleotide biosynthesis	dihydroorotate dehydrogenase	-1,4	0,0000968	-0,9	0,0432632	1,1	0,0003771	-0,4	0,1851241	0,1	0,8305025
	EF1715	*pyrDII*	Purines, pyrimidines, nucleosides, and nucleotides	Pyrimidine ribonucleotide biosynthesis	dihydroorotate dehydrogenase electron transfer subunit	-1,2	0,0011175	-0,9	0,0486374	1,0	0,0025433	-0,3	0,3955624	0,0	0,9036004
	EF1716	*pyraB*	Purines, pyrimidines, nucleosides, and nucleotides	Pyrimidine ribonucleotide biosynthesis	carbamoyl-phosphate synthase, large subunit	-0,2	0,5076688	-0,9	0,0535313	0,9	0,0047673	-0,4	0,2440529	-0,2	0,6209881
	EF1717	*pyraA*	Purines, pyrimidines, nucleosides, and nucleotides	Pyrimidine ribonucleotide biosynthesis	carbamoyl-phosphate synthase, small subunit	-0,6	0,0962418	-1,5	0,0010311	0,5	0,1405782	-0,1	0,798833	-0,5	0,0980765
	EF1718	*pyrC*	Purines, pyrimidines, nucleosides, and nucleotides	Pyrimidine ribonucleotide biosynthesis	dihydroorotase	-0,3	0,3533266	-0,8	0,0894106	0,1	0,8330065	0,3	0,387868	-0,4	0,2323285
	EF1719	*pyrB*	Purines, pyrimidines, nucleosides, and nucleotides	Pyrimidine ribonucleotide biosynthesis	aspartate carbamoyltransferase	-1,5	0,0008299	-1,1	0,0143043	0,1	0,8442207	-0,3	0,5212505	-0,1	0,7320139
	EF1720		Transport and binding proteins	Nucleosides, purines and pyrimidines	uracil permease	NA	NA	-1,6	0,000728	0,1	0,8528897	0,2	0,6449438	-0,3	0,2960229
	EF1721	*pyrR*	Transcription	Transcription factors	pyrimidine operon regulatory protein PyrR	0,3	0,4296931	-1,3	0,0037111	0,5	0,0910804	2,6	**0**	-0,3	0,3906649
	EF1722	*rluD*	Protein synthesis	tRNA and rRNA base modification	ribosomal large subunit pseudouridine synthase D	-0,2	0,5021898	-0,4	0,4004863	0,1	0,8461173	0,2	0,5873119	0,1	0,763563
	EF1723	*lspA*	Protein fate	Protein and peptide secretion and trafficking	signal peptidase II	-0,5	0,3176075	-0,5	0,282428	0,3	0,3134295	0,4	0,2178736	0,3	0,2994774
	EF1724		Unknown function	General	CBS domain protein	-0,4	0,2902575	-0,3	0,5544182	0,2	0,5211631	0,2	0,4918468	0,3	0,3174009
	EF1725	*fhs*	Central intermediary metabolism	One-carbon metabolism	formate--tetrahydrofolate ligase	0,0	0,9186559	-0,5	0,2826875	-0,3	0,4280255	0,1	0,88217	0,3	0,3675389
	EF1726		Cellular processes	Adaptations to atypical conditions	cold-shock domain family protein	0,1	0,888219	0,5	0,2974999	0,2	0,6191473	-0,1	0,7816717	0,4	0,2487949
	EF1727	*ebsa*	Cellular processes	Conjugation	ebsA protein	-0,5	0,29297	-0,5	0,293278	0,0	0,9292123	0,6	0,1147695	0,0	0,9059048
	EF1728	*ebsB*	Cellular processes	Conjugation	EbsB protein	0,2	0,5999854	-0,5	0,2928188	0,0	0,9466144	0,2	0,4735061	0,4	0,2538025
	EF1730		Cellular processes	Conjugation	EbsC protein	-1,1	0,0024064	-0,4	0,416802	-0,1	0,7507553	0,1	0,7096214	0,2	0,5188733
	EF1731	*aroD*	Amino acid biosynthesis	Aromatic amino acid family	3-dehydroquinate dehydratase, type I	-0,1	0,728124	-0,4	0,4064414	0,1	0,773867	0,4	0,2275418	0,1	0,8088797
	EF1732		Transport and binding proteins	Unknown substrate	ABC transporter, ATP-binding/permease protein, MDR family	-1,8	0,0000898	0,1	0,8474495	0,6	0,0485436	-0,4	0,2317907	0,4	0,1989322
	EF1733		Transport and binding proteins	Unknown substrate	ABC transporter, ATP-binding/permease protein, MDR family	-1,1	0,0029754	0,3	0,4541175	0,3	0,2684757	-0,6	0,0581404	0,5	0,162263
	EF1734		Hypothetical proteins	Conserved	conserved hypothetical protein	NA	NA	NA	NA	NA	NA	NA	NA	NA	NA
	EF1735		Hypothetical proteins		hypothetical protein	NA	NA	NA	NA	NA	NA	NA	NA	NA	NA
	EF1736	*nfo*	DNA metabolism	DNA replication, recombination, and repair	endonuclease IV	NA	NA	0,0	0,950237	0,2	0,4354509	0,0	0,9612389	-0,2	0,6268329
	EF1737		Hypothetical proteins		hypothetical protein	-1,1	0,0022867	-0,4	0,4050277	0,5	0,0870128	0,2	0,4561057	-0,1	0,8197492
	EF1738		Hypothetical proteins	Conserved	conserved hypothetical protein	-1,8	**0,0000017**	-0,5	0,2819439	0,6	0,0727344	0,3	0,3362413	-0,1	0,8297341
	EF1739	*tryS-2*	Protein synthesis	tRNA aminoacylation	tyrosyl-tRNA synthetase	-3,0	**0**	-0,4	0,3529184	-0,2	0,4687898	-0,3	0,3526123	-0,4	0,2293107
	EF1740		Cell envelope	Biosynthesis/degradation of murein sacculus/peptidoglycan	penicillin-binding protein 1B, putative	-0,3	0,4790959	-1,1	0,0155658	-0,3	0,2812626	0,1	0,7279005	0,0	0,92835
	EF1741	*ccpA*	Regulatory functions	DNA interactions	catabolite control protein A	-1,2	0,0012339	-0,8	0,070593	-0,2	0,4687126	0,1	0,6840821	-0,1	0,790946
	EF1743	*pepQ-2*	Protein fate	Degradation of proteins, peptides, and glycopeptides	proline dipeptidase	-0,4	0,3045471	-0,3	0,566544	0,0	0,9811208	0,3	0,3840551	0,1	0,7178354
	EF1744		Cellular processes	Adaptations to atypical conditions	general stress protein, putative	1,5	0,0000344	0,5	0,2562104	0,3	0,2683185	0,5	0,1633234	0,0	0,9611015
	EF1745		Hypothetical proteins	Conserved	conserved hypothetical protein	1,4	0,0001945	0,8	0,0970889	0,2	0,5520879	0,6	0,0941879	-0,1	0,7261472
	EF1746	*galU*	Cell envelope	Biosynthesis and degradation of surface poly/liposaccharides	UTP-glucose-1-phosphate uridylyltransferase	-0,5	0,1676478	-0,1	0,8371518	-0,1	0,8490669	0,3	0,3174279	-0,1	0,8310425
	EF1747	*gpsA*	Energy metabolism	Other	glycerol-3-phosphate dehydrogenase (NAD(P)+)	-0,6	0,099814	-0,4	0,4394768	0,0	0,940366	0,1	0,6703914	-0,1	0,7868061
	EF1748	*lgt*	Protein fate	Protein modification and repair	prolipoprotein diacylglyceryl transferase	-0,6	0,0799124	-0,2	0,7115618	0,1	0,7799603	0,1	0,7627472	0,2	0,4919899
	EF1749	*hprK*	Regulatory functions	Protein interactions	HPr(Ser) serine kinase/phosphatase	-1,0	0,0078537	-0,4	0,3792959	0,2	0,6293024	0,4	0,2810281	0,2	0,5684603
	EF1750		Unknown function	General	endo/excinuclease amino terminal domain protein	NA	NA	-0,2	0,6666498	-0,3	0,4129196	0,4	0,2453543	-0,2	0,452635
	EF1751		Cell envelope	Other	membrane protein, putative	2,0	**0**	1,9	0,0000709	0,2	0,5732158	0,4	0,3013773	0,3	0,4196812
	EF1752		Hypothetical proteins	Conserved	conserved hypothetical protein	1,8	**0,0000014**	1,2	0,0134224	0,0	0,9179661	0,1	0,6934322	0,2	0,4726979
	EF1753		Hypothetical proteins	Conserved	conserved hypothetical protein	2,6	**0**	3,2	**0**	-0,1	0,8003058	0,6	0,0665074	1,0	0,0029682
	EF1754		Unknown function	General	PhoU family protein	-1,1	0,0026399	-0,4	0,4003949	0,2	0,549419	-0,2	0,552424	0,1	0,72518
	EF1755		Transport and binding proteins	Anions	phosphate ABC transporter, ATP-binding protein	-1,3	0,0003627	-0,3	0,4851122	0,0	0,9687626	-0,2	0,6424366	0,0	0,8863766
	EF1756		Transport and binding proteins	Anions	phosphate ABC transporter, ATP-binding protein	-1,9	**0,0000001**	-0,8	0,0763268	-0,2	0,5285653	-0,2	0,4874558	0,0	0,9798074
	EF1757		Transport and binding proteins	Anions	phosphate ABC transporter, permease protein	-1,1	0,0019351	-0,7	0,1548965	-0,1	0,7903784	-0,4	0,2272837	-0,1	0,8707615
	EF1758		Transport and binding proteins	Anions	phosphate ABC transporter, permease protein	-1,2	0,001019	-0,8	0,085619	-0,4	0,2442187	-0,1	0,6984876	-0,1	0,6583448
	EF1759		Transport and binding proteins	Anions	phosphate ABC transporter, phosphate-binding protein	-0,9	0,0102732	-0,7	0,1394662	-0,6	0,0755975	0,0	0,9565212	0,0	0,9939857
	EF1760		Cellular processes	Cell division	cell division ABC transporter, permease protein FtsX, putative	-2,7	**0**	-0,7	0,1086486	0,1	0,6583531	-0,6	0,0992165	-0,1	0,6626561
	EF1761	*ftsE*	Cellular processes	Cell division	cell division ATP-binding protein FtsE	-2,4	**0**	-1,0	0,0287567	0,0	0,9484233	-0,4	0,1937863	-0,4	0,2370005
	EF1762	*prfB*	Protein synthesis	Translation factors	peptide chain release factor 2, programmed frameshift	-3,3	**0**	-0,6	0,1740802	-0,2	0,4518265	-0,6	0,0682132	0,2	0,6275811

Operon[a]	Locus	Gene	Functional category	Subcategory	Putative function	Blood[b]	P-value[c]	YTB_5[b]	P-value[c]	YTB_15[b]	P-value[c]	YTB_30[b]	P-value[c]	YTB_60[b]	P-value[c]
	EF1763	*secA*	Protein fate	Protein and peptide secretion and trafficking	preprotein translocase, SecA subunit	-0,8	0,0385632	-0,9	0,0651147	0,1	0,6424998	0,4	0,2144391	0,1	0,6604851
	EF1764	*yfiA*	Protein synthesis	Translation factors	ribosomal subunit interface protein	2,6	**0**	1,4	0,002916	0,3	0,3679976	0,8	0,0224328	-0,1	0,836264
	EF1765	*comFC*	Cellular processes	DNA transformation	competence protein F	NA	NA	0,3	0,498285	NA	NA	NA	NA	0,0	0,9924491
	EF1767	*comFA*	Cellular processes	DNA transformation	competence protein F	NA	NA	NA	NA	NA	NA	NA	NA	NA	NA
	EF1768		Transport and binding proteins	Unknown substrate	ABC transporter, ATP-binding protein	NA	NA	NA	NA	NA	NA	NA	NA	NA	NA
	EF1769		Signal transduction	PTS	PTS system, IIB component, putative	0,5	0,1376317	1,1	0,0151538	0,1	0,6353422	0,3	0,4234079	0,0	0,9387554
	EF1770		Hypothetical proteins		hypothetical protein	1,2	0,0025308	-0,3	0,585818	-0,4	0,3536938	0,4	0,2022129	0,3	0,3034025
	EF1771		Hypothetical proteins	Conserved	conserved hypothetical protein TIGR00257	NA	NA	-0,2	0,6408439	-0,1	0,7415121	-0,2	0,5426608	0,2	0,5041511
	EF1772		Hypothetical proteins	Conserved	conserved hypothetical protein	-0,1	0,7510007	-0,5	0,25426	NA	NA	-0,1	0,6969091	0,1	0,69708
	EF1773		Fatty acid and phospholipid metabolism	Biosynthesis	short chain dehydrogenase	-1,1	0,0026597	-0,7	0,1360577	0,4	0,1835067	-0,1	0,7480866	0,3	0,3600938
	EF1774		Hypothetical proteins	Conserved	conserved hypothetical protein	-2,2	**0**	-0,8	0,0821398	0,2	0,4876029	-0,3	0,3812981	0,2	0,6075843
	EF1775		Hypothetical proteins		hypothetical protein	0,3	0,4853085	0,3	0,5737833	0,3	0,3574542	-0,1	0,820533	0,2	0,5832753
	EF1776		Hypothetical proteins	Domain	conserved domain protein	0,5	0,21307	0,5	0,3207027	0,0	0,9265893	0,4	0,2864233	0,3	0,4161865
	EF1777	*purD*	Purines, pyrimidines, nucleosides, and nucleotides	Purine ribonucleotide biosynthesis	phosphoribosylamine--glycine ligase	NA	NA	0,2	0,7269337	NA	NA	NA	NA	-0,4	0,2471586
	EF1778	*purH*	Purines, pyrimidines, nucleosides, and nucleotides	Purine ribonucleotide biosynthesis	phosphoribosylaminoimidazolecarboxamide formyltransferase	NA	NA	NA	NA	NA	NA	NA	NA	1,4	0,0000493
	EF1779	*purN*	Purines, pyrimidines, nucleosides, and nucleotides	Purine ribonucleotide biosynthesis	phosphoribosylglycinamide formyltransferase	NA	NA	NA	NA	NA	NA	NA	NA	0,4	0,2490147
	EF1780	*purM*	Purines, pyrimidines, nucleosides, and nucleotides	Purine ribonucleotide biosynthesis	phosphoribosylformylglycinamidine cyclo-ligase	NA	NA	NA	NA	NA	NA	NA	NA	NA	NA
	EF1781	*purF*	Purines, pyrimidines, nucleosides, and nucleotides	Purine ribonucleotide biosynthesis	amidophosphoribosyltransferase	1,1	0,0039304	0,1	0,8282761	-0,2	0,6482972	0,2	0,6086009	0,2	0,5541987
	EF1782	*purL*	Purines, pyrimidines, nucleosides, and nucleotides	Purine ribonucleotide biosynthesis	phosphoribosylformylglycinamidine synthase II	0,2	0,5492743	0,3	0,5679362	NA	NA	NA	NA	0,1	0,8629566
	EF1783	*purQ*	Purines, pyrimidines, nucleosides, and nucleotides	Purine ribonucleotide biosynthesis	phosphoribosylformylglycinamidine synthetase I	NA	NA	-0,2	0,6913341	NA	NA	NA	NA	-0,2	0,6420435
	EF1784	*purS*	Purines, pyrimidines, nucleosides, and nucleotides	Purine ribonucleotide biosynthesis	phosphoribosylformylglycinamidine synthase, PurS protein	NA	NA	NA	NA	NA	NA	NA	NA	NA	NA
	EF1785	*purC*	Purines, pyrimidines, nucleosides, and nucleotides	Purine ribonucleotide biosynthesis	phosphoribosylaminoimidazole-succinocarboxamide synthase	NA	NA	NA	NA	NA	NA	0,5	0,2454151	NA	NA
	EF1786	*purK-1*	Purines, pyrimidines, nucleosides, and nucleotides	Purine ribonucleotide biosynthesis	phosphoribosylaminoimidazole carboxylase, ATPase subunit	NA	NA	NA	NA	NA	NA	NA	NA	NA	NA
	EF1787	*purE*	Purines, pyrimidines, nucleosides, and nucleotides	Purine ribonucleotide biosynthesis	phosphoribosylaminoimidazole carboxylase, catalytic subunit	0,7	0,0496166	0,1	0,8207781	0,0	0,9253671	0,9	0,0067791	0,1	0,7419847
	EF1789		Unknown function	General	SPFH domain/Band 7 family protein	0,2	0,6244063	0,3	0,4589033	-0,1	0,8494872	0,1	0,8090681	-0,2	0,5599836
	EF1790		Unknown function	General	YjeF-related protein	NA	NA	0,3	0,461413	NA	NA	0,0	0,9753943	0,2	0,5455173
	EF1791		Transport and binding proteins	Amino acids, peptides and amines	pheromone binding protein	NA	NA	NA	NA	NA	NA	NA	NA	NA	NA
	EF1792		Hypothetical proteins	Conserved	conserved hypothetical protein	NA	NA	NA	NA	NA	NA	NA	NA	0,5	0,1348715
	EF1793	*ilvE*	Amino acid biosynthesis	Pyruvate family	branched-chain amino acid aminotransferase	-0,5	0,1470175	-0,2	0,6734124	-0,2	0,6015335	0,2	0,4822299	0,0	0,9481611
	EF1794		Hypothetical proteins	Conserved	conserved hypothetical protein	2,4	**0**	0,2	0,6996077	0,1	0,7422399	0,6	0,0742052	0,3	0,4373923
	EF1796		Cell envelope	Other	lipoprotein, putative	4,2	**0**	0,8	0,0979625	1,0	0,0023854	1,3	0,0001042	0,5	0,1223972
	EF1797		Hypothetical proteins	Conserved	conserved hypothetical protein	NA	NA	-0,5	0,2759167	-0,1	0,8708041	-0,3	0,4465853	0,5	0,1352728
	EF1798		Hypothetical proteins		hypothetical protein	-2,2	**0,0000016**	-0,7	0,1575203	0,1	0,8425479	0,1	0,8065787	0,2	0,4877349
	EF1800		Hypothetical proteins	Conserved	conserved hypothetical protein	NA	NA	0,1	0,8018996	-0,3	0,3087404	-0,3	0,3063576	-0,5	0,1283149
	EF1801		Signal transduction	PTS	PTS system, IIA component	1,9	**0,0000003**	0,9	0,0651455	0,0	0,9170767	-0,4	0,2029686	-0,6	0,0778916
	EF1802		Signal transduction	PTS	PTS system, IID component	NA	NA	0,8	0,0945175	0,1	0,7866395	-0,4	0,2070422	-0,5	0,1724012
	EF1803		Signal transduction	PTS	PTS system, IIC component	0,7	0,0543891	0,0	0,9529704	0,4	0,2558182	-0,5	0,101578	-0,2	0,4610049
	EF1804		Signal transduction	PTS	PTS system, IIB component	0,9	0,0445811	0,4	0,5048945	0,1	0,7194215	-0,3	0,4050725	-0,1	0,6610423
	EF1805		Energy metabolism	Biosynthesis and degradation of polysaccharides	glycosyl hydrolase, family 35	0,2	0,6190308	0,1	0,8507704	0,1	0,72608	-0,7	0,1006736	-0,1	0,8173122
	EF1806	*lacC*	Energy metabolism	Biosynthesis and degradation of polysaccharides	tagatose-6-phosphate kinase	0,4	0,2402364	0,3	0,4528781	0,1	0,6755479	-0,8	0,0629226	-0,5	0,1396795
	EF1807	*lacD-2*	Energy metabolism	Biosynthesis and degradation of polysaccharides	tagatose 1,6-diphosphate aldolase	NA	NA	0,4	0,355218	-0,2	0,5356217	-0,6	0,0756238	0,0	0,9192497
	EF1808		Unknown function	Enzymes of unknown specificity	agaS protein	NA	NA	0,7	0,1058731	-0,3	0,3613335	-0,6	0,0860062	-0,2	0,5821912
	EF1809		Regulatory functions	DNA interactions	transcriptional regulator, GntR family	NA	NA	0,4	0,3410779	-0,5	0,0987527	-0,2	0,6546841	0,1	0,6956106
	EF1810	*gspA-1*	Cellular processes	Adaptations to atypical conditions	general stress protein A	2,6	**0**	0,0	0,9799842	0,4	0,3519444	0,0	0,9800765	0,6	0,0814148
	EF1811	*gspA-2*	Cellular processes	Adaptations to atypical conditions	general stress protein A	2,3	**0**	-0,5	0,3468801	NA	NA	NA	NA	0,1	0,7865928
	EF1812		Hypothetical proteins		hypothetical protein	NA	NA	NA	NA	NA	NA	NA	NA	NA	NA
	EF1813		Unknown function	General	sulfatase domain protein	NA	NA	0,3	0,5215434	NA	NA	NA	NA	-0,4	0,2107176
	EF1814		Transport and binding proteins	Other	drug resistance transporter, EmrB/QacA family protein	NA	NA	0,4	0,4451555	NA	NA	NA	NA	0,1	0,8565941
	EF1815		Regulatory functions	DNA interactions	transcriptional regulator, LysR family, putative	NA	NA	-0,1	0,8534258	NA	NA	NA	NA	0,4	0,2390275
	EF1816		Hypothetical proteins	Conserved	conserved hypothetical protein	NA	NA	0,1	0,7784639	-0,4	0,1661054	-0,1	0,7194168	0,4	0,1954889
	EF1817	*sprE*	Protein fate	Degradation of proteins, peptides, and glycopeptides	serine proteinase, V8 family	0,5	0,151262	-0,4	0,3906751	-0,4	0,2007429	-0,1	0,8819776	0,3	0,4228017
	EF1818	*gelE*	Cell envelope	Other	coccolysin	NA	NA	-0,2	0,6420974	-0,8	0,0075525	-0,3	0,4395432	0,1	0,7698691
	EF1820	*fsrC*	Signal transduction	Two-component systems	histidine kinase, putative	NA	NA	-0,3	0,5173119	-0,7	0,0357996	-0,5	0,1414678	0,4	0,2013957
	EF1821	*fsrB*	Unknown function	General	agrBfs protein	NA	NA	-0,9	0,0516739	NA	NA	-0,8	0,0204778	0,5	0,105771
	EF1822	*fsrA*	Signal transduction	Two-component systems	response regulator	NA	NA	-0,1	0,8683823	NA	NA	NA	NA	0,2	0,4744175
	EF1823		Cell envelope	Biosynthesis/degradation of murein sacculus/peptidoglycan	N-acetylmuramoyl-L-alanine amidase, family 4	NA	NA	0,6	0,3232522	-0,1	0,7968496	-0,3	0,4958729	-0,8	0,0119453
	EF1824		Cell envelope	Biosynthesis and degradation of surface poly/liposaccharides	glycosyl hydrolase, family 31/fibronectin type III domain protein	NA	NA	0,2	0,7440071	-0,2	0,5567383	-0,2	0,4700008	-0,1	0,7077536
	EF1825		Hypothetical proteins	Domain	conserved domain protein	-1,6	0,0004617	-0,5	0,3069802	-1,1	0,0003881	-0,5	0,141677	-0,2	0,5893833
	EF1826		Energy metabolism	Fermentation	alcohol dehydrogenase, zinc-containing	-0,6	0,1510498	-0,5	0,2954311	-1,3	0,0000267	-0,6	0,0974839	-0,1	0,6831047
	EF1827		Hypothetical proteins	Conserved	conserved hypothetical protein	NA	NA	0,4	0,4555355	NA	NA	NA	NA	0,3	0,4153216
	EF1828		Transport and binding proteins	Other	glycerol uptake facilitator protein, putative	NA	NA	1,1	0,0445999	NA	NA	NA	NA	0,1	0,7381723
	EF1829		Signal transduction	PTS	PTS system, IID component	NA	NA	1,3	0,0270647	NA	NA	0,2	0,5496578	-0,1	0,8527728
	EF1830		Signal transduction	PTS	PTS system, IIC component	0,8	0,0227429	0,5	0,3930869	0,0	0,9774585	0,0	0,9588023	0,0	0,9986884
	EF1833		Hypothetical proteins		hypothetical protein	NA	NA	1,1	0,0448795	NA	NA	NA	NA	NA	NA
	EF1834	*lacB*	Energy metabolism	Biosynthesis and degradation of polysaccharides	galactose-6-phosphate isomerase, LacB subunit	NA	NA	0,6	0,3239511	NA	NA	0,1	0,825241	-0,2	0,4969076
	EF1835	*lacA*	Energy metabolism	Biosynthesis and degradation of polysaccharides	galactose-6-phosphate isomerase, LacA subunit	NA	NA	0,5	0,2772814	0,1	0,7567397	-0,1	0,801796	0,1	0,7854763
	EF1836		Signal transduction	PTS	PTS system, IIA component, putative	1,0	0,0044915	0,3	0,5384313	0,3	0,2962508	0,0	0,936521	-0,7	0,0256228
	EF1837		Signal transduction	PTS	PTS system, IIB component, putative	NA	NA	1,2	0,0378715	0,4	0,1600129	0,0	0,9325891	-0,8	0,0210824
	EF1838		Signal transduction	PTS	PTS system, IIC component	NA	NA	0,8	0,0800443	0,2	0,5944564	-0,5	0,1614936	-0,5	0,1387162
	EF1839	*lacR*	Regulatory functions	DNA interactions	lactose phosphotransferase system repressor LacR	NA	NA	0,2	0,7381703	0,3	0,3031591	0,1	0,789496	0,0	0,8940264
	EF1841		Unknown function	General	HD domain protein	0,8	0,0807863	-0,3	0,5834163	-0,2	0,5401084	1,0	0,0050285	0,1	0,7689519
	EF1843		Energy metabolism	Biosynthesis and degradation of polysaccharides	polysaccharide deacetylase family protein	NA	NA	1,1	0,0494962	NA	NA	NA	NA	-0,1	0,6802661
	EF1844		Hypothetical proteins		hypothetical protein	NA	NA	0,3	0,5252851	NA	NA	NA	NA	0,2	0,5495378
	EF1846		Hypothetical proteins		hypothetical protein	NA	NA	NA	NA	NA	NA	NA	NA	NA	NA
	EF1847		DNA metabolism	DNA replication, recombination, and repair	site-specific recombinase, phage integrase family	0,7	0,064399	0,2	0,6046096	0,3	0,3080322	0,4	0,2786325	0,3	0,3054715
	EF1848		Hypothetical proteins		hypothetical protein	NA	NA	0,7	0,2383624	-0,1	0,6961588	0,4	0,3667463	0,3	0,3040393
	EF1849		Hypothetical proteins	Conserved	conserved hypothetical protein	NA	NA	-0,2	0,6902717	NA	NA	NA	NA	-0,2	0,5590693
	EF1850		Hypothetical proteins	Conserved	conserved hypothetical protein	NA	NA	NA	NA	NA	NA	NA	NA	-0,1	0,8736409
	EF1851		Energy metabolism	Biosynthesis and degradation of polysaccharides	glycosyl hydrolase, family 35	NA	NA	-0,5	0,3125572	0,1	0,7388815	0,0	0,9560679	0,0	0,8959766
	EF1853		Hypothetical proteins		hypothetical protein	NA	NA	NA	NA	NA	NA	NA	NA	NA	NA
	EF1855		Mobile and extrachromosomal element functions	Transposon functions	transposase, IS256 family	-1,4	0,0014604	-0,7	0,12658	-0,6	0,0506302	0,1	0,7506362	0,2	0,4530634
	EF1857		Disrupted reading frame		transposase, truncation	NA	NA	-0,6	0,1664771	-0,1	0,7397365	-0,1	0,8639113	-0,2	0,5698008
	EF1858	*panD*	Biosynthesis of cofactors, prosthetic groups, carriers	Pantothenate and coenzyme A	aspartate 1-decarboxylase	-1,8	**0,0000015**	-0,6	0,1674506	-0,3	0,2699807	-0,4	0,238088	-0,4	0,1873044

Operon[a)]	Locus	Gene	Functional category	Subcategory	Putative function	Blood[b)]	P-value[c)]	YTB_5[b)]	P-value[c)]	YTB_15[b)]	P-value[c)]	YTB_30[b)]	P-value[c)]	YTB_60[b)]	P-value[c)]
	EF1859	*panC*	Biosynthesis of cofactors, prosthetic groups, carriers	Pantothenate and coenzyme A	pantoate--beta-alanine ligase	-1,2	0,0014582	-0,3	0,5557378	-0,4	0,2163331	-0,1	0,6814942	-0,2	0,5216925
	EF1860	*panB*	Biosynthesis of cofactors, prosthetic groups, carriers	Pantothenate and coenzyme A	3-methyl-2-oxobutanoate hydroxymethyltransferase	-0,4	0,2338069	-0,1	0,8068343	-0,2	0,5032742	0,0	0,9803523	0,1	0,7475685
	EF1861		Hypothetical proteins	Domain	conserved domain protein	-0,1	0,7579643	0,4	0,4331463	-0,1	0,7649941	0,2	0,6439505	0,2	0,5704467
	EF1863		Signal transduction	Two-component systems	sensor histidine kinase	NA	NA	-0,7	0,2467736	NA	NA	-0,7	0,0442221	0,5	0,1622667
	EF1864		Regulatory functions	DNA interactions	DNA-binding response regulator	NA	NA	-0,6	0,2243192	0,0	0,9188848	0,6	0,0858385	0,5	0,234731
	EF1867		Transport and binding proteins	Unknown substrate	permease, putative	NA	NA	0,0	0,9462367	-0,6	0,0519364	0,0	0,9620158	0,3	0,4011726
	EF1868		Transport and binding proteins	Unknown substrate	ABC transporter, ATP-binding protein	NA	NA	1,0	0,030205	NA	NA	NA	NA	0,1	0,7066007
	EF1869		Transport and binding proteins	Unknown substrate	permease, putative	NA	NA	NA	NA	NA	NA	NA	NA	NA	NA
	EF1871		Energy metabolism	Electron transport	thioredoxin family protein	NA	NA	0,4	0,442782	NA	NA	NA	NA	0,4	0,2084926
	EF1872		Hypothetical proteins	Domain	conserved domain protein	0,2	0,5806543	0,2	0,7312625	0,0	0,9296472	0,3	0,4446888	0,6	0,0567165
	EF1874		Mobile and extrachromosomal element functions	Transposon functions	transposase, IS256 family	NA	NA	0,1	0,7517201	NA	NA	0,2	0,6418141	0,1	0,6642235
	EF1875		Hypothetical proteins	Conserved	conserved hypothetical protein	NA	NA	NA	NA	NA	NA	NA	NA	0,2	0,5874794
	EF1876		Cell envelope	Other	lipoprotein, NLP/P60 family	NA	NA	NA	NA	NA	NA	NA	NA	NA	NA
	EF1877		Cell envelope	Other	membrane protein, putative	0,7	0,0512496	0,3	0,508397	-0,4	0,3453442	0,3	0,5422467	0,0	0,9770828
	EF1878		Unknown function	General	ATP/GTP-binding protein, putative	-0,4	0,2415647	-0,4	0,3578956	-0,3	0,3048523	0,4	0,2947085	0,3	0,3449547
	EF1879		Hypothetical proteins	Conserved	conserved hypothetical protein	-0,2	0,6294021	-0,4	0,4063763	0,2	0,444196	0,5	0,1521435	0,4	0,2081004
	EF1880		Hypothetical proteins		hypothetical protein	NA	NA	0,1	0,8533731	NA	NA	0,3	0,3888508	0,5	0,164244
	EF1881		Hypothetical proteins	Domain	conserved domain protein	NA	NA	NA	NA	NA	NA	NA	NA	0,6	0,0921354
	EF1882		Hypothetical proteins	Conserved	conserved hypothetical protein	NA	NA	NA	NA	NA	NA	NA	NA	0,7	0,0991008
	EF1883		Hypothetical proteins	Conserved	conserved hypothetical protein	1,2	0,0009088	0,4	0,5217998	-0,2	0,550658	NA	NA	0,3	0,3236114
	EF1884		Mobile and extrachromosomal element functions	Transposon functions	transposase, IS116/IS110/IS902 family	0,6	0,0947163	0,1	0,8787674	-0,4	0,2087508	0,1	0,6846568	0,1	0,8464895
	EF1885		Hypothetical proteins		hypothetical protein	NA	NA	-0,6	0,2379562	NA	NA	NA	NA	0,2	0,4949638
	EF1886		Regulatory functions	DNA interactions	transcriptional regulator, Cro/CI family	NA	NA	0,7	0,1387183	NA	NA	0,1	0,7229448	0,1	0,6928404
	EF1887		Hypothetical proteins	Conserved	conserved hypothetical protein	NA	NA	-0,1	0,785794	NA	NA	0,2	0,7062377	0,4	0,2396911
	EF1888		Hypothetical proteins		hypothetical protein	NA	NA	NA	NA	NA	NA	NA	NA	NA	NA
	EF1889		Hypothetical proteins	Domain	conserved domain protein	0,6	0,1024611	0,6	0,2016368	0,1	0,7848182	0,3	0,3000042	0,2	0,5604437
	EF1890		Hypothetical proteins	Domain	conserved domain protein	0,6	0,1482599	0,0	0,9166344	-0,3	0,2907237	-0,2	0,5102152	0,3	0,4382224
	EF1892		Cellular processes	Cell division	FtsK/SpoIIIE family protein	NA	NA	0,4	0,3755944	NA	NA	0,3	0,5026973	0,2	0,5381229
	EF1893		Hypothetical proteins		hypothetical protein	NA	NA	NA	NA	NA	NA	NA	NA	0,3	0,3838726
	EF1894		Hypothetical proteins	Conserved	conserved hypothetical protein	NA	NA	0,1	0,8533363	NA	NA	NA	NA	NA	NA
	EF1895		Hypothetical proteins	Conserved	conserved hypothetical protein	NA	NA	-0,3	0,6412749	NA	NA	NA	NA	0,2	0,4887119
	EF1896		Cell envelope	Other	cell wall surface anchor family protein	NA	NA	-0,3	0,5175092	NA	NA	NA	NA	0,0	0,9679349
	EF1897		Hypothetical proteins		hypothetical protein	0,1	0,9008074	0,3	0,4971704	-0,1	0,8598049	0,7	0,036506	0,4	0,2675429
	EF1898	*rplS*	Protein synthesis	Ribosomal proteins: synthesis and modification	ribosomal protein L19	-2,6	**0**	-0,4	0,4171047	-0,2	0,5361114	-0,9	0,004842	0,0	0,9755673
	EF1899	*trmD*	Protein synthesis	tRNA and rRNA base modification	tRNA (guanine-N1)-methyltransferase	-0,9	0,0173538	-0,9	0,0461862	-0,3	0,3883691	-0,2	0,5229024	-0,1	0,6753743
	EF1900	*rimM*	Transcription	RNA processing	16S rRNA processing protein RimM	-0,8	0,0274038	-1,0	0,0244418	-0,2	0,4638896	0,3	0,3814534	0,0	0,9252302
	EF1901		Transport and binding proteins	Cations and iron carrying compounds	Mn2+/Fe2+ transporter, NRAMP family	1,4	0,0001621	0,6	0,1849281	0,2	0,6154623	0,1	0,794893	0,0	0,9142828
	EF1902		Unknown function	Enzymes of unknown specificity	glyoxylase family protein	1,1	0,0037908	0,5	0,2929426	0,2	0,5603331	0,6	0,0916599	-0,3	0,3857693
	EF1903		Hypothetical proteins	Conserved	conserved hypothetical protein	1,9	**0,0000003**	2,0	0,0000176	0,2	0,5332243	0,5	0,1560222	0,8	0,0227331
	EF1904		Fatty acid and phospholipid metabolism	Degradation	glycerophosphoryl diester phosphodiesterase family protein	-0,4	0,2832072	1,1	0,0188113	0,1	0,7730191	0,3	0,4353867	0,4	0,221938
	EF1905		Hypothetical proteins		hypothetical protein	0,7	0,0952799	0,2	0,700383	0,3	0,2730728	0,0	0,9859799	0,2	0,5142117
	EF1906		Hypothetical proteins	Conserved	conserved hypothetical protein	-0,1	0,8916951	0,6	0,2219984	0,1	0,6684104	0,2	0,586514	-0,1	0,7388516
	EF1907		Unknown function	General	maoC family protein	0,0	0,9438668	0,4	0,3535475	0,0	0,9088901	0,2	0,6226749	0,0	0,9231156
	EF1908	*murC*	Cell envelope	Biosynthesis/degradation of murein sacculus/peptidoglycan	UDP-N-acetylmuramate--alanine ligase	-2,5	**0**	-1,1	0,0190499	-0,5	0,1228348	-0,4	0,24469	-0,8	0,0134414
	EF1909		Hypothetical proteins		hypothetical protein	-0,3	0,4229432	-0,2	0,6844917	0,0	0,9298368	0,2	0,4870052	-0,1	0,8779601
	EF1910		Hypothetical proteins	Conserved	conserved hypothetical protein	-0,5	0,239688	0,3	0,5445334	-0,2	0,6087268	0,0	0,9455131	-0,1	0,8290594
	EF1911		Hypothetical proteins	Conserved	conserved hypothetical protein	-0,4	0,3617056	0,3	0,5563671	-0,2	0,6094643	-0,2	0,6158604	0,5	0,1559024
	EF1912		Unknown function	General	ROK family protein	0,1	0,7526871	0,4	0,3605567	0,1	0,8540051	0,1	0,8637178	-0,2	0,5371271
	EF1913		Hypothetical proteins	Conserved	conserved hypothetical protein TIGR00278	0,2	0,5407679	0,3	0,5911097	0,1	0,7237281	0,1	0,6614861	-0,3	0,3619623
	EF1914		Hypothetical proteins		hypothetical protein	NA	NA	0,1	0,8807087	-0,4	0,3318384	NA	NA	0,0	0,9994774
	EF1915		Hypothetical proteins		hypothetical protein	NA	NA	0,1	0,8031868	NA	NA	NA	NA	0,2	0,4811061
	EF1916		Unknown function	General	GTP-binding protein	-1,8	**0,0000008**	-0,9	0,045876	-0,3	0,3869816	-0,2	0,5147421	0,0	0,9890186
	EF1917	*clpX*	Protein fate	Degradation of proteins, peptides, and glycopeptides	ATP-dependent Clp protease, ATP-binding subunit ClpX	-1,6	0,0000196	0,0	0,9605116	-0,1	0,6837231	-0,2	0,626645	-0,1	0,8793932
	EF1918		Hypothetical proteins	Conserved	conserved hypothetical protein	1,3	0,0002624	0,7	0,142182	0,2	0,55047	0,3	0,362976	-0,1	0,8572267
	EF1919		Unknown function	Enzymes of unknown specificity	acetyltransferase, GNAT family	1,9	**0,0000001**	1,2	0,0304407	0,2	0,4574066	-0,9	0,0088884	-1,3	0,0000485
	EF1920		Transport and binding proteins	Carbohydrates, organic alcohols, and acids	C4-dicarboxylate anaerobic carrier	1,5	0,000026	1,5	0,0009042	0,3	0,3978321	-0,8	0,0128644	-1,7	**0,0000004**
	EF1921		Purines, pyrimidines, nucleosides, and nucleotides	Salvage of nucleosides and nucleotides	inosine-uridine preferring nucleoside hydrolase	1,0	0,0047356	1,2	0,0083661	0,2	0,586237	-0,7	0,0284636	-1,3	0,0000719
	EF1922		Regulatory functions	DNA interactions	transcriptional regulator, LacI/carbohydrate kinase, PfkB	-0,4	0,4155373	0,2	0,6276328	-0,1	0,7475705	0,1	0,6584222	0,2	0,4694098
	EF1923		Hypothetical proteins		hypothetical protein	NA	NA	0,6	0,1839951	-0,4	0,290046	NA	NA	0,1	0,7720515
	EF1925		Hypothetical proteins		hypothetical protein	-0,6	0,1689869	0,5	0,2848503	-0,1	0,8610315	0,2	0,5635024	0,4	0,2381243
	EF1926		Hypothetical proteins	Conserved	conserved hypothetical protein	0,8	0,0222872	0,3	0,5743159	-0,1	0,7614941	0,1	0,6602364	0,3	0,4436891
	EF1927	*glpF*	Transport and binding proteins	Other	glycerol uptake facilitator protein	6,8	**0**	2,5	**0,0000001**	2,6	**0**	3,8	**0**	-0,2	0,5132738
	EF1928		Energy metabolism	Other	alpha-glycerophosphate oxidase	5,6	**0**	2,7	**0**	2,9	**0**	3,7	**0**	-0,1	0,7774123
	EF1929	*glpK*	Energy metabolism	Other	glycerol kinase	4,9	**0**	3,1	**0**	3,0	**0**	3,4	**0**	0,1	0,8343322
	EF1931		Hypothetical proteins	Conserved	conserved hypothetical protein	0,4	0,4107676	0,3	0,4804692	-0,3	0,4604629	0,4	0,2311914	0,0	0,9200327
	EF1932		Unknown function	Enzymes of unknown specificity	oxidoreductase, pyridine nucleotide-disulfide family	1,2	0,0011967	-0,6	0,1733282	-0,2	0,6105991	0,4	0,2924858	0,1	0,8123635
	EF1933		Hypothetical proteins		hypothetical protein	NA	NA	NA	NA	NA	NA	NA	NA	NA	NA
	EF1934		Hypothetical proteins	Domain	conserved domain protein	0,7	0,130019	0,0	0,9470961	0,3	0,4018588	0,4	0,2397134	-0,1	0,6871295
	EF1935		Hypothetical proteins		hypothetical protein	NA	NA	1,0	0,0390233	NA	NA	NA	NA	-0,6	0,1096156
	EF1936		Hypothetical proteins		hypothetical protein	0,3	0,3823691	0,8	0,0751318	-0,2	0,5098734	0,4	0,2302129	0,3	0,4234843
	EF1937		Hypothetical proteins	Conserved	conserved hypothetical protein	0,2	0,6644398	-0,1	0,8869685	-0,4	0,2554482	0,2	0,4687495	0,5	0,1649936
	EF1938		Transport and binding proteins	Cations and iron carrying compounds	cation-transporting ATPase, E1-E2 family	0,0	0,9863962	-0,1	0,7895702	0,0	0,9148072	-0,2	0,4823915	0,3	0,3881498
	EF1939		Hypothetical proteins	Conserved	conserved hypothetical protein	-1,2	0,006058	0,1	0,8410409	-0,2	0,6340083	-0,5	0,1403407	0,0	0,8910064
	EF1940		Regulatory functions	Other	cyclic AMP receptor protein, putative	-1,3	0,0005863	0,0	0,9825316	-0,2	0,5808492	-0,5	0,1555119	0,1	0,7642561
	EF1941		Hypothetical proteins		hypothetical protein	0,5	0,1755536	0,1	0,7870859	0,5	0,0999539	0,0	0,9381282	-0,1	0,731997
	EF1943		Transport and binding proteins	Other	drug resistance transporter, Bcr/CflA family protein	0,6	0,0860975	-0,1	0,8875579	0,2	0,4359561	0,2	0,5995961	0,1	0,7923615
	EF1944		Hypothetical proteins		hypothetical protein	NA	NA	NA	NA	NA	NA	NA	NA	NA	NA
	EF1945		Cell envelope	Other	membrane protein, putative	-0,3	0,5383236	0,1	0,7498113	-0,4	0,2614094	-0,4	0,2554619	-0,1	0,7141403
	EF1946		Hypothetical proteins		hypothetical protein	NA	NA	NA	NA	NA	NA	NA	NA	NA	NA
	EF1947		Hypothetical proteins		hypothetical protein	0,5	0,2241047	0,0	0,9210852	-0,1	0,645877	0,1	0,8523572	-0,7	0,0309353
	EF1948		Hypothetical proteins	Conserved	conserved hypothetical protein	NA	NA	NA	NA	0,6	0,0562236	NA	NA	-0,1	0,6630964
	EF1949		Hypothetical proteins	Conserved	conserved hypothetical protein	1,6	0,0003859	0,1	0,8736339	0,9	0,0049117	0,6	0,0720828	-1,7	**0,0000006**
	EF1950	*mphD*	Unknown function	General	mocD family protein, putative	NA	NA	NA	NA	0,4	0,2325233	0,3	0,3327573	-1,8	**0,0000001**

Operon[a]	Locus	Gene	Functional category	Subcategory	Putative function	Blood[b]	P-value[c]	YTB_5[b]	P-value[c]	YTB_15[b]	P-value[c]	YTB_30[b]	P-value[c]	YTB_60[b]	P-value[c]
	EF1951	*mphC*	Unknown function	General	phosphosugar-binding protein	NA	NA	1,0	0,0846496	0,4	0,2400886	0,0	0,9885692	-2,2	**0**
	EF1952	*mphB*	Signal transduction	PTS	PTS system, IID component	NA	NA	0,8	0,0895611	0,3	0,3519964	-0,1	0,8545064	-2,1	**0**
	EF1953	*mphA*	Signal transduction	PTS	PTS system, IIC component	NA	NA	NA	NA	NA	NA	0,0	0,9949164	-2,1	**0**
	EF1955	*mphR*	Regulatory functions	Protein interactions	sigma-54 dependent DNA-binding response regulator	NA	NA	0,2	0,662209	0,5	0,1050473	0,4	0,2238471	-0,4	0,2117265
	EF1956		Hypothetical proteins		hypothetical protein	1,1	0,0026392	0,0	0,9774569	0,2	0,5822831	0,2	0,6532447	0,2	0,5719426
	EF1958		Purines, pyrimidines, nucleosides, and nucleotides	Nucleotide and nucleoside interconversions	deoxyguanosinetriphosphate triphosphohydrolase, putative	-0,1	0,8045724	0,6	0,1909916	1,0	0,0015233	0,6	0,0619806	0,3	0,3206772
	EF1959		Hypothetical proteins	Domain	conserved domain protein	NA	NA	0,0	0,9873327	NA	NA	0,1	0,8363519	0,1	0,6646127
	EF1961	*eno*	Energy metabolism	Glycolysis/gluconeogenesis	enolase	-1,3	0,0005724	-0,3	0,4744945	-0,4	0,205059	-0,1	0,8403232	0,5	0,1393864
	EF1962	*tpiA*	Energy metabolism	Glycolysis/gluconeogenesis	triosephosphate isomerase	-0,8	0,0262809	-0,2	0,6046459	-0,6	0,0552922	0,1	0,8425725	0,7	0,0323269
	EF1963	*pgk*	Energy metabolism	Glycolysis/gluconeogenesis	phosphoglycerate kinase	-0,9	0,0179638	-0,5	0,3131989	-0,5	0,0973842	0,1	0,8494469	0,7	0,0435763
	EF1964	*gap-2*	Energy metabolism	Glycolysis/gluconeogenesis	glyceraldehyde 3-phosphate dehydrogenases	-1,2	0,001056	-0,3	0,5254488	-0,8	0,0086307	0,3	0,3211756	0,7	0,0383162
	EF1965		Regulatory functions	Other	transcriptional regulator, SorC family	0,3	0,4112675	-0,8	0,0831624	0,1	0,7171994	1,3	0,0001409	0,0	0,9663906
	EF1966		Unknown function	General	YitT family protein	0,2	0,6912883	-0,8	0,1039327	0,4	0,2466639	0,9	0,0097099	0,1	0,8240843
	EF1967		Hypothetical proteins	Conserved	conserved hypothetical protein	-2,0	**0,0000002**	-0,6	0,2261319	-0,1	0,6621114	0,3	0,3769613	0,3	0,3583008
	EF1968		Hypothetical proteins	Conserved	conserved hypothetical protein	-0,1	0,743496	-0,6	0,1683237	-0,6	0,0614196	0,3	0,3241597	0,3	0,4235852
	EF1969		Biosynthesis of cofactors, prosthetic groups, carriers	Thiamine	phosphomethylpyrimidine kinase, putative	NA	NA	NA	NA	NA	NA	NA	NA	NA	NA
	EF1970	*aspS*	Protein synthesis	tRNA aminoacylation	aspartyl-tRNA synthetase	-1,8	**0,0000009**	-0,9	0,051743	-0,3	0,4061746	-0,6	0,0598427	0,0	0,8841951
	EF1971	*hisS*	Protein synthesis	tRNA aminoacylation	histidyl-tRNA synthetase	-3,1	**0**	-1,2	0,0083437	-0,1	0,7154312	-0,1	0,7600878	0,0	0,9526575
	EF1972		Hypothetical proteins		hypothetical protein	-1,2	0,0011182	-0,5	0,2913962	0,5	0,1106759	0,6	0,1027217	0,6	0,0772377
	EF1973		Hypothetical proteins	Conserved	conserved hypothetical protein TIGR00256	-1,1	0,0021745	-0,3	0,4926815	0,1	0,6447013	0,5	0,105995	0,1	0,7323713
	EF1974	*relA*	Cellular processes	Adaptations to atypical conditions	GTP pyrophosphokinase	-0,5	0,1682187	-0,3	0,5179525	0,0	0,9104547	0,3	0,4177161	0,4	0,201991
	EF1975		Hypothetical proteins	Conserved	conserved hypothetical protein TIGR00046	NA	NA	NA	NA	NA	NA	NA	NA	NA	NA
	EF1976	*prmA*	Protein synthesis	Ribosomal proteins: synthesis and modification	ribosomal protein L11 methyltransferase	-1,1	0,0185125	-0,8	0,0818726	0,1	0,7861383	-0,1	0,8627851	0,1	0,8784765
	EF1977		Hypothetical proteins	Conserved	conserved hypothetical protein	-1,5	0,0000539	-0,9	0,0411579	-0,1	0,7973716	0,5	0,1696411	0,2	0,650647
	EF1978		DNA metabolism	DNA replication, recombination, and repair	DNA-3-methyladenine glycosylase	-0,6	0,1186332	-1,0	0,0277199	0,3	0,3243253	0,6	0,0943277	0,2	0,5925896
	EF1979		Unknown function	General	ATPase, AAA family	1,6	**0,0000152**	0,6	0,2184168	0,4	0,2198642	0,3	0,3800427	0,5	0,1700067
	EF1980		Hypothetical proteins		hypothetical protein	0,8	0,0210681	0,0	0,9816408	0,3	0,2693964	0,9	0,0089093	0,5	0,1340134
	EF1981		Hypothetical proteins		hypothetical protein	NA	NA	0,2	0,7480531	NA	NA	0,7	0,0326434	0,1	0,6636766
	EF1982		Cellular processes	Adaptations to atypical conditions	universal stress protein family	NA	NA	NA	NA	NA	NA	NA	NA	NA	NA
	EF1983	*ackA*	Energy metabolism	Fermentation	acetate kinase	-1,3	0,0004248	-0,7	0,1535758	0,4	0,237418	-0,1	0,7381635	0,2	0,4900468
	EF1985		Hypothetical proteins		hypothetical protein	1,0	0,0118395	NA	NA	NA	NA	NA	NA	0,0	0,906871
	EF1986	*comG6*	Cellular processes	DNA transformation	competence protein	NA	NA	NA	NA	NA	NA	0,7	0,0759916	0,3	0,4019121
	EF1987		Hypothetical proteins		hypothetical protein	NA	NA	NA	NA	NA	NA	NA	NA	0,4	0,2052452
	EF1988		Hypothetical proteins		hypothetical protein	NA	NA	NA	NA	NA	NA	NA	NA	NA	NA
	EF1989	*hemH*	Biosynthesis of cofactors, prosthetic groups, carriers	Heme, porphyrin, and cobalamin	ferrochelatase	NA	NA	-0,3	0,5422627	NA	NA	-0,2	0,5247142	0,5	0,1980995
	EF1990		Hypothetical proteins		hypothetical protein	NA	NA	NA	NA	NA	NA	NA	NA	NA	NA
	EF1991	*cspC*	Cellular processes	Adaptations to atypical conditions	cold shock protein CspC	2,0	**0**	-0,2	0,6022344	1,7	**0,0000001**	1,9	**0**	2,2	**0**
	EF1992		Mobile and extrachromosomal element functions	Prophage functions	endolysin	1,0	0,008158	0,0	0,9308849	NA	NA	NA	NA	0,3	0,360786
	EF1993		Mobile and extrachromosomal element functions	Prophage functions	holin	NA	NA	0,5	0,3769823	NA	NA	NA	NA	0,0	0,9518065
	EF1994		Hypothetical proteins	Conserved	conserved hypothetical protein	NA	NA	0,6	0,2232415	NA	NA	NA	NA	0,1	0,8441963
	EF1995		Hypothetical proteins		hypothetical protein	NA	NA	NA	NA	NA	NA	NA	NA	NA	NA
	EF2001		Mobile and extrachromosomal element functions	Prophage functions	minor structural protein	NA	NA	NA	NA	NA	NA	NA	NA	0,0	0,9136656
	EF2002		Mobile and extrachromosomal element functions	Prophage functions	tail protein, putative	NA	NA	NA	NA	NA	NA	NA	NA	NA	NA
	EF2003		Mobile and extrachromosomal element functions	Prophage functions	tape measure protein, putative	NA	NA	NA	NA	NA	NA	NA	NA	NA	NA
	EF2004		Hypothetical proteins	Domain	conserved domain protein	NA	NA	0,5	0,2697644	NA	NA	NA	NA	0,4	0,2815584
	EF2005		Mobile and extrachromosomal element functions	Prophage functions	major tail protein, putative	NA	NA	0,3	0,4814541	NA	NA	NA	NA	0,2	0,554439
	EF2006		Hypothetical proteins		hypothetical protein	NA	NA	0,2	0,7000284	NA	NA	NA	NA	NA	NA
	EF2007		Hypothetical proteins		hypothetical protein	NA	NA	NA	NA	NA	NA	NA	NA	0,0	0,9656421
	EF2008		Protein synthesis	Ribosomal proteins: synthesis and modification	ribosomal protein L23, putative	NA	NA	NA	NA	NA	NA	NA	NA	NA	NA
	EF2010		Hypothetical proteins		hypothetical protein	NA	NA	NA	NA	NA	NA	NA	NA	NA	NA
	EF2011		Hypothetical proteins		hypothetical protein	NA	NA	0,8	0,1528859	NA	NA	NA	NA	-0,2	0,6168477
	EF2012		Hypothetical proteins	Conserved	conserved hypothetical protein	NA	NA	0,0	0,9348654	NA	NA	0,4	0,3247564	NA	NA
	EF2013		Hypothetical proteins		hypothetical protein	NA	NA	NA	NA	NA	NA	NA	NA	0,2	0,620585
	EF2014		Unknown function	General	coenzyme F420 hydrogenase domain protein	NA	NA	0,3	0,490184	NA	NA	0,2	0,6412317	0,3	0,4175236
	EF2015		Mobile and extrachromosomal element functions	Prophage functions	minor head protein, putative	NA	NA	0,5	0,3683199	-0,1	0,7762295	NA	NA	0,3	0,4261954
	EF2016		Mobile and extrachromosomal element functions	Prophage functions	portal protein	NA	NA	0,9	0,1145252	NA	NA	NA	NA	0,3	0,327094
	EF2017		Mobile and extrachromosomal element functions	Prophage functions	terminase, large subunit, putative	-0,1	0,8140692	0,0	0,9220137	-0,3	0,3990987	-0,3	0,4348287	0,1	0,6662314
	EF2018		Hypothetical proteins	Conserved	conserved hypothetical protein	NA	NA	0,7	0,1130358	NA	NA	0,0	0,9081222	0,0	0,891162
	EF2019		Hypothetical proteins		hypothetical protein	NA	NA	0,5	0,3238462	-0,1	0,650194	0,1	0,7942924	0,2	0,4923548
	EF2020		Hypothetical proteins		hypothetical protein	NA	NA	0,6	0,1707251	NA	NA	NA	NA	0,3	0,3190873
	EF2021		Hypothetical proteins		hypothetical protein	NA	NA	0,9	0,1327156	NA	NA	NA	NA	0,3	0,3899178
	EF2022		Hypothetical proteins	Domain	conserved domain protein	1,0	0,0054538	0,2	0,6887061	-0,4	0,2254632	-0,1	0,6696364	0,1	0,6889548
	EF2023		Hypothetical proteins		hypothetical protein	NA	NA	0,7	0,2155527	NA	NA	0,1	0,7629632	-0,2	0,5373981
	EF2024		Regulatory functions	DNA interactions	transcriptional regulator, ArpU family	NA	NA	1,0	0,0690558	NA	NA	0,4	0,1971539	0,1	0,7498471
	EF2025		Hypothetical proteins		hypothetical protein	-0,4	0,4016174	-0,2	0,6632443	-0,1	0,6978688	0,0	0,9438613	0,2	0,6490772
	EF2027		Hypothetical proteins	Conserved	conserved hypothetical protein	NA	NA	0,8	0,1663199	NA	NA	NA	NA	0,2	0,5479441
	EF2028		Hypothetical proteins		hypothetical protein	1,3	0,0003352	1,1	0,06372	-0,2	0,5080791	NA	NA	0,4	0,2083621
	EF2031		Hypothetical proteins	Conserved	conserved hypothetical protein	NA	NA	0,9	0,0956027	0,2	0,6780384	NA	NA	0,0	0,989303
	EF2032		Hypothetical proteins		hypothetical protein	NA	NA	NA	NA	NA	NA	NA	NA	0,4	0,2081758
	EF2033		Hypothetical proteins		hypothetical protein	NA	NA	NA	NA	NA	NA	NA	NA	NA	NA
	EF2034		Hypothetical proteins		hypothetical protein	1,1	0,0171975	0,2	0,6019733	0,0	0,9277833	0,7	0,037998	0,5	0,1579692
	EF2036		Hypothetical proteins		hypothetical protein	NA	NA	NA	NA	NA	NA	NA	NA	NA	NA
	EF2037		Regulatory functions	Other	antirepressor, putative	NA	NA	1,1	0,0468438	0,5	0,1437122	0,7	0,0565459	0,1	0,7045429
	EF2038		Hypothetical proteins		hypothetical protein	NA	NA	NA	NA	NA	NA	NA	NA	NA	NA
	EF2039		Hypothetical proteins	Conserved	conserved hypothetical protein	1,2	0,0011294	0,0	0,9542057	-0,2	0,5939773	0,2	0,5598771	0,2	0,5419858
	EF2040		Regulatory functions	DNA interactions	transcriptional regulator, Cro/CI family	1,4	0,0024125	0,0	0,98371	-0,1	0,6756286	0,0	0,9910583	0,2	0,4676367
	EF2041		Hypothetical proteins		hypothetical protein	NA	NA	-0,1	0,7662067	-0,2	0,501516	-0,1	0,7391445	0,1	0,7116844
	EF2042		Hypothetical proteins	Domain	conserved domain protein	-1,5	0,0000818	-0,3	0,5217058	0,1	0,8198716	0,0	0,9233759	0,3	0,3696405
	EF2043		Mobile and extrachromosomal element functions	Prophage functions	site-specific recombinase, phage integrase family	-1,1	0,0033671	-0,6	0,1781229	0,5	0,153845	0,1	0,7892893	0,0	0,8831427
	EF2044	*comG3*	Cellular processes	DNA transformation	competence protein	NA	NA	0,1	0,8565822	NA	NA	NA	NA	0,5	0,1641819
	EF2045	*comG2*	Cellular processes	DNA transformation	competence protein	NA	NA	0,3	0,5742939	NA	NA	NA	NA	0,4	0,2241402
	EF2046	*comG1*	Cellular processes	DNA transformation	competence protein	NA	NA	NA	NA	0,0	0,9201522	NA	NA	0,7	0,0300105
	EF2047		Transport and binding proteins	Amino acids, peptides and amines	amino acid permease family protein	-0,1	0,8850919	-1,1	0,0222181	-0,3	0,3137774	-0,1	0,7148947	0,3	0,380642

Operon[a]	Locus	Gene	Functional category	Subcategory	Putative function	Blood[b]	P-value[c]	YTB_5[b]	P-value[c]	YTB_15[b]	P-value[c]	YTB_30[b]	P-value[c]	YTB_60[b]	P-value[c]
	EF2048		Hypothetical proteins	Conserved	conserved hypothetical protein TIGR00048	NA	NA	-0,8	0,1425998	NA	NA	-0,8	0,0244701	-0,1	0,7958859
	EF2049		Transport and binding proteins	Unknown substrate	ABC transporter, permease protein, putative	NA	NA	0,8	0,0776596	0,5	0,106952	0,0	0,89109	-0,8	0,0230562
	EF2050		Transport and binding proteins	Unknown substrate	ABC transporter, ATP-binding protein	NA	NA	0,1	0,854789	-0,4	0,2144974	-0,3	0,3926825	-0,2	0,6070116
	EF2051		Regulatory functions	DNA interactions	transcriptional regulator, GntR family	0,3	0,3831075	0,2	0,6780083	0,4	0,2131974	0,8	0,0111881	0,4	0,287343
	EF2052		Cellular processes	Cell division	cell division protein, FtsK/SpoIIIE family	-0,3	0,3616278	-0,7	0,1584931	0,0	0,8863101	0,2	0,5423006	0,2	0,4716721
	EF2055		Unknown function	Enzymes of unknown specificity	oxidoreductase, pyridine nucleotide-disulfide family	-2,4	**0**	-0,6	0,1604034	0,7	0,0201983	0,1	0,7644705	-0,4	0,2219775
	EF2056		Biosynthesis of cofactors, prosthetic groups, carriers	Menaquinone and ubiquinone	1,4-dihydroxy-2-naphthoate octaprenyltransferase, putative	-1,5	0,0007948	-0,4	0,3496062	0,7	0,0378297	0,2	0,462824	0,0	0,9036082
	EF2057		Biosynthesis of cofactors, prosthetic groups, carriers	Menaquinone and ubiquinone	heptaprenyl diphosphate synthase, component II, putative	-0,9	0,0130068	-0,5	0,2476992	0,0	0,9471677	0,0	0,9056592	-0,1	0,655527
	EF2058		Energy metabolism	Electron transport	transport ATP-binding protein CydD, putative	-0,9	0,0115226	0,2	0,7087341	0,2	0,5694793	-0,5	0,1037496	0,3	0,4068601
	EF2059		Energy metabolism	Electron transport	transport ATP-binding protein CydC, putative	-1,3	0,0003915	0,0	0,9913258	0,3	0,3432932	-0,5	0,1189393	0,5	0,1572192
	EF2060	*cydB*	Energy metabolism	Electron transport	cytochrome d ubiquinol oxidase, subunit II	0,3	0,3460797	0,2	0,7269421	0,2	0,4931117	-0,2	0,4981341	0,2	0,5341328
	EF2061	*cydA*	Energy metabolism	Electron transport	cytochrome d ubiquinol oxidase, subunit I	-0,1	0,7696726	-0,3	0,523799	0,1	0,6496136	0,1	0,7106577	0,1	0,7552601
	EF2062		Hypothetical proteins		hypothetical protein	NA	NA	NA	NA	NA	NA	NA	NA	NA	NA
	EF2063		Regulatory functions	DNA interactions	transcriptional regulator, AraC family	-1,7	**0,0000026**	-0,6	0,2100492	0,3	0,3858878	-0,2	0,505702	0,3	0,4353731
	EF2064	*topB-1*	DNA metabolism	DNA replication, recombination, and repair	DNA topoisomerase III	0,1	0,7733631	-0,9	0,0597684	-0,2	0,6502039	0,1	0,7693932	0,2	0,582765
	EF2065		Hypothetical proteins	Conserved	conserved hypothetical protein	0,0	0,9991703	-0,2	0,5978655	-0,2	0,5880215	NA	NA	0,2	0,6307021
	EF2066		Regulatory functions	DNA interactions	transcriptional regulator, TetR family	-2,0	**0,0000062**	-0,8	0,0738988	-0,3	0,388701	-0,4	0,197976	-0,1	0,8084857
	EF2067		Hypothetical proteins	Conserved	conserved hypothetical protein TIGR00481	0,7	0,0711125	NA	NA	NA	NA	NA	NA	NA	NA
	EF2068		Transport and binding proteins	Other	multidrug resistance protein, putative	NA	NA	-0,2	0,5956796	NA	NA	0,0	0,9225729	0,3	0,3512987
	EF2069		Hypothetical proteins		hypothetical protein	NA	NA	NA	NA	NA	NA	NA	NA	NA	NA
	EF2070	*trmU*	Protein synthesis	tRNA and rRNA base modification	tRNA (5-methylaminomethyl-2-thiouridylate)-methyltransferase	-0,7	0,0462956	-0,5	0,2614181	-0,2	0,5140002	-0,4	0,2918394	-0,3	0,309063
	EF2071		Hypothetical proteins	Conserved	conserved hypothetical protein	-0,1	0,8848802	-0,1	0,7998573	-0,2	0,6308146	-0,2	0,4910194	-0,1	0,8397859
	EF2072		Unknown function	Enzymes of unknown specificity	aminotransferase, class V	-1,1	0,017974	-1,1	0,0162625	0,0	0,9916061	0,1	0,7927388	0,1	0,7889192
	EF2073	*prsA-1*	Purines, pyrimidines, nucleosides, and nucleotides	Purine ribonucleotide biosynthesis	ribose-phosphate pyrophosphokinase	-0,2	0,6803171	-0,1	0,7527391	0,6	0,0747711	0,7	0,0403864	0,1	0,8284312
	EF2074	*efaC*	Transport and binding proteins	Unknown substrate	ABC transporter, ATP-binding protein	3,6	**0**	0,7	0,1140879	1,4	0,0002619	1,9	**0**	1,0	0,0021284
	EF2075	*efaB*	Transport and binding proteins	Unknown substrate	ABC transporter, permease protein	2,4	**0**	0,5	0,3060406	0,6	0,0603045	0,8	0,0174108	0,8	0,0225725
	EF2076	*efaA*	Cellular processes	Cell adhesion	endocarditis specific antigen	1,4	0,0002688	-0,1	0,8493867	0,3	0,3604369	0,5	0,1443245	0,9	0,0045477
	EF2077		Transport and binding proteins	Unknown substrate	ABC transporter, ATP-binding protein	-2,8	**0**	-0,4	0,3434059	0,1	0,864331	0,2	0,5706057	0,0	0,9178204
	EF2078		Hypothetical proteins		hypothetical protein	NA	NA	NA	NA	NA	NA	NA	NA	NA	NA
	EF2079		Protein fate	Degradation of proteins, peptides, and glycopeptides	peptidase, M20/M25/M40 family	NA	NA	-0,2	0,7363187	0,0	0,9621609	-0,3	0,3496644	0,5	0,1412219
	EF2080		Cell envelope	Other	lipoprotein, YaeC family	NA	NA	0,1	0,9262918	NA	NA	0,4	0,2359872	0,1	0,6918983
	EF2081		Transport and binding proteins	Unknown substrate	ABC transporter, permease protein	0,4	0,2974099	-0,1	0,8244031	-0,1	0,7238757	0,1	0,8604796	0,1	0,8427462
	EF2082		Transport and binding proteins	Unknown substrate	ABC transporter, ATP-binding protein	NA	NA	0,6	0,2665327	NA	NA	NA	NA	0,3	0,3265823
	EF2083		Hypothetical proteins		hypothetical protein	NA	NA	NA	NA	NA	NA	0,5	0,2313364	-0,1	0,7954818
	EF2084		Hypothetical proteins		hypothetical protein	NA	NA	NA	NA	NA	NA	NA	NA	NA	NA
	EF2085		Hypothetical proteins	Conserved	conserved hypothetical protein	NA	NA	-0,4	0,4341979	-0,1	0,7272635	-0,3	0,4314361	0,3	0,3231741
	EF2086		Mobile and extrachromosomal element functions	Prophage functions	endolysin	0,0	0,947261	0,1	0,9003434	0,2	0,4837888	0,1	0,8511644	-0,3	0,3417921
	EF2087		Mobile and extrachromosomal element functions	Prophage functions	holin	0,7	0,1109926	-0,1	0,8106787	0,2	0,5483953	0,8	0,0136071	0,2	0,5318456
	EF2088		Hypothetical proteins		hypothetical protein	NA	NA	NA	NA	NA	NA	NA	NA	NA	NA
	EF2089		Hypothetical proteins		hypothetical protein	NA	NA	0,5	0,2793592	0,1	0,7913302	0,2	0,5005353	-0,9	0,0064508
	EF2090		Hypothetical proteins		hypothetical protein	NA	NA	0,2	0,6191427	0,1	0,8063636	-0,2	0,6469791	-0,3	0,345071
	EF2091		Hypothetical proteins		hypothetical protein	NA	NA	0,2	0,6444513	NA	NA	0,1	0,7147542	-0,3	0,4331987
	EF2092		Hypothetical proteins		hypothetical protein	0,8	0,0394028	0,0	0,9229525	NA	NA	0,0	0,9396608	-0,4	0,2615028
	EF2093		Unknown function	General	endolysin domain protein	-0,1	0,8616783	0,2	0,5928749	-0,3	0,3427459	0,0	0,9248148	-0,8	0,0150904
	EF2094		Hypothetical proteins		hypothetical protein	-0,1	0,7000265	0,3	0,5463691	-0,2	0,4302755	0,0	0,9853452	-1,1	0,0005588
	EF2095		Hypothetical proteins		hypothetical protein	NA	NA	0,6	0,2912024	1,3	0,0000478	1,1	0,0058349	0,6	0,0897841
	EF2096		Mobile and extrachromosomal element functions	Prophage functions	tail protein	NA	NA	0,0	0,9592417	-0,2	0,538222	-0,2	0,6428046	-0,6	0,073315
	EF2097		Hypothetical proteins		hypothetical protein	NA	NA	0,2	0,6462149	NA	NA	-0,3	0,354458	-0,3	0,318936
	EF2098		Hypothetical proteins		hypothetical protein	NA	NA	-0,3	0,5545788	0,0	0,8994684	-0,3	0,3979268	-0,5	0,1160859
	EF2099		Hypothetical proteins		hypothetical protein	NA	NA	0,0	0,9315988	-0,4	0,2282706	NA	NA	-0,3	0,3463703
	EF2100		Hypothetical proteins		hypothetical protein	NA	NA	0,1	0,8302975	NA	NA	NA	NA	-0,6	0,077181
	EF2101		Hypothetical proteins		hypothetical protein	-1,0	0,0300523	0,4	0,3826915	0,0	0,9513161	-0,1	0,8170514	-1,1	0,0009286
	EF2102		Hypothetical proteins		hypothetical protein	-1,5	0,0011729	0,2	0,6325371	-0,3	0,4765415	-0,4	0,2621115	-0,9	0,0087776
	EF2103		Hypothetical proteins		hypothetical protein	NA	NA	0,2	0,6813735	NA	NA	-0,6	0,0896124	-0,3	0,3121788
	EF2104		Hypothetical proteins		hypothetical protein	NA	NA	0,0	0,9356037	-0,5	0,1086285	-0,5	0,1603119	-0,8	0,0231324
	EF2105		Hypothetical proteins		hypothetical protein	-2,3	**0,0000002**	0,2	0,6689311	-0,1	0,6622382	-0,3	0,4161028	-1,4	0,0000296
	EF2106		Hypothetical proteins	Domain	conserved domain protein	NA	NA	0,6	0,2169258	-0,3	0,3726035	0,0	0,9988617	-0,9	0,007128
	EF2107		Hypothetical proteins		hypothetical protein	NA	NA	-0,4	0,374744	0,4	0,2841901	0,2	0,5538995	-0,4	0,182831
	EF2108		Hypothetical proteins		hypothetical protein	-1,9	0,0000353	0,0	0,9595722	0,1	0,7992553	-0,3	0,3857648	-0,8	0,0163774
	EF2109		Hypothetical proteins	Domain	conserved domain protein	NA	NA	0,3	0,5938727	NA	NA	NA	NA	-0,2	0,5842298
	EF2110		Hypothetical proteins		hypothetical protein	NA	NA	0,0	0,9357995	NA	NA	-0,5	0,1295753	-0,4	0,1881498
	EF2111		Hypothetical proteins		hypothetical protein	-1,9	0,0000322	0,0	0,9998572	-0,1	0,7696481	-0,5	0,2725212	-1,2	0,0002115
	EF2112		Hypothetical proteins		hypothetical protein	NA	NA	0,4	0,45139	-0,3	0,2908796	-0,2	0,6128388	-0,7	0,0444374
	EF2113		Hypothetical proteins	Conserved	conserved hypothetical protein	0,0	0,9830728	0,2	0,6654479	-0,3	0,3553078	-0,4	0,235299	-0,7	0,0265575
	EF2114		Mobile and extrachromosomal element functions	Prophage functions	adenine methyltransferase, putative	0,5	0,2491572	0,6	0,2133816	0,2	0,5907274	0,0	0,97665	0,1	0,6792891
	EF2115		Regulatory functions	DNA interactions	transcriptional regulator, ArpU family	NA	NA	0,4	0,3902134	NA	NA	-0,1	0,7250186	0,0	0,8963685
	EF2116		Hypothetical proteins		hypothetical protein	NA	NA	NA	NA	NA	NA	NA	NA	NA	NA
	EF2117		Hypothetical proteins	Conserved	conserved hypothetical protein	NA	NA	NA	NA	NA	NA	NA	NA	NA	NA
	EF2118		Hypothetical proteins	Domain	conserved domain protein	1,4	0,0000813	-0,3	0,5839697	-0,8	0,0110488	-0,3	0,4322553	-0,3	0,336834
	EF2119		Hypothetical proteins		hypothetical protein	NA	NA	-0,1	0,8921303	NA	NA	-0,4	0,2715905	-0,4	0,2396106
	EF2120		Hypothetical proteins	Conserved	conserved hypothetical protein	1,0	0,0101471	-0,2	0,678317	-0,6	0,0476756	-0,6	0,0774075	-0,4	0,2690805
	EF2121		Hypothetical proteins		hypothetical protein	NA	NA	NA	NA	NA	NA	NA	NA	NA	NA
	EF2122		Hypothetical proteins		hypothetical protein	NA	NA	NA	NA	NA	NA	NA	NA	NA	NA
	EF2123		Hypothetical proteins		hypothetical protein	NA	NA	0,2	0,6020226	NA	NA	-0,3	0,4928164	0,0	0,910176
	EF2124		Mobile and extrachromosomal element functions	Prophage functions	methyltransferase, putative	1,5	0,0000661	-0,6	0,2183769	-0,5	0,0888732	-0,1	0,7575956	-0,5	0,1734842
	EF2125		Hypothetical proteins		hypothetical protein	0,9	0,0186442	0,6	0,1653598	0,3	0,3127869	0,2	0,6489393	-0,2	0,5083942
	EF2126		Hypothetical proteins		hypothetical protein	1,3	0,0006427	0,4	0,3708734	-0,9	0,0036839	-0,8	0,0239329	-0,5	0,1387626
	EF2127		Hypothetical proteins		hypothetical protein	NA	NA	NA	NA	NA	NA	-0,2	0,6139551	-0,1	0,8361015
	EF2128		Hypothetical proteins	Conserved	conserved hypothetical protein	NA	NA	NA	NA	NA	NA	NA	NA	NA	NA
	EF2129		Mobile and extrachromosomal element functions	Prophage functions	DNA replication protein, putative	1,4	0,0001541	0,1	0,7910063	-0,7	0,0256298	-0,3	0,4299743	-0,5	0,1376287
	EF2130		Unknown function	General	DnaD domain protein	1,3	0,0003527	-0,2	0,6899866	-0,7	0,0272043	-0,2	0,5343693	-0,6	0,085448
	EF2131		Hypothetical proteins	Conserved	conserved hypothetical protein	1,2	0,0008054	0,2	0,6169862	-0,4	0,1949482	-0,5	0,1103765	-0,7	0,0361106
	EF2132		Mobile and extrachromosomal element functions	Prophage functions	recT protein, putative	1,0	0,009899	0,5	0,2417589	-1,2	0,0001717	-0,7	0,0276267	-0,5	0,1388581

Operon[a]	Locus	Gene	Functional category	Subcategory	Putative function	Blood[b]	P-value[c]	YTB_5[b]	P-value[c]	YTB_15[b]	P-value[c]	YTB_30[b]	P-value[c]	YTB_60[b]	P-value[c]
	EF2133		Hypothetical proteins		hypothetical protein	NA	NA	NA	NA	NA	NA	NA	NA	-0,1	0,7380709
	EF2134		Hypothetical proteins		hypothetical protein	1,5	0,0000348	-0,2	0,6531775	-0,7	0,0869482	-0,6	0,1520295	-0,1	0,7047806
	EF2135		Hypothetical proteins		hypothetical protein	1,5	0,0000514	-0,1	0,8496372	-0,3	0,403295	-0,3	0,4583369	0,1	0,6857014
	EF2136		Hypothetical proteins		hypothetical protein	NA	NA	NA	NA	NA	NA	NA	NA	NA	NA
	EF2137		Hypothetical proteins		hypothetical protein	NA	NA	NA	NA	NA	NA	NA	NA	NA	NA
	EF2138		Regulatory functions	DNA interactions	transcriptional regulator, Cro/CI family	NA	NA	NA	NA	NA	NA	NA	NA	NA	NA
	EF2139		Hypothetical proteins		hypothetical protein	NA	NA	0,4	0,5297627	-0,1	0,7694421	-0,5	0,1252948	-0,5	0,1616941
	EF2140		Hypothetical proteins	Conserved	conserved hypothetical protein	NA	NA	NA	NA	NA	NA	NA	NA	NA	NA
	EF2141		Mobile and extrachromosomal element functions	Prophage functions	transcriptional regulator, Cro/CI family	NA	NA	NA	NA	NA	NA	NA	NA	NA	NA
	EF2142		Regulatory functions	DNA interactions	transcriptional regulator, Cro/CI family	1,4	0,0001108	1,3	0,006197	0,1	0,8733915	-0,2	0,6368676	0,1	0,6649296
	EF2143		Hypothetical proteins	Conserved	conserved hypothetical protein	1,8	**0,0000018**	1,1	0,0131805	0,0	0,9138402	-0,3	0,4430935	0,0	0,9517425
	EF2144		Cell envelope	Other	lipoprotein, putative	2,0	**0,0000001**	0,9	0,0541817	0,2	0,5290745	-0,2	0,5347702	-0,1	0,8191067
	EF2145		Mobile and extrachromosomal element functions	Prophage functions	site-specific recombinase, phage integrase family	NA	NA	0,1	0,8236466	NA	NA	NA	NA	0,0	0,9958501
	EF2146		Cell envelope	Other	membrane protein, putative	-1,3	0,0005551	-0,1	0,8749376	0,2	0,5262853	-0,1	0,7292212	0,1	0,8357037
	EF2147		Hypothetical proteins		hypothetical protein	NA	NA	NA	NA	NA	NA	NA	NA	NA	NA
	EF2148		Hypothetical proteins		hypothetical protein	-1,6	0,0000219	-0,9	0,0607513	0,0	0,962232	-0,2	0,5507722	0,5	0,1202303
	EF2149		Hypothetical proteins		hypothetical protein	NA	NA	-0,3	0,4808983	-0,2	0,5767544	-0,3	0,3417988	0,1	0,7117535
	EF2150		Cellular processes	Toxin production and resistance	FemAB family protein	-2,8	**0**	-0,5	0,2857504	-0,2	0,5150303	-0,4	0,2564414	0,1	0,7684044
	EF2151	*glmS*	Central intermediary metabolism	Amino sugars	glucosamine--fructose-6-phosphate aminotransferase, isomerizing	-4,4	**0**	-2,1	**0,0000057**	-1,3	0,0000274	-0,6	0,0606988	0,1	0,7575013
	EF2152		Transport and binding proteins	Cations and iron carrying compounds	cobalt transport family protein	-2,3	**0**	-1,1	0,0159143	-0,2	0,5515646	-0,6	0,0633473	0,0	0,9465981
	EF2153		Transport and binding proteins	Unknown substrate	ABC transporter, ATP-binding protein	-4,0	**0**	-1,4	0,0022983	-0,1	0,7504141	-0,2	0,5507083	-0,4	0,2383878
	EF2154		Hypothetical proteins	Conserved	conserved hypothetical protein	-1,9	**0,0000002**	-0,6	0,2229063	-0,5	0,1470122	-0,5	0,1263118	-0,2	0,6167857
	EF2156		Hypothetical proteins	Conserved	conserved hypothetical protein	-0,5	0,203999	-0,6	0,1884631	0,1	0,7362738	-0,1	0,8423405	0,1	0,8357193
	EF2157		Hypothetical proteins	Conserved	conserved hypothetical protein TIGR00159	-0,6	0,0948591	-0,7	0,1217288	0,1	0,7701674	0,1	0,8347932	0,1	0,8520164
	EF2158		Energy metabolism	Electron transport	pyruvate ferredoxin/flavodoxin oxidoreductase family protein	-3,1	**0**	-1,5	0,0008538	-1,3	0,0000259	-1,1	0,0015855	0,6	0,0510668
	EF2159	*glnA*	Amino acid biosynthesis	Glutamate family	glutamine synthetase, type I	-2,7	**0**	-1,9	0,0000439	-1,2	0,0002111	0,1	0,831606	0,2	0,6118883
	EF2160	*glnR*	Regulatory functions	DNA interactions	regulatory protein GlnR	-1,8	**0,0000015**	-1,4	0,0039558	-1,4	**0,0000136**	0,4	0,2560298	0,6	0,0753048
	EF2161		Unknown function	General	GTP-binding protein	-1,9	**0,0000151**	-0,7	0,1448839	-0,3	0,3624596	-0,3	0,3652761	0,0	0,8915218
	EF2162	*miaA*	Protein synthesis	tRNA and rRNA base modification	tRNA delta(2)-isopentenylpyrophosphate transferase	NA	NA	-0,6	0,1714694	0,0	0,9408055	0,0	0,9596132	0,1	0,7526212
	EF2163		Fatty acid and phospholipid metabolism	Degradation	glycerophosphoryl diester phosphodiesterase, putative	NA	NA	-1,4	0,0018342	0,4	0,2488028	0,6	0,057505	0,3	0,3997132
	EF2164		Cell envelope	Other	membrane protein, putative	-1,3	0,0005114	-0,4	0,409826	0,3	0,3061523	-0,2	0,6043794	0,4	0,251085
	EF2165		Energy metabolism	Sugars	NAD-dependent epimerase/dehydratase family protein	-1,2	0,0010443	-0,2	0,724114	0,1	0,8501078	-0,3	0,3484718	0,1	0,757848
	EF2166		Cell envelope	Other	membrane protein, putative	-0,9	0,0116203	-0,4	0,4003799	-0,4	0,2641287	-0,9	0,0067031	0,1	0,8409121
	EF2167		Cell envelope	Biosynthesis and degradation of surface poly/liposaccharides	glycosyl transferase, group 2 family protein	-2,1	**0**	-0,5	0,2515131	-0,2	0,4993058	-0,6	0,0869246	0,2	0,5240312
	EF2168		Cell envelope	Biosynthesis and degradation of surface poly/liposaccharides	licD1 protein, putative	NA	NA	-0,7	0,1559055	NA	NA	-0,4	0,3316847	0,2	0,6490166
	EF2169		Cell envelope	Other	membrane protein, putative	-3,6	**0**	-0,8	0,1013625	0,0	0,9262099	-0,7	0,0705797	0,0	0,9793018
	EF2170		Cell envelope	Biosynthesis and degradation of surface poly/liposaccharides	glycosyl transferase, group 2 family protein	-2,2	**0,0000007**	-1,1	0,0178758	0,3	0,3023025	0,0	0,9519841	-0,1	0,816609
	EF2171		Unknown function	Enzymes of unknown specificity	epimerase/dehydratase, putative	-1,4	0,0000732	-1,2	0,0100822	-0,6	0,0582314	-0,6	0,0710988	-0,4	0,2795632
	EF2172	*ispD*	Hypothetical proteins	Conserved	2-C-methyl-D-erythritol 4-phosphate cytidylyltransferase	-0,5	0,21618	-1,2	0,0122241	-0,4	0,1847959	-0,2	0,5493503	-0,3	0,3571921
	EF2173		Mobile and extrachromosomal element functions	Transposon functions	ISEf1, transposase	-2,9	**0**	-0,7	0,1274865	-0,2	0,5806255	-0,6	0,0585119	0,0	0,9486112
	EF2174		Hypothetical proteins	Domain	conserved domain protein	-1,7	**0,0000061**	-0,9	0,0649367	0,1	0,8070129	-0,5	0,1385939	0,4	0,2210738
	EF2175		Unknown function	General	licD-related protein	NA	NA	-1,0	0,0319852	0,2	0,6336942	-0,2	0,5966717	0,0	0,912148
	EF2176		Cell envelope	Biosynthesis and degradation of surface poly/liposaccharides	glycosyl transferase, group 2 family protein	NA	NA	-0,7	0,1553092	NA	NA	0,1	0,8674958	0,4	0,2849861
	EF2177	*epaR*	Cell envelope	Biosynthesis and degradation of surface poly/liposaccharides	bacterial sugar transferase	-2,7	**0**	-0,7	0,1433026	-0,3	0,2974113	-0,6	0,0834609	-0,1	0,8143607
	EF2178	*epaQ*	Cell envelope	Other	membrane protein, putative	-2,8	**0**	-0,3	0,4721715	-0,1	0,8224453	-0,8	0,0121716	0,2	0,5353796
	EF2179	*epaP*	Hypothetical proteins	Conserved	conserved hypothetical protein	-3,1	**0**	-0,5	0,2448953	-0,1	0,6584338	-1,0	0,0030914	0,1	0,754928
	EF2180	*epaO*	Cell envelope	Biosynthesis and degradation of surface poly/liposaccharides	glycosyl transferase, group 2 family protein	-3,0	**0**	-0,3	0,4631143	0,1	0,8143442	-0,9	0,0087145	0,2	0,5595935
	EF2181	*epaN*	Cell envelope	Biosynthesis and degradation of surface poly/liposaccharides	glycosyl transferase, group 2 family protein	-2,9	**0**	-0,8	0,0901009	0,1	0,7667486	-0,5	0,167125	0,2	0,5025186
	EF2182	*epaM*	Transport and binding proteins	Unknown substrate	ABC transporter, ATP-binding protein	-2,5	**0**	-1,1	0,0186629	0,1	0,7371867	-0,1	0,8357425	0,1	0,6719186
	EF2183	*epaL*	Transport and binding proteins	Unknown substrate	ABC transporter, permease protein	-1,9	**0,0000003**	-1,0	0,0301108	0,0	0,9261722	-0,1	0,7192614	-0,3	0,4408053
	EF2184	*epaK*	Hypothetical proteins		hypothetical protein	-0,9	0,0186025	-0,6	0,1770049	-0,2	0,4305403	-0,2	0,6978787	0,3	0,4020442
	EF2185		Mobile and extrachromosomal element functions	Transposon functions	ISEf1, transposase	-2,7	**0**	-0,6	0,2236835	-0,2	0,6013763	-0,8	0,0227158	0,0	0,9707804
	EF2186		Hypothetical proteins	Domain	conserved domain protein	-1,9	**0,0000001**	-0,5	0,3028492	0,1	0,7245098	-0,9	0,008837	0,0	0,9619193
	EF2187		Mobile and extrachromosomal element functions	Transposon functions	IS256, transposase	-1,0	0,0049185	-0,9	0,0576452	-0,2	0,6134359	0,0	0,9933042	0,1	0,7033092
	EF2188		Unknown function	General	racemase domain protein	-2,0	**0**	-0,5	0,258606	0,0	0,9219557	-0,7	0,0433557	0,2	0,6161071
	EF2189	*epaJ*	Hypothetical proteins	Conserved	conserved hypothetical protein	-2,0	**0,0000001**	-0,6	0,2158565	-0,1	0,8647333	-1,0	0,0140888	0,1	0,8169749
	EF2190	*epaI*	Cell envelope	Biosynthesis and degradation of surface poly/liposaccharides	glycosyl transferase, group 2 family protein	-1,9	**0,0000004**	-0,5	0,2718753	-0,1	0,807203	-0,7	0,08092	-0,3	0,3277548
	EF2191	*epaH*	Cell envelope	Biosynthesis and degradation of surface poly/liposaccharides	dTDP-4-dehydrorhamnose reductase	-1,4	0,0023078	-0,5	0,310569	0,2	0,521754	-0,8	0,0259255	0,0	0,961403
	EF2192	*epaG*	Cell envelope	Biosynthesis and degradation of surface poly/liposaccharides	dTDP-glucose 4,6-dehydratase	-1,7	**0,0000055**	-0,1	0,8170064	0,1	0,8113981	-0,3	0,3863724	-0,1	0,8078128
	EF2193	*epaF*	Cell envelope	Biosynthesis and degradation of surface poly/liposaccharides	dTDP-4-dehydrorhamnose 3,5-epimerase	-1,2	0,0006616	-0,3	0,5084062	-0,2	0,5274508	-0,2	0,6127781	-0,2	0,5778911
	EF2194	*epaE*	Cell envelope	Biosynthesis and degradation of surface poly/liposaccharides	glucose-1-phosphate thymidylyltransferase	-2,2	**0**	-0,7	0,1548196	-0,1	0,6485951	-0,2	0,4823709	0,0	0,9848423
	EF2195	*epaD*	Cell envelope	Biosynthesis and degradation of surface poly/liposaccharides	glycosyl transferase, group 2 family protein	-1,9	**0,0000001**	-1,0	0,0363923	-0,2	0,5524806	-0,5	0,2653262	-0,1	0,7059553
	EF2196	*epaC*	Cell envelope	Biosynthesis and degradation of surface poly/liposaccharides	glycosyl transferase, group 2 family protein	-0,5	0,1666985	-0,8	0,0996499	-0,1	0,6573517	-0,1	0,6816118	-0,2	0,6520573
	EF2197	*epaB*	Cell envelope	Biosynthesis and degradation of surface poly/liposaccharides	glycosyl transferase, group 2 family protein	-1,8	**0,0000034**	-1,7	0,0003875	-0,1	0,7045204	0,0	0,9893712	0,0	0,9336544
	EF2198	*epaA*	Cell envelope	Biosynthesis and degradation of surface poly/liposaccharides	glycosyl transferase, group 4 family protein	-2,1	**0,000002**	-0,7	0,1489752	0,1	0,8003445	0,0	0,8837557	0,3	0,3372804
	EF2199	*yihY*	Transcription	RNA processing	YihY family protein	NA	NA	-0,1	0,8052396	-0,6	0,0430916	-0,8	0,0211811	0,6	0,0662447
	EF2200	*map*	Protein fate	Protein modification and repair	methionine aminopeptidase, type I	0,2	0,5267078	-0,1	0,8313964	0,3	0,3412763	0,3	0,357057	0,3	0,4328561
	EF2201		Energy metabolism	Electron transport	flavodoxin	-1,7	**0,0000044**	-0,8	0,10403	-0,2	0,4943158	-0,1	0,8984142	-0,2	0,4596198
	EF2202		Regulatory functions	Other	tspO protein, putative	NA	NA	0,4	0,3953963	-0,1	0,7435841	0,4	0,2088798	0,4	0,2724641
	EF2203		Regulatory functions	DNA interactions	transcriptional regulator, TetR family	1,6	**0,0000066**	0,6	0,2111212	0,4	0,2481898	0,2	0,6435703	-0,2	0,6384857
	EF2205		Hypothetical proteins	Conserved	conserved hypothetical protein	NA	NA	-0,4	0,486319	-1,0	0,0095995	-1,0	0,0023231	0,4	0,2175103
	EF2206		Unknown function	Enzymes of unknown specificity	cytidine/deoxycytidylate deaminase family protein	NA	NA	-0,3	0,5263019	-0,2	0,4779494	-0,3	0,3327279	0,7	0,0426033
	EF2207		Regulatory functions	DNA interactions	DNA-binding protein, Fis family	-0,6	0,0885098	-0,3	0,5535476	0,1	0,6488067	0,1	0,8176537	0,1	0,8508345
	EF2208		Cellular processes	Toxin production and resistance	phenazine biosynthesis protein PhzF family	-0,3	0,4650759	0,1	0,8833257	0,1	0,8107341	0,0	0,9405863	0,1	0,7622952
	EF2209		Hypothetical proteins		hypothetical protein	2,4	**0**	0,3	0,5718644	0,3	0,3669924	0,5	0,1413568	-0,1	0,8248035
	EF2210		Hypothetical proteins	Conserved	conserved hypothetical protein	0,6	0,1268626	-0,1	0,8865211	-0,2	0,5821729	-0,3	0,3175028	0,2	0,5405311
	EF2211		Hypothetical proteins	Conserved	conserved hypothetical protein TIGR01655	0,4	0,3434101	0,6	0,202236	0,3	0,3322534	0,0	0,9153678	0,1	0,6678444
	EF2212		Hypothetical proteins	Conserved	conserved hypothetical protein	-0,3	0,4376038	-0,8	0,0695242	0,1	0,7307128	0,6	0,079789	0,2	0,5625358
	EF2213		Signal transduction	PTS	PTS system, IIBC components	-0,1	0,7640555	1,4	0,0018029	0,8	0,0089547	0,3	0,3820127	-0,6	0,0709084
	EF2214		Unknown function	Enzymes of unknown specificity	glyoxylase family protein	1,2	0,0011431	-0,8	0,1048243	0,3	0,3780029	0,7	0,0285685	0,2	0,5474376
	EF2215		Hypothetical proteins	Conserved	conserved hypothetical protein	NA	NA	-0,3	0,5877276	0,1	0,636994	-0,1	0,7411905	0,1	0,7975928
	EF2216		Hypothetical proteins	Domain	conserved domain protein	-0,3	0,4813775	-0,1	0,8077077	-0,4	0,2629658	-0,2	0,4783905	0,3	0,3480024
	EF2217		Cell envelope	Biosynthesis and degradation of surface poly/liposaccharides	alpha-1,2-mannosidase, putative	-0,3	0,4616698	0,8	0,0996773	0,5	0,1366112	0,1	0,8403351	-0,6	0,0596386

Operon[a]	Locus	Gene	Functional category	Subcategory	Putative function	Blood[b]	P-value[c]	YTB_5[b]	P-value[c]	YTB_15[b]	P-value[c]	YTB_30[b]	P-value[c]	YTB_60[b]	P-value[c]
	EF2218		Regulatory functions	DNA interactions	DNA-binding response regulator, AraC family	NA	NA	-0,7	0,1429514	0,4	0,2377218	0,1	0,8517797	-0,8	0,0139943
	EF2219		Signal transduction	Two-component systems	sensor histidine kinase	0,2	0,733979	-0,1	0,9130096	-0,1	0,666777	-0,2	0,5597401	-0,6	0,051841
	EF2220		Hypothetical proteins	Conserved	conserved hypothetical protein	1,6	**0,0000079**	0,6	0,1685613	0,5	0,1454793	0,6	0,0574479	-1,7	**0,0000002**
	EF2221		Transport and binding proteins	Unknown substrate	ABC transporter, substrate-binding protein	4,1	**0**	1,7	0,0002935	1,2	0,0000945	1,6	**0,0000029**	-1,7	**0,0000003**
	EF2222		Transport and binding proteins	Unknown substrate	ABC transporter, permease protein	NA	NA	NA	NA	NA	NA	NA	NA	NA	NA
	EF2223		Transport and binding proteins	Unknown substrate	ABC transporter, permease protein	2,3	**0**	2,5	**0,0000001**	1,1	0,0002651	1,0	0,0028924	-1,8	**0**
	EF2224		Cell envelope	Other	cell wall surface anchor family protein	-0,6	0,172689	-0,2	0,6589458	0,0	0,9542877	-0,1	0,6725472	0,2	0,6456236
	EF2225		Regulatory functions	DNA interactions	transcriptional regulator, MerR family	NA	NA	NA	NA	NA	NA	NA	NA	NA	NA
	EF2226		Transport and binding proteins	Unknown substrate	ABC transporter, ATP-binding/permease protein	0,0	0,9729272	-0,1	0,8023757	-0,2	0,4579244	0,7	0,0258484	1,3	0,0000868
	EF2227		Transport and binding proteins	Unknown substrate	ABC transporter, ATP-binding/permease protein	NA	NA	0,2	0,7140427	NA	NA	0,5	0,139813	0,4	0,2285016
	EF2228		Protein synthesis	tRNA aminoacylation	tryptophanyl-tRNA synthetase	-0,4	0,3517869	-0,1	0,8341741	0,0	0,9900838	-0,2	0,5968356	0,3	0,4449463
	EF2229		Hypothetical proteins	Domain	conserved domain protein	NA	NA	NA	NA	NA	NA	NA	NA	-0,2	0,6424223
	EF2230		Hypothetical proteins		hypothetical protein	NA	NA	0,3	0,5448687	NA	NA	-0,1	0,7271324	0,0	0,8942783
	EF2231		Hypothetical proteins	Conserved	conserved hypothetical protein	0,9	0,0118454	0,4	0,4620459	-0,2	0,5655789	-0,4	0,1959207	-0,6	0,0601691
	EF2232		Transport and binding proteins	Unknown substrate	ABC transporter, permease protein	1,2	0,006804	0,7	0,2211735	-0,2	0,5932802	-0,4	0,2389622	-0,9	0,0058986
	EF2233		Transport and binding proteins	Unknown substrate	ABC transporter, permease protein	NA	NA	0,2	0,7723835	0,0	0,9419642	-0,9	0,0106753	-1,1	0,0009937
	EF2234		Transport and binding proteins	Carbohydrates, organic alcohols, and acids	sugar ABC transporter, sugar-binding protein, putative	NA	NA	0,3	0,5532367	NA	NA	-0,1	0,7782058	-0,7	0,0340247
	EF2235		Energy metabolism	Biosynthesis and degradation of polysaccharides	glucuronyl hydrolase, putative	NA	NA	0,0	0,9831504	NA	NA	-0,3	0,3144823	-0,3	0,3963971
	EF2236		Hypothetical proteins	Conserved	conserved hypothetical protein	NA	NA	0,2	0,6017673	0,6	0,0467028	-0,6	0,0807433	-0,3	0,3916505
	EF2237		Cell envelope	Other	lipoprotein, putative	NA	NA	0,1	0,8147945	NA	NA	0,3	0,4397554	NA	NA
	EF2238		Regulatory functions	DNA interactions	sugar-binding transcriptional regulator, LacI family	NA	NA	0,0	0,9587104	0,1	0,8323925	0,3	0,3234649	0,0	0,9738326
	EF2239		Hypothetical proteins		hypothetical protein	NA	NA	0,0	0,9549545	-0,4	0,3616757	-0,1	0,7487646	0,2	0,6113018
	EF2240		DNA metabolism	DNA replication, recombination, and repair	site-specific recombinase, phage integrase family	NA	NA	-0,5	0,2356164	0,4	0,3077341	-0,1	0,8423025	0,1	0,6789866
	EF2241		Hypothetical proteins	Conserved	conserved hypothetical protein	NA	NA	-0,4	0,4507974	0,9	0,0059005	0,2	0,6528492	0,2	0,6178365
	EF2243		Hypothetical proteins	Conserved	conserved hypothetical protein	NA	NA	0,1	0,861878	NA	NA	NA	NA	0,4	0,2735149
	EF2244		Hypothetical proteins	Conserved	conserved hypothetical protein	NA	NA	NA	NA	NA	NA	NA	NA	0,3	0,3193579
	EF2245		Hypothetical proteins	Conserved	conserved hypothetical protein	NA	NA	0,2	0,7534484	NA	NA	-0,2	0,5926828	0,0	0,9380421
	EF2247		Regulatory functions	Other	transcriptional regulator	NA	NA	NA	NA	NA	NA	0,2	0,6597014	0,1	0,8367688
	EF2248		Hypothetical proteins		hypothetical protein	-1,6	**0,0000083**	-0,9	0,0509329	0,3	0,4233535	0,1	0,7169268	0,1	0,8730007
	EF2249		Hypothetical proteins		hypothetical protein	NA	NA	NA	NA	NA	NA	NA	NA	0,3	0,3121535
	EF2250		Hypothetical proteins	Domain	conserved domain protein	NA	NA	-0,4	0,528361	-0,3	0,3628063	NA	NA	0,1	0,7762372
	EF2251		Hypothetical proteins		hypothetical protein	NA	NA	-0,2	0,6718068	NA	NA	NA	NA	0,1	0,8408335
	EF2252		Hypothetical proteins		hypothetical protein	NA	NA	NA	NA	NA	NA	NA	NA	-0,5	0,2677234
	EF2253		Hypothetical proteins	Conserved	conserved hypothetical protein	NA	NA	NA	NA	NA	NA	NA	NA	NA	NA
	EF2254		Hypothetical proteins	Conserved	conserved hypothetical protein	NA	NA	NA	NA	NA	NA	0,0	0,9335295	NA	NA
	EF2255		DNA metabolism	DNA replication, recombination, and repair	site-specific recombinase, phage integrase family	1,4	0,0021624	-0,1	0,7487271	1,0	0,0018532	0,9	0,0052465	-0,1	0,768779
	EF2257		Signal transduction	PTS	PTS system, IIC component, putative	NA	NA	-0,7	0,159582	0,2	0,489766	0,4	0,2504854	0,1	0,7519411
	EF2258		Hypothetical proteins	Domain	conserved domain protein	NA	NA	0,2	0,6692309	-0,1	0,7481386	0,4	0,3802222	0,2	0,520492
	EF2259		Regulatory functions	Other	phosphosugar-binding transcriptional regulator, putative	-0,6	0,1520488	-0,2	0,7241756	0,2	0,6207941	0,2	0,6309285	0,2	0,5825723
	EF2260		Hypothetical proteins	Conserved	conserved hypothetical protein	-1,2	0,0058997	-0,6	0,1789394	0,3	0,3935942	0,6	0,067184	0,2	0,5014533
	EF2261		Hypothetical proteins		hypothetical protein	NA	NA	NA	NA	NA	NA	NA	NA	NA	NA
	EF2262		Hypothetical proteins		hypothetical protein	1,2	0,0014563	-0,3	0,5713515	-0,2	0,4305612	0,1	0,7019362	0,3	0,4394202
	EF2263		Central intermediary metabolism	Other	gluconate 5-dehydrogenase, putative	NA	NA	-0,1	0,7862677	-0,2	0,4970188	-0,3	0,4188282	0,2	0,6123955
	EF2264	*kduI-2*	Energy metabolism	Biosynthesis and degradation of polysaccharides	4-deoxy-l-threo-5-hexosulose-uronate ketol-isomerase	NA	NA	0,2	0,6713813	0,2	0,6254321	-0,2	0,478577	0,0	0,9448159
	EF2265		Energy metabolism	Sugars	carbohydrate kinase, pfkB family	NA	NA	-0,2	0,7340182	0,5	0,219109	-0,2	0,4709659	-0,1	0,724042
	EF2266		Energy metabolism	Entner-Doudoroff	2-dehydro-3-deoxyphosphogluconate aldolase	NA	NA	NA	NA	NA	NA	-0,3	0,3753777	0,4	0,1811077
	EF2267		Signal transduction	PTS	PTS system, IIA component	0,4	0,3534405	-0,2	0,6854125	0,0	0,9192744	-0,1	0,8009716	-0,1	0,8515327
	EF2268		Hypothetical proteins	Conserved	conserved hypothetical protein	0,3	0,347365	-0,2	0,7100072	0,1	0,7745135	-0,5	0,1600245	-0,1	0,730702
	EF2269		Signal transduction	PTS	PTS system, IID component	NA	NA	-0,2	0,7646305	0,0	0,8975211	0,2	0,6332095	-0,1	0,7823272
	EF2270		Signal transduction	PTS	PTS system, IIC component	NA	NA	0,1	0,7898372	-0,4	0,1823018	-0,4	0,2250028	0,1	0,7728385
	EF2271		Signal transduction	PTS	PTS system, IIB component	NA	NA	-0,6	0,2693358	-0,3	0,5079452	-0,6	0,1589416	0,3	0,4336613
	EF2272		Energy metabolism	Biosynthesis and degradation of polysaccharides	glucuronyl hydrolase, putative	NA	NA	-0,2	0,7322668	0,3	0,4387871	-0,5	0,1776722	0,2	0,6438975
	EF2273		Regulatory functions	DNA interactions	transcriptional regulator, GntR family	-0,4	0,3227989	-0,7	0,1122753	0,1	0,7307756	0,0	0,9165604	0,2	0,634822
	EF2275		Hypothetical proteins		hypothetical protein	NA	NA	-0,3	0,4961386	NA	NA	0,1	0,7818506	0,2	0,6056465
	EF2276		Hypothetical proteins		hypothetical protein	NA	NA	-0,3	0,4830849	-1,1	0,0011835	-0,3	0,3169794	0,3	0,3863144
	EF2277		Hypothetical proteins	Conserved	conserved hypothetical protein	NA	NA	NA	NA	NA	NA	NA	NA	0,6	0,0736586
	EF2278		Cell envelope	Other	lipoprotein, NLP/P60 family	NA	NA	NA	NA	NA	NA	NA	NA	NA	NA
	EF2279		Cell envelope	Other	membrane protein, putative	0,3	0,5545556	-0,6	0,2982821	-0,6	0,0805275	0,1	0,8299227	0,5	0,1602588
	EF2280		Hypothetical proteins	Conserved	conserved hypothetical protein	-0,3	0,5434397	-0,4	0,3648674	-0,5	0,1189075	-0,1	0,7462715	0,6	0,0893434
	EF2281		Hypothetical proteins	Conserved	conserved hypothetical protein	0,3	0,3836729	0,1	0,8424552	0,0	0,9958434	-0,4	0,2057675	0,3	0,4179262
	EF2282		Hypothetical proteins	Domain	conserved domain protein	-0,8	0,02639	-0,9	0,0430914	-0,2	0,5352974	0,1	0,7790562	0,4	0,1751016
	EF2283		DNA metabolism	DNA replication, recombination, and repair	site-specific recombinase, resolvase family, putative	-0,3	0,3934407	-0,6	0,1784858	0,0	0,9946285	0,3	0,3434831	0,4	0,2430348
	EF2284		Hypothetical proteins		hypothetical protein	NA	NA	0,3	0,6479484	NA	NA	NA	NA	0,2	0,6294308
	EF2285		Hypothetical proteins		hypothetical protein	NA	NA	0,7	0,1615493	-0,3	0,3775079	NA	NA	0,0	0,9594832
	EF2286		Unknown function	General	ParB-like nuclease domain protein	0,8	0,0295131	0,0	0,9202376	-0,4	0,1903747	-0,3	0,4644688	-0,2	0,6435043
	EF2287		Hypothetical proteins		hypothetical protein	NA	NA	0,9	0,0443553	-0,5	0,1515025	-0,5	0,1341713	0,0	0,8974451
	EF2288		Hypothetical proteins		hypothetical protein	NA	NA	NA	NA	NA	NA	NA	NA	NA	NA
	EF2289		Hypothetical proteins		hypothetical protein	NA	NA	NA	NA	NA	NA	0,0	0,9450158	-0,3	0,4245681
	EF2290		Transcription	Transcription factors	RNA polymerase sigma-70 factor, ECF subfamily	1,2	0,0081909	0,5	0,2866165	-0,1	0,7015198	-0,3	0,5236784	0,1	0,687284
	EF2291		Regulatory functions	DNA interactions	transcriptional regulator, Cro/CI family	0,6	0,1276942	0,6	0,2159976	0,2	0,60236	0,6	0,0822888	0,4	0,217354
	EF2292		Hypothetical proteins		hypothetical protein	0,2	0,5218699	0,2	0,6459487	-0,1	0,7303216	0,1	0,702694	0,3	0,3087169
	EF2293	*vanX*	Protein fate	Degradation of proteins, peptides, and glycopeptides	D-alanyl-D-alanine dipeptidase	0,0	0,9342569	-0,1	0,8949399	0,4	0,2460857	0,7	0,0308148	0,4	0,2213526
	EF2294	*vanB*	Cellular processes	Toxin production and resistance	D-alanine--D-lactate ligase	0,6	0,1445831	0,3	0,5026837	-0,1	0,6613941	-0,1	0,7789222	0,4	0,223383
	EF2295		Cellular processes	Toxin production and resistance	D-specific alpha-keto acid dehydrogenase	NA	NA	0,8	0,1036968	NA	NA	NA	NA	0,9	0,0100092
	EF2296	*vanW*	Signal transduction	Two-component systems	vancomycin B-type resistance protein VanW	NA	NA	0,4	0,4315817	-0,8	0,0105025	-0,6	0,0622449	0,3	0,400093
	EF2297	*vanYB*	Cellular processes	Toxin production and resistance	D-alanyl-D-alanine carboxypeptidase	0,4	0,4100915	0,5	0,3716725	0,0	0,9982365	0,0	0,959827	0,1	0,6716615
	EF2298	*vanSB*	Signal transduction	Two-component systems	sensor histidine kinase VanSB	0,3	0,3950747	-0,1	0,8935972	0,0	0,9675293	-0,1	0,8816798	0,1	0,7584704
	EF2299	*vanRB*	Signal transduction	Two-component systems	DNA-binding response regulator VanRB	0,1	0,8235717	-0,2	0,6764114	0,1	0,7318184	-0,1	0,7880049	0,2	0,4788961
	EF2300		Cellular processes	Toxin production and resistance	streptomycin resistance protein, putative	-0,2	0,6461145	-0,1	0,800968	0,2	0,5519865	-0,2	0,621549	0,5	0,1614736
	EF2302		Hypothetical proteins	Conserved	conserved hypothetical protein	-1,0	0,0302232	-0,2	0,6479907	-0,2	0,5684419	-0,8	0,0233089	0,6	0,0854407
	EF2303		Hypothetical proteins	Conserved	conserved hypothetical protein	-1,3	0,0006048	0,0	0,9799341	-0,2	0,6090673	-0,9	0,007462	0,3	0,3550908
	EF2304		Regulatory functions	DNA interactions	transcriptional regulator, Cro/CI family	-0,3	0,4337451	0,0	0,9334353	0,0	0,8820205	-0,4	0,2456292	-0,2	0,5289369
	EF2305		DNA metabolism	Other	toprim domain protein	0,2	0,5099968	-0,5	0,2700641	0,4	0,2176149	-0,2	0,6265349	-0,4	0,2052302

Operon[a]	Locus	Gene	Functional category	Subcategory	Putative function	Blood[b]	P-value[c]	YTB_5[b]	P-value[c]	YTB_15[b]	P-value[c]	YTB_30[b]	P-value[c]	YTB_60[b]	P-value[c]
	EF2306		Hypothetical proteins	Conserved	conserved hypothetical protein	-0,6	0,0825728	-0,2	0,6856847	-0,2	0,6291674	-0,4	0,2414861	0,3	0,4423216
	EF2307		Hypothetical proteins	Conserved	conserved hypothetical protein	-1,4	0,0000854	-0,5	0,319618	-0,1	0,7372136	-0,5	0,1636555	0,2	0,4850213
	EF2308		Hypothetical proteins		hypothetical protein	NA	NA	NA	NA	NA	NA	NA	NA	NA	NA
	EF2309		Hypothetical proteins		hypothetical protein	-0,3	0,4777905	0,3	0,4995393	0,2	0,4675151	0,4	0,2535062	0,5	0,1488366
	EF2310		Hypothetical proteins		hypothetical protein	0,6	0,0919002	0,1	0,7484684	0,3	0,2956816	-0,2	0,6381135	0,6	0,0800747
	EF2311		Hypothetical proteins		hypothetical protein	NA	NA	0,0	0,9444238	0,0	0,8850892	-0,2	0,4996936	0,2	0,6308184
	EF2312	*topB-2*	DNA metabolism	DNA replication, recombination, and repair	DNA topoisomerase III	0,2	0,6608502	0,2	0,6631256	0,4	0,2301189	0,0	0,9086258	0,4	0,1894357
	EF2313		Hypothetical proteins		hypothetical protein	NA	NA	NA	NA	NA	NA	NA	NA	0,2	0,5888429
	EF2314		Cellular processes	Toxin production and resistance	bacteriocin, putative	0,1	0,8563581	0,4	0,3864622	0,6	0,0690691	0,0	0,9359952	0,5	0,1566472
	EF2315		Hypothetical proteins		hypothetical protein	NA	NA	0,5	0,3269493	0,1	0,665751	-0,1	0,7149426	0,7	0,0494348
	EF2316		Hypothetical proteins	Domain	conserved domain protein	0,8	0,0375889	0,0	0,9382173	0,4	0,2027906	-0,1	0,8052859	0,8	0,0192454
	EF2317		Hypothetical proteins		hypothetical protein	NA	NA	NA	NA	NA	NA	NA	NA	NA	NA
	EF2318		Protein fate	Degradation of proteins, peptides, and glycopeptides	peptidase, M23/M37 family	0,1	0,8481919	0,3	0,4753802	0,5	0,104438	-0,1	0,6761671	0,4	0,1911596
	EF2319		Hypothetical proteins		hypothetical protein	NA	NA	0,7	0,1381131	1,8	**0,0000049**	0,1	0,7287177	0,2	0,5378523
	EF2320		Mobile and extrachromosomal element functions	Plasmid functions	traE protein, putative	0,5	0,2930007	0,5	0,2802694	0,3	0,3274323	NA	NA	0,7	0,031669
	EF2321		Hypothetical proteins		hypothetical protein	1,2	0,0018543	0,2	0,6475667	0,0	0,9704728	0,1	0,7149185	0,5	0,1587405
	EF2322		Hypothetical proteins	Domain	conserved domain protein	1,1	0,0031859	0,5	0,3966258	0,5	0,0902628	0,2	0,6097369	0,6	0,0906001
	EF2324		Hypothetical proteins	Conserved	conserved hypothetical protein	NA	NA	NA	NA	NA	NA	NA	NA	0,3	0,3978674
	EF2325		Hypothetical proteins		hypothetical protein	NA	NA	0,5	0,3275912	0,3	0,4304663	0,3	0,3306627	0,3	0,4209847
	EF2326		Mobile and extrachromosomal element functions	Transposon functions	group II intron reverse transcriptase maturase	NA	NA	-0,3	0,5784489	NA	NA	NA	NA	0,5	0,125066
	EF2327		Hypothetical proteins		hypothetical protein	0,7	0,053337	-0,3	0,5285404	-0,3	0,4973415	0,1	0,8307269	0,3	0,4321699
	EF2328		Mobile and extrachromosomal element functions	Plasmid functions	TraG family protein	0,5	0,1841098	0,0	0,9265755	-0,3	0,3567009	0,3	0,4309943	0,0	0,9794037
	EF2329		Hypothetical proteins		hypothetical protein	NA	NA	0,3	0,5793551	NA	NA	NA	NA	-0,2	0,6071545
	EF2330		Hypothetical proteins		hypothetical protein	NA	NA	1,5	0,0098494	NA	NA	NA	NA	0,1	0,6981725
	EF2331		Hypothetical proteins		hypothetical protein	NA	NA	0,3	0,5414287	NA	NA	0,2	0,5612139	0,1	0,6564444
	EF2332		Hypothetical proteins	Domain	conserved domain protein	NA	NA	-0,1	0,8873596	NA	NA	0,3	0,3411455	0,4	0,2685145
	EF2333		Hypothetical proteins		hypothetical protein	1,0	0,0195109	-0,2	0,685736	NA	NA	0,6	0,0690004	-0,1	0,7602102
	EF2334		Hypothetical proteins	Domain	conserved domain protein	0,7	0,0558185	-0,4	0,4611524	NA	NA	NA	NA	0,6	0,0875681
	EF2335		Hypothetical proteins	Conserved	conserved hypothetical protein	NA	NA	0,0	0,9462315	-0,4	0,3125009	-0,1	0,8226143	0,1	0,7607001
	EF2336		Hypothetical proteins	Conserved	conserved hypothetical protein	NA	NA	NA	NA	NA	NA	NA	NA	NA	NA
	EF2337		Hypothetical proteins		hypothetical protein	NA	NA	NA	NA	NA	NA	NA	NA	NA	NA
	EF2338		Regulatory functions	DNA interactions	transcriptional regulator, Cro/CI family	NA	NA	NA	NA	NA	NA	NA	NA	NA	NA
	EF2339		Hypothetical proteins		hypothetical protein	NA	NA	NA	NA	NA	NA	NA	NA	0,1	0,8653393
	EF2340		DNA metabolism	Restriction/modification	C-5 cytosine-specific DNA methylase	NA	NA	NA	NA	NA	NA	NA	NA	NA	NA
	EF2341		Hypothetical proteins		hypothetical protein	-0,8	0,0657273	0,8	0,0953773	0,5	0,1233446	0,1	0,6723003	0,1	0,7464707
	EF2342		Hypothetical proteins		hypothetical protein	-0,4	0,2603164	0,9	0,0492288	0,3	0,3706832	0,1	0,855724	0,3	0,3885453
	EF2343		Cellular processes	Cell division	FtsK/SpoIIIE family protein	NA	NA	NA	NA	NA	NA	NA	NA	NA	NA
	EF2344		Hypothetical proteins		hypothetical protein	NA	NA	1,0	0,0909489	NA	NA	NA	NA	0,1	0,8727031
	EF2345		Hypothetical proteins	Conserved	conserved hypothetical protein	NA	NA	0,4	0,4559216	NA	NA	0,7	0,0710993	0,3	0,3523788
	EF2346		Hypothetical proteins	Conserved	conserved hypothetical protein	1,4	0,001806	-0,6	0,3087406	NA	NA	NA	NA	0,1	0,8089909
	EF2347		Cell envelope	Other	cell wall surface anchor family protein	NA	NA	-0,3	0,5705531	NA	NA	NA	NA	NA	NA
	EF2348		Hypothetical proteins		hypothetical protein	-1,4	0,002419	-0,3	0,5780538	0,2	0,48156	0,0	0,9788517	0,5	0,1735673
	EF2349		Hypothetical proteins		hypothetical protein	-1,3	0,0009712	0,1	0,8365268	0,3	0,2977936	0,2	0,567798	0,1	0,6581324
	EF2350		Regulatory functions	DNA interactions	transcriptional regulator, Cro/CI family	NA	NA	-0,8	0,1034087	0,4	0,2118078	0,5	0,1860952	0,2	0,5098345
	EF2351		Hypothetical proteins		hypothetical protein	-2,2	**0**	-0,7	0,1514718	-0,2	0,5623604	-0,4	0,2176421	0,2	0,6386395
	EF2352	*lepA*	Unknown function	General	GTP-binding protein LepA	-1,9	**0,0000001**	-0,8	0,075564	-0,3	0,3559797	-0,2	0,5215841	0,0	0,9874252
	EF2353		Unknown function	Enzymes of unknown specificity	acetyltransferase, GNAT family	-1,0	0,0072345	-0,1	0,8285957	-0,1	0,8115464	-0,1	0,7793783	-0,1	0,7747457
	EF2354		Hypothetical proteins	Conserved	conserved hypothetical protein	-0,8	0,0276776	-0,4	0,3573208	0,3	0,3286344	0,0	0,926594	0,2	0,5883524
	EF2355	*clpB*	Protein fate	Degradation of proteins, peptides, and glycopeptides	ATP-dependent Clp protease, ATP-binding subunit ClpB	2,0	**0,0000001**	0,7	0,1593758	0,1	0,8676332	0,0	0,9434942	0,1	0,8442332
	EF2357		Hypothetical proteins	Conserved	conserved hypothetical protein	0,8	0,0261559	-0,3	0,6275519	0,4	0,2434761	0,3	0,4152103	0,3	0,3104347
	EF2360		Hypothetical proteins		hypothetical protein	NA	NA	NA	NA	NA	NA	NA	NA	NA	NA
	EF2361	*purB*	Purines, pyrimidines, nucleosides, and nucleotides	Purine ribonucleotide biosynthesis	adenylosuccinate lyase	-2,1	**0**	-1,1	0,0216477	0,1	0,8362578	-0,2	0,6408184	-0,2	0,4721937
	EF2362	*purK-2*	Purines, pyrimidines, nucleosides, and nucleotides	Purine ribonucleotide biosynthesis	phosphoribosylaminoimidazole carboxylase, ATPase subunit	-1,8	**0,0000018**	-1,4	0,0025253	0,0	0,9145318	0,0	0,9509373	-0,4	0,2359579
	EF2363		Hypothetical proteins		hypothetical protein	1,1	0,002161	-0,3	0,5133603	0,1	0,751343	0,1	0,8231333	-0,2	0,6331051
	EF2364		Transport and binding proteins	Nucleosides, purines and pyrimidines	xanthine permease	-0,7	0,0639281	-1,0	0,0266637	-0,1	0,8004978	0,1	0,8456773	0,2	0,5923033
	EF2365	*xpt*	Purines, pyrimidines, nucleosides, and nucleotides	Salvage of nucleosides and nucleotides	xanthine phosphoribosyltransferase	-2,3	**0,0000001**	-1,2	0,0082854	-0,1	0,7561928	0,4	0,2360999	0,4	0,2463966
	EF2366		Hypothetical proteins	Conserved	conserved hypothetical protein	-0,3	0,4139288	-0,7	0,1424147	-0,1	0,7934604	0,4	0,2300856	0,0	0,9051168
	EF2367		Cell envelope	Biosynthesis/degradation of murein sacculus/peptidoglycan	N-acetylmuramoyl-L-alanine amidase, family 4	-0,1	0,8322794	-0,4	0,4418425	0,1	0,6687646	0,3	0,3698343	0,1	0,8417675
	EF2368		Hypothetical proteins		hypothetical protein	NA	NA	-0,6	0,2091301	0,4	0,2801758	0,6	0,0794976	0,1	0,7725065
	EF2369		Hypothetical proteins		hypothetical protein	0,2	0,5625477	-0,1	0,8746235	0,0	0,8830711	0,2	0,4726568	-0,1	0,813966
	EF2370		Unknown function	Enzymes of unknown specificity	oxidoreductase, Gfo/Idh/MocA family	1,3	0,0006352	0,5	0,3066533	0,3	0,3278298	0,5	0,1240763	0,2	0,479925
	EF2371	*asnS*	Protein synthesis	tRNA aminoacylation	asparaginyl-tRNA synthetase	-1,7	**0,0000071**	-0,5	0,2800271	0,0	0,8923709	-0,3	0,3769125	-0,1	0,8748218
	EF2372	*aspB*	Amino acid biosynthesis	Aspartate family	aspartate aminotransferase	-1,8	**0,0000014**	-1,0	0,0301137	-0,1	0,819796	-0,3	0,4118212	-0,1	0,6727538
	EF2373		Hypothetical proteins	Conserved	conserved hypothetical protein	NA	NA	-1,4	0,002223	0,0	0,9650726	0,1	0,7978427	0,1	0,854172
	EF2374		DNA metabolism	DNA replication, recombination, and repair	DNA polymerase III, epsilon subunit/ATP-dependent helicase DinG	NA	NA	-1,0	0,0271713	0,1	0,7931096	0,3	0,4398995	0,4	0,2768946
	EF2376		Hypothetical proteins		hypothetical protein	NA	NA	-0,5	0,3449224	NA	NA	NA	NA	0,2	0,5204374
	EF2377		Transport and binding proteins	Amino acids, peptides and amines	amino acid permease family protein	NA	NA	-0,8	0,0983674	0,1	0,6766463	0,3	0,4403845	0,2	0,5274973
	EF2378		DNA metabolism	DNA replication, recombination, and repair	DNA polymerase III, alpha subunit, Gram-positive type	-2,1	**0**	-0,6	0,2346523	-0,2	0,557007	-0,4	0,2104531	0,1	0,8664688
	EF2379	*proS*	Protein synthesis	tRNA aminoacylation	prolyl-tRNA synthetase	0,0	0,9530572	-0,3	0,5854002	-0,1	0,854424	0,3	0,3103112	0,0	0,9598459
	EF2380		Protein fate	Degradation of proteins, peptides, and glycopeptides	membrane-associated zinc metalloprotease, putative	-0,7	0,0447753	-0,3	0,4770496	0,1	0,7574895	0,0	0,9950544	0,2	0,6465815
	EF2381		Hypothetical proteins		hypothetical protein	NA	NA	NA	NA	NA	NA	NA	NA	NA	NA
	EF2382	*gdh*	Energy metabolism	Other	glucose 1-dehydrogenase	1,3	0,0004251	0,4	0,3547781	0,3	0,269995	0,7	0,0690068	0,0	0,985941
	EF2383		Hypothetical proteins	Conserved	conserved hypothetical protein	NA	NA	-0,2	0,6136639	NA	NA	-0,2	0,4823959	0,3	0,3598136
	EF2384		Hypothetical proteins		hypothetical protein	NA	NA	NA	NA	NA	NA	NA	NA	NA	NA
	EF2385		Hypothetical proteins		hypothetical protein	NA	NA	-0,1	0,8386583	0,3	0,4649573	-0,1	0,8753413	0,5	0,1255961
	EF2386		Hypothetical proteins		hypothetical protein	-0,2	0,5867693	-0,2	0,6059987	0,0	0,9920137	-0,1	0,8446045	0,6	0,0803547
	EF2387		Cellular processes	Cell division	chromosome partitioning ATPase, ParA family	NA	NA	-0,3	0,4935398	NA	NA	0,1	0,8636553	0,3	0,3159774
	EF2388		Hypothetical proteins		hypothetical protein	0,8	0,0355329	-0,4	0,3446612	-0,5	0,1015367	NA	NA	0,0	0,9138128
	EF2389		Hypothetical proteins		hypothetical protein	NA	NA	0,7	0,2253428	NA	NA	0,2	0,4784222	0,3	0,4291722
	EF2390		Hypothetical proteins	Conserved	conserved hypothetical protein	0,4	0,2435601	-0,2	0,7271734	0,4	0,1616043	0,7	0,0433132	0,0	0,91442
	EF2391		Unknown function	General	NifU family protein	-0,2	0,7035658	-0,1	0,8445381	0,5	0,1446905	0,3	0,4016673	0,2	0,5858187
	EF2392		Unknown function	Enzymes of unknown specificity	aminotransferase, class V	-0,3	0,5441164	-0,1	0,8332211	0,4	0,2150779	0,5	0,1526428	0,2	0,5598953
	EF2393		Hypothetical proteins	Conserved	conserved hypothetical protein	-0,3	0,3637749	-0,2	0,6495779	0,5	0,1452105	0,8	0,0172079	-0,3	0,2950379

Operon[a)]	Locus	Gene	Functional category	Subcategory	Putative function	Blood[b)]	P-value[c)]	YTB_5[b)]	P-value[c)]	YTB_15[b)]	P-value[c)]	YTB_30[b)]	P-value[c)]	YTB_60[b)]	P-value[c)]
	EF2394		Transport and binding proteins	Unknown substrate	ABC transporter, ATP-binding protein	0,1	0,8053841	-0,2	0,5936828	0,4	0,1560707	0,9	0,0063167	-0,1	0,7906871
	EF2395	*frr*	Protein synthesis	Translation factors	ribosome recycling factor	-2,5	**0**	-1,6	0,0007577	0,1	0,8371298	-0,6	0,0720537	0,2	0,5903153
	EF2396	*pyrH*	Purines, pyrimidines, nucleosides, and nucleotides	Nucleotide and nucleoside interconversions	uridylate kinase	-2,8	**0**	-0,9	0,0408019	-0,1	0,8279704	-0,5	0,1045369	0,1	0,7868565
	EF2397	*tsf*	Protein synthesis	Translation factors	translation elongation factor Ts	NA	NA	-0,7	0,1510675	0,2	0,6041908	-0,7	0,0277114	0,5	0,1069904
	EF2398	*rpsB*	Protein synthesis	Ribosomal proteins: synthesis and modification	ribosomal protein S2	-2,9	**0**	-0,9	0,0523554	-0,1	0,6657088	-0,3	0,4512806	0,0	0,908736
	EF2399		Unknown function	Enzymes of unknown specificity	acetyltransferase, GNAT family	-1,2	0,0008606	-0,7	0,1265879	0,1	0,816515	-0,2	0,5590982	0,2	0,5781001
	EF2400		Protein synthesis	tRNA and rRNA base modification	RNA methyltransferase, TrmH family	0,4	0,3366784	-0,2	0,6297581	0,2	0,6274906	0,3	0,3040221	0,6	0,0517121
	EF2401	*acyP*	Fatty acid and phospholipid metabolism	Other	acylphosphatase	0,9	0,0440366	0,3	0,4567299	-0,1	0,7116848	0,3	0,379618	-0,4	0,2321098
	EF2404		Hypothetical proteins		hypothetical protein	-0,7	0,0608028	-0,6	0,1923355	-0,2	0,5414178	-0,1	0,8093717	-0,3	0,4112245
	EF2405		Hypothetical proteins		hypothetical protein	-1,8	**0,0000004**	-0,4	0,3954544	-0,3	0,3827547	-0,8	0,0214116	0,1	0,7935978
	EF2406	*glyS*	Protein synthesis	tRNA aminoacylation	glycyl-tRNA synthetase, beta subunit	-1,9	**0,0000001**	-0,4	0,3663717	-0,4	0,2213081	-0,7	0,0326218	-0,1	0,6942357
	EF2407	*glyQ*	Protein synthesis	tRNA aminoacylation	glycyl-tRNA synthetase, alpha subunit	-3,1	**0**	-1,3	0,0042704	-0,5	0,1261786	-0,8	0,0117234	-0,2	0,5090673
	EF2408		Hypothetical proteins		hypothetical protein	-1,2	0,0097253	-0,9	0,0400248	-0,5	0,0989583	-0,3	0,3312596	0,5	0,1547571
	EF2409		DNA metabolism	DNA replication, recombination, and repair	DNA repair protein RecO, putative	-1,3	0,0050311	0,0	0,9172833	0,4	0,1639779	0,3	0,3110807	0,0	0,9825293
	EF2410	*era*	Regulatory functions	Small molecule interactions	GTP-binding protein Era	NA	NA	-0,2	0,6587399	0,3	0,3882137	NA	NA	0,1	0,8263013
	EF2411	*dgkA*	Fatty acid and phospholipid metabolism	Biosynthesis	diacylglycerol kinase	-1,6	0,0000183	0,0	0,9418349	0,0	0,906802	0,2	0,5079137	0,0	0,9152938
	EF2412		Hypothetical proteins	Conserved	conserved hypothetical protein TIGR00043	-0,2	0,6205267	-0,4	0,3603413	-0,1	0,8525486	0,0	0,9407222	0,1	0,8675937
	EF2413		Unknown function	General	HD domain protein	-1,5	0,0000343	-0,3	0,5684543	-0,2	0,4301076	-0,4	0,294217	-0,1	0,769905
	EF2414	*phoH*	Unknown function	General	phoH-like protein	-1,0	0,0309261	-0,6	0,1629579	0,2	0,5898178	0,2	0,5207306	0,1	0,8485823
	EF2415		Hypothetical proteins	Conserved	conserved hypothetical protein	-1,4	0,000075	-0,6	0,2245972	-0,1	0,812921	0,0	0,9588536	0,2	0,5253929
	EF2416	*rpsU*	Protein synthesis	Ribosomal proteins: synthesis and modification	ribosomal protein S21	-1,6	**0,0000143**	-0,5	0,3146997	-0,1	0,6517887	0,2	0,5492594	0,3	0,3618526
	EF2417		Regulatory functions	DNA interactions	transcriptional regulator, Fur family	-0,1	0,7394271	0,9	0,0479799	0,2	0,5747458	0,3	0,4043886	-0,1	0,8053376
	EF2419		Hypothetical proteins	Conserved	conserved hypothetical protein	0,1	0,7797166	0,5	0,2797969	0,1	0,6857308	0,2	0,5870144	0,6	0,0875806
	EF2420	*thrB*	Amino acid biosynthesis	Aspartate family	homoserine kinase	0,4	0,328621	0,0	0,933286	-0,1	0,7239308	-0,1	0,6713016	0,3	0,3772628
	EF2421	*thrC*	Amino acid biosynthesis	Aspartate family	threonine synthase	0,5	0,1916266	0,0	0,9920394	0,0	0,9645611	-0,1	0,8443704	0,1	0,6840712
	EF2422	*hom*	Amino acid biosynthesis	Aspartate family	homoserine dehydrogenase	0,2	0,5913546	0,1	0,7664923	0,0	0,9470791	-0,1	0,7343048	0,0	0,9265728
	EF2423		Regulatory functions	DNA interactions	transcriptional regulator, ArsR family	0,3	0,3552583	-0,1	0,9034727	-0,3	0,4258225	0,1	0,757194	0,2	0,5630704
	EF2424		Amino acid biosynthesis	Glutamate family	pyrroline-5-carboxylate reductase, putative	-0,1	0,8027367	0,1	0,7843378	-0,1	0,8475353	-0,1	0,7544544	0,0	0,9748937
	EF2425		Energy metabolism	Sugars	phosphoglucomutase/phosphomannomutase family protein	-0,1	0,7756563	0,2	0,6735864	0,0	0,9406069	0,0	0,8838179	-0,1	0,8121958
	EF2426		Regulatory functions	DNA interactions	transcriptional regulator, GntR family	-1,4	0,0018992	-0,5	0,3064244	0,1	0,8422377	-0,4	0,2221697	0,0	0,9677695
	EF2427		Hypothetical proteins		hypothetical protein	1,2	0,0015079	0,8	0,0908546	0,4	0,2260152	0,2	0,5023417	0,8	0,0181483
	EF2428		Regulatory functions	DNA interactions	transcriptional regulator, PadR family	-0,4	0,3697093	0,1	0,8175915	0,5	0,2333841	0,7	0,0376233	1,1	0,0007505
	EF2429	*guaC*	Purines, pyrimidines, nucleosides, and nucleotides	Nucleotide and nucleoside interconversions	GMP reductase	-1,2	0,0015017	-0,8	0,0883944	0,5	0,0903096	0,8	0,0111288	0,3	0,3721671
	EF2430		Transport and binding proteins	Nucleosides, purines and pyrimidines	xanthine/uracil permease family protein	NA	NA	-0,5	0,335908	0,0	0,9896347	0,4	0,2525917	0,3	0,3207604
	EF2431		Unknown function	Enzymes of unknown specificity	chlorohydrolase family protein	NA	NA	0,0	0,9665058	0,2	0,5881921	1,2	0,0004011	0,4	0,2188371
	EF2432		Unknown function	Enzymes of unknown specificity	metallo-beta-lactamase superfamily protein	-1,1	0,0043837	NA	NA	NA	NA	0,0	0,9050838	NA	NA
	EF2433		Energy metabolism	Glycolysis/gluconeogenesis	phosphoglycerate mutase family protein	0,0	0,9088844	0,1	0,8345743	0,2	0,5959448	0,1	0,7261289	-0,1	0,8326157
	EF2434		Regulatory functions	DNA interactions	phosphosugar-binding transcriptional regulator, RpiR family	-0,5	0,1371682	0,4	0,4071263	0,3	0,3999496	0,1	0,8477853	0,0	0,9054764
	EF2435		Signal transduction	PTS	PTS system, IIBC components	0,6	0,1708578	-0,5	0,3261912	0,7	0,083266	0,3	0,3065382	0,1	0,8460718
	EF2436		Unknown function	General	glucokinase regulator-related protein	0,2	0,5591534	-0,1	0,853364	0,4	0,1694599	0,1	0,8046219	0,0	0,9290459
	EF2437		Hypothetical proteins	Conserved	conserved hypothetical protein	-0,7	0,0974994	0,2	0,7133414	0,3	0,2735905	0,1	0,6761938	-0,2	0,6487896
	EF2438		Signal transduction	PTS	PTS system, IIA component	NA	NA	-0,8	0,0819841	0,5	0,1153223	-0,1	0,7536044	0,0	0,9814821
	EF2439		Cellular processes	Toxin production and resistance	undecaprenol kinase, putative	-3,2	**0**	-0,8	0,0769639	-0,1	0,8722357	-0,3	0,3708038	0,0	0,912662
	EF2440		Unknown function	General	celC-related protein	-1,4	0,0015427	0,5	0,3124462	0,2	0,5760042	-0,2	0,5802711	-0,2	0,5722078
	EF2441		Hypothetical proteins	Conserved	conserved hypothetical protein	-0,9	0,0341653	0,3	0,4951381	0,1	0,6976949	-0,1	0,6863832	-0,1	0,6937428
	EF2442		Transport and binding proteins	Anions	phosphate transporter family protein	-0,6	0,1188447	0,4	0,3782699	0,3	0,3043428	0,0	0,9853197	0,1	0,7275076
	EF2443	*rpsT*	Protein synthesis	Ribosomal proteins: synthesis and modification	ribosomal protein S20	-2,8	**0**	-1,0	0,0231196	-0,2	0,497793	-0,7	0,033445	0,1	0,6809026
	EF2444		Fatty acid and phospholipid metabolism	Other	acyl-CoA thioester hydrolase	-2,9	**0**	-1,1	0,0203045	0,3	0,3477133	-0,2	0,4898807	0,0	0,9543848
	EF2445		Biosynthesis of cofactors, prosthetic groups, carriers	Pantothenate and coenzyme A	2-dehydropantoate 2-reductase, putative	-0,6	0,1224817	0,2	0,6336537	0,3	0,3342705	0,1	0,7491778	-0,4	0,2260939
	EF2446		Hypothetical proteins	Conserved	conserved hypothetical protein	-1,0	0,0343013	-0,2	0,648103	0,5	0,1030996	0,6	0,0593933	0,3	0,3840759
	EF2447		Cellular processes	DNA transformation	DNA internalization-related competence protein ComEC/Rec2	-0,7	0,0850073	-0,9	0,0524821	-0,1	0,842498	-0,1	0,8546212	0,1	0,8043909
	EF2448		Cellular processes	DNA transformation	comE operon protein 2, putative	-0,1	0,8765944	-0,3	0,4716623	-0,1	0,7541123	0,2	0,5158886	0,4	0,3488027
	EF2449	*comEA*	Cellular processes	DNA transformation	competence protein comEA	NA	NA	0,5	0,3718785	0,9	0,0258244	NA	NA	-0,1	0,707869
	EF2450		Unknown function	General	PDZ domain protein	-1,8	**0,0000041**	0,0	0,9560479	-0,1	0,6610761	0,1	0,8099126	0,2	0,6285251
	EF2451	*coaD*	Biosynthesis of cofactors, prosthetic groups, carriers	Pantothenate and coenzyme A	pantetheine-phosphate adenylyltransferase	-1,7	0,0002187	-0,4	0,34979	0,1	0,7322531	0,0	0,9412467	0,1	0,7656462
	EF2452		Unknown function	Enzymes of unknown specificity	methylase, putative	-1,3	0,0005737	-0,4	0,3445631	0,1	0,8532606	0,3	0,4141618	0,2	0,5835866
	EF2453		Hypothetical proteins	Conserved	conserved hypothetical protein	-0,3	0,4143558	-0,3	0,5757444	0,1	0,7465478	0,2	0,4656714	-0,1	0,7107718
	EF2454		Hypothetical proteins	Conserved	conserved hypothetical protein	-0,7	0,0526207	-0,4	0,4350624	0,3	0,416359	0,3	0,4192851	-0,1	0,655089
	EF2455		Hypothetical proteins		hypothetical protein	-1,0	0,0119273	-0,4	0,4367221	0,0	0,8774168	0,0	0,8985255	0,1	0,8371524
	EF2456	*pycA*	Energy metabolism	Glycolysis/gluconeogenesis	pyruvate carboxylase	-0,5	0,1427382	-0,2	0,6626302	0,2	0,5793352	0,0	0,882752	0,1	0,8811584
	EF2457		Cellular processes	Cell division	cell division protein, FtsW/RodA/SpoVE family	-1,1	0,002677	-0,2	0,7393995	0,1	0,7486082	0,0	0,9670925	0,1	0,7121298
	EF2458		Hypothetical proteins	Conserved	conserved hypothetical protein	-0,9	0,0476871	-0,4	0,3471931	0,4	0,2184168	0,1	0,6808403	0,0	0,9461019
	EF2459	*recQ-2*	DNA metabolism	DNA replication, recombination, and repair	ATP-dependent DNA helicase RecQ	-1,0	0,0043297	-0,3	0,4746435	0,2	0,5260926	-0,1	0,8454018	-0,1	0,6857152
	EF2460		Unknown function	General	GTP-binding protein TypA	0,2	0,6674228	-0,7	0,1483659	-0,2	0,5815888	-0,3	0,4142112	0,0	0,9575924
	EF2461		Unknown function	General	inositol monophosphatase protein family	-0,3	0,3459116	-0,7	0,1138473	-0,1	0,776408	0,3	0,4203966	0,0	0,9167091
	EF2462		Hypothetical proteins	Conserved	conserved hypothetical protein	-0,6	0,1844428	-0,7	0,1288969	0,3	0,2777657	0,1	0,7783022	0,1	0,7092016
	EF2463		Transport and binding proteins	Anions	voltage-gated chloride channel family protein	NA	NA	NA	NA	NA	NA	NA	NA	NA	NA
	EF2464		Hypothetical proteins	Domain	conserved domain protein	NA	NA	NA	NA	NA	NA	-0,1	0,8156663	0,0	0,9356944
	EF2465		Hypothetical proteins		hypothetical protein	NA	NA	0,0	0,9420203	NA	NA	0,1	0,8326726	0,1	0,7340092
	EF2466		Hypothetical proteins	Domain	conserved domain protein	NA	NA	NA	NA	NA	NA	NA	NA	NA	NA
	EF2467		Hypothetical proteins	Conserved	conserved hypothetical protein	NA	NA	0,2	0,6723435	NA	NA	NA	NA	0,5	0,2571296
	EF2469		Regulatory functions	DNA interactions	transcriptional regulator, Cro/CI family	NA	NA	NA	NA	NA	NA	-0,2	0,5257546	0,2	0,6285543
	EF2470		Unknown function	General	HD domain protein	0,1	0,7178202	-0,4	0,3600102	0,4	0,1553621	-0,2	0,5531776	0,1	0,8639514
	EF2471	*argS*	Protein synthesis	tRNA aminoacylation	arginyl-tRNA synthetase	-1,3	0,0003941	-0,2	0,7002123	0,1	0,7087114	-0,5	0,173241	0,0	0,8917558
	EF2472	*gcp*	Protein fate	Degradation of proteins, peptides, and glycopeptides	O-sialoglycoprotein endopeptidase	-2,2	**0**	-0,9	0,045245	-0,3	0,269732	-0,6	0,055538	-0,1	0,7295959
	EF2473		Protein synthesis	Ribosomal proteins: synthesis and modification	ribosomal-protein-alanine acetyltransferase, putative	NA	NA	-0,8	0,0763858	-0,3	0,4253591	-0,5	0,1724603	-0,3	0,3127518
	EF2474		Protein synthesis	Ribosomal proteins: synthesis and modification	ribosomal-protein-alanine acetyltransferase, putative	-2,5	**0**	-1,0	0,0308792	-0,3	0,3023378	-0,2	0,5694244	-0,2	0,5368459
	EF2475		Hypothetical proteins	Conserved	conserved hypothetical protein	-1,7	**0,0000069**	-1,2	0,0116798	-0,2	0,56725	-0,1	0,7989279	-0,2	0,6356687
	EF2476		Cell envelope	Biosynthesis/degradation of murein sacculus/peptidoglycan	penicillin-binding protein 4	0,5	0,1853065	0,3	0,5512908	0,1	0,6756293	0,3	0,4372512	0,0	0,9453741
	EF2477		Hypothetical proteins	Conserved	conserved hypothetical protein	2,2	**0**	1,3	0,0053872	0,4	0,1711206	0,6	0,0774647	0,4	0,2075153
	EF2478		Hypothetical proteins	Conserved	conserved hypothetical protein	1,6	0,0000225	0,5	0,2619058	0,3	0,3728991	0,3	0,3986535	0,4	0,1937456
	EF2479		Hypothetical proteins	Conserved	conserved hypothetical protein	1,9	**0,0000009**	0,6	0,2262764	0,5	0,1259167	0,2	0,4850897	0,3	0,3540322
	EF2480		Hypothetical proteins	Conserved	conserved hypothetical protein	-0,1	0,8407527	-0,1	0,7941682	0,1	0,7359302	0,0	0,9889655	0,3	0,319463

Operon[a]	Locus	Gene	Functional category	Subcategory	Putative function	Blood[b]	P-value[c]	YTB_5[b]	P-value[c]	YTB_15[b]	P-value[c]	YTB_30[b]	P-value[c]	YTB_60[b]	P-value[c]
	EF2481		Unknown function	Enzymes of unknown specificity	hydrolase, haloacid dehalogenase-like family	NA	NA	0,5	0,2429977	0,4	0,3366191	0,4	0,2209802	0,3	0,3939746
	EF2483		Hypothetical proteins		hypothetical protein	1,4	0,0001761	0,0	0,9876882	-0,2	0,5218453	NA	NA	0,4	0,2127738
	EF2484		Hypothetical proteins	Conserved	conserved hypothetical protein	-1,7	**0,0000046**	-0,4	0,4292498	-0,9	0,0042804	-1,3	0,0014329	-0,1	0,729101
	EF2485	*cpsK*	Transport and binding proteins	Unknown substrate	ABC transporter, permease protein	-2,9	**0**	-0,1	0,9072637	-1,4	**0,0000065**	-1,6	**0,0000022**	0,0	0,9087179
	EF2486	*cpsJ*	Transport and binding proteins	Unknown substrate	ABC transporter, ATP-binding protein	-3,5	**0**	-0,1	0,8549169	-1,3	0,0000518	-1,6	**0,0000033**	-0,1	0,8722111
	EF2487	*cpsI*	Cell envelope	Biosynthesis and degradation of surface poly/liposaccharides	UDP-galactopyranose mutase	-2,4	**0**	-0,3	0,5481724	-1,1	0,0003599	-1,4	0,0000418	0,1	0,6880764
	EF2488	*cpsH*	Cell envelope	Other	lipoprotein, putative	-2,5	**0**	-0,4	0,4345619	-1,4	**0,000014**	-1,3	0,0000606	0,1	0,8694569
	EF2489	*cpsG*	Cell envelope	Biosynthesis/degradation of murein sacculus/peptidoglycan	MurB family protein	-2,9	**0**	-0,4	0,4271672	-1,1	0,0002713	-1,2	0,000473	-0,3	0,3962635
	EF2490	*cpsF*	Hypothetical proteins	Conserved	conserved hypothetical protein	-3,2	**0**	-0,7	0,1381417	-1,0	0,0019039	-0,8	0,0126826	0,0	0,9379301
	EF2491	*cpsE*	Cell envelope	Biosynthesis and degradation of surface poly/liposaccharides	glycosyl transferase, group 2 family protein	-1,9	**0,0000003**	-1,6	0,0005906	-0,9	0,003021	-1,0	0,0033239	-0,1	0,6757597
	EF2492	*cpsD*	Cell envelope	Biosynthesis and degradation of surface poly/liposaccharides	glycosyl transferase, group 2 family protein	-2,1	**0**	-0,8	0,0860458	-1,1	0,0007203	-0,6	0,0799127	0,0	0,9462414
	EF2493	*cpsC*	Cell envelope	Biosynthesis/degradation of murein sacculus/peptidoglycan	teichoic acid biosynthesis protein, putative	NA	NA	-0,4	0,3473859	0,0	0,9001955	-0,2	0,5457387	0,3	0,4197703
	EF2494	*cdsA*	Fatty acid and phospholipid metabolism	Biosynthesis	phosphatidate cytidylyltransferase	-0,8	0,0287643	-0,6	0,1926259	0,1	0,7958363	0,2	0,5081534	0,3	0,3831547
	EF2495	*uppS*	Biosynthesis of cofactors, prosthetic groups, carriers	Other	undecaprenyl diphosphate synthase	-0,6	0,1324367	-0,3	0,4857293	0,0	0,952053	0,4	0,2495363	0,0	0,931612
	EF2496		Cellular processes	Other	pheromone cOB1 precursor/lipoprotein, YaeC family	-0,7	0,045038	-0,3	0,465707	0,0	0,932205	-0,6	0,0532843	-0,1	0,8688168
	EF2497		Transport and binding proteins	Unknown substrate	ABC transporter, permease protein	-1,2	0,0010103	-0,6	0,2005209	-0,4	0,2654003	-0,9	0,0050416	-0,1	0,7560284
	EF2498		Transport and binding proteins	Unknown substrate	ABC transporter, ATP-binding protein	-1,1	0,0020074	-0,7	0,1211114	-0,3	0,3776169	-0,5	0,1033349	0,2	0,4561695
	EF2499		Hypothetical proteins		hypothetical protein	NA	NA	NA	NA	NA	NA	NA	NA	NA	NA
	EF2500		Unknown function	General	GcvH family protein	-2,5	**0**	-1,0	0,0347892	-0,2	0,518604	0,0	0,9512833	-0,1	0,8542048
	EF2501		Cellular processes	Detoxification	arsenate reductase, putative	-2,0	**0,0000001**	-0,8	0,0749171	-0,1	0,6715634	-0,1	0,8015365	-0,1	0,7036568
	EF2502		Cellular processes	Cell division	cell division protein, FtsW/RodA/SpoVE family	-0,1	0,686739	-1,4	0,0029576	-0,4	0,1910826	0,0	0,9620801	-0,1	0,6934649
	EF2503		Hypothetical proteins	Domain	conserved domain protein	NA	NA	NA	NA	NA	NA	NA	NA	NA	NA
	EF2504		Hypothetical proteins		hypothetical protein	-1,1	0,0113032	-0,7	0,1220643	-1,4	0,0003132	NA	NA	0,1	0,8400537
	EF2505		Cell envelope	Other	cell wall surface anchor family protein	-0,7	0,0954017	-0,7	0,109399	-0,2	0,5264035	-0,6	0,0868541	0,0	0,9743223
	EF2507		Hypothetical proteins	Conserved	conserved hypothetical protein	0,0	0,9195786	0,3	0,5625099	0,0	0,9656481	-0,2	0,554072	0,1	0,7266317
	EF2508		Regulatory functions	DNA interactions	transcriptional regulator, Cro/CI family	0,6	0,0762984	-0,1	0,8916707	0,0	0,9471539	0,2	0,5738248	0,2	0,5716289
	EF2509		Unknown function	General	AzlC family protein	NA	NA	0,0	0,9482919	0,7	0,0322478	0,0	0,925175	0,0	0,8840475
	EF2512		Cell envelope	Other	lipoprotein, putative	NA	NA	NA	NA	NA	NA	NA	NA	NA	NA
	EF2513		Cell envelope	Other	lipoprotein, putative	0,9	0,0138254	0,0	0,9846834	0,0	0,9010142	0,3	0,438148	-0,1	0,8210942
	EF2514		Hypothetical proteins		hypothetical protein	NA	NA	NA	NA	NA	NA	NA	NA	-0,6	0,15129
	EF2515		Hypothetical proteins	Domain	conserved domain protein	0,4	0,377962	0,2	0,6739075	-0,5	0,1107654	0,1	0,8422532	-0,5	0,1500199
	EF2516		Cell envelope	Other	membrane protein, putative	NA	NA	NA	NA	NA	NA	NA	NA	NA	NA
	EF2517		Mobile and extrachromosomal element functions	Plasmid functions	conjugal transfer protein, putative	NA	NA	NA	NA	NA	NA	NA	NA	-0,1	0,7998217
	EF2518		Hypothetical proteins	Domain	conserved domain protein	2,9	**0**	0,2	0,7173805	0,1	0,6387103	0,2	0,5804617	0,3	0,3493907
	EF2519		Hypothetical proteins	Conserved	conserved hypothetical protein	1,3	0,0004637	-0,4	0,3529698	0,0	0,9523159	0,2	0,5737567	0,1	0,8258437
	EF2520		Hypothetical proteins	Conserved	conserved hypothetical protein	1,6	0,0004067	0,1	0,8078736	0,2	0,4717131	0,0	0,9609558	0,2	0,4840929
	EF2521		Hypothetical proteins	Conserved	conserved hypothetical protein	1,0	0,020404	0,0	0,9575471	NA	NA	NA	NA	-0,8	0,0157259
	EF2522		Hypothetical proteins		hypothetical protein	NA	NA	0,4	0,5190601	NA	NA	NA	NA	0,0	0,9352006
	EF2523		Hypothetical proteins		hypothetical protein	NA	NA	-0,3	0,5512502	NA	NA	0,2	0,5814681	0,1	0,8481165
	EF2524		Cell envelope	Surface structures	sortase family protein	0,9	0,048905	NA	NA	NA	NA	NA	NA	NA	NA
	EF2525		Cell envelope	Other	cell wall surface anchor family protein	1,2	0,0085036	-0,6	0,215904	NA	NA	0,6	0,1467546	-0,9	0,0058904
	EF2526		Hypothetical proteins		hypothetical protein	NA	NA	NA	NA	NA	NA	NA	NA	NA	NA
	EF2527		Hypothetical proteins	Domain	conserved domain protein	NA	NA	NA	NA	NA	NA	-0,5	0,1680541	0,0	0,9056453
	EF2528		Regulatory functions	DNA interactions	transcriptional regulator, Cro/CI family	NA	NA	NA	NA	NA	NA	NA	NA	-0,1	0,7167905
	EF2529		Hypothetical proteins	Conserved	conserved hypothetical protein	NA	NA	NA	NA	NA	NA	NA	NA	-0,3	0,4041056
	EF2530		Hypothetical proteins		hypothetical protein	NA	NA	NA	NA	NA	NA	NA	NA	NA	NA
	EF2531		Hypothetical proteins		hypothetical protein	NA	NA	NA	NA	NA	NA	NA	NA	-0,1	0,8358603
	EF2532		Hypothetical proteins		hypothetical protein	1,0	0,0062098	-0,2	0,7167021	-0,2	0,5562138	-0,3	0,4369677	-0,1	0,7483929
	EF2533		Cellular processes	Cell division	FtsK/SpoIIIE family protein	1,4	0,0003456	0,4	0,4170418	-0,5	0,2422383	-0,4	0,1920938	-1,3	0,0000769
	EF2534		Hypothetical proteins	Conserved	conserved hypothetical protein	2,1	**0**	0,0	0,914814	-0,1	0,8502765	-0,3	0,4482864	-0,9	0,0102289
	EF2535		Unknown function	General	nucleotidyltransferase domain protein	1,5	0,0000333	-0,2	0,6211243	0,0	0,9927991	0,1	0,698102	-0,1	0,7046692
	EF2536		Hypothetical proteins	Conserved	conserved hypothetical protein	2,3	**0**	0,0	0,9871334	0,0	0,9721946	0,4	0,2099582	-0,1	0,8803026
	EF2537		Hypothetical proteins		hypothetical protein	NA	NA	NA	NA	NA	NA	NA	NA	NA	NA
	EF2538		Hypothetical proteins		hypothetical protein	2,0	**0,0000134**	NA	NA	-0,7	0,0442477	NA	NA	0,2	0,4631191
	EF2539		Hypothetical proteins		hypothetical protein	NA	NA	-0,4	0,4587536	NA	NA	NA	NA	NA	NA
	EF2540		Hypothetical proteins		hypothetical protein	1,4	0,0015648	-0,4	0,4213936	-0,1	0,7789456	-0,5	0,1514897	-0,4	0,2743395
	EF2541		Hypothetical proteins		hypothetical protein	NA	NA	NA	NA	NA	NA	NA	NA	NA	NA
	EF2542		Hypothetical proteins		hypothetical protein	NA	NA	NA	NA	NA	NA	NA	NA	NA	NA
	EF2543		Regulatory functions	Other	transcriptional regulator, putative	NA	NA	NA	NA	NA	NA	NA	NA	0,4	0,19191
	EF2544		Regulatory functions	DNA interactions	transcriptional regulator, Cro/CI family	0,4	0,2653182	-0,4	0,4917944	0,1	0,7960396	0,2	0,6133168	0,3	0,3227917
	EF2545		Hypothetical proteins	Conserved	conserved hypothetical protein	NA	NA	0,3	0,4863312	-0,1	0,6799923	-0,3	0,4331998	-0,2	0,537248
	EF2546		DNA metabolism	DNA replication, recombination, and repair	site-specific recombinase, phage integrase family	NA	NA	0,4	0,3987707	NA	NA	NA	NA	NA	NA
	EF2547		Hypothetical proteins		hypothetical protein	1,6	0,0000395	0,5	0,3356938	1,2	0,0014558	1,4	0,0006585	0,8	0,0111449
	EF2548		Hypothetical proteins	Conserved	conserved hypothetical protein	1,2	0,005813	-0,6	0,2151479	0,3	0,2808568	0,1	0,8922544	0,3	0,4472254
	EF2549	*upp*	Purines, pyrimidines, nucleosides, and nucleotides	Salvage of nucleosides and nucleotides	uracil phosphoribosyltransferase	0,1	0,6850638	0,1	0,7913351	0,0	0,9133814	0,6	0,059579	0,1	0,6834738
	EF2550	*glyA*	Amino acid biosynthesis	Serine family	serine hydroxymethyltransferase	-0,2	0,6131499	-0,6	0,2265815	0,5	0,1263218	0,3	0,3463545	-0,2	0,5753859
	EF2552		Unknown function	General	Sua5/YciO/YrdC/YwlC family protein	-2,9	**0**	-1,1	0,0157335	-0,2	0,5152934	-0,3	0,3014611	0,1	0,7556997
	EF2553	*hemK*	Biosynthesis of cofactors, prosthetic groups, carriers	Heme, porphyrin, and cobalamin	hemK protein	-3,2	**0**	-1,4	0,002331	0,1	0,7503062	-0,1	0,8099502	0,1	0,8577214
	EF2554	*prfA*	Protein synthesis	Translation factors	peptide chain release factor 1	-1,3	0,0007619	-1,5	0,0015128	0,3	0,4112453	0,2	0,5142339	0,1	0,8553396
	EF2555	*tdK*	Purines, pyrimidines, nucleosides, and nucleotides	Nucleotide and nucleoside interconversions	thymidine kinase	-0,7	0,0640554	-1,4	0,0027852	-0,1	0,7468719	0,5	0,1297902	0,0	0,9811011
	EF2556		Energy metabolism	Anaerobic	fumarate reductase flavoprotein subunit precursor, putative	-3,5	**0**	-1,3	0,0062701	-2,1	**0**	-1,3	0,0000659	0,1	0,7295493
	EF2558		Transport and binding proteins	Cations and iron carrying compounds	cation transporter	NA	NA	-0,2	0,6941563	0,3	0,4013274	-0,1	0,8090513	-0,1	0,8463136
	EF2559		Energy metabolism	Electron transport	pyruvate flavodoxin/ferredoxin oxidoreductase family protein	NA	NA	0,2	0,7051892	-0,4	0,1714735	-2,0	**0**	-0,9	0,0053609
	EF2560	*gltA*	Amino acid biosynthesis	Glutamate family	glutamate synthase (NADPH), homotetrameric	-0,3	0,5711518	0,1	0,8626629	-0,3	0,2862569	-1,8	**0,0000001**	-0,5	0,1634514
	EF2561		Purines, pyrimidines, nucleosides, and nucleotides	Pyrimidine ribonucleotide biosynthesis	dihydroorotate dehydrogenase electron transfer subunit, putative	0,3	0,4866157	-0,3	0,5870636	-0,3	0,323715	-1,1	0,001216	-0,5	0,108332
	EF2562		Energy metabolism	Electron transport	flavodoxin	NA	NA	0,0	0,946676	-0,7	0,0207722	-1,5	**0,0000076**	-0,5	0,1365773
	EF2563		Hypothetical proteins	Conserved	conserved hypothetical protein	NA	NA	0,2	0,6856053	-0,9	0,0201835	-1,6	**0,0000009**	-0,6	0,0774592
	EF2564		Hypothetical proteins	Domain	conserved domain protein	NA	NA	NA	NA	NA	NA	-1,0	0,0022924	-0,8	0,0636223
	EF2565		Hypothetical proteins	Conserved	conserved hypothetical protein	NA	NA	NA	NA	NA	NA	-1,1	0,0010588	-0,2	0,639025
	EF2566		Hypothetical proteins	Conserved	conserved hypothetical protein	NA	NA	0,2	0,629308	NA	NA	-0,8	0,015773	0,0	0,9855555
	EF2567	*selD*	Protein synthesis	tRNA aminoacylation	selenide, water dikinase	0,3	0,4024323	0,0	0,9695058	-0,5	0,1380191	-1,2	0,0003572	-0,4	0,1889254
	EF2568		Unknown function	Enzymes of unknown specificity	aminotransferase, class V	-0,7	0,0589931	0,0	0,9937792	-0,7	0,0335768	-1,5	**0,0000063**	-0,4	0,2673003
	EF2569		Hypothetical proteins	Conserved	conserved hypothetical protein	NA	NA	-0,1	0,8481355	-0,8	0,0154554	-1,5	**0,0000044**	-0,6	0,0539552

Operon[a]	Locus	Gene	Functional category	Subcategory	Putative function	Blood[b]	P-value[c]	YTB_5[b]	P-value[c]	YTB_15[b]	P-value[c]	YTB_30[b]	P-value[c]	YTB_60[b]	P-value[c]
	EF2570		Unknown function	Enzymes of unknown specificity	aldehyde oxidoreductase, putative	-0,2	0,56979	-0,1	0,9039154	-0,8	0,008155	-1,1	0,0011355	-0,4	0,1755471
	EF2571		Hypothetical proteins	Domain	conserved domain protein	-0,5	0,2223341	-0,3	0,5660764	-0,3	0,2900252	-0,7	0,0354393	-0,3	0,3792723
	EF2572		Unknown function	General	molybdenum transport domain protein	-1,0	0,0262214	-0,2	0,7382715	-0,2	0,5180591	-0,8	0,0178159	-0,4	0,2882626
	EF2573		Transport and binding proteins	Nucleosides, purines and pyrimidines	xanthine/uracil permease family protein	-0,6	0,1502981	-0,2	0,5992724	0,1	0,8390659	-1,6	0,0001114	-0,4	0,2433762
	EF2574		Transcription	Degradation of RNA	endoribonuclease L-PSP, putative	0,4	0,3601044	0,1	0,8570061	-0,4	0,2715564	-1,3	0,0001613	-0,6	0,0556113
	EF2575	*arcC-4*	Energy metabolism	Amino acids and amines	carbamate kinase	NA	NA	0,2	0,5977266	-0,2	0,5789832	-1,2	0,0003899	-0,1	0,6524663
	EF2576		Hypothetical proteins		hypothetical protein	NA	NA	NA	NA	NA	NA	NA	NA	-0,3	0,4419944
	EF2577		Unknown function	Enzymes of unknown specificity	aspartate/ornithine carbamoyltransferase family protein	NA	NA	NA	NA	NA	NA	-0,7	0,0408842	-0,3	0,3430352
	EF2578		Protein fate	Degradation of proteins, peptides, and glycopeptides	peptidase, M20/M25/M40 family	NA	NA	NA	NA	NA	NA	-0,7	0,1028857	0,1	0,8152068
	EF2579		Energy metabolism	Other	diaminopropionate ammonia-lyase, putative	-1,7	0,0001828	-0,7	0,152465	-0,2	0,51831	NA	NA	-0,1	0,8573883
	EF2580		Purines, pyrimidines, nucleosides, and nucleotides	Salvage of nucleosides and nucleotides	D-hydantoinase	0,8	0,0339558	0,0	0,99122	-0,3	0,4011116	-0,4	0,2510361	-0,2	0,5665721
	EF2581		Unknown function	Enzymes of unknown specificity	oxidoreductase, pyridine nucleotide-disulfide family	0,2	0,6995335	1,2	0,0411489	NA	NA	0,0	0,8850824	-0,6	0,0843753
	EF2582		Unknown function	Enzymes of unknown specificity	chlorohydrolase family protein	NA	NA	0,6	0,2227414	NA	NA	-0,2	0,6377057	-0,5	0,0981697
	EF2583		Hypothetical proteins	Conserved	conserved hypothetical protein	-0,2	0,7217125	-0,3	0,5751205	0,0	0,9459504	-0,5	0,1415836	-0,1	0,7939733
	EF2584		Hypothetical proteins		hypothetical protein	NA	NA	NA	NA	NA	NA	NA	NA	NA	NA
	EF2585		Cell envelope	Biosynthesis/degradation of murein sacculus/peptidoglycan	mur ligase family protein	-1,3	0,0005838	-0,9	0,0670044	-0,1	0,686777	-0,2	0,5718713	-0,5	0,1062759
	EF2586		Biosynthesis of cofactors, prosthetic groups, carriers	Heme, porphyrin, and cobalamin	cobyric acid synthase, putative	-1,6	**0,0000082**	-0,5	0,2812632	-0,3	0,3925723	-0,4	0,2241597	-0,4	0,2588699
	EF2587		Purines, pyrimidines, nucleosides, and nucleotides	Salvage of nucleosides and nucleotides	inosine-uridine preferring nucleoside hydrolase	-2,8	**0**	-0,7	0,156614	0,1	0,7626924	-0,6	0,0779496	0,2	0,4574743
	EF2588		Hypothetical proteins	Conserved	conserved hypothetical protein	-2,5	**0**	-0,7	0,1267017	0,1	0,712599	-0,8	0,0155339	0,3	0,433131
	EF2589	*manA*	Energy metabolism	Sugars	mannose-6-phosphate isomerase, class I	-0,7	0,1374418	-0,2	0,5918287	-0,2	0,5673588	-0,2	0,6472422	0,1	0,7936753
	EF2590		Hypothetical proteins	Conserved	conserved hypothetical protein	NA	NA	-0,9	0,0629397	-0,6	0,0410142	-0,6	0,1085239	0,2	0,468222
	EF2591		Unknown function	Enzymes of unknown specificity	glyoxalase family protein	-1,5	0,0000518	-1,5	0,0011741	-0,7	0,0206711	0,1	0,8433852	-0,3	0,4034563
	EF2592		Transport and binding proteins	Unknown substrate	ABC transporter, ATP-binding/permease protein	-1,0	0,0042461	-0,9	0,0642564	-0,7	0,033359	-1,1	0,0007912	-0,2	0,5395879
	EF2593		Transport and binding proteins	Unknown substrate	ABC transporter, ATP-binding/permease protein	-1,6	0,0000166	-0,9	0,0576545	-0,5	0,0897933	-0,5	0,1503737	-0,2	0,5221129
	EF2594		Regulatory functions	DNA interactions	transcriptional regulator, TetR family	-1,7	0,0000986	-0,9	0,0596097	-0,7	0,0306363	-0,3	0,357556	-0,3	0,4343347
	EF2595	*gmk-1*	Purines, pyrimidines, nucleosides, and nucleotides	Nucleotide and nucleoside interconversions	guanylate kinase	0,1	0,705943	0,5	0,2821747	0,3	0,2788648	0,3	0,4296098	0,2	0,6112465
	EF2597		Energy metabolism	Biosynthesis and degradation of polysaccharides	glycosyl hydrolase, family 1	-1,2	0,0056941	-0,4	0,3430312	0,1	0,8549361	0,0	0,9165953	0,2	0,6378199
	EF2598		Signal transduction	PTS	PTS system, beta-glucoside-specific IIABC component	0,7	0,0628335	-0,5	0,3060231	-0,5	0,0892136	NA	NA	0,1	0,65367
	EF2599		Regulatory functions	RNA interactions	transcriptional antiterminator, bglG family	NA	NA	-0,1	0,8223431	NA	NA	-0,3	0,3884727	-0,1	0,8807476
	EF2601		Fatty acid and phospholipid metabolism	Biosynthesis	acyl carrier protein, putative	-1,1	0,0132402	-1,1	0,0212539	0,0	0,8755552	-0,4	0,2777103	-0,2	0,6077866
	EF2602		Hypothetical proteins	Conserved	conserved hypothetical protein	NA	NA	-0,1	0,7515405	0,6	0,0969548	0,1	0,6884241	0,1	0,8211151
	EF2603		Signal transduction	PTS	PTS system, IIA component	1,1	0,0030165	0,5	0,2593057	0,3	0,268695	0,3	0,353842	0,1	0,7636307
	EF2604		Hypothetical proteins	Conserved	conserved hypothetical protein	0,7	0,0425582	-0,9	0,0521345	-0,2	0,4628604	-0,1	0,7855686	0,0	0,9546669
	EF2605	*murAA*	Cell envelope	Biosynthesis/degradation of murein sacculus/peptidoglycan	UDP-N-acetylglucosamine 1-carboxyvinyltransferase 1	-1,7	**0,0000021**	-1,8	0,0001445	-0,4	0,2633798	0,2	0,60185	-0,3	0,3215477
	EF2606		Hypothetical proteins	Conserved	conserved hypothetical protein	-1,3	0,0005583	-1,2	0,0110005	-0,4	0,1622406	-0,1	0,6961848	-0,1	0,671994
	EF2607	*atpC*	Energy metabolism	ATP-proton motive force interconversion	ATP synthase F1, epsilon subunit	-1,3	0,0002392	-0,6	0,1859297	-0,4	0,1721539	-0,4	0,2001077	-0,1	0,7905532
	EF2608	*atpD*	Energy metabolism	ATP-proton motive force interconversion	ATP synthase F1, beta subunit	-1,5	0,0000569	-0,6	0,180983	-0,6	0,0603499	-0,3	0,3146849	-0,3	0,4213608
	EF2609	*atpG*	Energy metabolism	ATP-proton motive force interconversion	ATP synthase F1, gamma subunit	-1,6	**0,0000088**	-0,6	0,1723739	-0,7	0,0321177	-0,5	0,1282704	-0,3	0,4198791
	EF2610	*atpA*	Energy metabolism	ATP-proton motive force interconversion	ATP synthase F1, alpha subunit	-1,4	0,0001731	-0,5	0,2449266	-0,9	0,0045964	-0,7	0,0257946	-0,3	0,3392256
	EF2611	*atpH*	Energy metabolism	ATP-proton motive force interconversion	ATP synthase F1, delta subunit	-1,2	0,0006236	-0,8	0,0986325	-0,6	0,0645186	-0,5	0,1083078	-0,4	0,2221584
	EF2612	*atpF*	Energy metabolism	ATP-proton motive force interconversion	ATP synthase F0, B subunit	-1,3	0,0005541	-0,8	0,0911069	-0,8	0,0124222	-0,5	0,1771468	-0,4	0,2544965
	EF2613	*atpE*	Energy metabolism	ATP-proton motive force interconversion	ATP synthase F0, C subunit	-2,0	**0**	-0,9	0,0447748	-0,7	0,0202048	-0,5	0,1621228	-0,4	0,2734684
	EF2614	*atpB*	Energy metabolism	ATP-proton motive force interconversion	ATP synthase F0, A subunit	-1,5	0,0000648	-0,6	0,1670471	-0,9	0,0054333	-0,3	0,3269231	-0,6	0,053492
	EF2615		Hypothetical proteins		hypothetical protein	-1,1	0,0020661	-0,7	0,1110091	-0,9	0,0064941	-0,4	0,2252852	-0,2	0,5375854
	EF2616	*smpB*	Protein synthesis	Other	SsrA-binding protein	0,8	0,0395746	0,3	0,5861453	0,0	0,8992568	0,4	0,2580331	0,3	0,4320684
	EF2617	*vacB*	Transcription	Degradation of RNA	ribonuclease R	0,7	0,0641474	0,5	0,3097773	0,1	0,7584915	0,4	0,1911812	0,2	0,608906
	EF2618		Fatty acid and phospholipid metabolism	Degradation	carboxylesterase precursor, putative	0,3	0,3872542	0,2	0,7058304	0,3	0,3395557	0,7	0,0478585	0,5	0,1707926
	EF2619		Hypothetical proteins		hypothetical protein	NA	NA	NA	NA	NA	NA	0,6	0,0751722	0,4	0,326641
	EF2620	*secG*	Protein fate	Protein and peptide secretion and trafficking	preprotein translocase, SecG subunit	-0,4	0,2727367	-1,1	0,0207305	0,5	0,1155925	1,0	0,0025755	0,7	0,0490812
	EF2621		Hypothetical proteins	Conserved	conserved hypothetical protein	-1,5	0,0000292	-0,4	0,4167761	-2,1	**0**	-1,5	**0,0000047**	0,2	0,4653832
	EF2622		Hypothetical proteins	Conserved	conserved hypothetical protein	-1,6	**0,0000131**	-0,4	0,3798177	-1,9	**0**	-1,5	**0,0000132**	0,4	0,2724926
	EF2623	*cadA*	Transport and binding proteins	Cations and iron carrying compounds	cadmium-translocating P-type ATPase	-3,1	**0**	-0,1	0,8457787	-3,9	**0**	-4,2	**0**	0,3	0,3919142
	EF2624		Hypothetical proteins		hypothetical protein	NA	NA	NA	NA	NA	NA	NA	NA	0,3	0,3426903
	EF2625	*nadE*	Biosynthesis of cofactors, prosthetic groups, carriers	Pyridine nucleotides	NH(3)-dependent NAD+ synthetase	-0,9	0,0171584	-0,4	0,4069739	-0,1	0,8431321	0,1	0,7777188	-0,1	0,7577823
	EF2626		Biosynthesis of cofactors, prosthetic groups, carriers	Pyridine nucleotides	nicotinate phosphoribosyltransferase, putative	-0,8	0,0298816	-0,7	0,1179147	-0,1	0,7219423	0,3	0,3714605	-0,2	0,5688785
	EF2627		Cell envelope	Biosynthesis and degradation of surface poly/liposaccharides	teichoic acid glycosylation protein, putative	-0,3	0,5257821	-1,6	0,0004732	0,0	0,9845291	0,9	0,0083704	0,2	0,6182719
	EF2628		Cell envelope	Biosynthesis/degradation of murein sacculus/peptidoglycan	N-acetylmuramoyl-L-alanine amidase, family 4	0,0	0,94288	-0,4	0,3946085	0,3	0,2768562	0,6	0,0555107	0,0	0,9592306
	EF2629		Hypothetical proteins		hypothetical protein	NA	NA	0,1	0,8681568	0,6	0,0732749	0,2	0,5443205	0,1	0,794885
	EF2630		Regulatory functions	Other	transcriptional regulator	NA	NA	0,0	0,9325173	0,5	0,2337036	0,0	0,8887653	0,1	0,6794719
	EF2631		Hypothetical proteins		hypothetical protein	NA	NA	NA	NA	NA	NA	NA	NA	0,4	0,2586725
	EF2632		Mobile and extrachromosomal element functions	Transposon functions	IS256, transposase	-1,5	0,000053	-1,0	0,0323189	-0,2	0,5492883	0,1	0,7836309	-0,1	0,8732894
	EF2633	*groEL*	Protein fate	Protein folding and stabilization	chaperonin, 60 kDa	-1,6	**0,0000088**	-0,6	0,202556	-0,9	0,0042377	-0,7	0,0356041	-1,0	0,0026218
	EF2634	*groES*	Protein fate	Protein folding and stabilization	chaperonin, 10 kDa	-0,6	0,0794163	-0,5	0,2950891	-0,8	0,0138158	-0,8	0,0120365	-0,8	0,0176718
	EF2636		Hypothetical proteins		hypothetical protein	NA	NA	NA	NA	NA	NA	NA	NA	NA	NA
	EF2637		Unknown function	General	abortive infection protein	0,8	0,0302394	0,3	0,5655663	0,3	0,299836	0,3	0,3635678	0,4	0,1749924
	EF2638		Unknown function	General	DNA-binding protein, putative	0,8	0,0229756	0,2	0,629569	0,6	0,0402299	0,8	0,0233898	0,1	0,6675551
	EF2639		Transport and binding proteins	Unknown substrate	ABC transporter, ATP-binding protein	-1,5	0,001173	-1,1	0,0220086	-0,5	0,1056025	-0,3	0,3893065	-0,1	0,6987742
	EF2640		Regulatory functions	DNA interactions	transcriptional regulator, GntR family	0,1	0,8321058	-0,7	0,1932374	-0,3	0,3461582	0,1	0,8233921	0,3	0,4026024
	EF2641		Transport and binding proteins	Amino acids, peptides and amines	glycine betaine/L-proline ABC transporter, ATP-binding subunit	-1,6	0,0003835	-1,5	0,001434	-0,6	0,0555973	-0,2	0,6074027	0,7	0,0274655
	EF2642		Transport and binding proteins	Amino acids, peptides and amines	glycine betaine/L-proline ABC transporter/-binding/permease	-3,1	**0**	-1,5	0,0010837	-0,5	0,1071598	-1,0	0,0021166	1,1	0,0012825
	EF2643		Hypothetical proteins	Conserved	conserved hypothetical protein	NA	NA	0,2	0,6876136	0,7	0,0639116	0,6	0,0921513	0,1	0,8674256
	EF2644		Unknown function	General	diacylglycerol kinase catalytic domain protein	3,0	**0**	0,7	0,1174394	0,4	0,1620151	0,3	0,3822747	0,0	0,9824477
	EF2645		Hypothetical proteins	Domain	conserved domain protein	0,3	0,4937043	0,1	0,8955803	0,1	0,6731468	-0,8	0,0155033	0,1	0,6909331
	EF2646		Energy metabolism	Glycolysis/gluconeogenesis	glycerate kinase, putative	2,3	**0**	1,0	0,0231475	0,3	0,3206204	-0,8	0,0186759	-0,1	0,6720341
	EF2647		Transport and binding proteins	Carbohydrates, organic alcohols, and acids	permease, GntP family	2,1	**0**	1,0	0,0377533	0,3	0,3777963	-1,7	**0,0000002**	0,1	0,7057443
	EF2648		Hypothetical proteins	Conserved	conserved hypothetical protein	-0,5	0,2610659	-0,2	0,6855977	0,5	0,1025593	0,0	0,9052125	0,3	0,4490807
	EF2649		Transport and binding proteins	Amino acids, peptides and amines	spermidine/putrescine ABC transporter/-binding protein	-2,0	**0,0000114**	-0,4	0,3458384	0,1	0,6736263	-0,6	0,056658	-0,2	0,6166605
	EF2650		Transport and binding proteins	Amino acids, peptides and amines	spermidine/putrescine ABC transporter, permease protein	-1,1	0,0035597	-0,5	0,3243662	-0,1	0,816069	-0,8	0,0137011	-0,2	0,5165683
	EF2651		Transport and binding proteins	Amino acids, peptides and amines	spermidine/putrescine ABC transporter, permease protein	-0,5	0,182367	-0,3	0,5810976	-0,3	0,4169057	-0,9	0,0055755	-0,4	0,2242553
	EF2652		Transport and binding proteins	Amino acids, peptides and amines	spermidine/putrescine ABC transporter, ATP-binding subunit	-0,3	0,4721623	-0,3	0,5873823	-0,3	0,3045606	-0,5	0,1136429	-0,3	0,3116906
	EF2653		Regulatory functions	DNA interactions	transcriptional regulator, Cro/CI family	-1,0	0,0195594	-0,2	0,6591192	0,0	0,9558051	0,1	0,8327087	-0,1	0,819768
	EF2654		Energy metabolism	Fermentation	alcohol dehydrogenase, zinc-containing	-0,2	0,6201438	0,1	0,8734782	-0,2	0,5886783	0,0	0,9638464	-0,2	0,6408566
	EF2655		Unknown function	General	flavoprotein-related protein	-1,2	0,0016206	0,0	0,9240224	0,1	0,6774769	0,1	0,7441378	0,1	0,848596

Operon[a]	Locus	Gene	Functional category	Subcategory	Putative function	Blood[b]	P-value[c]	YTB_5[b]	P-value[c]	YTB_15[b]	P-value[c]	YTB_30[b]	P-value[c]	YTB_60[b]	P-value[c]
	EF2656		Unknown function	Enzymes of unknown specificity	flavoprotein family protein	-1,4	0,0002153	0,0	0,9953647	0,0	0,8890809	0,0	0,9703431	0,0	0,9141435
	EF2657		Cell envelope	Other	membrane protein, putative	-0,6	0,1002555	0,1	0,7764977	0,2	0,612097	0,0	0,9026243	-0,1	0,8491779
	EF2658		Cellular processes	Toxin production and resistance	FemAB family protein	-1,7	**0,0000055**	-1,6	0,0007565	-0,5	0,1372478	0,0	0,9606064	-0,1	0,7130008
	EF2659		Hypothetical proteins	Conserved	conserved hypothetical protein	-4,0	**0**	-1,0	0,0235394	-0,2	0,5773645	-0,6	0,083176	0,0	0,936134
	EF2661		Unknown function	General	diacylglycerol kinase catalytic domain protein	NA	NA	-1,2	0,0101964	0,3	0,2728602	0,1	0,7711129	0,1	0,7810692
	EF2662		Cell envelope	Other	choline binding protein	-1,2	0,0062962	-0,1	0,7984927	-0,1	0,836296	-0,6	0,084978	0,0	0,9390609
	EF2663		DNA metabolism	DNA replication, recombination, and repair	helicase, putative, RecD/TraA family	NA	NA	NA	NA	NA	NA	NA	NA	NA	NA
	EF2664		Energy metabolism	Glycolysis/gluconeogenesis	phosphoglycerate mutase family protein	-1,1	0,0028951	-0,8	0,0918085	-0,3	0,374634	0,3	0,3568515	0,3	0,3255863
	EF2665		Protein synthesis	tRNA and rRNA base modification	RNA methyltransferase, TrmH family	NA	NA	-0,2	0,7102358	-0,1	0,714543	-0,1	0,6821741	-0,2	0,4518767
	EF2666		Protein synthesis	tRNA and rRNA base modification	ribosomal RNA large subunit methyltransferase A, putative	-1,4	0,0001118	-0,8	0,0872381	-0,2	0,5085766	0,3	0,4053273	0,0	0,93341
	EF2667		Transport and binding proteins	Cations and iron carrying compounds	copper homeostasis protein, putative	-1,1	0,0034454	-0,5	0,2383763	-0,2	0,4590839	0,1	0,7087343	0,0	0,9175796
	EF2668	*mgtE*	Transport and binding proteins	Cations and iron carrying compounds	magnesium transporter	-1,3	0,0002208	-0,5	0,3189117	-0,1	0,8027031	0,3	0,3465692	-0,1	0,6547642
	EF2669		Protein synthesis	tRNA and rRNA base modification	ribosomal large subunit pseudouridine synthase, RluD subfamily	-1,0	0,0052344	-0,5	0,3279105	-0,2	0,5271457	0,1	0,7307695	-0,3	0,3667348
	EF2670		Biosynthesis of cofactors, prosthetic groups, carriers	Pyridine nucleotides	inorganic polyphosphate/ATP-NAD kinase, putative	-0,6	0,1003161	-0,6	0,2210225	-0,3	0,3769095	0,2	0,4729158	-0,4	0,2344665
	EF2671		Hypothetical proteins	Conserved	conserved hypothetical protein	-0,2	0,6296352	-0,3	0,4709396	-0,1	0,6580434	0,1	0,7939229	0,1	0,8178129
	EF2672		Hypothetical proteins	Conserved	conserved hypothetical protein	1,5	0,0000246	0,5	0,2811914	0,7	0,0255549	0,9	0,008243	0,1	0,7442083
	EF2673		Hypothetical proteins	Domain	conserved domain protein	1,3	0,0002624	0,6	0,1750862	0,9	0,0068165	0,9	0,0065101	0,2	0,461125
	EF2674	*pepF*	Protein fate	Degradation of proteins, peptides, and glycopeptides	oligoendopeptidase F,plasmid	0,0	0,9501362	-0,4	0,4424257	-0,2	0,5237681	-0,7	0,0496686	0,0	0,9975852
	EF2675		Cellular processes	DNA transformation	competence protein, putative	NA	NA	0,7	0,2403065	NA	NA	NA	NA	NA	NA
	EF2677		Regulatory functions	Other	negative regulator of genetic competence MecA, putative	1,8	**0,000001**	1,0	0,0309603	-0,3	0,338043	0,1	0,6788319	-0,1	0,8197329
	EF2678		Hypothetical proteins	Conserved	conserved hypothetical protein	1,8	**0,0000004**	1,5	0,0012916	0,4	0,1642533	0,8	0,0209685	0,4	0,265069
	EF2679	*trpS*	Protein synthesis	tRNA aminoacylation	tryptophanyl-tRNA synthetase	-0,8	0,0731863	-0,2	0,7462165	0,2	0,5588906	-0,2	0,6658822	0,1	0,8767987
	EF2680		Transport and binding proteins	Unknown substrate	ABC transporter, ATP-binding/permease protein	-0,4	0,2297548	0,1	0,8418318	0,2	0,5813836	0,5	0,1175646	-0,3	0,3213318
	EF2681		Unknown function	Enzymes of unknown specificity	hydrolase, haloacid dehalogenase-like family	-1,0	0,0209936	0,1	0,9275494	-1,1	0,0003807	0,2	0,6175287	-0,7	0,0381722
	EF2682		Hypothetical proteins	Conserved	conserved hypothetical protein	NA	NA	0,6	0,3104551	-0,8	0,013683	NA	NA	-0,4	0,2788277
	EF2683		Hypothetical proteins	Conserved	conserved hypothetical protein	NA	NA	-0,2	0,6811522	NA	NA	-0,3	0,5235985	0,2	0,4761253
	EF2684		Hypothetical proteins	Conserved	conserved hypothetical protein	NA	NA	0,0	0,9374075	-0,5	0,1532302	NA	NA	-0,1	0,8024452
	EF2687		Hypothetical proteins	Domain	conserved domain protein	-0,4	0,3182758	0,1	0,9074978	0,0	0,916431	-0,4	0,247165	0,1	0,8426955
	EF2688		Unknown function	General	Snf2 family protein	-0,4	0,3036718	0,1	0,8938898	0,0	0,9745707	-0,3	0,3742093	0,0	0,9274794
	EF2689	*sbcC*	DNA metabolism	DNA replication, recombination, and repair	exonuclease SbcC	-0,9	0,0531545	-0,3	0,5428221	0,0	0,9609072	0,3	0,4139125	0,0	0,9755281
	EF2690	*sbcD*	DNA metabolism	DNA replication, recombination, and repair	exonuclease SbcD	-0,5	0,2005227	0,6	0,1698261	0,2	0,5449909	0,1	0,6917166	0,4	0,1866347
	EF2691		Fatty acid and phospholipid metabolism	Biosynthesis	1-acyl-sn-glycerol-3-phosphate acyltransferase, putative	-0,5	0,1494294	0,0	0,9584748	0,0	0,899949	0,6	0,09596	0,2	0,6417454
	EF2692		Hypothetical proteins	Conserved	conserved hypothetical protein	0,3	0,4955394	-0,4	0,344487	0,6	0,0578168	0,7	0,0386977	0,5	0,1413057
	EF2693		Hypothetical proteins	Conserved	conserved hypothetical protein	0,2	0,7223152	-0,2	0,7237322	0,0	0,9069097	0,4	0,2380286	0,2	0,5172603
	EF2694	*pfs*	Purines, pyrimidines, nucleosides, and nucleotides	Salvage of nucleosides and nucleotides	MTA/SAH nucleosidase	0,6	0,0893482	0,0	0,9301583	-0,2	0,5702972	0,2	0,6007975	0,1	0,7917706
	EF2695		Hypothetical proteins		hypothetical protein	0,1	0,7902706	-0,1	0,8041639	-0,1	0,7577008	0,1	0,7583561	0,0	0,9821292
	EF2696		DNA metabolism	DNA replication, recombination, and repair	MutT/nudix family protein	0,3	0,363373	-0,1	0,8379943	0,1	0,6667622	0,3	0,3274553	0,4	0,2528355
	EF2697		Hypothetical proteins	Domain	conserved domain protein	1,6	**0,0000106**	1,9	0,000034	0,2	0,6334895	0,6	0,0681179	1,0	0,0020171
	EF2698		Cellular processes	Toxin production and resistance	tellurite resistance protein, putative	1,1	0,0036131	1,6	0,000474	0,3	0,3857284	0,8	0,016962	0,5	0,1518717
	EF2699		Hypothetical proteins		hypothetical protein	NA	NA	NA	NA	NA	NA	NA	NA	NA	NA
	EF2700		DNA metabolism	DNA replication, recombination, and repair	MutT/nudix family protein	0,7	0,0593283	0,3	0,4587404	0,0	0,8948297	0,0	0,91541	0,0	0,9352708
	EF2701		Unknown function	Enzymes of unknown specificity	acetyltransferase, GNAT family	0,4	0,2534068	0,1	0,7789044	0,0	0,8888021	-0,1	0,8597218	0,0	0,9715729
	EF2702		Hypothetical proteins		hypothetical protein	1,8	**0,0000008**	0,5	0,3249552	0,8	0,0092898	1,4	0,0000343	0,2	0,5730795
	EF2703		Regulatory functions	Other	transcriptional regulator	0,2	0,6298509	0,3	0,5162443	1,1	0,0006416	1,2	0,0002061	0,4	0,1810546
	EF2704	*mutY*	DNA metabolism	DNA replication, recombination, and repair	A/G-specific adenine glycosylase	-0,2	0,5033886	-0,1	0,8690733	0,2	0,435886	0,1	0,8195277	0,1	0,6559502
	EF2705		Regulatory functions	Other	regulatory protein RecX, putative	0,2	0,5040085	-0,4	0,449021	0,2	0,4901177	0,2	0,4869329	0,1	0,6646092
	EF2706		Protein synthesis	tRNA and rRNA base modification	RNA methyltransferase, TrmA family	-0,2	0,6714716	-0,5	0,3211283	-0,2	0,6145279	0,2	0,6425197	0,1	0,6817304
	EF2707		Hypothetical proteins		hypothetical protein	0,7	0,051625	0,2	0,6054983	0,2	0,4294909	0,0	0,8937742	-0,1	0,6718944
	EF2708		Cell envelope	Other	membran protein, putative	2,0	**0**	0,4	0,3466515	1,1	0,0005662	0,7	0,0305366	0,1	0,7041001
	EF2709		Energy metabolism	Biosynthesis and degradation of polysaccharides	glycosyl hydrolase, family 2	NA	NA	-0,4	0,4467746	NA	NA	0,5	0,1520844	-0,3	0,4425804
	EF2710		Transport and binding proteins	Amino acids, peptides and amines	amino acid permease family protein	NA	NA	1,1	0,0577205	NA	NA	NA	NA	0,0	0,9959803
	EF2711		Regulatory functions	DNA interactions	transcriptional regulator, AraC family	0,5	0,1986934	1,1	0,0175541	0,4	0,1995525	0,5	0,1552015	0,1	0,8306367
	EF2712		Hypothetical proteins		hypothetical protein	1,3	0,0002611	2,0	0,0000178	0,1	0,8489675	1,3	0,0001327	0,8	0,0135526
	EF2713		Cell envelope	Other	cell wall surface anchor family protein	2,8	**0**	2,7	**0**	-0,5	0,1509943	0,7	0,0272944	1,3	0,0001337
	EF2715	*rplL*	Protein synthesis	Ribosomal proteins: synthesis and modification	ribosomal protein L7/L12	-4,0	**0**	-1,1	0,0186412	-0,3	0,419833	-1,4	0,0000343	0,2	0,5789709
	EF2716	*rplJ*	Protein synthesis	Ribosomal proteins: synthesis and modification	ribosomal protein L10	-4,7	**0**	-0,8	0,0978458	-0,3	0,2673408	-1,1	0,0006523	0,1	0,8677034
	EF2718	*rplA*	Protein synthesis	Ribosomal proteins: synthesis and modification	ribosomal protein L1	-4,2	**0**	-1,4	0,0021031	-0,6	0,0568782	-1,1	0,0011552	-0,3	0,3579011
	EF2719	*rplK*	Protein synthesis	Ribosomal proteins: synthesis and modification	ribosomal protein L11	-3,6	**0**	-1,3	0,0063829	-0,4	0,2594915	-0,5	0,1482795	-0,3	0,3815283
	EF2720		Transport and binding proteins	Unknown substrate	ABC transporter, ATP-binding protein	0,8	0,0798648	-0,3	0,5855723	0,5	0,087992	0,6	0,092723	0,7	0,0264576
	EF2721	*sdhB-2*	Energy metabolism	Amino acids and amines	L-serine dehydratase, iron-sulfur-dependent, beta subunit	-1,2	0,0018406	-1,0	0,0297459	0,2	0,4869371	0,0	0,9797263	-0,4	0,2071237
	EF2722	*sdhA-2*	Energy metabolism	Amino acids and amines	L-serine dehydratase, iron-sulfur-dependent, alpha subunit	-1,7	**0,0000029**	-1,2	0,0096369	0,2	0,4698229	0,0	0,9993861	-0,4	0,1929838
	EF2723		Hypothetical proteins	Conserved	conserved hypothetical protein	NA	NA	NA	NA	NA	NA	NA	NA	NA	NA
	EF2724		Protein fate	Degradation of proteins, peptides, and glycopeptides	peptidase, M42 family	0,3	0,4299532	0,4	0,4319319	NA	NA	NA	NA	-0,3	0,3996745
	EF2725		Transport and binding proteins	Amino acids, peptides and amines	pheromone binding protein	NA	NA	NA	NA	NA	NA	NA	NA	-0,3	0,4861225
	EF2727		Regulatory functions	Other	phosphosugar-binding transcriptional regulator, putative	0,9	0,0134531	0,3	0,5522629	1,4	0,0004092	0,8	0,0193756	0,2	0,5116831
	EF2728		Cell envelope	Other	lipoprotein, putative	-1,6	0,0003172	-1,2	0,0092402	-0,1	0,8288973	0,6	0,0993988	-0,1	0,8015481
	EF2729	*nusG*	Transcription	Transcription factors	transcription antitermination protein NusG	-3,4	**0**	-1,6	0,0004429	-0,4	0,1545593	-0,1	0,7554283	-0,4	0,2633174
	EF2730	*secE*	Protein fate	Protein and peptide secretion and trafficking	preprotein translocase, SecE subunit	0,3	0,4207816	-0,3	0,64444	-0,2	0,6071512	0,8	0,0191148	0,6	0,0804215
	EF2731	*rpmG-2*	Protein synthesis	Ribosomal proteins: synthesis and modification	ribosomal protein L33	-0,9	0,0475768	-1,0	0,0368202	0,6	0,0408804	0,7	0,026385	0,6	0,0713932
	EF2732		Unknown function	General	CBS domain protein	1,7	**0,000002**	0,4	0,3475816	0,1	0,6361249	0,3	0,3985273	0,3	0,4238264
	EF2733	*murB*	Cell envelope	Biosynthesis/degradation of murein sacculus/peptidoglycan	UDP-N-acetylenolpyruvoylglucosamine reductase	-0,5	0,1996335	-0,3	0,4642439	-0,2	0,5820719	0,1	0,6940537	-0,1	0,6738042
	EF2734		Unknown function	Enzymes of unknown specificity	oxidoreductase, Gfo/Idh/MocA family	-0,6	0,1715219	0,2	0,7349039	0,5	0,1508454	0,3	0,3739021	0,1	0,8285842
	EF2735	*exoA*	DNA metabolism	DNA replication, recombination, and repair	exodeoxyribonuclease	-1,1	0,0025381	0,3	0,4883348	0,1	0,8324614	-0,3	0,3241749	0,0	0,9325164
	EF2736		DNA metabolism	Degradation of DNA	exonuclease	-0,2	0,5869006	0,4	0,4262457	0,0	0,895527	-0,4	0,1866671	-0,1	0,8712608
	EF2738	*ahpF*	Unknown function	Enzymes of unknown specificity	thioredoxin reductase/glutathione-related protein	-1,8	**0,0000015**	-0,7	0,1293371	-2,0	**0**	-1,7	**0,0000004**	0,1	0,7876941
	EF2739	*ahpC*	Cellular processes	Detoxification	alkyl hydroperoxide reductase, C subunit	-1,4	0,000076	-0,7	0,1500626	-1,9	**0**	-1,2	0,0002133	0,2	0,5376695
	EF2740		Unknown function	Enzymes of unknown specificity	O-methyltransferase family protein	-0,3	0,4339806	-0,9	0,0502647	0,7	0,0368846	0,0	0,9911548	0,1	0,8053172
	EF2741	*lplA-2*	Protein fate	Protein modification and repair	lipoate-protein ligase A	0,7	0,0458638	0,1	0,846995	0,3	0,351816	0,6	0,0786973	-0,3	0,3817017
	EF2742		Hypothetical proteins	Conserved	conserved hypothetical protein	0,3	0,3616086	-0,1	0,8762951	0,2	0,5758683	0,5	0,1030761	-0,2	0,4534868
	EF2743		Hypothetical proteins	Domain	conserved domain protein	0,3	0,3816767	0,2	0,6399929	0,0	0,913935	-0,3	0,4021858	-0,1	0,7936007
	EF2744		Protein fate	Degradation of proteins, peptides, and glycopeptides	peptidase, M42 family	0,3	0,4454039	0,0	0,974335	0,1	0,8202028	0,0	0,935351	0,0	0,9884271
	EF2746	*dltD*	Cell envelope	Biosynthesis and degradation of surface poly/liposaccharides	dltD protein	-0,8	0,0425412	-0,2	0,7343875	0,5	0,1175921	0,1	0,7688544	-0,2	0,541086
	EF2747	*dltC*	Cell envelope	Biosynthesis and degradation of surface poly/liposaccharides	D-alanyl carrier protein	0,2	0,6788611	0,3	0,5321249	0,1	0,8533029	-0,4	0,2594252	-0,4	0,1750818

Operon[a)]	Locus	Gene	Functional category	Subcategory	Putative function	Blood[b)]	P-value[c)]	YTB_5[b)]	P-value[c)]	YTB_15[b)]	P-value[c)]	YTB_30[b)]	P-value[c)]	YTB_60[b)]	P-value[c)]
	EF2748	*dltB*	Cell envelope	Biosynthesis and degradation of surface poly/liposaccharides	basic membrane protein DltB	-2,2	**0**	0,3	0,4771036	0,2	0,5739965	-0,5	0,1166102	-0,6	0,0796266
	EF2749	*dltA*	Cell envelope	Biosynthesis and degradation of surface poly/liposaccharides	D-alanine-activating enzyme, putative	-2,9	**0**	0,3	0,5224776	0,1	0,698394	-0,7	0,0550427	-0,6	0,0805099
	EF2750		Hypothetical proteins	Conserved	conserved hypothetical protein	NA	NA	-0,3	0,5606824	NA	NA	0,1	0,7812563	-0,2	0,6387899
	EF2751		Transport and binding proteins	Unknown substrate	permease protein, putative	NA	NA	0,0	0,9321957	0,3	0,3553969	0,5	0,1758729	0,3	0,3337148
	EF2752		Transport and binding proteins	Unknown substrate	ABC transporter, ATP-binding protein	0,3	0,4489648	0,4	0,3467812	0,6	0,0530379	0,5	0,1335124	0,1	0,7228903
	EF2754	*nrdD*	Purines, pyrimidines, nucleosides, and nucleotides	2'-Deoxyribonucleotide metabolism	anaerobic ribonucleoside-triphosphate reductase	-0,9	0,0196756	-1,5	0,0016237	-1,9	**0**	-1,8	**0**	-0,4	0,1792111
	EF2755	*nrdG*	Purines, pyrimidines, nucleosides, and nucleotides	2'-Deoxyribonucleotide metabolism	anaerobic ribonucleoside-triphosphate reductase activating protein	-1,2	0,0007186	-1,3	0,0051674	-2,2	**0**	-2,3	**0**	-0,5	0,1560092
	EF2756	*dinP*	Unknown function	General	DNA-damage-inducible protein P	1,0	0,0051342	0,2	0,6296713	0,2	0,458324	-0,3	0,4533801	-0,3	0,4116487
	EF2757		Hypothetical proteins	Conserved	conserved hypothetical protein TIGR00245	-1,6	0,0003372	-0,6	0,1658046	0,1	0,7412592	-0,2	0,5300314	0,3	0,4352818
	EF2758		Transport and binding proteins	Unknown substrate	ABC transporter, ATP-binding protein	NA	NA	-0,3	0,5848638	0,6	0,0511478	0,8	0,0221167	0,1	0,7553779
	EF2759		Unknown function	Enzymes of unknown specificity	tetrapyrrole methylase family protein	-1,0	0,0041221	-0,3	0,4944629	0,0	0,8775188	-0,1	0,8432907	0,2	0,6788353
	EF2760		Hypothetical proteins	Conserved	conserved hypothetical protein	-0,1	0,7685682	0,1	0,8284759	0,0	0,9358555	0,2	0,5927071	0,0	0,8857514
	EF2761		Hypothetical proteins	Conserved	conserved hypothetical protein	-0,3	0,3572991	0,1	0,8667979	-0,1	0,7731659	0,2	0,5596643	-0,2	0,6316286
	EF2762	*holB*	DNA metabolism	DNA replication, recombination, and repair	DNA polymerase III, delta prime subunit	-0,1	0,731279	-0,3	0,5111527	-0,1	0,7121011	0,1	0,6966398	-0,1	0,7805459
	EF2763		Hypothetical proteins	Conserved	conserved hypothetical protein	0,9	0,0098609	0,0	0,9708786	0,0	0,9101011	0,2	0,4901924	0,0	0,9196306
	EF2764	*tmk*	Purines, pyrimidines, nucleosides, and nucleotides	Nucleotide and nucleoside interconversions	thymidylate kinase	0,9	0,0101687	-0,1	0,8686823	0,0	0,9867009	0,2	0,4871974	0,0	0,97847
	EF2765		Hypothetical proteins		hypothetical protein	-1,6	0,0004814	-1,1	0,0172324	-0,2	0,4377459	0,3	0,3822008	-0,1	0,8776508
	EF2766	*recR*	DNA metabolism	DNA replication, recombination, and repair	recombination protein RecR	0,5	0,1634962	-0,8	0,0770072	-0,5	0,114308	0,2	0,5440303	-0,1	0,7336851
	EF2767		Regulatory functions	Other	transcriptional regulator	NA	NA	0,1	0,7937186	0,4	0,3221073	0,6	0,0869031	-0,2	0,5769971
	EF2768		Hypothetical proteins	Conserved	conserved hypothetical protein	NA	NA	-0,1	0,8470233	0,4	0,1727781	0,3	0,3685552	0,2	0,5350046
	EF2769		Transport and binding proteins	Unknown substrate	ABC transporter, ATP-binding protein	NA	NA	0,2	0,6302017	0,6	0,097845	0,6	0,0709563	0,2	0,5493512
	EF2770		Hypothetical proteins	Conserved	conserved hypothetical protein	-1,1	0,0190649	-0,1	0,7542812	0,9	0,0038535	1,1	0,0014227	0,5	0,1684175
	EF2771		Hypothetical proteins	Conserved	conserved hypothetical protein	-3,1	**0**	0,3	0,536095	0,6	0,0548928	-0,2	0,4738302	-0,6	0,0716396
	EF2773		Transport and binding proteins	Unknown substrate	major facilitator family transporter	NA	NA	-0,1	0,8831662	NA	NA	0,4	0,3657264	-0,4	0,2058853
	EF2774		Hypothetical proteins	Conserved	conserved hypothetical protein	NA	NA	NA	NA	0,0	0,9293403	0,3	0,2987126	-0,5	0,1350889
	EF2775	*thiD*	Biosynthesis of cofactors, prosthetic groups, carriers	Thiamine	phosphomethylpyrimidine kinase	NA	NA	0,1	0,8092333	0,5	0,2196826	0,2	0,5497398	-0,1	0,749364
	EF2776	*thiE*	Biosynthesis of cofactors, prosthetic groups, carriers	Thiamine	thiamine-phosphate pyrophosphorylase	NA	NA	0,3	0,5746089	-0,2	0,5675965	0,6	0,0536355	0,0	0,9244926
	EF2777		Biosynthesis of cofactors, prosthetic groups, carriers	Thiamine	hydroxyethylthiazole kinase, putative	NA	NA	-0,2	0,6966495	0,2	0,6393941	0,7	0,0444896	0,0	0,9881858
	EF2778		Hypothetical proteins	Conserved	conserved hypothetical protein	NA	NA	0,2	0,6324126	0,0	0,9403864	0,4	0,2262818	0,3	0,3587045
	EF2780		Hypothetical proteins	Conserved	conserved hypothetical protein TIGR00103	-1,3	0,000646	-0,5	0,2850347	0,0	0,9081301	0,1	0,7381172	0,1	0,6910563
	EF2781	*dnaX*	DNA metabolism	DNA replication, recombination, and repair	DNA polymerase III, gamma and tau subunits	-0,8	0,0744454	-0,6	0,2147766	0,0	0,9760821	0,2	0,4742643	0,0	0,8901276
	EF2782		Energy metabolism	Sugars	galactose-1-phosphate uridylyltransferase, putative	-0,8	0,0736926	-0,7	0,1374083	0,1	0,828037	0,0	0,909501	0,1	0,722938
	EF2783	*galE-2*	Energy metabolism	Sugars	UDP-glucose 4-epimerase	-0,3	0,4721	-0,2	0,664772	-0,1	0,8697636	0,0	0,9151816	-0,3	0,3083769
	EF2784		Hypothetical proteins	Conserved	conserved hypothetical protein	2,6	**0**	0,0	0,9280311	-0,2	0,4419537	0,2	0,495754	0,4	0,238519
	EF2785		Hypothetical proteins		hypothetical protein	1,4	0,000167	0,5	0,2886783	0,3	0,3655574	0,1	0,8117149	-0,2	0,4718509
	EF2786		Hypothetical proteins	Conserved	conserved hypothetical protein	1,0	0,0044645	0,2	0,7322894	0,2	0,6228719	-0,1	0,8429353	0,0	0,9169154
	EF2787		Cellular processes	Detoxification	rhodanese family protein	-1,1	0,0027063	-0,4	0,3630607	0,0	0,9094425	-0,1	0,762601	-0,1	0,7136531
	EF2788	*glcK*	Energy metabolism	Glycolysis/gluconeogenesis	glucokinase	-1,2	0,0009234	-0,5	0,2666463	0,0	0,9186844	0,0	0,9715644	-0,2	0,5411703
	EF2789		Hypothetical proteins	Conserved	conserved hypothetical protein	-0,9	0,0103784	-0,2	0,6604834	-0,1	0,8542843	0,2	0,6120577	0,1	0,7743701
	EF2790		Transport and binding proteins	Unknown substrate	small hydrophobic molecule transporter protein, putative	0,0	0,9621536	0,0	0,9269119	-0,1	0,7545239	-0,1	0,7974447	0,0	0,9582565
	EF2791		Unknown function	Enzymes of unknown specificity	5-formyltetrahydrofolate cyclo-ligase family protein	-0,3	0,3437987	0,1	0,7933183	0,0	0,9288567	0,1	0,7009322	0,0	0,9563418
	EF2792		Hypothetical proteins	Conserved	conserved hypothetical protein	0,4	0,3262609	-0,2	0,6573585	-0,1	0,6632126	0,2	0,6038471	-0,3	0,4381578
	EF2793		Hypothetical proteins	Conserved	conserved hypothetical protein	-1,2	0,0009077	-0,6	0,229041	-0,4	0,1745992	-0,2	0,5435151	0,2	0,6504675
	EF2794		Cell envelope	Other	membrane protein, putative	-3,3	**0**	-0,4	0,4423856	-0,1	0,6581458	0,0	0,9582952	0,3	0,3348056
	EF2795		Cell envelope	Other	LysM domain lipoprotein	-2,0	**0**	-0,1	0,8909941	-0,3	0,4243983	-0,1	0,67103	0,2	0,4893591
	EF2796		Hypothetical proteins		hypothetical protein	0,2	0,5319765	0,4	0,373318	0,6	0,0509627	0,1	0,7416732	0,2	0,4875477
	EF2797		Hypothetical proteins		hypothetical protein	0,9	0,0364118	0,1	0,8758255	NA	NA	0,3	0,4015396	-0,2	0,4613599
	EF2798		Hypothetical proteins		hypothetical protein	-1,5	0,0001351	-0,9	0,0426337	-0,2	0,5172989	-0,3	0,4106138	-0,3	0,3381764
	EF2800		Mobile and extrachromosomal element functions	Transposon functions	ISEf1, transposase	-3,2	**0**	-0,7	0,128152	-0,1	0,7459434	-0,5	0,1587593	0,0	0,9923872
	EF2801		Hypothetical proteins		hypothetical protein	-1,1	0,0019186	-1,3	0,0049275	-0,2	0,5175892	0,0	0,9726312	0,1	0,8100301
	EF2802		Mobile and extrachromosomal element functions	Prophage functions	endolysin	-0,8	0,038257	0,3	0,5219749	-1,0	0,0013203	-0,9	0,0099128	-1,6	**0,0000018**
	EF2803		Mobile and extrachromosomal element functions	Prophage functions	holin	NA	NA	0,1	0,9105253	NA	NA	NA	NA	-0,4	0,3206745
	EF2804		Hypothetical proteins	Conserved	conserved hypothetical protein	NA	NA	1,0	0,0831842	NA	NA	NA	NA	-0,4	0,3613832
	EF2805		Hypothetical proteins	Conserved	conserved hypothetical protein	NA	NA	NA	NA	NA	NA	NA	NA	NA	NA
	EF2806		Hypothetical proteins		hypothetical protein	NA	NA	NA	NA	NA	NA	NA	NA	-0,2	0,7048552
	EF2807		Hypothetical proteins		hypothetical protein	0,6	0,075601	0,5	0,3750613	NA	NA	NA	NA	-0,2	0,65128
	EF2810		Hypothetical proteins	Conserved	conserved hypothetical protein	NA	NA	NA	NA	NA	NA	NA	NA	-0,3	0,3813592
	EF2811		Mobile and extrachromosomal element functions	Prophage functions	minor structural protein	NA	NA	NA	NA	NA	NA	NA	NA	0,1	0,7996672
	EF2812		Hypothetical proteins	Conserved	conserved hypothetical protein	-1,2	0,0025847	-1,0	0,0233161	0,1	0,6930987	0,3	0,3338906	0,0	0,9234764
	EF2813		Mobile and extrachromosomal element functions	Prophage functions	tail tape meausure protein	NA	NA	NA	NA	NA	NA	NA	NA	NA	NA
	EF2814		Hypothetical proteins		hypothetical protein	-0,1	0,7897402	0,0	0,9940448	NA	NA	NA	NA	NA	NA
	EF2815		Mobile and extrachromosomal element functions	Prophage functions	major tail protein, phi13 family	NA	NA	-0,3	0,641478	NA	NA	NA	NA	0,0	0,9003176
	EF2816		Hypothetical proteins	Conserved	conserved hypothetical protein TIGR01725	NA	NA	0,5	0,3595886	NA	NA	NA	NA	0,2	0,5466832
	EF2817		Hypothetical proteins	Conserved	conserved hypothetical protein	NA	NA	NA	NA	NA	NA	NA	NA	-0,3	0,4682447
	EF2818		Mobile and extrachromosomal element functions	Prophage functions	minor structural protein, putative	NA	NA	NA	NA	NA	NA	NA	NA	NA	NA
	EF2819		Hypothetical proteins		hypothetical protein	NA	NA	0,1	0,8309427	NA	NA	NA	NA	-0,9	0,0324126
	EF2820		Mobile and extrachromosomal element functions	Prophage functions	major capsid protein	NA	NA	-0,1	0,9263658	NA	NA	NA	NA	NA	NA
	EF2821		Protein fate	Other	prohead protease	NA	NA	0,8	0,1824723	NA	NA	NA	NA	-1,0	0,0044092
	EF2822		Mobile and extrachromosomal element functions	Prophage functions	portal protein	NA	NA	0,9	0,1341832	NA	NA	NA	NA	NA	NA
	EF2823		Mobile and extrachromosomal element functions	Prophage functions	terminase, large subunit, putative	NA	NA	-0,8	0,1354592	0,1	0,6936712	0,3	0,482196	-0,3	0,3178405
	EF2824		Hypothetical proteins		hypothetical protein	NA	NA	NA	NA	NA	NA	NA	NA	NA	NA
	EF2825		Hypothetical proteins	Conserved	conserved hypothetical protein	NA	NA	0,7	0,1906103	NA	NA	NA	NA	0,4	0,2720216
	EF2826		Hypothetical proteins		hypothetical protein	1,4	0,001406	0,4	0,5083877	NA	NA	NA	NA	0,5	0,1597114
	EF2828		Regulatory functions	DNA interactions	transcriptional regulator, ArpU family	NA	NA	NA	NA	NA	NA	NA	NA	0,3	0,5000371
	EF2829		Hypothetical proteins		hypothetical protein	NA	NA	NA	NA	NA	NA	NA	NA	NA	NA
	EF2830		Hypothetical proteins		hypothetical protein	NA	NA	NA	NA	NA	NA	NA	NA	NA	NA
	EF2831		Hypothetical proteins		hypothetical protein	NA	NA	0,6	0,3208643	NA	NA	NA	NA	0,5	0,1650127
	EF2832		Hypothetical proteins	Domain	conserved domain protein	1,3	0,0009248	-0,2	0,6817305	NA	NA	NA	NA	0,0	0,9527593
	EF2833		Hypothetical proteins	Conserved	conserved hypothetical protein	0,5	0,1612229	0,7	0,1425117	0,2	0,4825389	0,3	0,3888535	0,2	0,5852317
	EF2834		Hypothetical proteins		hypothetical protein	NA	NA	-0,5	0,2575347	-1,7	**0,0000108**	-1,2	0,0005884	-0,1	0,8294672
	EF2835		Hypothetical proteins	Conserved	conserved hypothetical protein	NA	NA	-1,2	0,0154448	0,1	0,7141775	0,5	0,1920017	-0,1	0,786797
	EF2836		Hypothetical proteins	Conserved	conserved hypothetical protein	NA	NA	NA	NA	NA	NA	NA	NA	NA	NA
	EF2837		Hypothetical proteins		hypothetical protein	0,7	0,0534294	-0,4	0,4112603	NA	NA	NA	NA	0,2	0,5938757

Operon[a]	Locus	Gene	Functional category	Subcategory	Putative function	Blood[b]	P-value[c]	YTB_5[b]	P-value[c]	YTB_15[b]	P-value[c]	YTB_30[b]	P-value[c]	YTB_60[b]	P-value[c]
	EF2838		Mobile and extrachromosomal element functions	Prophage functions	DNA replication protein DnaC, putative	0,6	0,1128012	0,2	0,6597619	NA	NA	0,8	0,0246583	0,1	0,7877574
	EF2839		Unknown function	General	DnaD domain protein	NA	NA	0,3	0,6477616	NA	NA	NA	NA	-0,4	0,2088557
	EF2840		Hypothetical proteins	Conserved	conserved hypothetical protein	NA	NA	0,6	0,3326334	NA	NA	NA	NA	NA	NA
	EF2841		Mobile and extrachromosomal element functions	Prophage functions	recT protein, putative	NA	NA	NA	NA	NA	NA	NA	NA	NA	NA
	EF2842		Hypothetical proteins		hypothetical protein	NA	NA	NA	NA	NA	NA	NA	NA	NA	NA
	EF2843		Hypothetical proteins		hypothetical protein	0,7	0,0544638	-0,2	0,7087073	NA	NA	NA	NA	-0,2	0,5812619
	EF2844		Hypothetical proteins		hypothetical protein	0,6	0,0911837	-0,3	0,5505781	-0,2	0,6938494	NA	NA	0,1	0,729105
	EF2845		Hypothetical proteins		hypothetical protein	NA	NA	0,4	0,3713227	NA	NA	NA	NA	-0,2	0,5092691
	EF2846		Hypothetical proteins	Domain	conserved domain protein	1,3	0,0005304	-0,1	0,7602842	0,5	0,1852013	0,2	0,5826738	0,0	0,9153715
	EF2847		Hypothetical proteins	Domain	conserved domain protein	1,0	0,0066688	-0,3	0,4561925	0,0	0,9756576	0,0	0,926185	-0,2	0,4949471
	EF2848		Hypothetical proteins		hypothetical protein	0,6	0,1848535	-0,5	0,276033	0,1	0,6368315	0,6	0,0960162	-0,1	0,8681157
	EF2849		Hypothetical proteins	Conserved	conserved hypothetical protein	0,4	0,3596336	0,0	0,9520686	0,3	0,4039955	0,3	0,3992004	-0,1	0,8238682
	EF2850		Hypothetical proteins	Conserved	conserved hypothetical protein	0,4	0,3209713	0,0	0,9597776	-0,1	0,7100427	-0,2	0,6315872	0,1	0,7867304
	EF2851		Hypothetical proteins	Conserved	conserved hypothetical protein	0,1	0,7421556	0,0	0,9900707	0,0	0,9983102	-0,6	0,0581024	0,0	0,962768
	EF2852		Mobile and extrachromosomal element functions	Prophage functions	transcriptional regulator, Cro/CI family	0,0	0,9519586	0,3	0,479163	0,3	0,357804	-0,4	0,2267275	0,0	0,8990019
	EF2853		Hypothetical proteins	Conserved	conserved hypothetical protein	NA	NA	-0,3	0,568023	0,0	0,9441151	0,0	0,9617289	0,1	0,765747
	EF2854		Transport and binding proteins	Cations and iron carrying compounds	ion transporter, putative	-0,6	0,1881112	-0,3	0,4963765	0,3	0,3959803	0,0	0,9932723	0,0	0,9905382
	EF2855		Mobile and extrachromosomal element functions	Prophage functions	site-specific recombinase, phage integrase family	1,1	0,0018153	0,3	0,5071408	-0,2	0,6321454	-0,3	0,4100547	0,0	0,9677882
	EF2856	*rpmG-3*	Protein synthesis	Ribosomal proteins: synthesis and modification	ribosomal protein L33	-1,4	0,0000886	-0,5	0,2915538	-0,3	0,2982588	-0,6	0,0896808	0,2	0,4900669
	EF2857		Cell envelope	Biosynthesis/degradation of murein sacculus/peptidoglycan	penicillin-binding protein 2B	-0,2	0,6353863	0,5	0,3067599	0,0	0,8854411	0,0	0,9460419	-0,2	0,5600852
	EF2858	*thrS*	Protein synthesis	tRNA aminoacylation	threonyl-tRNA synthetase	-1,0	0,0063836	-0,5	0,3076676	-0,4	0,1929234	-0,4	0,2128342	-0,2	0,5350959
	EF2859		Energy metabolism	Other	4-oxalocrotonate tautomerase, putative	-0,6	0,0819933	-0,3	0,5577427	-0,4	0,2211102	-0,7	0,0305762	-0,2	0,4633237
	EF2860		Unknown function	General	ErfK/YbiS/YcfS/YnhG family protein, putative	-0,2	0,5184265	-0,2	0,7127105	-0,1	0,6423922	0,2	0,5899455	-0,1	0,6907137
	EF2861	*aadK*	Cellular processes	Toxin production and resistance	aminoglycoside 6-adenylyltransferase	NA	NA	-0,6	0,20844	0,2	0,4748167	0,2	0,6160096	-0,1	0,6561088
	EF2862		Hypothetical proteins	Conserved	conserved hypothetical protein	1,0	0,0075676	0,1	0,8791119	0,3	0,4133439	0,0	0,92057	-0,3	0,3768846
	EF2863		Energy metabolism	Biosynthesis and degradation of polysaccharides	endo-beta-N-acetylglucosaminidase	1,7	**0,0000024**	0,6	0,2172735	0,5	0,1022015	0,2	0,6164132	-1,1	0,0008098
	EF2864		Hypothetical proteins	Domain	conserved domain protein	0,7	0,0402389	0,2	0,6792309	-0,3	0,3092068	0,1	0,7290558	0,1	0,8664557
	EF2866		Hypothetical proteins	Conserved	conserved hypothetical protein TIGR01033	-1,3	0,0003069	-1,0	0,0382961	-0,2	0,5546362	0,3	0,3059702	-0,3	0,4348717
	EF2867		Hypothetical proteins	Conserved	conserved hypothetical protein	-1,7	**0,000002**	-0,3	0,4679908	0,2	0,5172261	-0,4	0,3020235	0,1	0,7900876
	EF2868		Hypothetical proteins	Conserved	conserved hypothetical protein	-1,0	0,0076211	-0,7	0,1231023	-0,1	0,8381887	-0,1	0,6686867	-0,3	0,3487873
	EF2869		Disrupted reading frame		conserved hypothetical protein, truncation	-0,7	0,0632946	-0,8	0,0842667	0,0	0,9571975	0,0	0,8907919	-0,1	0,8159047
	EF2870		Unknown function	General	HD domain protein	-1,0	0,0065383	-0,7	0,1174113	-0,4	0,2186496	-0,1	0,7989721	-0,2	0,5653235
	EF2871	*nadD*	Biosynthesis of cofactors, prosthetic groups, carriers	Pyridine nucleotides	nicotinate-nucleotide adenylyltransferase	-1,5	0,0007427	-0,9	0,0541197	-0,2	0,462235	-0,4	0,261201	-0,1	0,6788174
	EF2872		Hypothetical proteins	Conserved	conserved hypothetical protein TIGR00253	-0,8	0,0288848	-0,9	0,0555322	-0,2	0,4312922	-0,3	0,4277906	-0,1	0,7348865
	EF2873		Unknown function	General	GTPase of unknown function	-1,0	0,0047003	-1,6	0,0007811	-0,4	0,2005288	0,2	0,5531798	-0,4	0,1940588
	EF2874		Unknown function	Enzymes of unknown specificity	hydrolase, HAD subfamily IIIA	-0,1	0,8014538	-0,7	0,1110696	0,1	0,6736709	0,7	0,0274042	-0,1	0,7436812
	EF2875	*accA*	Fatty acid and phospholipid metabolism	Biosynthesis	acetyl-CoA carboxylase, carboxyl transferase alpha subunit	1,7	**0,0000047**	2,7	**0**	4,2	**0**	2,6	**0**	1,3	0,0000974
	EF2876	*accD*	Fatty acid and phospholipid metabolism	Biosynthesis	acetyl-CoA carboxylase, carboxyl transferase beta subunit	1,2	0,0015694	3,1	**0**	4,4	**0**	2,7	**0**	1,1	0,0014374
	EF2877	*accC*	Fatty acid and phospholipid metabolism	Biosynthesis	acetyl-CoA carboxylase, biotin carboxylase	1,5	0,0000325	3,2	**0**	3,8	**0**	2,6	**0**	1,3	0,0000846
	EF2878	*fabZ-2*	Fatty acid and phospholipid metabolism	Biosynthesis	(3R)-hydroxymyristoyl-(acyl-carrier-protein) dehydratase	1,7	**0,0000104**	3,5	**0**	4,1	**0**	2,9	**0**	1,2	0,0003062
	EF2879	*accB*	Fatty acid and phospholipid metabolism	Biosynthesis	acetyl-CoA carboxylase, biotin carboxyl carrier protein	1,5	0,0011997	3,0	**0**	3,8	**0**	2,6	**0**	1,1	0,0006683
	EF2880	*fabF-2*	Fatty acid and phospholipid metabolism	Biosynthesis	3-oxoacyl-(acyl-carrier-protein) synthase II	2,2	**0**	3,4	**0**	3,9	**0**	3,0	**0**	1,2	0,0004569
	EF2881	*fabG*	Fatty acid and phospholipid metabolism	Biosynthesis	3-oxoacyl-(acyl-carrier-protein) reductase	2,7	**0**	3,2	**0**	3,5	**0**	2,8	**0**	1,4	0,0000201
	EF2882	*fabD*	Fatty acid and phospholipid metabolism	Biosynthesis	malonyl CoA-acyl carrier protein transacylase	1,2	0,0099115	3,3	**0**	4,1	**0**	3,6	**0**	1,4	0,0000256
	EF2883	*fabK*	Fatty acid and phospholipid metabolism	Biosynthesis	enoyl-(acyl-carrier-protein) reductase II	1,0	0,009027	2,8	**0**	3,6	**0**	3,0	**0**	1,2	0,0001867
	EF2884		Fatty acid and phospholipid metabolism	Biosynthesis	acyl carrier protein, putative	NA	NA	NA	NA	NA	NA	NA	NA	NA	NA
	EF2885	*fabH*	Fatty acid and phospholipid metabolism	Biosynthesis	3-oxoacyl-(acyl-carrier-protein) synthase III	3,2	**0**	3,8	**0**	4,3	**0**	4,3	**0**	1,0	0,0015566
	EF2886		Regulatory functions	DNA interactions	transcriptional regulator, MarR family	2,6	**0**	4,0	**0**	3,8	**0**	4,2	**0**	1,1	0,0009453
	EF2888		Hypothetical proteins	Conserved	conserved hypothetical protein	0,0	0,9204517	-0,4	0,374571	-0,1	0,8270327	0,1	0,6667125	0,2	0,6336256
	EF2889	*glxR*	Energy metabolism	Other	2-hydroxy-3-oxopropionate reductase	0,8	0,0333433	0,8	0,1027624	0,2	0,5632797	0,5	0,1052695	0,2	0,638898
	EF2890		Cell envelope	Biosynthesis and degradation of surface poly/liposaccharides	glycosyl transferase, group 1 family protein	-1,1	0,0018997	0,0	0,9595054	0,1	0,7840899	0,0	0,9875146	0,2	0,5206097
	EF2891		Cell envelope	Biosynthesis and degradation of surface poly/liposaccharides	glycosyl transferase, group 1 family protein	-1,4	0,0001827	-0,1	0,7878036	0,3	0,3941807	0,5	0,1732821	0,3	0,3033366
	EF2892		Hypothetical proteins		hypothetical protein	NA	NA	NA	NA	NA	NA	NA	NA	NA	NA
	EF2893		Hypothetical proteins		hypothetical protein	NA	NA	-0,3	0,5888048	0,1	0,7660971	0,6	0,0808147	0,2	0,4638203
	EF2894		Cellular processes	Adaptations to atypical conditions	general stress protein 13, putative	-0,7	0,0573711	0,0	0,9508468	0,0	0,988407	0,3	0,4092988	0,1	0,7047659
	EF2895		Unknown function	Enzymes of unknown specificity	aminotransferase, class II	-0,5	0,2192422	-0,1	0,9096737	0,2	0,5126771	0,0	0,9156458	0,0	0,8920546
	EF2896		Hypothetical proteins		hypothetical protein	NA	NA	0,0	0,9472738	NA	NA	NA	NA	0,1	0,6839707
	EF2898		Protein fate	Protein folding and stabilization	peptidyl-prolyl cis-trans isomerase, cyclophilin-type	1,1	0,0028631	0,9	0,0442825	0,2	0,5727977	0,4	0,2034881	0,3	0,4315855
	EF2899		Unknown function	Enzymes of unknown specificity	oxidoreductase, pyridine nucleotide-disulfide family	-2,8	**0**	-1,0	0,0360881	0,2	0,4753747	0,2	0,5017884	-0,3	0,3212087
	EF2900		Hypothetical proteins		hypothetical protein	NA	NA	NA	NA	NA	NA	NA	NA	NA	NA
	EF2901		Unknown function	Enzymes of unknown specificity	D-isomer specific 2-hydroxyacid dehydrogenase family protein	1,4	0,0001697	0,3	0,5557229	0,5	0,1144728	0,5	0,1575368	0,0	0,8862525
	EF2902		Purines, pyrimidines, nucleosides, and nucleotides	Other	2,3-cyclic-nucleotide 2-phosphodiesterase, putative	-1,8	0,0000499	0,2	0,7342802	0,0	0,9815772	0,1	0,7539605	0,0	0,912068
	EF2903		Transport and binding proteins	Unknown substrate	ABC transporter, substrate-binding protein	-0,9	0,0132012	0,4	0,3394611	0,1	0,7033949	0,5	0,1801051	-0,5	0,1850166
	EF2904		Hypothetical proteins		hypothetical protein	-1,0	0,0044882	0,4	0,3616637	0,1	0,816513	0,1	0,7450525	-0,4	0,1825362
	EF2905		Transport and binding proteins	Unknown substrate	ABC transporter, permease protein	-1,5	0,0000251	0,1	0,8520769	-0,2	0,6185743	-0,2	0,5858346	-0,5	0,1262842
	EF2906		Transport and binding proteins	Unknown substrate	ABC transporter, permease protein	-1,5	0,0000393	-0,3	0,5506325	-0,1	0,8505291	-0,2	0,6520848	-0,6	0,0588769
	EF2907		Transport and binding proteins	Unknown substrate	ABC transporter, ATP-binding protein	-1,6	**0,0000063**	-0,8	0,0733396	0,1	0,6571052	0,3	0,4544532	-0,5	0,122862
	EF2908		Cell envelope	Biosynthesis and degradation of surface poly/liposaccharides	glycosyl transferase, group 2 family protein	-1,9	**0,0000002**	-0,5	0,2752788	0,0	0,9091211	0,1	0,6765381	0,1	0,8000138
	EF2909		Hypothetical proteins	Conserved	conserved hypothetical protein	0,7	0,060053	0,0	0,9698983	0,5	0,1513967	0,4	0,2746444	0,0	0,8828435
	EF2910		Transport and binding proteins	Cations and iron carrying compounds	potassium uptake protein	-0,2	0,5615807	0,3	0,5824037	0,2	0,5936854	0,0	0,9359549	0,0	0,8912994
	EF2911		Regulatory functions	DNA interactions	DNA-binding response regulator, LuxR family	-0,5	0,1722316	0,8	0,1003032	-0,4	0,2209507	0,0	0,9980031	0,1	0,8804618
	EF2912		Signal transduction	Two-component systems	sensor histidine kinase, putative	-0,7	0,0703955	0,6	0,1607248	0,0	0,8790916	0,0	0,9954479	0,2	0,5535847
	EF2913		Hypothetical proteins	Conserved	conserved hypothetical protein	0,7	0,0642655	0,7	0,1582843	0,3	0,3986929	0,7	0,0288229	0,8	0,0232618
	EF2914	*greA*	Transcription	Transcription factors	transcription elongation factor GreA	-1,8	**0,0000025**	-1,5	0,0013934	-0,3	0,4059598	0,5	0,1592663	-0,2	0,4889193
	EF2915		Hypothetical proteins	Conserved	conserved hypothetical protein TIGR00247	-0,7	0,0727319	-0,9	0,043465	0,2	0,4701095	0,4	0,2100631	0,1	0,7182303
	EF2916		Unknown function	Enzymes of unknown specificity	hydrolase, haloacid dehalogenase-like family	-0,1	0,7144213	-0,3	0,5102533	0,3	0,4197477	-0,1	0,7732039	0,1	0,8638124
	EF2917		Cell envelope	Biosynthesis and degradation of surface poly/liposaccharides	UDP-N-acetylglucosamine 2-epimerase	0,1	0,7135363	-0,1	0,9088613	0,4	0,1635309	0,2	0,608741	0,2	0,4820204
	EF2918		Unknown function	Enzymes of unknown specificity	bacterial transferase, putative	-0,6	0,1832238	-0,7	0,108086	0,7	0,0207956	0,3	0,4383365	0,3	0,3548193
	EF2919		Transport and binding proteins	Unknown substrate	ABC transporter, ATP-binding/permease protein	-1,5	0,0000658	-0,5	0,3078034	0,3	0,3355802	-0,8	0,0111675	0,2	0,6336099
	EF2920		Transport and binding proteins	Unknown substrate	ABC transporter, ATP-binding/permease protein	-1,6	**0,0000147**	-0,8	0,087406	0,3	0,311033	-0,5	0,1176317	0,0	0,8831533
	EF2921		Hypothetical proteins		hypothetical protein	NA	NA	-0,1	0,8334566	NA	NA	NA	NA	0,3	0,4364134
	EF2922		Hypothetical proteins	Conserved	conserved hypothetical protein	1,3	0,0049116	-0,5	0,2386821	0,0	0,9920116	0,6	0,1002361	0,6	0,0896101
	EF2923		Hypothetical proteins	Conserved	conserved hypothetical protein	-1,5	0,0000846	-1,0	0,0250906	0,2	0,5266143	-0,2	0,5204873	-0,1	0,8225129

Operon[a]	Locus	Gene	Functional category	Subcategory	Putative function	Blood[b]	P-value[c]	YTB_5[b]	P-value[c]	YTB_15[b]	P-value[c]	YTB_30[b]	P-value[c]	YTB_60[b]	P-value[c]
	EF2924		Unknown function	Enzymes of unknown specificity	metallo-beta-lactamase superfamily protein	-2,0	**0,0000001**	-1,1	0,0162105	0,3	0,2899007	-0,1	0,7952147	0,0	0,9588736
	EF2925		Cellular processes	Adaptations to atypical conditions	cold-shock domain family protein	-0,7	0,0443267	-0,3	0,5846874	0,2	0,6114296	0,4	0,2482553	-0,3	0,3573772
	EF2926	*radC*	DNA metabolism	DNA replication, recombination, and repair	DNA repair protein RadC	NA	NA	NA	NA	NA	NA	1,2	0,0045891	0,4	0,297705
	EF2927		Unknown function	Enzymes of unknown specificity	hydrolase, haloacid dehalogenase-like family	0,5	0,1475114	0,3	0,5597781	0,3	0,2703387	0,6	0,0583711	0,0	0,9346923
	EF2928		Biosynthesis of cofactors, prosthetic groups, carriers	Folic acid	FolC family protein	0,4	0,3119142	0,7	0,1431137	0,1	0,7407362	0,3	0,3854601	0,1	0,8264026
	EF2929		Cell envelope	Other	membrane protein, putative	-1,0	0,0054153	0,1	0,9114683	0,0	0,9196647	-0,1	0,8483769	0,2	0,6504338
	EF2930		Hypothetical proteins	Conserved	conserved hypothetical protein	-1,2	0,0009617	-0,2	0,7412394	0,4	0,2409849	0,0	0,9331289	0,4	0,2808096
	EF2931	*valS*	Protein synthesis	tRNA aminoacylation	valyl-tRNA synthetase	-1,8	**0,0000007**	-0,5	0,2485577	-0,1	0,6963126	-0,5	0,1708417	0,2	0,5681685
	EF2932		Unknown function	Enzymes of unknown specificity	AhpC/TSA family protein	0,4	0,3038676	0,6	0,2273751	-0,2	0,4743899	0,7	0,0419401	-0,1	0,8289455
	EF2933		Unknown function	General	DNA-binding protein,putative	-2,3	**0**	-1,6	0,0005745	-1,5	**0,000001**	0,1	0,6961677	0,0	0,9427578
	EF2934	*thiI*	Biosynthesis of cofactors, prosthetic groups, carriers	Thiamine	thiazole biosynthesis protein ThiI	-0,7	0,0588093	-1,1	0,019155	-0,1	0,6564711	0,2	0,5388157	-0,1	0,8437237
	EF2935		Transport and binding proteins	Nucleosides, purines and pyrimidines	xanthine/uracil permeases family protein	-0,3	0,4850419	-0,5	0,2690651	-0,1	0,7617114	-0,5	0,1370931	0,1	0,7447665
	EF2936		Hypothetical proteins		hypothetical protein	1,2	0,0009312	0,5	0,2889105	0,4	0,183652	0,3	0,3360769	0,4	0,2778423
	EF2937		Hypothetical proteins		hypothetical protein	0,9	0,0385521	0,3	0,5122099	0,6	0,0428734	0,5	0,1603045	0,3	0,3577702
	EF2938		Hypothetical proteins	Conserved	conserved hypothetical protein	0,4	0,3422306	0,1	0,8728425	0,8	0,0118406	0,5	0,1671754	0,5	0,1608506
	EF2939		Cellular processes	Adaptations to atypical conditions	cold-shock domain family protein	0,6	0,0783406	-0,1	0,7586412	-0,2	0,5999284	0,1	0,7591696	-0,3	0,4090448
	EF2940		Hypothetical proteins		hypothetical protein	0,5	0,193873	0,3	0,4657191	-0,3	0,3351816	-0,1	0,8499553	-0,4	0,2308704
	EF2941		Hypothetical proteins		hypothetical protein	NA	NA	-0,3	0,5048214	0,1	0,8783968	0,0	0,9520089	-0,1	0,7511436
	EF2942		Hypothetical proteins		hypothetical protein	-0,3	0,3970592	0,1	0,7735133	0,4	0,1756724	0,3	0,4556927	-0,4	0,2487964
	EF2943		Hypothetical proteins		hypothetical protein	-0,4	0,3199338	0,0	0,9478435	0,1	0,7028312	-0,2	0,4988254	-0,2	0,6413412
	EF2944		Hypothetical proteins		hypothetical protein	-0,3	0,5244062	-0,1	0,901493	0,0	0,9248694	0,2	0,5827024	-0,2	0,5440648
	EF2945		Hypothetical proteins		hypothetical protein	NA	NA	-0,1	0,8878512	NA	NA	-0,2	0,5540474	0,3	0,4326815
	EF2946		Hypothetical proteins		hypothetical protein	0,9	0,0228002	-0,2	0,7019133	-0,3	0,361723	NA	NA	0,0	0,9559697
	EF2947		Hypothetical proteins	Domain	conserved domain protein	1,0	0,0339006	-0,3	0,5638099	0,4	0,2724921	0,1	0,7537316	-0,5	0,1157859
	EF2948		Unknown function	General	DNA primase domain protein	1,5	0,0000338	-0,1	0,8860849	-0,1	0,8470282	-0,7	0,0492978	-0,6	0,0652459
	EF2949		Hypothetical proteins		hypothetical protein	1,3	0,0051779	-0,6	0,2554101	0,1	0,8549624	-0,3	0,3479916	-0,1	0,8268545
	EF2950		Hypothetical proteins		hypothetical protein	1,7	0,0000926	-0,2	0,5951497	0,4	0,3601854	0,3	0,4134358	-0,1	0,7643582
	EF2951		Hypothetical proteins	Conserved	conserved hypothetical protein	1,2	0,0011762	-0,3	0,5146347	0,2	0,6334781	-0,1	0,8468367	-0,1	0,7912465
	EF2952		Hypothetical proteins		hypothetical protein	0,8	0,0294066	0,0	0,9506227	-0,1	0,6824734	NA	NA	0,1	0,6754002
	EF2953		Hypothetical proteins		hypothetical protein	2,2	**0,0000013**	0,1	0,7465676	0,1	0,8600869	-0,3	0,4444742	-0,4	0,2301507
	EF2954		Regulatory functions	DNA interactions	transcriptional regulator, Cro/CI family	0,6	0,1294834	0,3	0,5541943	0,1	0,7090002	-0,3	0,393944	-0,2	0,456446
	EF2955		Mobile and extrachromosomal element functions	Prophage functions	site-specific recombinase, phage integrase family	0,6	0,1191197	0,3	0,5774518	0,2	0,5494514	-0,2	0,6398747	0,0	0,9170727
	EF2956		Unknown function	Enzymes of unknown specificity	oxidoreductase, short-chain dehydrogenase/reductase family	-0,4	0,2587396	-0,5	0,2706196	0,3	0,3551278	0,2	0,6332273	0,4	0,1884833
	EF2957		Unknown function	Enzymes of unknown specificity	hexapeptide-repeat containing-acetyltransferase	-0,8	0,0419479	-0,3	0,45871	0,1	0,8440875	0,0	0,9730372	0,4	0,2124948
	EF2958		Regulatory functions	DNA interactions	transcriptional regulator, LysR family	0,5	0,1948449	-0,2	0,6693604	-0,2	0,5304587	-0,2	0,6208991	0,0	0,9579505
	EF2959		Transport and binding proteins	Carbohydrates, organic alcohols, and acids	ribose uptake protein, putative	-0,5	0,2270798	0,7	0,1215324	0,2	0,5016689	0,0	0,9923823	-0,4	0,2132679
	EF2960		Transport and binding proteins	Carbohydrates, organic alcohols, and acids	ribose transporter protein RbsD	-1,8	0,0000595	0,4	0,3306866	0,2	0,5000388	0,0	0,9498342	-0,4	0,2304329
	EF2961	*rbsK*	Energy metabolism	Sugars	ribokinase	-1,4	0,0002199	0,4	0,3693014	0,1	0,8227993	0,1	0,860681	-0,3	0,3032413
	EF2962		Regulatory functions	DNA interactions	sugar-binding transcriptional regulator, LacI family	-2,5	**0**	-1,2	0,010741	0,0	0,8999275	0,6	0,0886185	0,0	0,9600569
	EF2963		Unknown function	Enzymes of unknown specificity	esterase, putative	0,0	0,9835984	-0,1	0,8921082	0,6	0,0755127	0,4	0,3884496	0,4	0,2885188
	EF2964		Transport and binding proteins	Unknown substrate	putative transport protein, SgaT family	1,2	0,0013323	1,7	0,000265	0,5	0,1442282	-0,6	0,082268	-0,8	0,0124311
	EF2965		Hypothetical proteins	Conserved	conserved hypothetical protein	0,6	0,1147224	1,3	0,0056768	0,2	0,4472069	-1,2	0,0005369	-0,8	0,0158198
	EF2966		Regulatory functions	RNA interactions	transcriptional antiterminator, bglG family	-0,4	0,2866268	0,7	0,1444008	0,3	0,3710184	-1,7	**0,0000007**	-0,4	0,2058695
	EF2967		Hypothetical proteins		hypothetical protein	NA	NA	0,5	0,2639076	-0,6	0,1122557	-0,5	0,1186028	0,4	0,2170262
	EF2968		Cell envelope	Other	cell wall surface anchor family protein	NA	NA	-0,1	0,9034376	NA	NA	NA	NA	0,2	0,5122159
	EF2969		Hypothetical proteins	Conserved	conserved hypothetical protein	NA	NA	0,7	0,1964849	NA	NA	NA	NA	-0,5	0,1383265
	EF2970		Hypothetical proteins	Domain	conserved domain protein	NA	NA	0,0	0,9972766	0,0	0,9291119	0,0	0,9858715	0,1	0,7070312
	EF2972		Cellular processes	Toxin production and resistance	amidinotransferase family protein	1,1	0,0024485	1,2	0,0090518	0,5	0,1344761	0,5	0,1556533	0,2	0,4884405
	EF2973	*phoZ*	Central intermediary metabolism	Phosphorus compounds	alkaline phosphatase	NA	NA	0,3	0,5493119	-0,3	0,4606198	-0,3	0,3369482	0,7	0,0375904
	EF2974		DNA metabolism	Other	MutS2 family protein	0,4	0,3593439	0,3	0,4833696	-0,3	0,3103756	NA	NA	0,6	0,075875
	EF2975		Hypothetical proteins	Conserved	conserved hypothetical protein	1,5	0,0000226	0,3	0,5021805	0,1	0,6706004	0,1	0,8364087	-0,6	0,0882623
	EF2976		Unknown function	Enzymes of unknown specificity	pyridoxal phosphate-dependent enzyme, putative	1,6	**0,0000099**	0,5	0,2976717	0,1	0,6986357	-0,1	0,8187253	-0,6	0,0796355
	EF2977	*mpoD*	Signal transduction	PTS	PTS system, IID component	2,6	**0**	2,9	**0**	3,1	**0**	2,5	**0**	1,5	**0,0000091**
	EF2978	*mpoC*	Signal transduction	PTS	PTS system, IIC component	1,1	0,0146529	3,4	**0**	4,2	**0**	3,5	**0**	1,4	0,0000222
	EF2979	*mpoB*	Signal transduction	PTS	PTS system, IIB component	1,2	0,0014747	2,9	**0**	3,7	**0**	3,3	**0**	1,2	0,0002627
	EF2980	*mpoA*	Signal transduction	PTS	PTS system, IIA component	NA	NA	-0,3	0,5098576	NA	NA	-0,6	0,0940067	0,0	0,9373933
	EF2981	*mpoR*	Transcription	Transcription factors	sigma-54 dependent DNA-binding response regulator	3,1	**0**	3,9	**0**	4,3	**0**	4,4	**0**	1,1	0,0013087
	EF2982	*pgm*	Energy metabolism	Glycolysis/gluconeogenesis	phosphoglycerate mutase family protein	2,6	**0**	3,9	**0**	3,7	**0**	4,2	**0**	1,1	0,0013371
	EF2983		Protein synthesis	tRNA aminoacylation	glutamyl-tRNA(Gln) amidotransferase, A subunit, putative	NA	NA	0,8	0,0793411	NA	NA	0,5	0,1078582	0,2	0,4759155
	EF2984		Regulatory functions	Other	transcriptional regulator, putative	-0,1	0,7390644	-0,6	0,2231239	0,1	0,8725745	0,2	0,5451772	0,1	0,8029001
	EF2985		Transport and binding proteins	Unknown substrate	permease, putative	0,9	0,0127884	0,8	0,0976722	0,3	0,4254249	0,6	0,0918337	0,2	0,4865617
	EF2986		Transport and binding proteins	Unknown substrate	ABC transporter, ATP-binding protein	-1,0	0,0060242	-0,1	0,8641777	0,1	0,7013211	0,0	0,9063031	0,2	0,523936
	EF2987		Hypothetical proteins	Conserved	conserved hypothetical protein	-1,3	0,0006744	-0,1	0,7568482	0,4	0,2067503	0,5	0,150717	0,3	0,2942559
	EF2988		Cellular processes	Detoxification	rhodanese family protein	1,4	0,0001108	-0,8	0,1796488	NA	NA	NA	NA	NA	NA
	EF2989		Central intermediary metabolism	Other	coenzyme A disulfide reductase	NA	NA	-0,2	0,6655306	0,9	0,0230261	NA	NA	0,3	0,3763321
	EF2990		Cellular processes	Detoxification	rhodanese family protein	-0,9	0,0248758	-0,1	0,8833202	0,1	0,7544606	0,2	0,5586837	0,2	0,5502539
	EF2991		Hypothetical proteins	Conserved	conserved hypothetical protein	-0,7	0,0706039	-0,1	0,8114334	0,2	0,4575479	0,0	0,9994371	0,0	0,9905823
	EF2992		Transport and binding proteins	Unknown substrate	major facilitator family transporter	NA	NA	0,3	0,54141	NA	NA	NA	NA	0,2	0,5111763
	EF2994		Unknown function	Enzymes of unknown specificity	aminotransferase, class V	1,1	0,0040728	0,8	0,0678578	0,1	0,8346483	0,5	0,1683826	0,2	0,5087714
	EF2995		Hypothetical proteins	Conserved	conserved hypothetical protein	-3,0	**0**	-1,0	0,0244949	0,3	0,2954696	0,2	0,4766255	-0,3	0,4331106
	EF2996		Hypothetical proteins	Conserved	conserved hypothetical protein	NA	NA	0,3	0,4747848	NA	NA	NA	NA	-0,2	0,4831162
	EF2997		Protein fate	Degradation of proteins, peptides, and glycopeptides	peptidase, M20/M25/M40 family	1,4	0,0001752	0,2	0,5907403	0,5	0,0967369	0,5	0,1321685	0,0	0,9416818
	EF2999		Purines, pyrimidines, nucleosides, and nucleotides	Other	allantoinase, putative	-0,9	0,0190077	0,5	0,3102228	0,1	0,7735529	0,4	0,239414	-0,4	0,3077386
	EF3000		Transport and binding proteins	Nucleosides, purines and pyrimidines	cytosine/purines, uracil, thiamine, allantoin permease family protein	-0,7	0,0529185	0,3	0,5413608	0,1	0,8283575	0,2	0,4909326	-0,5	0,1343462
	EF3001		Regulatory functions	Other	protease synthase and sporulation negative regulatory protein pai 1	NA	NA	0,1	0,8276068	-0,3	0,4927032	-0,2	0,6934588	-0,6	0,0732449
	EF3002		Regulatory functions	DNA interactions	transcriptional regulator, MarR family	-1,4	0,0000802	-0,2	0,6087583	-0,1	0,8329577	-0,1	0,7601871	-0,6	0,0662407
	EF3003		Cell envelope	Other	lipoprotein, putative	-1,5	0,0000311	-0,8	0,0676545	0,2	0,5826166	0,3	0,3019821	-0,5	0,1329178
	EF3004		Transport and binding proteins	Anions	sulfate transporter family/STAS domain protein	-1,8	**0,0000008**	-0,5	0,2951128	0,0	0,9450357	0,2	0,6517655	0,1	0,6897571
	EF3005		Energy metabolism	Other	choloylglycine hydrolase family protein	0,7	0,0581284	0,0	0,9897041	0,4	0,174658	0,3	0,317814	0,0	0,8990317
	EF3006		Hypothetical proteins	Conserved	conserved hypothetical protein	-0,3	0,3826956	0,2	0,6234744	0,2	0,5905402	0,0	0,9221962	0,1	0,851011
	EF3007		Hypothetical proteins	Conserved	conserved hypothetical protein	-0,4	0,2936651	0,8	0,0703966	-0,4	0,1970072	0,0	0,9963119	0,1	0,8492893
	EF3008		Hypothetical proteins	Conserved	conserved hypothetical protein	-0,7	0,0737621	0,7	0,113051	-0,1	0,7389217	0,1	0,8093792	0,2	0,624919
	EF3009		Hypothetical proteins	Conserved	conserved hypothetical protein	0,8	0,0341117	0,6	0,1844187	0,3	0,3384595	0,8	0,0248573	0,8	0,0168707

Operon[a]	Locus	Gene	Functional category	Subcategory	Putative function	Blood[b]	P-value[c]	YTB_5[b]	P-value[c]	YTB_15[b]	P-value[c]	YTB_30[b]	P-value[c]	YTB_60[b]	P-value[c]
	EF3010		Hypothetical proteins	Conserved	conserved hypothetical protein	-1,8	**0,0000012**	-1,7	0,0003109	-0,3	0,3679189	0,6	0,0801326	-0,2	0,4989374
	EF3011		Hypothetical proteins	Conserved	conserved hypothetical protein	-0,3	0,4163093	-0,9	0,0473769	0,3	0,4092815	0,4	0,1877758	0,1	0,6845101
	EF3012		Cell envelope	Other	membrane protein, putative	0,2	0,7026974	-0,2	0,6462379	0,2	0,5671674	-0,1	0,7387841	0,1	0,8196346
	EF3013		Hypothetical proteins	Domain	conserved domain protein	0,1	0,9032943	-0,1	0,7882641	0,4	0,2192687	0,1	0,6911491	0,2	0,4964903
	EF3014		Transport and binding proteins	Cations and iron carrying compounds	cation-transporting ATPase, E1-E2 family	-0,6	0,2165686	-0,6	0,168454	0,8	0,0148325	0,4	0,2091664	0,3	0,3542737
	EF3015		Transport and binding proteins	Unknown substrate	sodium:dicarboxylate symporter family protein	-1,7	**0,0000016**	-0,4	0,3413576	0,3	0,3792055	-0,8	0,0195447	0,2	0,535367
	EF3016		Hypothetical proteins	Conserved	conserved hypothetical protein	-1,6	0,0000201	-0,8	0,0693362	0,3	0,2986782	-0,5	0,1425898	0,0	0,9297888
	EF3017	*truA-2*	Protein synthesis	tRNA and rRNA base modification	tRNA pseudouridine synthase A	1,4	0,0023224	-0,4	0,3329279	NA	NA	0,3	0,3779301	0,4	0,2401358
	EF3018		Hypothetical proteins	Domain	conserved domain protein	NA	NA	-0,5	0,2717893	0,0	0,9980052	0,6	0,0794542	0,5	0,1154641
	EF3019		Hypothetical proteins	Conserved	conserved hypothetical protein	-1,7	**0,0000043**	-1,1	0,015852	0,1	0,8278711	-0,1	0,700719	0,0	0,9062223
	EF3020		Hypothetical proteins	Conserved	conserved hypothetical protein	-1,7	**0,0000033**	-1,1	0,0141045	0,4	0,2526847	-0,1	0,724577	0,0	0,9051065
	EF3021		Hypothetical proteins	Conserved	conserved hypothetical protein	-0,8	0,0357018	-0,2	0,66193	0,2	0,4652119	0,4	0,2327407	-0,3	0,3687701
	EF3022		Transport and binding proteins	Unknown substrate	sodium:dicarboxylate symporter family protein	NA	NA	-0,3	0,5343364	NA	NA	NA	NA	0,5	0,2625469
	EF3023		Cellular processes	Pathogenesis	polysaccharide lyase, family 8	0,5	0,1541908	0,2	0,6089998	0,3	0,3930845	0,7	0,0521256	0,0	0,9774627
	EF3024		Hypothetical proteins		hypothetical protein	0,4	0,2323855	0,6	0,2134669	0,0	0,8868839	0,2	0,5800266	0,1	0,8434532
	EF3025		Hypothetical proteins	Conserved	conserved hypothetical protein TIGR00246	-1,3	0,0004913	0,1	0,9095089	-0,1	0,8364027	-0,1	0,8128046	0,1	0,8366628
	EF3027	*htrA*	Protein fate	Degradation of proteins, peptides, and glycopeptides	serine protease DO	-1,7	**0,0000021**	-0,6	0,2337134	-0,2	0,493796	-0,4	0,1899352	0,2	0,5828715
	EF3028		Unknown function	General	putative tRNA-binding domain protein	0,4	0,3166208	0,5	0,2479468	-0,1	0,6626573	0,7	0,0454025	0,0	0,9018288
	EF3029		Signal transduction	PTS	PTS system, IID component	-2,2	**0**	-1,6	0,0007006	-1,5	**0,0000026**	0,2	0,6035551	-0,1	0,852053
	EF3030		Signal transduction	PTS	PTS system, IIC component	-0,6	0,1281157	-1,0	0,0270054	-0,1	0,7862354	0,2	0,5314831	-0,1	0,7698088
	EF3031		Signal transduction	PTS	PTS system, IIB component	-0,2	0,5224917	-0,5	0,2407561	-0,1	0,6574086	-0,5	0,1462422	0,1	0,811511
	EF3032		Signal transduction	PTS	conserved hypothetical protein	1,4	0,0001371	0,7	0,1388858	0,4	0,1873893	0,3	0,4382608	0,4	0,1763328
	EF3033		Signal transduction	PTS	PTS system, IIA component	1,0	0,006376	0,5	0,2891464	0,5	0,0973557	0,4	0,2344893	0,4	0,2378081
	EF3034		Regulatory functions	DNA interactions	transcriptional regulator, GntR family	0,3	0,4356185	0,3	0,5072892	0,9	0,0043249	0,5	0,1333416	0,3	0,3328933
	EF3035		Cellular processes	Adaptations to atypical conditions	universal stress protein family	0,5	0,1554838	-0,2	0,6352533	-0,2	0,5944494	0,1	0,7666659	-0,3	0,4426028
	EF3036		Energy metabolism	Electron transport	thioredoxin family protein	0,2	0,5567964	0,4	0,4095222	-0,2	0,4655251	-0,1	0,7671877	-0,5	0,1414366
	EF3037	*pepA*	Protein fate	Degradation of proteins, peptides, and glycopeptides	glutamyl-aminopeptidase	NA	NA	-0,6	0,324628	0,0	0,9679388	0,0	0,9686489	-0,1	0,7652284
	EF3039		Hypothetical proteins	Conserved	conserved hypothetical protein	NA	NA	NA	NA	NA	NA	NA	NA	NA	NA
	EF3040		Hypothetical proteins		hypothetical protein	NA	NA	NA	NA	NA	NA	NA	NA	NA	NA
	EF3041		Transport and binding proteins	Amino acids, peptides and amines	pheromone binding protein	0,6	0,0994933	-0,1	0,7502372	NA	NA	NA	NA	0,2	0,7044137
	EF3042		Signal transduction	PTS	PTS system, IID component	1,0	0,031477	-0,6	0,2385046	-0,4	0,2225271	NA	NA	0,0	0,9105063
	EF3043		Signal transduction	PTS	PTS system, IIC component	1,0	0,0078788	-0,4	0,4027138	0,1	0,845127	0,0	0,9679286	-0,6	0,0543855
	EF3044	*nagA-2*	Central intermediary metabolism	Amino sugars	N-acetylglucosamine-6-phosphate deacetylase	1,6	0,0000194	-0,1	0,8035035	-0,1	0,7567396	-0,8	0,0156862	-0,8	0,0165627
	EF3045		Signal transduction	PTS	PTS system, IIB component	NA	NA	NA	NA	NA	NA	NA	NA	NA	NA
	EF3046		Signal transduction	PTS	PTS system, IIA component	1,5	0,0000765	-0,1	0,7634775	0,1	0,7520213	0,2	0,4693979	-0,4	0,2460849
	EF3047		Hypothetical proteins	Conserved	conserved hypothetical protein	1,0	0,0042008	-0,3	0,5230151	0,2	0,5893811	0,0	0,8984317	0,0	0,9037401
	EF3048		Hypothetical proteins	Conserved	conserved hypothetical protein	1,0	0,0092311	0,1	0,7938531	-0,2	0,4356033	NA	NA	0,0	0,910871
	EF3049		Regulatory functions	Other	phosphosugar-binding transcriptional regulator, RpiR family	2,3	**0,0000003**	0,6	0,1850063	-0,1	0,8407829	-0,3	0,3076102	-0,5	0,1076243
	EF3050	*tag-2*	DNA metabolism	DNA replication, recombination, and repair	DNA-3-methyladenine glycosylase I	0,7	0,0744586	0,5	0,2571335	-0,2	0,5241956	-0,3	0,3886814	-0,2	0,4675919
	EF3051		Hypothetical proteins	Domain	conserved domain protein	0,7	0,0524655	0,4	0,369947	0,1	0,8649935	-0,2	0,4601424	-0,1	0,7147074
	EF3052		Hypothetical proteins		hypothetical protein	-0,6	0,0995916	-0,2	0,593159	0,2	0,5190158	0,2	0,5486873	0,4	0,2297689
	EF3053		Hypothetical proteins	Conserved	conserved hypothetical protein	-0,4	0,337351	-0,1	0,9027485	0,0	0,9273047	-0,2	0,5659403	0,2	0,5112846
	EF3054		Cell envelope	Other	lipoprotein, putative	0,5	0,1784489	0,0	0,9259849	-0,4	0,2078727	-0,4	0,2926837	-0,2	0,5539406
	EF3055		Hypothetical proteins	Conserved	conserved hypothetical protein	-0,3	0,4521842	0,9	0,0456635	0,1	0,8084615	-0,1	0,8630564	-0,5	0,1447878
	EF3056		Cell envelope	Surface structures	sortase family protein	-1,8	0,0000436	0,7	0,1388425	0,1	0,7243985	0,0	0,9165575	-0,4	0,1878807
	EF3057		Hypothetical proteins		hypothetical protein	-1,6	0,0000248	0,5	0,2550534	0,2	0,535552	0,0	0,8891403	-0,4	0,2264787
	EF3058		Regulatory functions	Protein interactions	phosphotyrosine protein phosphatase	-1,9	**0,0000002**	-0,9	0,0451708	0,1	0,8611885	0,7	0,0389914	0,0	0,9971318
	EF3059		Regulatory functions	DNA interactions	transcriptional regulator, TetR family	0,1	0,8551221	0,2	0,6184508	0,4	0,2527295	0,3	0,4149643	0,1	0,7927292
	EF3060	*salA*	Unknown function	Enzymes of unknown specificity	secreted lipase, putative	1,2	0,0015119	1,8	0,0000702	0,4	0,1676285	-0,5	0,1005822	-0,9	0,0054113
	EF3061	*mreD*	Cell envelope	Biosynthesis/degradation of murein sacculus/peptidoglycan	rod shape-determining protein MreD	0,6	0,1124479	1,4	0,0017792	0,2	0,5717152	-1,1	0,0007878	-0,9	0,0102931
	EF3062	*mreC*	Cell envelope	Biosynthesis/degradation of murein sacculus/peptidoglycan	rod shape-determining protein MreC	-0,3	0,3562717	0,9	0,0518222	0,2	0,5644735	-1,7	**0,0000004**	-0,5	0,1509575
	EF3063		Cellular processes	Toxin production and resistance	immunity protein, putative	NA	NA	NA	NA	NA	NA	NA	NA	NA	NA
	EF3064	*pnpA*	Transcription	Degradation of RNA	polyribonucleotide nucleotidyltransferase	NA	NA	0,2	0,7882396	NA	NA	NA	NA	0,4	0,2229163
	EF3065	*rpsO*	Protein synthesis	Ribosomal proteins: synthesis and modification	ribosomal protein S15	NA	NA	NA	NA	NA	NA	NA	NA	-0,4	0,3447118
	EF3066	*def-1*	Protein fate	Protein modification and repair	polypeptide deformylase	NA	NA	0,6	0,2115325	-0,2	0,5764249	0,0	0,9571785	-0,2	0,6428225
	EF3067		Unknown function	Enzymes of unknown specificity	hydrolase, haloacid dehalogenase-like family	NA	NA	0,7	0,2364774	NA	NA	NA	NA	0,1	0,8354345
	EF3068		Hypothetical proteins	Conserved	conserved hypothetical protein	1,3	0,0002863	1,4	0,0023745	0,3	0,3304361	0,5	0,1682141	0,2	0,536082
	EF3069		Transport and binding proteins	Carbohydrates, organic alcohols, and acids	formate/nitrite transporter family protein	NA	NA	0,3	0,48909	-0,4	0,2750336	-0,4	0,2173498	0,6	0,0718287
	EF3070	*rpsD*	Protein synthesis	Ribosomal proteins: synthesis and modification	ribosomal protein S4	0,5	0,25662	0,3	0,5506809	-0,4	0,2279316	0,3	0,4507141	0,4	0,2862424
	EF3071		Hypothetical proteins		hypothetical protein	NA	NA	NA	NA	NA	NA	NA	NA	NA	NA
	EF3072		Biosynthesis of cofactors, prosthetic groups, carriers	Biotin	BioY family protein	1,5	0,0000485	0,6	0,1638707	0,0	0,9189725	-0,2	0,6012499	-0,8	0,0184894
	EF3073		Protein fate	Protein and peptide secretion and trafficking	signal peptidase I	0,2	0,6495656	0,6	0,231187	0,3	0,3299329	-0,2	0,5704423	0,2	0,5759936
	EF3074		Hypothetical proteins		hypothetical protein	-1,1	0,014357	0,1	0,8815483	-0,3	0,3027706	0,1	0,8213633	-0,3	0,3972315
	EF3075		Hypothetical proteins		hypothetical protein	0,5	0,2124269	0,2	0,6316806	0,3	0,3863939	0,2	0,488448	0,0	0,9674557
	EF3076		Cell envelope	Other	cell wall surface anchor family protein	NA	NA	0,7	0,1436055	NA	NA	0,4	0,2229531	0,2	0,4981093
	EF3078		Hypothetical proteins	Conserved	conserved hypothetical protein	-0,5	0,1346499	-0,5	0,243088	-0,2	0,5366324	-0,4	0,2183952	0,0	0,8951458
	EF3079		Unknown function	Enzymes of unknown specificity	acetyltransferase, GNAT family	-2,0	**0**	-0,7	0,138068	-0,1	0,7171456	-0,5	0,1506119	0,0	0,9314671
	EF3080	*pepT-2*	Protein fate	Degradation of proteins, peptides, and glycopeptides	peptidase T	0,0	0,9770408	0,2	0,711143	0,2	0,603852	-0,4	0,2884536	0,4	0,2737084
	EF3081		Transport and binding proteins	Amino acids, peptides and amines	pheromone binding protein	NA	NA	0,5	0,2574069	NA	NA	0,1	0,6580972	0,3	0,403333
	EF3082	*fatB*	Transport and binding proteins	Cations and iron carrying compounds	iron compound ABC transporter, substrate-binding protein	3,5	**0**	-0,1	0,788728	3,4	**0**	4,9	**0**	-0,3	0,3164082
	EF3083	*ceuD*	Transport and binding proteins	Cations and iron carrying compounds	iron compound ABC transporter, ATP-binding protein	3,0	**0**	-0,2	0,6697151	2,7	**0**	4,3	**0**	0,0	0,892587
	EF3084	*ceuC*	Transport and binding proteins	Cations and iron carrying compounds	iron compound ABC transporter, permease protein	2,8	**0**	0,0	0,9873106	2,4	**0**	3,7	**0**	0,0	0,9045259
	EF3085	*ceuB*	Transport and binding proteins	Cations and iron carrying compounds	iron compound ABC transporter, permease protein	4,1	**0**	0,2	0,7204544	3,6	**0**	4,8	**0**	-0,3	0,4141179
	EF3086		Hypothetical proteins	Conserved	conserved hypothetical protein	0,4	0,2254038	0,1	0,8446768	0,4	0,2154951	0,2	0,5724439	0,3	0,3004474
	EF3087		Hypothetical proteins		hypothetical protein	0,5	0,1417305	-0,3	0,5017895	0,4	0,1983937	0,2	0,5807336	0,4	0,270256
	EF3088		Hypothetical proteins		hypothetical protein	0,5	0,1584291	-0,1	0,8238065	0,1	0,7594293	0,4	0,2372338	0,1	0,8024063
	EF3089		Biosynthesis of cofactors, prosthetic groups, carriers	Glutathione and analogs	glutamate--cysteine ligase, putative/amino acid ligase, putative	1,3	0,0006452	0,3	0,454942	0,2	0,5511976	0,4	0,2303928	NA	NA
	EF3090		Unknown function	Enzymes of unknown specificity	isochorismatase family protein	0,6	0,0952498	0,6	0,2179585	-0,1	0,7271422	0,5	0,1359218	-0,1	0,8543886
	EF3091		Unknown function	General	YitT family protein	-0,9	0,0110971	-0,3	0,5660284	0,1	0,7616989	0,2	0,4957633	-0,2	0,534296
	EF3092		Unknown function	Enzymes of unknown specificity	glyoxalase family protein	1,6	0,0000176	0,3	0,5723429	0,2	0,4591774	0,3	0,3301433	0,2	0,5973991
	EF3093		Hypothetical proteins	Conserved	conserved hypothetical protein	-1,3	0,0002236	-0,1	0,8548416	0,1	0,6967665	0,0	0,9143887	0,4	0,1818326
	EF3094	*ftsY*	Protein fate	Protein and peptide secretion and trafficking	signal recognition particle-docking protein FtsY	-2,2	**0**	-0,2	0,6042702	-0,2	0,5639772	0,2	0,6143172	0,0	0,920765
	EF3095		Unknown function	Enzymes of unknown specificity	hydrolase, haloacid dehalogenase-like family	-1,7	**0,0000025**	-0,4	0,4030666	-0,4	0,2075752	0,1	0,8721782	-0,1	0,7755499

Operon[a]	Locus	Gene	Functional category	Subcategory	Putative function	Blood[b]	P-value[c]	YTB_5[b]	P-value[c]	YTB_15[b]	P-value[c]	YTB_30[b]	P-value[c]	YTB_60[b]	P-value[c]
	EF3096		Cellular processes	Cell division	chromosome partition protein SMC	-0,1	0,7013549	-1,1	0,0143513	-0,4	0,1786578	0,2	0,5746526	-0,1	0,7508333
	EF3097	*rnc*	Transcription	RNA processing	ribonuclease III	-2,0	**0**	-0,8	0,0815239	-0,2	0,4317739	0,2	0,5127105	-0,2	0,6332069
	EF3099		Transport and binding proteins	Unknown substrate	transporter accessory protein, putative	NA	NA	-0,3	0,5979929	NA	NA	0,2	0,5124076	0,3	0,3928925
	EF3100		Mobile and extrachromosomal element functions	Transposon functions	IS256, transposase	-1,6	**0,0000114**	-1,0	0,0256671	-0,3	0,4077169	0,2	0,6028231	0,0	0,9571339
	EF3101		Hypothetical proteins	Domain	conserved domain protein	-0,6	0,1991536	0,1	0,8934134	NA	NA	NA	NA	0,1	0,7940373
	EF3102		Hypothetical proteins		hypothetical protein	0,2	0,6688533	-0,3	0,5115515	-0,6	0,1489152	NA	NA	0,4	0,2013654
	EF3103		Cell envelope	Other	membrane protein, putative	NA	NA	-0,2	0,7240467	-0,6	0,0541525	-0,2	0,4705974	0,4	0,2348053
	EF3104		Transport and binding proteins	Unknown substrate	ABC transporter, ATP-binding protein	NA	NA	0,3	0,5265436	NA	NA	0,1	0,7024348	0,4	0,1780421
	EF3105		Hypothetical proteins		hypothetical protein	NA	NA	-0,2	0,7426405	0,7	0,0574392	0,4	0,2550097	0,5	0,1488993
	EF3106		Transport and binding proteins	Amino acids, peptides and amines	peptide ABC transporter, peptide-binding protein	6,1	**0**	0,8	0,0917328	1,3	0,0000277	-0,4	0,2246696	0,9	0,0042799
	EF3107		Transport and binding proteins	Amino acids, peptides and amines	peptide ABC transporter, permease protein	5,1	**0**	1,4	0,0017986	0,9	0,0068968	-0,6	0,0741668	1,0	0,0022004
	EF3108		Transport and binding proteins	Amino acids, peptides and amines	peptide ABC transporter, permease protein	4,9	**0**	1,6	0,000519	0,8	0,0103549	-0,6	0,0657768	1,1	0,0013738
	EF3109		Transport and binding proteins	Amino acids, peptides and amines	peptide ABC transporter, ATP-binding protein	4,0	**0**	0,9	0,0625326	0,6	0,0724841	-0,5	0,1553321	1,2	0,0004831
	EF3110		Transport and binding proteins	Amino acids, peptides and amines	peptide ABC transporter, ATP-binding protein	2,8	**0**	0,9	0,0498375	1,5	**0,0000015**	0,2	0,5990876	1,4	**0,0000125**
	EF3111	*acpP*	Fatty acid and phospholipid metabolism	Biosynthesis	acyl carrier protein	-1,7	**0,0000041**	-1,0	0,0360384	-0,2	0,5075184	-0,2	0,5207745	0,1	0,8771424
	EF3112	*plsX*	Fatty acid and phospholipid metabolism	Biosynthesis	fatty acid/phospholipid synthesis protein PlsX	-1,4	0,0001812	-0,8	0,0788107	-0,2	0,5366208	-0,2	0,5860823	-0,2	0,5305064
	EF3113	*recG*	DNA metabolism	DNA replication, recombination, and repair	ATP-dependent DNA helicase RecG	-1,8	**0,0000007**	-0,8	0,0831103	-0,3	0,3139074	-0,4	0,2904932	-0,1	0,8260986
	EF3114		Unknown function	General	DAK2 domain protein	-0,3	0,463956	-0,3	0,5486234	-0,2	0,5239245	-0,2	0,6002721	-0,2	0,521414
	EF3115		Hypothetical proteins	Conserved	conserved hypothetical protein	-0,5	0,1693238	-0,3	0,5016318	-0,2	0,438826	-0,1	0,8302548	-0,1	0,6684262
	EF3116	*rpmB*	Protein synthesis	Ribosomal proteins: synthesis and modification	ribosomal protein L28	-3,0	**0**	-1,3	0,0042483	-0,3	0,2766213	-0,3	0,4191969	-0,1	0,8070698
	EF3117		Unknown function	Enzymes of unknown specificity	thiamin pyrophosphokinase family protein	-0,8	0,0240941	-0,4	0,4095135	0,0	0,966589	-0,2	0,6308977	-0,1	0,8654017
	EF3118	*rpe*	Energy metabolism	Pentose phosphate pathway	ribulose-phosphate 3-epimerase	-1,0	0,0064501	-0,3	0,4680169	0,0	0,9739766	0,0	0,9802876	-0,1	0,8736203
	EF3119		Hypothetical proteins	Conserved	conserved hypothetical protein TIGR00157	-0,6	0,1207616	-0,2	0,60193	0,3	0,3897933	0,1	0,7106213	0,1	0,7752282
	EF3120		Regulatory functions	Protein interactions	serine/threonine protein kinase	0,0	0,9753191	-0,4	0,3926142	-0,2	0,4967276	-0,3	0,4503714	0,1	0,671979
	EF3121		Unknown function	Enzymes of unknown specificity	protein phosphatase 2C, putative	-2,4	**0**	-0,4	0,3453491	-0,2	0,4816266	-0,5	0,1457817	-0,1	0,7402648
	EF3122	*sun*	Protein synthesis	tRNA and rRNA base modification	Sun protein	-1,9	**0,0000002**	-0,7	0,1158052	-0,2	0,5058514	-0,5	0,1320302	-0,1	0,8207207
	EF3123	*fmt*	Protein synthesis	tRNA aminoacylation	methionyl-tRNA formyltransferase	-2,4	**0**	-1,0	0,0299248	0,1	0,778511	-0,2	0,4999284	0,0	0,9656812
	EF3124	*def-2*	Protein fate	Protein modification and repair	polypeptide deformylase, authentic frameshift	-1,6	**0,0000116**	-0,9	0,0414031	-0,2	0,6255295	0,0	0,9200302	0,1	0,7072276
	EF3125	*priA*	DNA metabolism	DNA replication, recombination, and repair	primosomal protein n	-0,9	0,0127298	-0,8	0,0895157	-0,3	0,3103504	-0,2	0,6416629	0,0	0,9999949
	EF3126	*rpoZ*	Transcription	DNA-dependent RNA polymerase	DNA-directed RNA polymerase, omega subunit	-1,3	0,0006268	-1,0	0,0295417	-0,3	0,3523704	0,0	0,9407818	0,3	0,3055826
	EF3127	*gmk-2*	Purines, pyrimidines, nucleosides, and nucleotides	Nucleotide and nucleoside interconversions	guanylate kinase	-2,0	**0,0000056**	-1,0	0,0347792	-0,2	0,5837124	0,4	0,184607	0,3	0,4470867
	EF3129		Cellular processes	Toxin production and resistance	D-alanyl-D-alanine carboxypeptidase	-1,1	0,0038947	-0,1	0,8366555	0,4	0,1981062	0,5	0,1694728	0,3	0,3845165
	EF3130		Hypothetical proteins		hypothetical protein	0,9	0,0119325	-0,1	0,8089746	0,4	0,2429432	0,7	0,0386201	0,2	0,5038929
	EF3131		Hypothetical proteins	Conserved	conserved hypothetical protein TIGR00255	0,9	0,0143738	-0,2	0,6889711	0,0	0,9280179	0,4	0,2260982	0,0	0,9492437
	EF3132		Hypothetical proteins	Conserved	conserved hypothetical protein	0,7	0,0459615	-0,3	0,5863239	0,1	0,8336665	0,4	0,2085591	0,3	0,41951
	EF3133		Hypothetical proteins	Conserved	conserved hypothetical protein	0,2	0,5701436	0,0	0,9320228	0,3	0,2897306	0,8	0,0265126	0,5	0,1101705
	EF3134	*eda-2*	Energy metabolism	Entner-Doudoroff	2-dehydro-3-deoxyphosphogluconate aldolase	6,5	**0**	0,4	0,4140104	0,1	0,8197157	0,9	0,0048585	-0,1	0,6879506
	EF3135		Energy metabolism	Sugars	mannonate dehydratase, putative	5,0	**0**	0,5	0,314282	0,2	0,4869437	1,0	0,0026204	0,2	0,6032824
	EF3136		Signal transduction	PTS	PTS system, IIA component	5,3	**0**	0,4	0,4525465	1,0	0,0010171	1,2	0,000277	0,4	0,2063283
	EF3137		Signal transduction	PTS	PTS system, IIB component	6,2	**0**	1,7	0,0003433	1,2	0,0001665	1,3	0,0000582	0,4	0,2315248
	EF3138		Signal transduction	PTS	PTS system, IID component	5,8	**0**	2,2	**0,0000021**	1,6	0,0000449	1,3	0,0000604	0,5	0,1735168
	EF3139		Signal transduction	PTS	PTS system, IIC component	5,1	**0**	2,2	**0,000002**	1,3	0,000069	0,9	0,0053555	0,6	0,0852824
	EF3140		Energy metabolism	Fermentation	alcohol dehydrogenase, iron-containing	5,0	**0**	2,4	**0,0000001**	1,3	0,0000284	1,2	0,0002133	1,2	0,000283
	EF3141		Unknown function	Enzymes of unknown specificity	D-isomer specific 2-hydroxyacid dehydrogenase family protein	3,5	**0**	1,7	0,0001656	1,1	0,0002711	1,1	0,0008128	1,3	0,0000769
	EF3142		Energy metabolism	Sugars	6-phosphogluconate dehydrogenase family protein	3,2	**0**	1,4	0,0033472	1,1	0,0006835	0,9	0,0063357	1,3	0,0000756
	EF3144		Regulatory functions	DNA interactions	phosphosugar-binding transcriptional regulator, RpiR family	1,1	0,0017888	0,6	0,269195	0,4	0,3159363	-0,1	0,7816556	0,0	0,8982551
	EF3145		Hypothetical proteins		hypothetical protein	NA	NA	-0,4	0,362784	0,5	0,1537514	0,5	0,1731292	0,2	0,5566232
	EF3146		Hypothetical proteins		hypothetical protein	0,0	0,9997974	0,5	0,3260819	0,4	0,2082937	0,7	0,0541666	0,2	0,5909895
	EF3148	*pgsA*	Fatty acid and phospholipid metabolism	Biosynthesis	CDP-diacylglycerol-glycerol-3-phosphate 3-phosphatidyltransferase	0,0	0,9210969	-0,1	0,7495169	0,2	0,6109272	0,5	0,1507495	0,1	0,6616314
	EF3149		Hypothetical proteins	Domain	conserved domain protein	-0,7	0,0651238	-0,2	0,6021163	0,1	0,8037368	0,4	0,231259	0,0	0,9010225
	EF3150		Protein fate	Degradation of proteins, peptides, and glycopeptides	peptidase, M16 family	-0,1	0,6937257	0,1	0,841251	0,2	0,5071663	0,5	0,1631757	-0,1	0,7284391
	EF3151		Hypothetical proteins	Conserved	conserved hypothetical protein	0,0	0,9935874	-0,3	0,5566218	0,1	0,7430001	0,3	0,4104594	0,2	0,6079548
	EF3152	*mscL*	Cellular processes	Adaptations to atypical conditions	large conductance mechanosensitive channel protein	1,4	0,0001802	0,5	0,2370483	0,6	0,0722727	0,5	0,1609641	-0,2	0,4700988
	EF3153		Hypothetical proteins	Conserved	conserved hypothetical protein	0,2	0,632889	0,1	0,7633436	0,1	0,6655529	0,2	0,5468578	0,2	0,593048
	EF3154		Hypothetical proteins	Conserved	conserved hypothetical protein	0,0	0,9308471	0,1	0,8762683	-0,2	0,6051596	0,4	0,1992126	0,2	0,6201758
	EF3155		Hypothetical proteins	Conserved	conserved hypothetical protein	NA	NA	0,2	0,6305886	NA	NA	NA	NA	0,1	0,6545684
	EF3156		Regulatory functions	DNA interactions	transcriptional regulator, GntR family	0,1	0,7137909	0,3	0,4753591	0,5	0,1234296	0,7	0,0315031	0,4	0,2576502
	EF3157		Energy metabolism	Biosynthesis and degradation of polysaccharides	glycosyl hydrolase, family 65	-0,9	0,0093279	1,7	0,0002604	0,9	0,0064093	0,8	0,0169346	-0,1	0,7619742
	EF3158		Unknown function	Enzymes of unknown specificity	hydrolase, haloacid dehalogenase-like family	0,0	0,9941944	1,4	0,0024561	1,0	0,0020686	1,3	0,0001488	-0,2	0,6324365
	EF3160		Hypothetical proteins		hypothetical protein	NA	NA	0,1	0,8339116	NA	NA	1,0	0,0189119	0,5	0,1648036
	EF3161		Hypothetical proteins		hypothetical protein	NA	NA	0,7	0,1959678	NA	NA	NA	NA	0,0	0,9121115
	EF3163	*prsA-2*	Purines, pyrimidines, nucleosides, and nucleotides	Purine ribonucleotide biosynthesis	ribose-phosphate pyrophosphokinase	-0,9	0,0172168	-1,4	0,0027366	-0,4	0,1753688	-0,1	0,7207867	-0,3	0,3130592
	EF3164	*msrB*	Unknown function	General	PilB family protein	2,1	**0**	0,2	0,653752	0,6	0,0488543	0,1	0,7557983	-0,1	0,8744307
	EF3165	*maf*	Cellular processes	Cell division	maf protein	-1,0	0,0056136	-0,2	0,6082624	0,0	0,9822526	-0,3	0,4292457	0,1	0,6598935
	EF3166	*hexB*	DNA metabolism	DNA replication, recombination, and repair	DNA mismatch repair protein HexB	-0,6	0,1254756	-0,3	0,5054076	0,0	0,9816032	0,0	0,9908637	0,2	0,5597052
	EF3167	*hexA*	DNA metabolism	DNA replication, recombination, and repair	DNA mismatch repair protein HexA	-1,1	0,002543	-0,2	0,6329999	-0,1	0,8684284	-0,3	0,4480153	-0,1	0,8189525
	EF3168		Hypothetical proteins	Conserved	conserved hypothetical protein	-0,3	0,4724852	-0,5	0,2549953	-0,4	0,1979811	0,2	0,5966429	-0,2	0,5327966
	EF3169		Hypothetical proteins	Conserved	conserved hypothetical protein TIGR00282	-0,6	0,1249491	-0,3	0,5856259	0,0	0,989413	0,3	0,4489076	0,3	0,4414769
	EF3170		Unknown function	General	HD/KH domain protein	-0,4	0,3329378	-0,1	0,7888138	-0,1	0,7864997	0,1	0,8689737	-0,3	0,3445478
	EF3171	*recA*	DNA metabolism	DNA replication, recombination, and repair	recA protein	1,4	0,0000758	0,2	0,7392885	0,0	0,9654163	0,0	0,9435293	-0,4	0,2088239
	EF3172	*cinA*	DNA metabolism	DNA replication, recombination, and repair	competence/damage-inducible protein CinA	-1,1	0,0028097	-0,6	0,2009564	0,2	0,4621214	0,4	0,2235767	-0,4	0,2349374
	EF3173		Hypothetical proteins		hypothetical protein	-1,9	**0,0000001**	-0,4	0,3752697	-1,4	**0,0000069**	-1,1	0,0009087	-0,1	0,7164105
	EF3174		Hypothetical proteins	Conserved	conserved hypothetical protein	1,0	0,0067512	-0,3	0,5650284	-0,4	0,2393948	-0,1	0,6816189	0,0	0,9822587
	EF3175		Unknown function	General	rrf2 family protein	-0,1	0,6988078	-0,4	0,3771156	-0,3	0,2743745	0,1	0,6970295	-0,2	0,5176651
	EF3176		Cell envelope	Other	membrane protein, putative	-0,4	0,3796291	-0,3	0,553838	-0,3	0,3490019	0,2	0,654346	-0,2	0,4884272
	EF3177		Hypothetical proteins	Conserved	conserved hypothetical protein	0,1	0,7508833	-0,2	0,658246	0,3	0,356964	0,1	0,6910204	-0,2	0,4871941
	EF3178		Protein fate	Degradation of proteins, peptides, and glycopeptides	peptidase, M20/M25/M40 family	1,7	**0,0000023**	0,3	0,4592838	0,0	0,9638455	0,5	0,1654106	-0,2	0,5749349
	EF3179		Hypothetical proteins	Conserved	conserved hypothetical protein	3,0	**0**	0,4	0,4296942	-0,2	0,5039326	0,3	0,3346881	0,2	0,5811538
	EF3180	*sigV*	Transcription	Transcription factors	RNA polymerase sigma-70 factor, ECF subfamily	2,2	**0**	0,1	0,8088962	0,2	0,6028126	0,5	0,1444129	0,3	0,3170458
	EF3181		Hypothetical proteins		hypothetical protein	-1,1	0,0039089	0,1	0,7532285	0,2	0,5309342	-0,1	0,8101058	0,2	0,6099337
	EF3182		Hypothetical proteins		hypothetical protein	NA	NA	-0,2	0,7369278	0,0	0,9026933	0,0	0,8845613	0,3	0,3409795
	EF3183		Cell envelope	Other	cell wall surface anchor family protein, putative	NA	NA	-0,1	0,7559698	NA	NA	0,0	0,9856777	0,4	0,2443471
	EF3184		Hypothetical proteins	Conserved	conserved hypothetical protein	-0,6	0,1490241	0,0	0,9958638	0,2	0,5468461	0,1	0,7294739	0,5	0,1014945

Operon[a)]	Locus	Gene	Functional category	Subcategory	Putative function	Blood[b)]	P-value[c)]	YTB_5[b)]	P-value[c)]	YTB_15[b)]	P-value[c)]	YTB_30[b)]	P-value[c)]	YTB_60[b)]	P-value[c)]
	EF3185		Hypothetical proteins	Conserved	conserved hypothetical protein	0,5	0,2085649	0,0	0,950343	0,1	0,7768504	0,0	0,9679994	0,4	0,2958014
	EF3186		Hypothetical proteins	Conserved	conserved hypothetical protein	0,5	0,1990696	0,3	0,5580954	-0,2	0,5553732	0,1	0,7531732	0,5	0,1682901
	EF3187		Cell envelope	Other	cell wall surface anchor family protein	NA	NA	0,1	0,8994602	NA	NA	0,4	0,1930291	0,2	0,498343
	EF3188		Hypothetical proteins	Conserved	conserved hypothetical protein	0,2	0,6844225	0,3	0,5454134	0,3	0,3657838	0,1	0,7475933	0,6	0,0620483
	EF3189		Hypothetical proteins		hypothetical protein	1,3	0,0034508	0,1	0,7819263	NA	NA	0,2	0,664431	0,9	0,006108
	EF3191		Fatty acid and phospholipid metabolism	Degradation	lipase, putative	2,3	**0**	0,8	0,1031345	0,2	0,4468667	0,2	0,580164	1,5	**0,0000061**
	EF3192		Unknown function	Enzymes of unknown specificity	isochorismatase family protein	3,2	**0**	1,1	0,0178899	0,7	0,0360149	0,7	0,0311372	1,7	**0,0000002**
	EF3193	*lrgB*	Unknown function	General	LrgB family protein	7,9	**0**	2,7	**0**	2,3	**0**	2,4	**0**	5,3	**0**
	EF3194	*lrgA*	Unknown function	General	LrgA family protein	5,6	**0**	2,2	**0,0000013**	2,1	**0**	2,1	**0**	4,7	**0**
	EF3196		Signal transduction	Two-component systems	response regulator	1,1	0,002783	0,5	0,2637048	1,4	0,0000163	0,7	0,0318901	0,5	0,1355002
	EF3197		Signal transduction	Two-component systems	sensor histidine kinase	1,1	0,004309	0,8	0,0803815	1,1	0,0006614	0,9	0,0072858	0,2	0,5236816
	EF3198		Cell envelope	Other	lipoprotein, YaeC family	3,2	**0**	1,3	0,0037146	2,3	**0**	1,9	**0**	1,2	0,0003124
	EF3199		Transport and binding proteins	Unknown substrate	ABC transporter, permease protein	2,8	**0**	1,7	0,0002662	2,4	**0**	1,6	**0,0000025**	1,5	**0,0000041**
	EF3200		Transport and binding proteins	Unknown substrate	ABC transporter, ATP-binding protein	2,3	**0**	1,3	0,0045422	2,5	**0**	1,5	**0,000007**	1,3	0,0001273
	EF3201		Unknown function	General	OsmC/Ohr family protein	NA	NA	-0,7	0,1455591	0,1	0,7321718	0,5	0,1443456	0,0	0,9596242
	EF3202	*rpsN-3*	Protein synthesis	Ribosomal proteins: synthesis and modification	ribosomal protein S14	NA	NA	-0,9	0,0499339	0,8	0,0302998	0,4	0,2676224	0,2	0,6487419
	EF3203	*rpmG-4*	Protein synthesis	Ribosomal proteins: synthesis and modification	ribosomal protein L33	0,6	0,1893975	0,1	0,8329264	0,3	0,2866488	NA	NA	0,2	0,5698738
	EF3204		Unknown function	General	cobalamin synthesis protein/P47K family protein	0,4	0,3615397	-0,6	0,1706142	0,4	0,2542442	0,3	0,3315674	-0,2	0,5279146
	EF3205		Hypothetical proteins	Conserved	conserved hypothetical protein	NA	NA	0,2	0,6618489	NA	NA	NA	NA	0,5	0,1579124
	EF3206		Cell envelope	Other	adhesion lipoprotein	-1,9	0,0000171	-1,4	0,0018616	0,4	0,2661361	0,2	0,4981136	0,3	0,4107097
	EF3207		Unknown function	Enzymes of unknown specificity	dihydrouridine synthase family protein	-2,0	**0,0000001**	-1,1	0,0137309	0,2	0,5595124	0,1	0,7715628	-0,1	0,6833291
	EF3208		Transport and binding proteins	Unknown substrate	ABC transporter, permease protein	-1,7	**0,0000086**	-0,2	0,5922521	0,1	0,8232817	-0,3	0,4357052	-0,2	0,4939618
	EF3209		Transport and binding proteins	Unknown substrate	ABC transporter, ATP-binding protein	-2,1	**0,0000001**	-0,8	0,0864407	0,0	0,9310675	-0,1	0,6721052	-0,2	0,4895426
	EF3210		Signal transduction	PTS	PTS system, IIA component, putative	NA	NA	0,3	0,6314888	-0,7	0,0350484	NA	NA	0,5	0,1579134
	EF3211		Signal transduction	PTS	PTS system, IIB component	NA	NA	0,2	0,6947561	NA	NA	NA	NA	0,4	0,257364
	EF3212		Signal transduction	PTS	PTS system, IIC component	NA	NA	NA	NA	NA	NA	NA	NA	0,2	0,5684113
	EF3213		Signal transduction	PTS	PTS system, IID component	NA	NA	NA	NA	NA	NA	NA	NA	0,4	0,3750511
	EF3214		Transcription	Other	ATP-dependent helicase, DEAH-box family, putative	-1,3	0,0010155	-0,7	0,1216826	0,4	0,2591042	0,2	0,5733556	0,2	0,6176521
	EF3215		Mobile and extrachromosomal element functions	Transposon functions	IS256, transposase	-1,6	**0,0000153**	-0,9	0,0446461	-0,3	0,4058478	0,0	0,8833675	0,0	0,9993258
	EF3216		Regulatory functions	Other	transcriptional regulator, putative	0,2	0,6154955	-0,1	0,7942654	-0,2	0,4405552	-0,1	0,6893946	0,1	0,6961185
	EF3217		Unknown function	Enzymes of unknown specificity	helicase, putative	-0,2	0,6286063	0,5	0,3173248	-0,1	0,8015575	-0,2	0,6218327	-0,2	0,5583857
	EF3218		DNA metabolism	DNA replication, recombination, and repair	mutator MutT protein, putative	-0,3	0,5760141	0,0	0,9304158	0,3	0,3753531	0,1	0,8611549	0,0	0,9768799
	EF3220		Hypothetical proteins		hypothetical protein	-0,6	0,1060165	0,0	0,9579862	0,3	0,3402587	0,3	0,4345842	0,1	0,7058161
	EF3221		Regulatory functions	DNA interactions	transcriptional regulator, Cro/CI family	0,4	0,2435088	-0,2	0,69234	0,1	0,7166715	0,0	0,8871619	0,0	0,9546631
	EF3222		Hypothetical proteins		hypothetical protein	NA	NA	NA	NA	NA	NA	NA	NA	0,3	0,3018616
	EF3223		Hypothetical proteins		hypothetical protein	NA	NA	0,0	0,9181638	NA	NA	NA	NA	0,0	0,889169
	EF3224		Hypothetical proteins		hypothetical protein	NA	NA	0,0	0,9197521	NA	NA	0,0	0,9438414	0,2	0,5189756
	EF3225		Hypothetical proteins	Conserved	conserved hypothetical protein	NA	NA	NA	NA	1,0	0,0072149	-0,1	0,8824453	0,1	0,8803111
	EF3226		DNA metabolism	DNA replication, recombination, and repair	Rep protein	NA	NA	NA	NA	NA	NA	0,4	0,2313322	0,2	0,6725633
	EF3227		Hypothetical proteins	Conserved	conserved hypothetical protein	0,8	0,0281704	-0,2	0,7152312	NA	NA	NA	NA	0,1	0,7595708
	EF3230	*rpsI*	Protein synthesis	Ribosomal proteins: synthesis and modification	ribosomal protein S9	-3,4	**0**	-1,5	0,0014262	-0,2	0,5224972	-0,5	0,1041426	-0,1	0,746816
	EF3232		Hypothetical proteins		hypothetical protein	NA	NA	NA	NA	NA	NA	NA	NA	NA	NA
	EF3233		Cellular processes	Adaptations to atypical conditions	Dps family protein	1,5	0,0000284	0,5	0,3094709	-1,2	0,0001038	-0,4	0,2082484	0,0	0,9172584
	EF3234		Cell envelope	Other	lipoprotein, putative	NA	NA	-0,4	0,4502498	-0,2	0,4590795	0,0	0,9929144	0,3	0,3436151
	EF3235		Energy metabolism	Sugars	gluconate kinase, putative	1,2	0,0059575	0,1	0,81131	0,4	0,2479677	0,3	0,4475397	0,1	0,7767823
	EF3236		Protein fate	Protein and peptide secretion and trafficking	type III leader peptidase family	NA	NA	0,2	0,7553278	0,1	0,8144129	-0,1	0,8401373	0,2	0,5443221
	EF3237	*rpoC*	Transcription	DNA-dependent RNA polymerase	DNA-directed RNA polymerase, beta-prime subunit	-2,9	**0**	-0,4	0,3706786	-0,1	0,8505603	-0,8	0,0129292	0,2	0,6401573
	EF3238	*rpoB*	Transcription	DNA-dependent RNA polymerase	DNA-directed RNA polymerase, beta subunit	-3,3	**0**	-0,9	0,0626286	-0,3	0,3406758	-0,8	0,0231983	-0,2	0,618291
	EF3239		Hypothetical proteins	Conserved	conserved hypothetical protein	1,6	**0,0000065**	1,3	0,00455	0,0	0,8851068	0,2	0,5090699	0,7	0,0349276
	EF3240	*birA*	Regulatory functions	DNA interactions	BirA bifunctional protein	1,1	0,0156884	-0,7	0,1204211	0,0	0,9111114	0,0	0,9587848	0,3	0,3197816
	EF3241		Cellular processes	Other	abortive phage resistance protein, putative	0,0	0,975265	0,0	0,992458	0,2	0,4600941	0,0	0,9428124	0,1	0,8124905
	EF3242		Cellular processes	Other	abortive phage resistance protein, putative	0,2	0,6441903	0,1	0,8317573	0,3	0,3821634	-0,2	0,5776827	0,2	0,4557496
	EF3243		Hypothetical proteins		hypothetical protein	NA	NA	-0,1	0,9207294	NA	NA	0,0	0,9323526	0,4	0,2447514
	EF3244		Hypothetical proteins		hypothetical protein	NA	NA	NA	NA	NA	NA	NA	NA	NA	NA
	EF3245		Unknown function	Enzymes of unknown specificity	cell-envelope associated acid phosphatase	-1,8	**0,0000006**	-0,6	0,219078	-0,2	0,5970248	-1,3	0,0015311	-0,6	0,0656224
	EF3247		Hypothetical proteins		hypothetical protein	0,9	0,0546514	-0,3	0,4614403	0,0	0,8776354	0,3	0,4177586	0,5	0,1327825
	EF3248		Hypothetical proteins		hypothetical protein	NA	NA	0,0	0,9244749	-0,1	0,7904699	-0,1	0,7327661	0,2	0,5367226
	EF3249		Hypothetical proteins	Conserved	conserved hypothetical protein	NA	NA	NA	NA	NA	NA	NA	NA	NA	NA
	EF3250		Hypothetical proteins		hypothetical protein	NA	NA	-0,4	0,4037488	0,4	0,2420013	0,5	0,1785877	0,1	0,7028482
	EF3251		Hypothetical proteins		hypothetical protein	NA	NA	0,3	0,4623081	-0,2	0,5364755	NA	NA	0,7	0,0281921
	EF3252		Hypothetical proteins		hypothetical protein	0,4	0,2304157	-0,2	0,6203389	0,4	0,2523487	0,2	0,5360169	-0,2	0,4917508
	EF3253		Cell envelope	Other	cell wall surface anchor family protein	-0,5	0,2257198	0,1	0,8753819	0,9	0,0046929	0,7	0,02643	-0,1	0,6908939
	EF3254		Biosynthesis of cofactors, prosthetic groups, carriers	Menaquinone and ubiquinone	1,4-dihydroxy-2-naphthoate octaprenyltransferase, putative	-3,2	**0**	-1,2	0,0120973	-1,5	**0,0000009**	-0,8	0,0207641	0,5	0,1560049
	EF3255		Cell envelope	Other	thiamin biosynthesis lipoprotein ApbE, putative	-3,5	**0**	-1,3	0,0044606	-1,2	0,0001233	-0,6	0,0541028	0,6	0,0818852
	EF3256		Cell envelope	Other	pheromone cAD1 precursor lipoprotein	-2,9	**0**	-1,8	0,0000908	-2,1	**0**	-0,3	0,3937153	-0,1	0,8687187
	EF3257		Unknown function	Enzymes of unknown specificity	oxidoreductase, pyridine nucleotide-disulfide family	-4,4	**0**	-1,8	0,0000673	-1,4	**0,0000107**	-0,8	0,0234589	-0,4	0,2793139
	EF3258		Hypothetical proteins	Conserved	conserved hypothetical protein	NA	NA	-0,3	0,4633688	-1,0	0,0011371	0,2	0,6201273	0,3	0,3633299
	EF3259		Hypothetical proteins	Domain	conserved domain protein	-1,5	0,0000318	-1,1	0,0136875	-0,8	0,0075484	0,0	0,9535719	0,0	0,9798631
	EF3260		Biosynthesis of cofactors, prosthetic groups, carriers	Menaquinone and ubiquinone	heptaprenyl diphosphate synthase, component II, putative	-2,0	**0**	-0,9	0,0601581	-0,7	0,0277315	-0,3	0,3021568	-0,1	0,754081
	EF3261		Regulatory functions	DNA interactions	transcriptional regulator, AbrB family	NA	NA	0,1	0,9113037	-0,4	0,2548704	-0,3	0,3317726	0,5	0,1094304
	EF3262		Regulatory functions	DNA interactions	transcriptional regulator, PemK family	NA	NA	0,2	0,6759596	0,0	0,9689659	0,3	0,3354704	0,5	0,124856
	EF3263		Hypothetical proteins	Conserved	conserved hypothetical protein	NA	NA	NA	NA	NA	NA	NA	NA	NA	NA
	EF3264		Hypothetical proteins		hypothetical protein	NA	NA	NA	NA	NA	NA	NA	NA	NA	NA
	EF3265	*folP*	Biosynthesis of cofactors, prosthetic groups, carriers	Folic acid	dihydropteroate synthase	0,1	0,7043546	-0,1	0,8565764	0,1	0,6808942	-0,1	0,6783596	0,0	0,9590059
	EF3266		Unknown function	General	Ham1 family protein, putative	NA	NA	-0,1	0,9123168	0,6	0,0493233	0,6	0,0742588	-0,2	0,6278876
	EF3267	*folE*	Biosynthesis of cofactors, prosthetic groups, carriers	Folic acid	GTP cyclohydrolase I	0,5	0,1650077	-0,2	0,6391324	0,3	0,3246492	0,4	0,2533618	0,0	0,9129084
	EF3268	*folK*	Biosynthesis of cofactors, prosthetic groups, carriers	Folic acid	hydroxymethyldihydropteridine pyrophosphokinase	0,1	0,7282941	-0,2	0,6230878	0,5	0,0960737	0,4	0,2243301	-0,2	0,5788467
	EF3269	*folB*	Biosynthesis of cofactors, prosthetic groups, carriers	Folic acid	dihydroneopterin aldolase	0,7	0,1137054	0,0	0,9476332	0,2	0,5463369	0,2	0,4759747	0,0	0,9490487
	EF3270	*gor*	Cellular processes	Detoxification	glutathione reductase	-0,7	0,0545732	-0,4	0,4437002	-0,1	0,7094273	-0,2	0,5462922	-0,4	0,2289631
	EF3271		Hypothetical proteins		hypothetical protein	-1,7	**0,0000026**	-0,4	0,3461197	-0,1	0,7436466	-0,7	0,0379785	-0,4	0,2849019
	EF3272		Regulatory functions	DNA interactions	zinc-binding transcriptional regulator, Cro/CI family	-1,6	0,0000205	-0,4	0,4068582	0,0	0,9842408	-0,8	0,0183536	-0,4	0,2892785
	EF3273		Hypothetical proteins		hypothetical protein	-1,3	0,0025859	-0,6	0,1673909	0,5	0,1032196	-0,2	0,6376531	-0,2	0,5105072
	EF3274		Hypothetical proteins		hypothetical protein	NA	NA	-0,9	0,0535583	0,3	0,3623092	-0,1	0,754991	-0,1	0,7331709

Operon[a]	Locus	Gene	Functional category	Subcategory	Putative function	Blood[b]	P-value[c]	YTB_5[b]	P-value[c]	YTB_15[b]	P-value[c]	YTB_30[b]	P-value[c]	YTB_60[b]	P-value[c]
	EF3275		Central intermediary metabolism	Other	hydantoinase/oxoprolinase	0,2	0,7247025	NA	NA	NA	NA	NA	NA	NA	NA
	EF3276		Hypothetical proteins	Conserved	conserved hypothetical protein	0,0	0,976126	-0,9	0,051804	-0,3	0,35522	-0,4	0,2537514	0,1	0,7588165
	EF3277		Transport and binding proteins	Nucleosides, purines and pyrimidines	cytosine permease, putative	NA	NA	0,7	0,2020956	NA	NA	0,5	0,1419227	0,0	0,9035214
	EF3278		Hypothetical proteins	Conserved	conserved hypothetical protein	-1,9	**0,0000003**	-0,7	0,1402867	-0,9	0,0028726	-0,8	0,0119115	0,1	0,7877286
	EF3279		Protein fate	Degradation of proteins, peptides, and glycopeptides	peptidase, U32 family	-2,8	**0**	-1,2	0,0108557	-1,5	**0,0000038**	-0,8	0,0229972	0,0	0,9117604
	EF3280		Protein fate	Degradation of proteins, peptides, and glycopeptides	peptidase, U32 family, putative	-3,1	**0**	-1,2	0,0109354	-1,3	0,0000255	-0,5	0,1137006	-0,2	0,5607124
	EF3281		Hypothetical proteins	Domain	conserved domain protein	-0,6	0,0963024	-0,6	0,1853257	-0,7	0,0270717	0,0	0,9587613	0,0	0,9417063
	EF3282	*clpC*	Protein fate	Degradation of proteins, peptides, and glycopeptides	ATP-dependent Clp protease, ATP-binding subunit ClpC	1,8	**0,0000015**	0,5	0,3123905	-0,1	0,8566419	-0,4	0,2053359	-0,3	0,3249685
	EF3283	*ctsR*	Regulatory functions	DNA interactions	transcriptional regulator CtsR	0,9	0,0217005	-0,1	0,7880942	0,0	0,9246635	0,1	0,7528773	-0,5	0,1127332
	EF3284		Amino acid biosynthesis	Aspartate family	cystathionine gamma-synthase, putative	0,0	0,9929805	-0,3	0,4964716	-0,2	0,4480364	-0,1	0,6868051	-0,2	0,5557766
	EF3285		Signal transduction	PTS	PTS system, IIC component	NA	NA	0,5	0,3524796	NA	NA	0,5	0,2562381	-0,1	0,8362217
	EF3286		Regulatory functions	Other	phosphosugar-binding transcriptional regulator, putative	0,1	0,8321416	0,1	0,8650476	0,4	0,2566605	0,6	0,0787759	0,1	0,6808281
	EF3287		Hypothetical proteins		hypothetical protein	NA	NA	NA	NA	NA	NA	NA	NA	NA	NA
	EF3289		Regulatory functions	DNA interactions	DNA-binding response regulator	0,5	0,1655374	0,2	0,6742578	-0,2	0,4509612	-0,3	0,362933	0,0	0,931433
	EF3290		Signal transduction	Two-component systems	sensor histidine kinase	0,7	0,0477246	0,1	0,8200605	0,0	0,9638728	-0,3	0,3466813	0,0	0,8887371
	EF3292	*serS-2*	Protein synthesis	tRNA aminoacylation	seryl-tRNA synthetase	0,2	0,498198	0,3	0,456697	-0,2	0,4473493	-0,8	0,0140903	0,1	0,7451479
	EF3293	*guaB*	Purines, pyrimidines, nucleosides, and nucleotides	Purine ribonucleotide biosynthesis	inosine-5`-monophosphate dehydrogenase	-3,2	**0**	-1,5	0,0013458	-0,5	0,1113722	-0,4	0,2675634	-0,3	0,3903481
	EF3294		Cell envelope	Other	membrane protein, putative	-3,3	**0**	-0,4	0,3871996	-0,1	0,6354409	-1,0	0,0035444	0,2	0,6055827
	EF3295		Hypothetical proteins	Conserved	conserved hypothetical protein	-3,3	**0**	-0,9	0,048554	-0,5	0,1345047	-0,8	0,0179813	-0,1	0,804967
	EF3296		Unknown function	General	GTP-binding protein, GTP1/OBG family	-3,1	**0**	-1,2	0,0095566	-0,4	0,1924331	-0,6	0,0761699	-0,3	0,4205254
	EF3297		Hypothetical proteins	Conserved	conserved hypothetical protein	NA	NA	-1,4	0,002171	-0,2	0,4825014	-0,2	0,5374896	-0,2	0,5989251
	EF3298		Cellular processes	Cell division	chromosome partitioning protein ParB family	-3,8	**0**	-1,2	0,0094563	-0,3	0,3364142	-0,5	0,1058719	-0,1	0,8053766
	EF3299		Cellular processes	Cell division	ATPase, ParA family	-3,7	**0**	-1,5	0,001214	-0,2	0,443706	-0,6	0,0926412	-0,2	0,5288975
	EF3300	*gidB*	Unknown function	General	glucose-inhibited division protein B	-2,6	**0**	-1,4	0,0018636	-0,3	0,3624376	-0,2	0,6455505	0,1	0,837671
	EF3301		Hypothetical proteins		hypothetical protein	-0,2	0,5545176	0,7	0,1139927	0,0	0,9665214	0,1	0,7365031	-0,1	0,8354443
	EF3302		Hypothetical proteins		hypothetical protein	0,3	0,5369983	-0,4	0,4206057	0,4	0,1839153	0,0	0,9272249	0,2	0,4964892
	EF3303		Hypothetical proteins	Conserved	conserved hypothetical protein	-0,5	0,19042	-1,8	0,0001508	-2,5	**0**	-1,7	**0,0000002**	-1,0	0,0025689
	EF3304	*mipB*	Energy metabolism	Pentose phosphate pathway	transaldolase-like protein MIPB	5,1	**0**	-0,1	0,8498826	0,6	0,060904	1,4	0,0000486	-1,0	0,0029438
	EF3305		Signal transduction	PTS	PTS system, sorbitol-specific IIA component	3,4	**0**	-0,2	0,7125753	0,8	0,0104594	1,3	0,0000986	-0,9	0,0086659
	EF3306		Signal transduction	PTS	PTS system, sorbitol-specific IIBC components	5,3	**0**	0,3	0,6255994	0,6	0,0650039	1,4	0,0000362	-1,0	0,0031049
	EF3307		Signal transduction	PTS	PTS system, sorbitol-specific IIC component	NA	NA	1,4	0,0022319	0,8	0,0157728	0,9	0,0049296	-1,0	0,0017301
	EF3308	*srlR*	Regulatory functions	DNA interactions	transcriptional regulator SrlR	3,8	**0**	NA	NA	1,5	**0,0000032**	0,9	0,00805	-1,0	0,0036885
	EF3309	*srlM*	Regulatory functions	DNA interactions	putative transcriptional activator SrlM	3,4	**0**	2,0	0,0000187	1,5	**0,0000017**	0,8	0,0172619	-0,9	0,0043422
	EF3310		Unknown function	Enzymes of unknown specificity	oxidoreductase, short chain dehydrogenase/reductase family	3,7	**0**	2,1	**0,0000085**	1,1	0,0003146	0,9	0,0092248	-1,0	0,0022745
	EF3311	*gidA*	Unknown function	General	glucose-inhibited division protein A	-2,0	**0**	-0,3	0,5119361	-0,2	0,5893498	-1,3	0,0001328	0,1	0,7260863
	EF3312	*trmE*	Cellular processes	Detoxification	tRNA modification GTPase TrmE	-1,3	0,0003452	-0,4	0,3730346	0,0	0,910293	-1,3	0,0001705	0,3	0,4391998
	EF3313		Hypothetical proteins		hypothetical protein	NA	NA	-0,2	0,7406956	0,0	0,9048122	0,0	0,9546729	0,0	0,8840757
	EF3314		Cell envelope	Other	cell wall surface anchor family protein	NA	NA	NA	NA	NA	NA	NA	NA	NA	NA
	EF3315		Unknown function	General	CitG family protein	1,9	**0,0000003**	0,0	0,9785373	0,5	0,1104261	-0,3	0,3780042	-0,2	0,6295305
	EF3316		Unknown function	Enzymes of unknown specificity	malic enzyme family protein	2,7	**0**	-0,1	0,8436154	0,3	0,3402951	-0,2	0,5041006	0,1	0,7644568
	EF3317		Unknown function	Enzymes of unknown specificity	carboxylase, putative	4,3	**0**	0,3	0,4750769	-0,2	0,4341335	0,0	0,9343468	-0,5	0,1086375
	EF3318	*citX*	Energy metabolism	Fermentation	apo-citrate lyase pyrophosphoribosyl-dephosph-CoA transferase	NA	NA	NA	NA	NA	NA	NA	NA	-0,4	0,1846264
	EF3319	*citF*	Energy metabolism	Fermentation	citrate lyase, alpha subunit	4,5	**0**	0,3	0,5662729	-0,2	0,4829566	-0,3	0,3429266	-0,5	0,1180668
	EF3320	*citE*	Energy metabolism	Fermentation	citrate lyase, beta subunit	4,8	**0**	0,3	0,5143915	-0,3	0,3395282	-0,2	0,5540846	-0,6	0,0558877
	EF3321	*citD*	Energy metabolism	Fermentation	citrate lyase, gamma subunit	3,2	**0**	-0,4	0,4090066	0,0	0,8950578	-0,1	0,8287849	-0,7	0,0385291
	EF3322	*citC*	Energy metabolism	Fermentation	citrate lyase ligase	4,3	**0**	0,0	0,9763858	0,0	0,9782641	-0,1	0,78189	-0,6	0,0500117
	EF3323		Hypothetical proteins	Conserved	conserved hypothetical protein	NA	NA	NA	NA	NA	NA	NA	NA	NA	NA
	EF3324		Energy metabolism	Other	sodium ion-translocating decarboxylase, beta subunit	4,3	**0**	0,6	0,2343886	-0,3	0,3947611	-0,2	0,4915024	-0,8	0,0131291
	EF3325		Energy metabolism	Other	sodium ion-translocating decarboxylase/biotin carboxyl carrier protei	4,3	**0**	0,3	0,5051259	-0,5	0,1220518	-0,3	0,3505912	-0,8	0,0163384
	EF3326		Hypothetical proteins	Conserved	conserved hypothetical protein	NA	NA	-0,2	0,7194784	NA	NA	0,1	0,7461413	-0,5	0,1671045
	EF3327		Transport and binding proteins	Carbohydrates, organic alcohols, and acids	citrate transporter	3,3	**0**	0,1	0,7760882	-0,4	0,2391724	-0,8	0,019296	-1,2	0,0004184
	EF3328		Regulatory functions	DNA interactions	transcriptional regulator, GntR family	2,3	**0**	-0,5	0,2464567	0,4	0,176894	-0,2	0,5671216	-0,9	0,0092867
	EF3329		Regulatory functions	DNA interactions	DNA-binding response regulator	-1,0	0,0046019	-0,3	0,5615561	0,2	0,4361878	-0,1	0,6967352	0,2	0,5213007
	EF3330		Unknown function	General	jag protein, putative	-0,5	0,1845145	-0,3	0,503776	0,0	0,9589828	0,2	0,5241051	0,1	0,6589896
	EF3331		Cell envelope	Other	pheromone cCF10 percursor/lipoprotein, 60 kDa	-0,4	0,2648379	-0,4	0,4390717	0,1	0,7049122	0,0	0,9924105	0,1	0,8143468
	EF3332	*rnpA*	Transcription	RNA processing	ribonuclease P protein component	-1,2	0,0009712	-0,5	0,2999193	0,3	0,3940955	0,3	0,3887942	0,4	0,2319669
	EF3333	*rpmH*	Protein synthesis	Ribosomal proteins: synthesis and modification	ribosomal protein L34	-2,2	**0,0000011**	-1,1	0,0173668	0,0	0,9407903	-0,4	0,230387	0,3	0,296025
	EFA0001	*repA-1*	Mobile and extrachromosomal element functions	Plasmid functions	replication-associated protein RepA	-0,7	0,0460906	0,1	0,7596056	0,1	0,6491767	0,0	0,884514	-0,2	0,6438472
	EFA0002	*traB-1*	Mobile and extrachromosomal element functions	Plasmid functions	pheromone shutdown protein TraB	-0,1	0,9025325	0,1	0,8971507	0,3	0,3617061	-0,4	0,2208143	0,1	0,8688725
	EFA0003	*traC-1*	Mobile and extrachromosomal element functions	Plasmid functions	traC protein	0,1	0,8576252	-0,4	0,3346001	0,5	0,1260596	-0,2	0,6490269	0,2	0,4595357
	EFA0004	*traA*	Mobile and extrachromosomal element functions	Plasmid functions	traA protein	NA	NA	0,1	0,8768461	NA	NA	NA	NA	0,3	0,4313899
	EFA0005.	*iad*	Cellular processes	Other	sex pheromone inhibitor determinant	NA	NA	NA	NA	NA	NA	NA	NA	NA	NA
	EFA0006		Mobile and extrachromosomal element functions	Transposon functions	IS1216, transposase	-0,7	0,0505497	0,4	0,356103	-0,1	0,7770799	0,0	0,9108907	-0,1	0,7025729
	EFA0007		Cellular processes	Toxin production and resistance	ribosomal RNA adenine dimethylase family protein	-1,0	0,0084152	-0,5	0,2809215	-0,2	0,4574603	0,4	0,23976	0,1	0,8082818
	EFA0008		Disrupted reading frame		plasmid copy control protein, truncation	NA	NA	NA	NA	NA	NA	NA	NA	NA	NA
	EFA0009		Mobile and extrachromosomal element functions	Transposon functions	IS1216, transposase	-0,2	0,4961032	0,5	0,267926	0,2	0,513017	0,5	0,1247593	0,0	0,9495088
	EFA0010		Transport and binding proteins	Other	multidrug resistance protein	-0,1	0,8410432	0,4	0,4298246	0,4	0,1701017	0,3	0,3356264	0,0	0,9878294
	EFA0012		Mobile and extrachromosomal element functions	Plasmid functions	replication protein	0,2	0,5123585	-0,2	0,7385947	-0,3	0,4186186	0,3	0,3087598	0,3	0,3096997
	EFA0013		Mobile and extrachromosomal element functions	Transposon functions	IS1216, transposase	-0,7	0,0589994	0,6	0,2265185	0,1	0,8263597	0,2	0,581552	-0,2	0,6142383
	EFA0014		Transport and binding proteins	Other	drug resistance transporter, putative	0,2	0,5082665	0,1	0,7527886	0,0	0,879813	-0,2	0,6367387	-0,2	0,5956573
	EFA0015		Hypothetical proteins		hypothetical protein	NA	NA	-0,3	0,5336247	0,4	0,2629507	0,3	0,4462249	0,0	0,9232757
	EFA0016		Mobile and extrachromosomal element functions	Transposon functions	transposase, IS6 family	NA	NA	NA	NA	NA	NA	NA	NA	NA	NA
	EFA0017		Hypothetical proteins		hypothetical protein	0,6	0,0810746	0,0	0,9157268	-0,5	0,0837159	0,1	0,8466494	-0,4	0,19272
	EFA0019	*ssb-4*	DNA metabolism	DNA replication, recombination, and repair	single-strand binding protein	NA	NA	0,6	0,2183477	0,8	0,0416611	0,2	0,5284907	0,1	0,8773609
	EFA0020		Disrupted reading frame		conserved hypothetical protein, truncation	-0,7	0,1031453	-0,1	0,77355	0,1	0,7397723	-0,3	0,3252565	0,2	0,5764839
	EFA0021		Hypothetical proteins	Domain	conserved domain protein	NA	NA	-0,3	0,4705945	0,8	0,0287718	0,0	0,8962109	0,3	0,3568699
	EFA0022		Hypothetical proteins	Domain	conserved domain protein	NA	NA	0,3	0,4950079	0,9	0,005555	1,1	0,0007163	1,5	**0,0000095**
	EFA0024		Hypothetical proteins		hypothetical protein	1,3	0,0041647	0,2	0,7095018	0,0	0,8864889	1,0	0,0044449	0,3	0,3983347
	EFA0025		Hypothetical proteins		hypothetical protein	NA	NA	-0,2	0,7873385	NA	NA	NA	NA	0,4	0,2751467
	EFA0026		Hypothetical proteins		hypothetical protein	NA	NA	-0,6	0,2309603	NA	NA	NA	NA	0,2	0,5518848
	EFA0028		Hypothetical proteins	Domain	conserved domain protein	NA	NA	NA	NA	NA	NA	NA	NA	NA	NA
	EFA0029		Hypothetical proteins		hypothetical protein	NA	NA	0,1	0,8061181	NA	NA	NA	NA	0,2	0,588607
	EFA0030		Hypothetical proteins	Conserved	conserved hypothetical protein	0,2	0,5279012	0,0	0,9593449	NA	NA	NA	NA	0,5	0,1624629

Operon[a]	Locus	Gene	Functional category	Subcategory	Putative function	Blood[b]	P-value[c]	YTB_5[b]	P-value[c]	YTB_15[b]	P-value[c]	YTB_30[b]	P-value[c]	YTB_60[b]	P-value[c]
	EFA0031		Hypothetical proteins		hypothetical protein	NA	NA	0,6	0,2371313	-0,3	0,4529051	0,0	0,9308293	-0,1	0,7614119
	EFA0032		Cell envelope	Other	lipoprotein, putative	0,1	0,7351262	-0,1	0,8377194	0,0	0,9197954	0,4	0,2353048	0,3	0,3952519
	EFA0033		Hypothetical proteins	Conserved	conserved hypothetical protein	NA	NA	NA	NA	NA	NA	NA	NA	NA	NA
	EFA0034	*ssb-5*	DNA metabolism	DNA replication, recombination, and repair	single-strand binding protein	-0,6	0,2134374	-0,3	0,5334646	0,7	0,0249889	1,1	0,0012248	-0,1	0,8311656
	EFA0035		Hypothetical proteins	Conserved	conserved hypothetical protein	NA	NA	0,0	0,9594925	NA	NA	NA	NA	0,2	0,4628603
	EFA0036		Hypothetical proteins	Conserved	conserved hypothetical protein	0,7	0,1080505	0,0	0,99554	0,2	0,5700075	NA	NA	0,4	0,2099264
	EFA0037		Hypothetical proteins	Conserved	conserved hypothetical protein	NA	NA	0,6	0,2879981	NA	NA	NA	NA	-0,2	0,6173052
	EFA0038		Hypothetical proteins	Conserved	conserved hypothetical protein	NA	NA	0,2	0,7677915	NA	NA	NA	NA	-0,2	0,6395452
	EFA0039		Hypothetical proteins	Conserved	conserved hypothetical protein	NA	NA	NA	NA	NA	NA	NA	NA	0,2	0,5884543
	EFA0040		Hypothetical proteins		hypothetical protein	NA	NA	NA	NA	NA	NA	NA	NA	0,6	0,1457021
	EFA0041		Hypothetical proteins	Conserved	conserved hypothetical protein	NA	NA	NA	NA	NA	NA	NA	NA	0,2	0,6504831
	EFA0042		Cellular processes	Conjugation	cell wall surface anchor signal protein	1,1	0,0137309	-0,2	0,7733283	-0,2	0,4519963	NA	NA	0,4	0,2849029
	EFA0043		Hypothetical proteins	Conserved	conserved hypothetical protein	NA	NA	NA	NA	NA	NA	NA	NA	0,3	0,3097365
	EFA0044		Hypothetical proteins		hypothetical protein	NA	NA	NA	NA	NA	NA	NA	NA	0,0	0,8919377
	EFA0045		Hypothetical proteins	Conserved	conserved hypothetical protein	NA	NA	NA	NA	NA	NA	NA	NA	NA	NA
	EFA0046		Hypothetical proteins	Conserved	conserved hypothetical protein	NA	NA	NA	NA	NA	NA	NA	NA	NA	NA
	EFA0047	*asa1*	Cellular processes	Conjugation	aggregation substance Asa1	NA	NA	0,1	0,854517	NA	NA	NA	NA	0,4	0,1868371
	EFA0048		Hypothetical proteins	Conserved	conserved hypothetical protein	NA	NA	0,4	0,4979454	0,3	0,4056877	0,9	0,0277808	0,8	0,0217938
	EFA0050		Hypothetical proteins		hypothetical protein	NA	NA	0,3	0,5466426	NA	NA	NA	NA	NA	NA
	EFA0051		Hypothetical proteins		hypothetical protein	NA	NA	-0,7	0,1315589	NA	NA	NA	NA	0,4	0,2480388
	EFA0052	*sea1*	Cellular processes	Conjugation	surface exclusion protein Sea1	0,6	0,1089229	-0,6	0,2134478	0,6	0,0412206	0,8	0,0144468	0,0	0,9516962
	EFA0053		Hypothetical proteins		hypothetical protein	0,3	0,3831626	-0,3	0,6482271	0,2	0,4564096	0,3	0,3111652	-0,1	0,777553
	EFA0054	*traE1*	Regulatory functions	Other	regulatory protein TraE1	0,3	0,381801	-0,1	0,7572753	0,0	0,9067583	0,0	0,8867161	0,0	0,9285355
	EFA0056		Mobile and extrachromosomal element functions	Transposon functions	IS1216, transposase	-0,5	0,1826288	0,5	0,3179604	-0,1	0,7143256	0,3	0,4121129	0,1	0,8459304
	EFA0057	*copS*	Mobile and extrachromosomal element functions	Plasmid functions	copS protein	NA	NA	NA	NA	NA	NA	NA	NA	NA	NA
	EFA0058	*repE*	Mobile and extrachromosomal element functions	Plasmid functions	repE protein	-0,7	0,0608616	0,0	0,9583658	-0,2	0,4482511	0,8	0,0202049	-0,1	0,6857015
	EFA0059		Mobile and extrachromosomal element functions	Transposon functions	IS256, transposase	NA	NA	NA	NA	NA	NA	NA	NA	NA	NA
	EFA0060		Unknown function	Enzymes of unknown specificity	acetyltransferase, GNAT family	-0,9	0,0188497	0,1	0,9070024	-0,6	0,058518	-0,6	0,0803492	-0,1	0,6604751
	EFA0061		Cellular processes	Toxin production and resistance	6-aminoglycoside N-acetyltransferase	-1,8	**0,000001**	-0,2	0,6035804	-0,8	0,010152	-0,8	0,0232151	-0,1	0,7685054
	EFA0062		Mobile and extrachromosomal element functions	Transposon functions	IS256, transposase	-1,5	0,0000616	-0,9	0,0447626	-0,5	0,1289868	0,1	0,8254324	0,0	0,9794264
	EFA0063		Mobile and extrachromosomal element functions	Transposon functions	IS1216, transposase	-0,3	0,3699342	0,5	0,2736895	0,1	0,6925138	0,5	0,1058108	0,1	0,8673284
	EFA0065		Hypothetical proteins	Conserved	conserved hypothetical protein	-0,1	0,7912416	-0,2	0,6170292	0,0	0,9007122	-0,1	0,8687508	0,3	0,3407857
	EFA0066		Hypothetical proteins	Conserved	conserved hypothetical protein	0,0	0,8926046	-0,3	0,5233455	-0,2	0,4580601	-0,3	0,390336	0,3	0,4329781
	EFA0067		Signal transduction	PTS	PTS system, IIABC components	-0,2	0,6484226	0,4	0,4255127	0,2	0,5461947	-0,5	0,1122298	-0,2	0,5284265
	EFA0069	*scrB-2*	Energy metabolism	Biosynthesis and degradation of polysaccharides	sucrose-6-phosphate hydrolase	-0,3	0,4848811	0,5	0,3170173	0,3	0,3135283	-0,2	0,5958141	-0,2	0,6404329
	EFA0070	*scrR-2*	Regulatory functions	DNA interactions	sucrose operon repressor ScrR	-0,1	0,7004501	0,5	0,2403774	0,4	0,2433627	-0,1	0,711375	0,0	0,8915341
	EFA0071		Regulatory functions	DNA interactions	PemK family protein	0,8	0,0398316	0,8	0,0943829	0,1	0,7500047	0,0	0,9097463	0,7	0,0364243
	EFA0072		Unknown function	General	PemI family protein	1,1	0,0017736	1,0	0,0324882	-0,1	0,7823503	0,0	0,9777283	0,6	0,0540699
	EFA0073		DNA metabolism	DNA replication, recombination, and repair	site-specific recombinase, resolvase family	NA	NA	0,1	0,8556585	0,0	0,9175006	-0,1	0,8607115	0,2	0,4960215
	EFA0074		Hypothetical proteins	Domain	conserved domain protein	-0,1	0,6969421	0,4	0,3969602	0,0	0,9626374	-0,1	0,8558503	0,2	0,5281976
	EFA0075		Hypothetical proteins		hypothetical protein	NA	NA	0,4	0,4060219	0,2	0,5687609	-0,2	0,5438459	0,2	0,4515316
	EFA0076		Hypothetical proteins		hypothetical protein	NA	NA	-0,2	0,6537077	0,2	0,4636806	0,3	0,4200578	0,1	0,6805604
	EFA0078		DNA metabolism	DNA replication, recombination, and repair	ImpB/MucB/SamB family protein	0,8	0,0346608	-0,2	0,6202958	-0,5	0,127842	-0,6	0,0895473	-0,3	0,3234793
	EFA0079		Hypothetical proteins	Conserved	conserved hypothetical protein	1,2	0,0055263	0,0	0,9243235	0,0	0,9493998	-0,8	0,015703	-0,3	0,3008056
	EFA0080		Regulatory functions	Other	transcriptional regulator, UvrC family	0,4	0,2514109	0,4	0,4339981	-0,3	0,3315414	NA	NA	0,0	0,9434123
	EFA0081		Hypothetical proteins	Conserved	conserved hypothetical protein	0,0	0,915854	0,1	0,8169619	-0,2	0,5756586	-0,2	0,4638617	-0,1	0,812187
	EFA0082	*repC*	Mobile and extrachromosomal element functions	Plasmid functions	replication-associated protein RepC	-0,5	0,1723828	0,2	0,7256552	0,0	0,9131195	0,5	0,1169804	0,2	0,626181
	EFA0083	*repB-1*	Mobile and extrachromosomal element functions	Plasmid functions	replication-associated protein RepB	-0,5	0,1780345	0,2	0,6211763	0,1	0,6593918	0,0	0,9949586	-0,1	0,7378448
	EFB0001	*repA-2*	Mobile and extrachromosomal element functions	Plasmid functions	replication-associated protein RepA	-0,2	0,5897127	0,9	0,0603474	-0,1	0,8311397	-0,5	0,1005937	0,0	0,9313287
	EFB0003	*traB-2*	Mobile and extrachromosomal element functions	Plasmid functions	pheromone shutdown protein TraB	NA	NA	0,4	0,396398	0,1	0,6762177	-0,4	0,2663193	0,4	0,214522
	EFB0004	*traC-2*	Mobile and extrachromosomal element functions	Plasmid functions	TraC protein	0,4	0,3072153	0,6	0,2023703	0,0	0,9599625	-0,1	0,7925108	0,1	0,8273715
	EFB0005		Regulatory functions	DNA interactions	transcriptional regulator, Cro/CI family	-2,7	**0**	-1,1	0,0436128	-0,2	0,5727591	0,4	0,2543238	-0,2	0,5065207
	EFB0007		Mobile and extrachromosomal element functions	Plasmid functions	probable pheromone-responsive regulatory protein R	NA	NA	0,0	0,9896319	0,3	0,3404014	0,9	0,00875	0,5	0,1720777
	EFB0008		Hypothetical proteins		hypothetical protein	0,9	0,0123025	0,1	0,7476978	0,2	0,5050144	0,3	0,4007711	0,0	0,8975441
	EFB0009		Hypothetical proteins	Conserved	conserved hypothetical protein	NA	NA	NA	NA	NA	NA	NA	NA	NA	NA
	EFB0010	*prgA*	Cellular processes	Conjugation	surface exclusion protein PrgA	1,1	0,0034921	0,2	0,6496594	0,1	0,6980535	0,7	0,0334804	-0,4	0,2458947
	EFB0011	*prgB*	Cellular processes	Conjugation	aggregation substance PrgB	NA	NA	NA	NA	NA	NA	NA	NA	NA	NA
	EFB0012	*prgC*	Cellular processes	Conjugation	surface protein PrgC	1,1	0,0050597	0,5	0,3927742	NA	NA	NA	NA	-0,6	0,0752372
	EFB0013		Hypothetical proteins	Conserved	conserved hypothetical protein	NA	NA	0,7	0,189123	NA	NA	NA	NA	-0,9	0,0062898
	EFB0014		Hypothetical proteins	Conserved	conserved hypothetical protein	NA	NA	0,6	0,1745609	NA	NA	0,4	0,1833633	-0,6	0,158909
	EFB0015		Hypothetical proteins		hypothetical protein	NA	NA	0,8	0,088865	NA	NA	0,1	0,7630675	-0,3	0,4174575
	EFB0016		Hypothetical proteins		hypothetical protein	NA	NA	0,7	0,1211613	NA	NA	0,3	0,3024845	-0,4	0,2912303
	EFB0017		Hypothetical proteins	Conserved	conserved hypothetical protein	NA	NA	NA	NA	NA	NA	NA	NA	-1,2	0,0030488
	EFB0018		Hypothetical proteins		hypothetical protein	NA	NA	0,8	0,172787	NA	NA	NA	NA	-0,1	0,7901484
	EFB0019		Hypothetical proteins	Domain	conserved domain protein	NA	NA	0,0	0,9337764	NA	NA	NA	NA	0,0	0,9267825
	EFB0020		Hypothetical proteins	Domain	conserved domain protein	NA	NA	-0,1	0,8464007	0,0	0,9004752	0,0	0,8872291	0,1	0,8386662
	EFB0021		Hypothetical proteins	Conserved	conserved hypothetical protein	NA	NA	0,4	0,4734882	NA	NA	0,2	0,4663249	-0,2	0,5354717
	EFB0022		Hypothetical proteins		hypothetical protein	NA	NA	0,2	0,7012011	NA	NA	NA	NA	0,0	0,8898231
	EFB0023		Hypothetical proteins		hypothetical protein	NA	NA	-0,3	0,5058493	NA	NA	0,1	0,817897	0,2	0,5089558
	EFB0024		Hypothetical proteins		hypothetical protein	NA	NA	NA	NA	NA	NA	NA	NA	NA	NA
	EFB0025		Mobile and extrachromosomal element functions	Plasmid functions	TraG family protein	NA	NA	0,2	0,6241984	0,3	0,4439448	0,2	0,5483207	0,3	0,3831238
	EFB0026		Hypothetical proteins	Domain	conserved domain protein	NA	NA	-0,7	0,1610069	NA	NA	NA	NA	-0,2	0,6125283
	EFB0027		Unknown function	General	LtrD-related protein, putative	NA	NA	NA	NA	NA	NA	NA	NA	0,1	0,7671049
	EFB0029		Hypothetical proteins	Conserved	conserved hypothetical protein	NA	NA	-0,3	0,4862469	NA	NA	0,1	0,8802048	-0,5	0,1344523
	EFB0030		Mobile and extrachromosomal element functions	Plasmid functions	relaxase	NA	NA	NA	NA	NA	NA	NA	NA	-0,7	0,0956964
	EFB0031		Hypothetical proteins		hypothetical protein	NA	NA	NA	NA	NA	NA	NA	NA	NA	NA
	EFB0032		Hypothetical proteins		hypothetical protein	NA	NA	0,2	0,5943338	0,3	0,3495298	0,4	0,2198626	0,3	0,3055383
	EFB0033		Hypothetical proteins	Conserved	conserved hypothetical protein	NA	NA	0,0	0,9712822	NA	NA	0,0	0,9556483	-0,3	0,4438066
	EFB0034		Hypothetical proteins	Domain	conserved domain protein	NA	NA	-0,1	0,8832749	0,6	0,1491489	0,0	0,957102	0,2	0,4595811
	EFB0035		Hypothetical proteins		hypothetical protein	NA	NA	NA	NA	NA	NA	NA	NA	0,3	0,3314089
	EFB0036		Hypothetical proteins		hypothetical protein	NA	NA	-1,1	0,0141558	0,6	0,079441	-0,2	0,475253	0,4	0,1994613
	EFB0037		Hypothetical proteins		hypothetical protein	NA	NA	-0,1	0,7516862	0,9	0,0188171	0,4	0,3121791	0,1	0,7919575
	EFB0038		Hypothetical proteins		hypothetical protein	NA	NA	0,6	0,2691476	0,5	0,1974826	1,1	0,0015967	0,4	0,2596073

Operon[a]	Locus	Gene	Functional category	Subcategory	Putative function	Blood[b]	P-value[c]	YTB_5[b]	P-value[c]	YTB_15[b]	P-value[c]	YTB_30[b]	P-value[c]	YTB_60[b]	P-value[c]
	EFB0040		Hypothetical proteins		hypothetical protein	NA	NA	NA	NA	NA	NA	NA	NA	NA	NA
	EFB0041		Hypothetical proteins		hypothetical protein	NA	NA	NA	NA	NA	NA	NA	NA	0,1	0,7451322
	EFB0042		Hypothetical proteins		hypothetical protein	NA	NA	NA	NA	NA	NA	0,1	0,8207641	-0,5	0,1163383
	EFB0043	*ssb-6*	DNA metabolism	DNA replication, recombination, and repair	single-strand binding protein	NA	NA	0,2	0,7511786	NA	NA	NA	NA	0,1	0,6909048
	EFB0044		Hypothetical proteins		hypothetical protein	-2,4	**0,0000001**	-0,9	0,0586431	-0,2	0,4679891	-0,2	0,5047786	0,2	0,4926042
	EFB0045	*nuc-2*	DNA metabolism	DNA replication, recombination, and repair	thermonuclease precursor	NA	NA	NA	NA	NA	NA	NA	NA	NA	NA
	EFB0046		Hypothetical proteins		hypothetical protein	NA	NA	0,4	0,3696988	NA	NA	0,3	0,3916595	0,1	0,7341663
	EFB0047		Cell envelope	Other	membrane protein, putative	NA	NA	0,5	0,3169164	NA	NA	0,2	0,5904056	0,0	0,9902497
	EFB0048		Regulatory functions	DNA interactions	transcriptional regulator, Cro/CI family	-0,2	0,5433153	0,9	0,0477507	0,1	0,6419856	-0,2	0,613013	0,1	0,874855
	EFB0049		Cell envelope	Biosynthesis and degradation of surface poly/liposaccharides	glycosyl transferase, group 2 family protein	NA	NA	NA	NA	NA	NA	NA	NA	NA	NA
	EFB0050		Transport and binding proteins	Other	toxin ABC transporter, ATP-binding/permease protein	NA	NA	NA	NA	NA	NA	NA	NA	0,1	0,8705922
	EFB0051		Cell envelope	Other	membrane protein, putative	NA	NA	NA	NA	NA	NA	NA	NA	0,1	0,6827128
	EFB0052		Mobile and extrachromosomal element functions	Transposon functions	IS256, transposase	1,0	0,0095654	-2,4	0,0000348	NA	NA	0,0	0,9160308	-0,7	0,1079376
	EFB0053		Hypothetical proteins	Domain	conserved domain protein	-1,5	0,0000428	-0,9	0,0544544	-0,3	0,3471096	-0,2	0,6484385	0,0	0,9669786
	EFB0054		Hypothetical proteins	Conserved	conserved hypothetical protein	NA	NA	NA	NA	NA	NA	NA	NA	NA	NA
	EFB0055		Cell envelope	Other	lipoprotein, putative	NA	NA	NA	NA	NA	NA	NA	NA	NA	NA
	EFB0056		Hypothetical proteins		hypothetical protein	NA	NA	-0,1	0,873714	NA	NA	0,0	0,8840611	0,1	0,7273
	EFB0057		Hypothetical proteins		hypothetical protein	0,4	0,4136242	0,2	0,6496148	0,3	0,3855395	-0,1	0,8213764	0,1	0,7409803
	EFB0058		DNA metabolism	DNA replication, recombination, and repair	site-specific recombinase, resolvase family	NA	NA	NA	NA	NA	NA	NA	NA	NA	NA
	EFB0059		Hypothetical proteins		hypothetical protein	NA	NA	0,1	0,8327535	0,0	0,9869061	-0,3	0,377421	0,4	0,2601144
	EFB0060		DNA metabolism	DNA replication, recombination, and repair	ImpB/MucB/SamB family protein	-2,5	**0**	-0,8	0,0903105	-0,2	0,5507547	-0,4	0,2071019	0,3	0,3269225
	EFB0061		Hypothetical proteins		hypothetical protein	0,5	0,2099284	-0,7	0,1391325	-0,5	0,0925772	-1,2	0,0002651	-0,5	0,1366561
	EFB0062		Regulatory functions	Other	transcriptional regulator, UvrC family	-0,6	0,1567785	-1,0	0,028653	-0,2	0,4943488	-0,2	0,5718503	-0,5	0,1080444
	EFB0063	*prgN*	Mobile and extrachromosomal element functions	Plasmid functions	replication control protein PrgN	-0,1	0,8580654	-1,1	0,0167413	-0,5	0,1187711	-0,6	0,0810685	-0,7	0,0282554
	EFB0064		Cellular processes	Cell division	ParA family protein	NA	NA	-0,5	0,3091908	0,0	0,9101949	-0,1	0,7355839	-0,2	0,6034853
	EFB0065		Hypothetical proteins		hypothetical protein	0,2	0,588812	0,3	0,4739595	-0,2	0,5428375	-0,2	0,4677036	0,1	0,6545309
	EFC0001	*prgZ*	Transport and binding proteins	Amino acids, peptides and amines	pheromone binding protein	1,6	**0,0000141**	0,9	0,057072	0,3	0,3752046	-0,2	0,5768025	0,1	0,7924546
	EFC0003		Hypothetical proteins	Conserved	conserved hypothetical protein, degenerate	NA	NA	0,0	0,9726009	-0,1	0,6926505	0,0	0,9216191	0,3	0,3708881
	EFC0003		Mobile and extrachromosomal element functions	Transposon functions	IS1216, transposase	-0,1	0,7350407	0,4	0,3952693	0,2	0,4894645	0,5	0,1799926	0,1	0,8468862
	EFC0004		Mobile and extrachromosomal element functions	Transposon functions	IS256, transposase	-1,3	0,0002399	-0,8	0,0706457	-0,4	0,2666801	0,3	0,4214406	-0,1	0,8668159
	EFC0005		Unknown function	General	restriction endonuclease related protein	NA	NA	-0,1	0,7538879	0,0	0,9199124	NA	NA	-0,2	0,520073
	EFC0006		DNA metabolism	DNA replication, recombination, and repair	ImpB/MucB/SamB family protein	1,6	**0,0000132**	-0,1	0,8108889	0,3	0,3323517	-0,2	0,4811861	-0,5	0,1218181
	EFC0007		Mobile and extrachromosomal element functions	Transposon functions	IS1216, transposase	NA	NA	NA	NA	NA	NA	NA	NA	NA	NA
	EFC0008		Hypothetical proteins	Domain	conserved domain protein	NA	NA	NA	NA	NA	NA	NA	NA	NA	NA
	EFC0009		DNA metabolism	DNA replication, recombination, and repair	site-specific recombinase, resolvase family	-1,0	0,0358275	-0,2	0,726415	-0,1	0,6889615	-0,1	0,7948999	0,2	0,5339938
	EFC0010		Hypothetical proteins	Conserved	conserved hypothetical protein	-1,5	0,00076	-0,8	0,1006262	0,4	0,2506224	0,0	0,9725644	0,4	0,2680744
	EFC0011		Regulatory functions	Other	transcriptional regulator, UvrC family	NA	NA	-0,2	0,75255	NA	NA	NA	NA	0,2	0,5246108
	EFC0012		Hypothetical proteins	Conserved	conserved hypothetical protein	NA	NA	NA	NA	NA	NA	NA	NA	NA	NA
	EFC0013		Hypothetical proteins		hypothetical protein	NA	NA	-0,6	0,2385785	NA	NA	NA	NA	0,4	0,2636796
	EFC0014		Hypothetical proteins		hypothetical protein	NA	NA	0,3	0,5974371	NA	NA	0,0	0,9644348	0,3	0,2995857
	EFC0015		DNA metabolism	DNA replication, recombination, and repair	ImpB/MucB/SamB family protein	0,9	0,020836	-0,7	0,1501104	-0,3	0,3669508	-1,0	0,0036375	-0,5	0,168525
	EFC0016		Hypothetical proteins		hypothetical protein	1,5	0,0000254	-0,5	0,2748994	-0,1	0,8481864	-0,7	0,0551091	-0,2	0,5504617
	EFC0017		Hypothetical proteins		hypothetical protein	-0,2	0,6505605	-0,1	0,9145627	-0,2	0,5892331	NA	NA	0,5	0,1547567
	EFC0018	*repB-2*	Mobile and extrachromosomal element functions	Plasmid functions	replication-associated protein RepB	0,3	0,4769997	1,2	0,0096162	0,1	0,8641647	0,3	0,3980773	0,7	0,023686
	EFC0019		Mobile and extrachromosomal element functions	Plasmid functions	repS protein, putative	-2,6	**0**	-0,5	0,2539406	0,9	0,0068538	0,3	0,5023887	0,1	0,7506908
